# Integration of palladium-catalyzed C–N coupling into self-encoded libraries for accelerated hit discovery

**DOI:** 10.1039/d5cb00303b

**Published:** 2026-03-03

**Authors:** Edith van der Nol, Zhenshuo Luo, Qing Qing Gao, Nils Alexander Haupt, Sebastian Böcker, Sebastian Pomplun

**Affiliations:** a LACDR, Leiden University Leiden 2333 CC The Netherlands s.j.pomplun@lacdr.leidenuniv.nl; b Oncode Institute Utrecht 3521 AL The Netherlands; c Chair for Bioinformatics, Institute for Computer Science, Friedrich Schiller University Jena Jena 07737 Germany

## Abstract

Affinity screenings with encoded libraries are transformative tools for rapid hit discovery from vast compound collections. Yet the adaptation of established chemical reactions to DNA-encoded libraries (DELs) remains challenging due to DNA-compatibility constraints and mismatches between barcode and chemical structure in case of incomplete reactions or side product formation. Recently, we introduced self-encoded libraries (SELs) as a barcode-free alternative to DELs. The SEL platform offers unmatched flexibility in reaction conditions and decodes screening hits directly from their chemical structure, avoiding the problem of mismatched barcode-compound pairs. Here, we expand the SEL platform to Buchwald–Hartwig aminations, enabling the construction of new high diversity SELs. We performed a thorough reaction condition optimization and tested a scope of >170 different building blocks. We adapted our automated MS/MS-based decoding methodology SIRIUS-COMET to the resulting scaffolds, enabling accurate compound decoding from complex mixtures. A 25 725-member library was synthesized and screened all at once against carbonic anhydrase IX (CAIX), resulting in robust enrichment of hits with specific building block patterns and yielding several nanomolar-affinity binders. This work showcases the seamless integration of palladium-catalyzed cross-couplings into SELs, expanding the chemical space of this technology and accelerating hit discovery with high synthetic versatility.

## Abstract 2

We expanded the self-encoded library (SEL) platform *via* C–N cross-couplings, testing 170+ building blocks and generating a 25.725-member library. We established automated MS/MS decoding and demonstrated robust hit enrichment in screens against CAIX.

## Graphical abstract

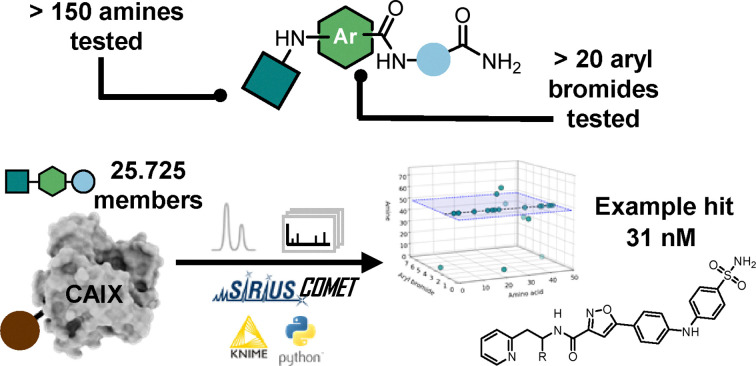

## Introduction

The identification of bioactive molecules is a crucial step in drug discovery, relying heavily on access to large and diverse collections of small molecules. Traditionally, high-throughput screening (HTS) involves the parallel screening of individual compounds but can be resource-intensive. Encoded-library techniques have emerged as a powerful alternative technology, enabling the rapid and cost-effective screening of millions or even billions of compounds simultaneously.^1–3^ Among these, DNA-encoded libraries (DELs) in particular has become a widespread technology in both industry and academia, leading to the discovery of numerous bioactive compounds across a broad range of targets.^1,2,4–6^

Obtaining high-quality combinatorial libraries requires robust, high-yielding chemical reactions to maintain synthetic fidelity. Incorporating a broad range of chemical transformations grants access to a large chemical space and, in turn, increases the probability of discovering novel bioactive molecules. DELs have inherent challenges due to the synthesis of small molecules on DNA strands and although the number of DEL-compatible reactions continues to grow, it remains challenging to find robust conditions for several chemical transformations.^7–9^ Among the palladium-catalyzed cross-coupling reactions described for DELs, Suzuki–Miyaura, Sonogashira and Heck couplings have been successfully implemented under mild, DNA-compatible conditions.^10–13^

The Buchwald–Hartwig amination has become a cornerstone transformation in medicinal chemistry due to the prevalence of arylated amines in pharmaceuticals and natural products.^10,14^ However, its on-DNA implementation is hindered by several DNA degradation conditions, such as high temperatures, palladium-catalyst interactions, and exposure to organic solvents.^12,15–18^ Additionally, the presence of air or water can lead to catalyst inactivation, further complicating its use in DELs. The reported examples of Buchwald–Hartwig on DNA often suffer from low yields and limited substrate scope.^15,19–23^

In principle, the presence of incomplete reaction products is not inherently problematic for affinity selections, as also shown by Cernak *et al.*, who used nanoscale synthesized libraries in affinity ranking experiments.^24^

However, in encoded libraries, such incompleteness leads to a mismatch between the molecular barcode and the corresponding truncated or deleted compound, resulting in misleading outcomes during hit decoding. Although strategies to mitigate these issues are under development, they inevitably introduce additional complexity to library synthesis.^25^

Recently, we introduced self-encoded libraries (SELs) as a barcode-free alternative to DELs.^26^ SELs are prepared by solid-phase synthesis and therefore offer exceptional flexibility in reaction conditions and catalyst compatibility with broad tolerance for organic reagents and elevated temperatures. In the SEL discovery workflow, combinatorial libraries are screened in affinity selections against a protein of interest. Enriched hit compounds are detected using tandem mass spectrometry (MS/MS) and decoded by an automated software (SIRIUS-COMET). SIRIUS-COMET enables the screening of very large libraries (up to 750 000 members) by accurately annotating compound structures from their fragmentation spectra, even in the presence of hundreds of isobaric compounds. Given the absence of DNA-compatibility constraints for library synthesis, the SEL platform enables the implementation of established medicinal chemistry transformations, including those challenging for DELs. Since the hit compounds are decoded directly from their own structure, there are no problems related to barcode-compound mismatches.

In this study, we implement the Buchwald–Hartwig reaction in our SEL platform. Building on previous reports on solid-phase based Buchwald–Hartwig aminations,^27–29^ we performed a thorough reaction condition optimization and a broad substrate scope. We expanded the SIRIUS-COMET decoding workflow to account for the resulting scaffolds and validated the annotation fidelity on ∼600 compounds. With the building blocks validated in the substrate scope, we built a 25 725 membered SEL and benchmarked the whole discovery workflow in affinity selections against carbonic anhydrase IX (CAIX). Robust enrichment of hits with experimentally validated nanomolar affinities were achieved, showcasing the compatibility of the SEL platform with this new library design using Buchwald–Hartwig aminations.

## Results

### Optimization of synthesis conditions for solid-phase Buchwald–Hartwig reactions

We started the condition optimization with the model reaction between solid-phase bound aryl bromide 1 and aniline (Table 1). For determining the conversion efficiency, we cleaved each compound from the solid support and analyzed the resulting mixture by LC-MS.^25^ In a first iteration we kept the Pd source (PdCl_2_), base (K_2_CO_3_) and solvent composition (DMF : H_2_O, 9 : 1) constant and tested a set of different phosphine-based ligands. XPhos and tBuXPhos resulted in moderate conversions of 43% and 40%, respectively, to 2, while the other ligands resulted in poor yields (Table 1, entries 1–6). Changing the base to Na_2_CO_3_, DBU or KOtBu, decreased the reaction conversion (Table 1, entries 7–9). Notably, by switching the palladium source to Pd(OAc)_2_ a substantially better conversion was observed (67%), and could even be improved (80%), using higher catalyst loading (Table 1, entries 10–11). A small solvent screen showed that DMF, dioxane, toluene and anisole gave all comparably good conversions (Table 1, entries 12–14). Based on the conversions and green chemistry guidelines, we prioritized the most environmentally benign option, anisole.^30^ Performing solid-phase reaction under fully inert conditions, especially when aiming at a combinatorial set up, is very challenging. As a feasible set up we performed the reactions in sealed Eppendorf tubes, purged with N_2_. Compared to performing the reactions under air, the reproducibility of the reaction outcomes improved substantially with this set up (data not shown).

**Table 1:** Optimization of the reaction between solid-phase coupled 2-(4-bromophenyl)acetic acid and aniline^a^

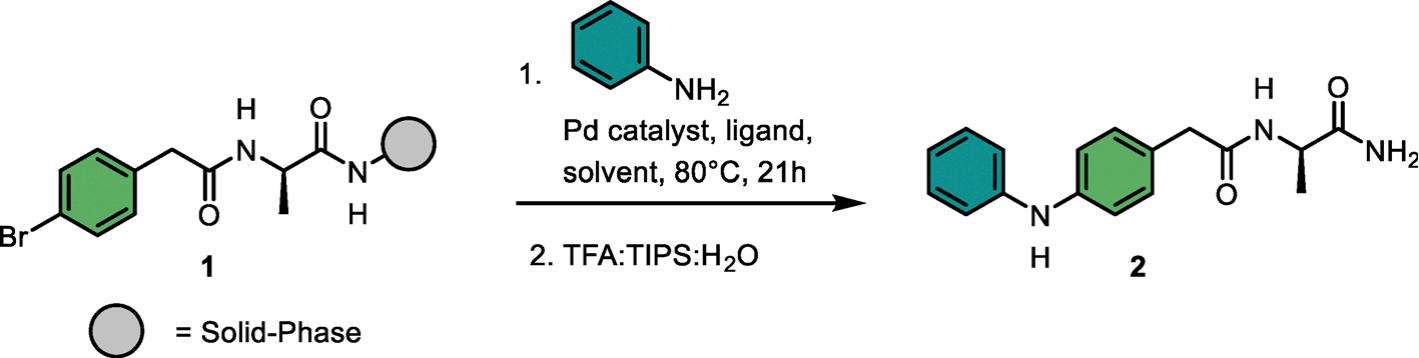					
Entry	Pd catalyst (mol%)	Ligand (mol%)	Base (2 eq.)	Solvent (9 : 1)	Conversion^b^ (%)
1	PdCl_2_ (10)	Xphos (20)	K_2_CO_3_	DMF : H_2_O	43
2	PdCl_2_ (10)	Johnphos (20)	K_2_CO_3_	DMF : H_2_O	11
3	PdCl_2_ (10)	CyJohnphos (20)	K_2_CO_3_	DMF : H_2_O	2
4	PdCl_2_ (10)	Davephos (20)	K_2_CO_3_	DMF : H_2_O	26
5	PdCl_2_ (10)	tBuXphos (20)	K_2_CO_3_	DMF : H_2_O	40
6	PdCl_2_ (10)	Sphos (20)	K_2_CO_3_	DMF : H_2_O	22
7	PdCl_2_ (10)	Xphos (20)	Na_2_CO_3_	DMF :H_2_O	26
8	PdCl_2_ (10)	Xphos (20)	DBU	DMF : H_2_O	0
9	PdCl_2_ (10)	Xphos (20)	KO*t*Bu	DMF : H_2_O	17
10	Pd(OAc)_2_ (10)	Xphos (20)	K_2_CO_3_	DMF : H_2_O	67
**11**	**Pd(OAc)**_**2**_ **(20)**	**Xphos (40)**	**K**_**2**_ **CO**_**3**_	**DMF : H**_**2**_ **O**	**80**
12	Pd(OAc)_2_ (20)	Xphos (40)	K_2_CO_3_	Toluene : H_2_O	78
13	Pd(OAc)_2_ (20)	Xphos (40)	K_2_CO_3_	Dioxane : H_2_O	76
**14**	**Pd(OAc)**_**2**_ **(20)**	**Xphos (40)**	**K**_**2**_ **CO**_**3**_	**Anisole : H**_**2**_ **O**	**83**

a Reaction conditions: 1 (5 µmol, 1 eq.), aniline (10 µmol, 2 eq.), base (10 µmol, 2 eq.), solvent : H_2_O (9 : 1, 100 µL), 80 °C, 21 h.

b Conversion was determined by LC-MS analysis thought peak integration of UV-absorption at 254 nm.

To evaluate the performance of the established conditions with aliphatic amines, we focused on the reaction with 1-methylpiperazine as a model substrate, given that piperidine and piperazine are among the most prevalent nitrogen heterocycles in FDA-approved drugs.^31^ When testing the reaction between model substrate 3 with 1-methylpiperazine under the optimized conditions for aniline, we obtained a conversion to product of only 3% (Table 2, entry 1) which required further improvement of reaction conditions. We first screened 10 different phosphine ligands (Table 2, entries 1–10). Nine of the ten ligands resulted in conversions between 0 and 7% and only tBuXPhos afforded a moderate conversion of 30% (Table 2, entry 10). Increasing the catalyst loading further improved the conversion to 41% (Table 2, entry 11). Another minor improvement to 46% was obtained by using Pd_2_(dba)_3_ (Table 2, entries 12–14). A subsequent solvent screening using different protic and aprotic solvents revealed toluene to be the optimal choice, leading to conversions of 61% (Table 2, entries 15–19). A similar performance was retained at slightly reduced catalyst loadings (Table 2, entry 20). While these results confirm the more challenging nature of Buchwald–Hartwig reactions for combinatorial library synthesis, the optimized conditions gave conversions over 50%, making it suitable for integration in our SEL platform.

**Table 2:** Optimization of the reaction between solid-phase coupled 2-(4-bromophenyl)acetic acid and 1-methylpiperazine^a^

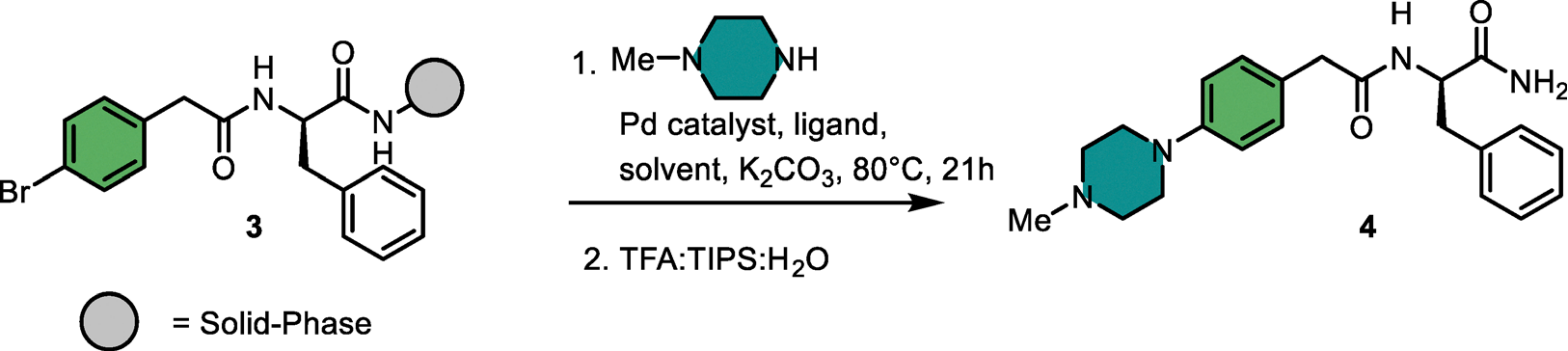				
Entry	Pd catalyst (mol%)	Ligand (mol%)	Solvent (9 : 1)	Conversion^b^ (%)
1	Pd(OAc)_2_ (20)	XPhos (40)	Anisole : H_2_O	3
2	Pd(OAc)_2_ (20)	BINAP (20)	Anisole : H_2_O	0
3	Pd(OAc)_2_ (20)	XantPhos (20)	Anisole : H_2_O	1
4	Pd(OAc)_2_ (20)	SPhos (40)	Anisole : H_2_O	2
5	Pd(OAc)_2_ (20)	RuPhos (40)	Anisole : H_2_O	4
6	Pd(OAc)_2_ (20)	JohnPhos (40)	Anisole : H_2_O	7
7	Pd(OAc)_2_ (20)	CyJohnPhos (40)	Anisole : H_2_O	1
8	Pd(OAc)_2_ (20)	DavePhos (40)	Anisole : H_2_O	2
9	Pd(OAc)_2_ (20)	BrettPhos (40)	Anisole : H_2_O	4
**10**	**Pd(OAc)**_**2**_ **(20)**	**tBuXPhos (40)**	**Anisole : H**_**2**_ **O**	**30**
**11**	**Pd(OAc)**_**2**_ **(50)**	**tBuXPhos (100)**	**Anisole : H**_**2**_ **O**	**41**
12	PdCl_2_ (50)	tBuXPhos (100)	Anisole : H_2_O	18
13	APC (25)	tBuXPhos (100)	Anisole : H_2_O	29
**14**	**Pd**_**2**_ **(dba)**_**3**_ **(25)**	**tBuXPhos (100)**	**Anisole : H**_**2**_ **O**	**46**
15	Pd_2_(dba)_3_ (25)	tBuXPhos (100)	DMF : H_2_O	59
16	Pd_2_(dba)_3_ (25)	tBuXPhos (100)	Dioxane : H_2_O	7
17	Pd_2_(dba)_3_ (25)	tBuXPhos (100)	THF : H_2_O	14
18	Pd_2_(dba)_3_ (25)	tBuXPhos (100)	Dibutylether : H_2_O	51
**19**	**Pd**_**2**_ **(dba)**_**3**_ **(25)**	**tBuXPhos (100)**	**Toluene : H**_**2**_ **O**	**61**
**20**	**Pd**_**2**_ **(dba)**_**3**_ **(20)**	**tBuXPhos (80)**	**Toluene : H**_**2**_ **O**	**60**

a Reaction conditions: 1 (10 µmol, 1 eq.), 1-methylpiperazine (20 µmol, 2 eq.), solvent : H_2_O (9 : 1, 300 µL), K_2_CO_3_ (20 µmol, 2 eq.), 80 °C, 18 h.

b Conversion was determined by LC-MS analysis through peak integration of UV-absorption at 254 nm.

### Building block scope

**Fig. 1:**
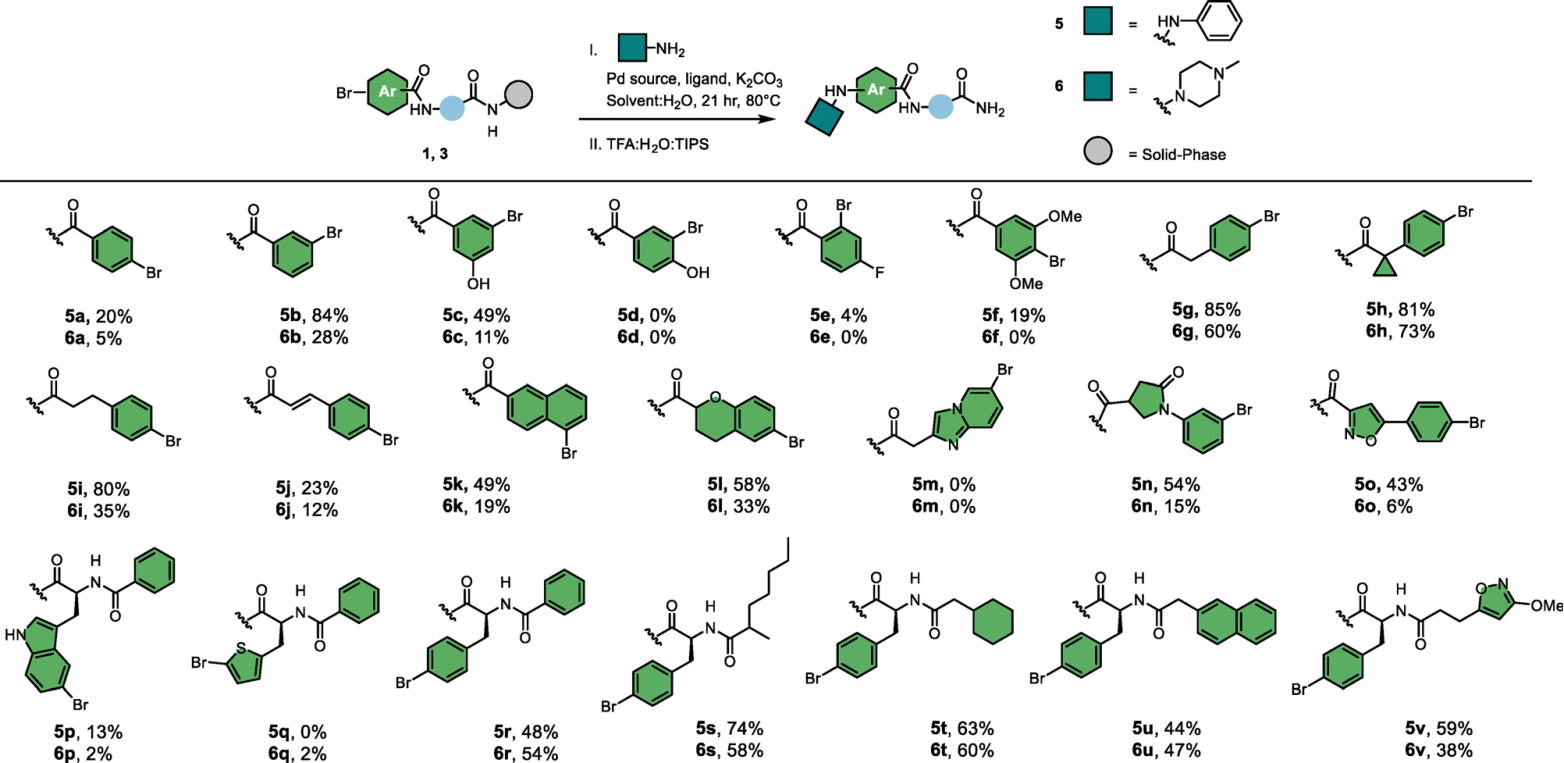
Scope of aryl bromides. ^*a*^ Compounds 5 were reacted with starting material 1 and compounds 6 with starting material 3. ^*b*^ Conversion was determined by LC-MS analysis thought peak integration of UV-absorption at 254 nm.

With the optimized conditions for aromatic and aliphatic amines, we evaluated the reaction scope on 22 different solid-phase bound aryl bromides, reacting each with aniline and 1-methylpiperazine. Looking ahead to the planned combinatorial library synthesis, we defined a threshold of 50% conversion to consider a building block suitable for library incorporation. From the 22 aryl bromides investigated (Fig. 1), nine resulted in conversions >50% when reacted with aniline and five when reacted with 1-methylpiperazine. The majority of substituted benzoic acids (5a–f, 6a–f) gave unsatisfactory results. Bromophenylacetic acid variants (5g, 6g) and bromophenylpropanoic acids (5i–j, 6i–j) were well-tolerated. The bicyclic structures (5k–n, 6k–n) showed a general preference for the coupling with aromatic amines compared to the reaction with aliphatic amines. Furthermore, bromophenylalanine derivatives bearing different carboxylic acid decorators (5r–v, 6r–v) generally resulted in reasonable conversions.

**Fig. 2:**
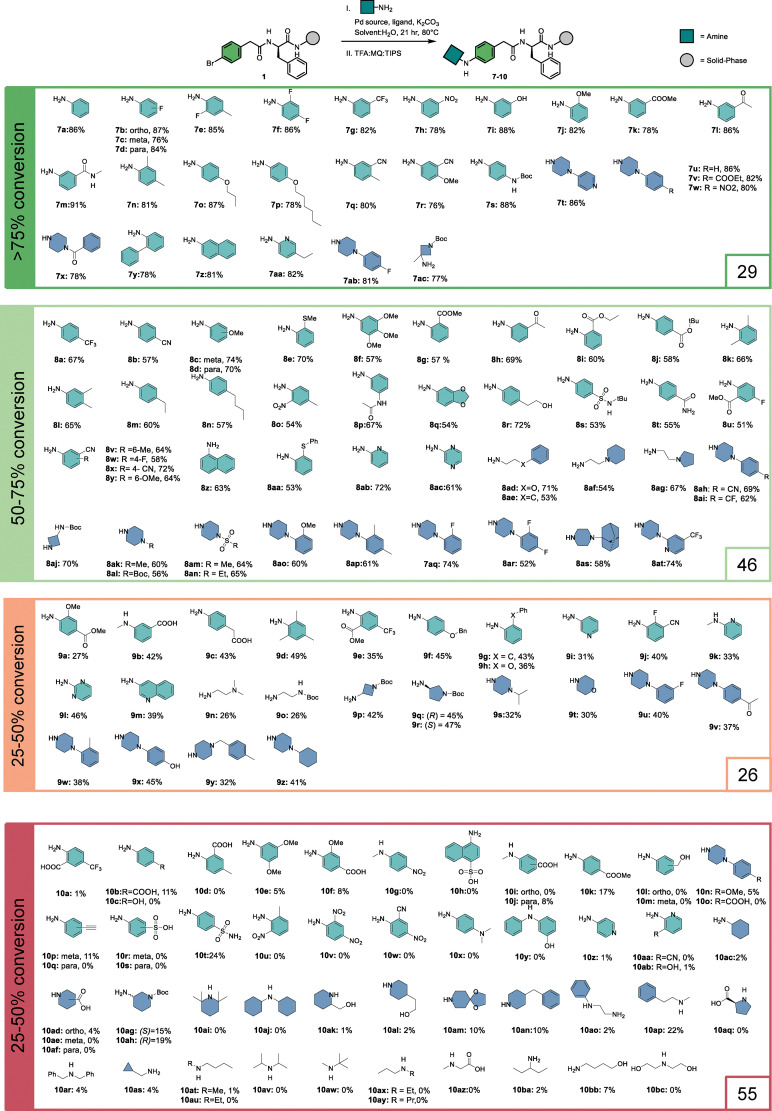
Scope of 156 amines. ^a^ The investigated amines are categorized based on conversion. ^b^ The turquoise colored amine used the reaction conditions from Table 1, entry 14. The dark blue amine used the reaction conditions from Table 2, entry 20. ^c^ Conversion was determined by LC-MS analysis through peak integration of UV-absorption at 254 nm.

Next, we tested the reaction between substrate 1 and 156 aromatic and aliphatic amine building blocks (Fig. 2). A broad range of aromatic amines bearing alkyl, alkoxy, fluoro, trifluoromethyl, nitrile, ester, and acetyl substituents were converted to the desired products with conversions >50%. In contrast, amines containing alkyne, carboxylic acid, sulfonic acid, free sulfonamide, and nitro substituents mostly resulted in low conversions. From the heterocyclic amines, 2-aminopyridine and 2-aminopyrazine underwent efficient coupling, whereas the 3-amino- and 4-aminopyridine isomers or substituted derivatives gave poor conversion, likely due to their reduced nucleophilicity. Several piperazine analogs and a few other cyclic- and primary aliphatic amines met the conversion threshold. Among the variants that displayed lower conversions, steric hindrance seemed to be the most prominent common feature. Overall, 75 building blocks showed conversion over 50%, providing a set of diverse building blocks for library synthesis.

### Library decoding

The SEL workflow entails screening a large library all at once against a target of interest *via* affinity selection. As opposed to classical AS-MS,^32–35^ no particular attention to the presence of isobaric compounds in a library is needed. This is possible thanks to our previously developed computational tool, SIRIUS-COMET, capable of annotating compounds from MS/MS data and annotate their structures.^26^

**Fig. 3:**
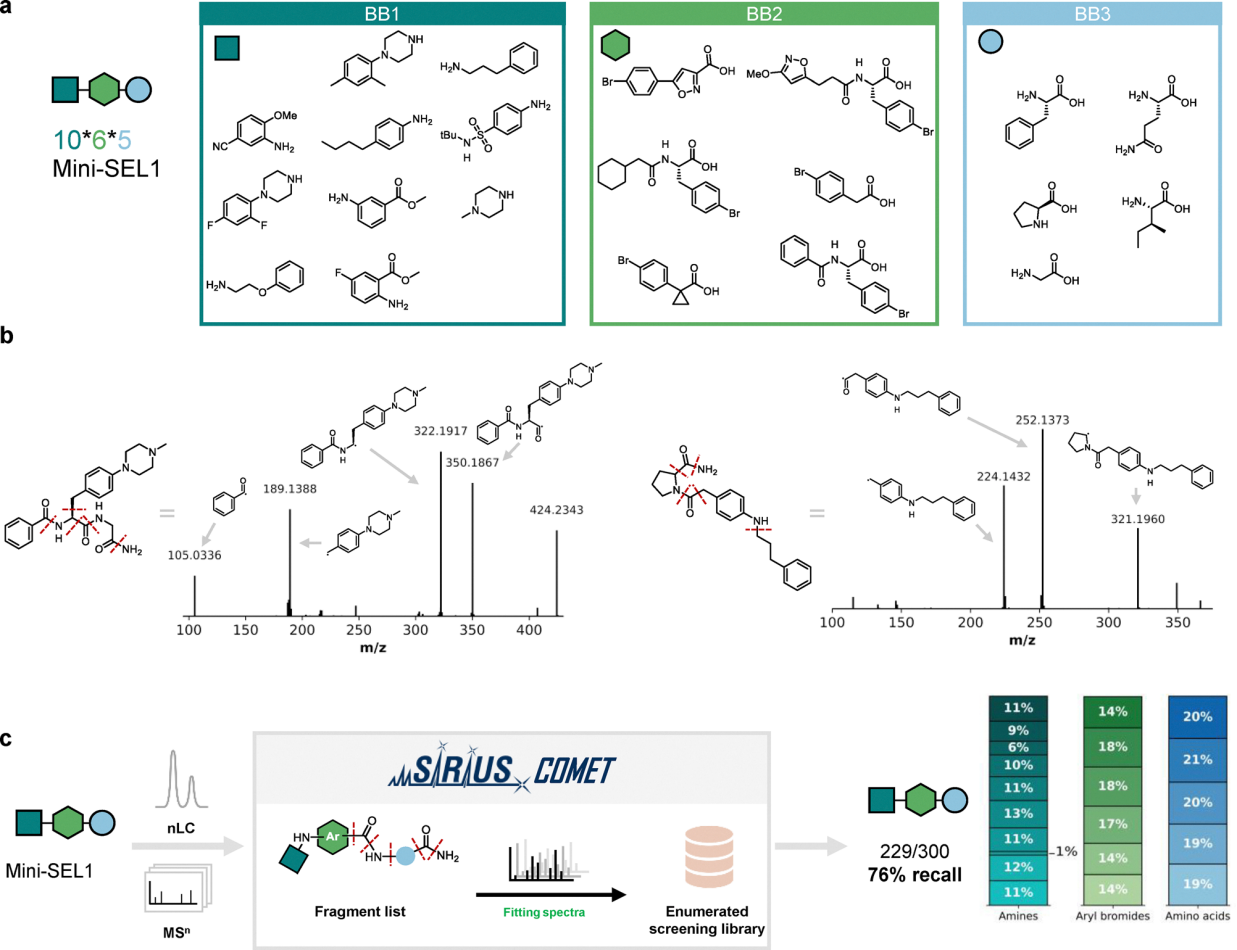
Library decoding using SIRIUS-COMET (a) MiniSEL1 was synthesized *via* split-and-pool synthesis on solid support using selected amino acid, aryl bromide and amine building blocks. (b) Examples of LC-MS/MS traces. The highest intensity fragments were used as input in SIRIUS-COMET. A generalized overview of the fragments is depicted in Fig. S7. (c) The LC-MS/MS data was imported into SIRIUS-COMET. MS/MS spectra were filtered based on the minimum number of matching peaks from the fragment list. Compounds are then annotated based on a database containing all library members. The workflow results in good recall rates considering when considering the top ranked annotated structures by EPIMETHEUS and building blocks are evenly distributed among the annotated structures.

In order to evaluate the decoding software and optimize parameters for this new Buchwald–Hartwig based library, we first synthesized two defined test libraries of 300 compounds each (miniSEL1 and miniSEL2, Fig. 3a and Fig. S1). Each library consists of a scaffold with three variable positions: an amino acid building blocks (BB3) coupled to an aryl bromides (BB2) and subsequently reacted with amines (BB1) using the established conditions. Each library was prepared *via* split-and-pool synthesis, bulk purified and then measured as a mixture by nanoLC–MS/MS. First, we analyzed a few selected spectra manually to observe overall trends in fragmentation of the library compounds. The most prominent and consistent fragmentation observed across scaffolds is the amide bond between the amino acid building block and the aryl bromide (see Fig. 3b for selected examples). Also, the terminal amide resulting from solid-phase synthesis was fragmented in all selected compounds. The C–N bond formed by the Buchwald–Hartwig coupling fragmented only sporadically with a low signal intensity. Increasing the fragmentation energy improved fragment intensity, but not enough to utilize it as a consistent fragment for library decoding (Fig. S2).

For the full decoding of our libraries, we used SIRIUS-COMET,^26^ a software platform that enables accurate annotation and decoding of combinatorial libraries and at the same time effectively reducing the number of false positive annotations. A fragment list based on the selected most prominent scaffold fragmentations is used to filter MS/MS scans based on a minimum number of matching peaks. The remaining spectra were then annotated using EPIMETHEUS, the ranking and substructure annotation module within the SIRIUS software, using the enumerated library database (represented as SMILES).

Analysis of the MS/MS spectra of miniSEL1 and 2 with SIRIUS-COMET resulted in the correct annotation of 229/300 library members for miniSEL1 (76%), and 218/300 (73%) for miniSEL2. An even distribution among the building blocks was observed, except for 4-butylaniline, indicating that combinations of the optimized building blocks were generally well tolerated (Fig. 3c and Fig. S3). These results confirm the successful synthesis under combinatorial settings of most library members, forming a robust basis for the preparation of larger libraries.

As SELs are decoded by their chemical structure, we investigated whether common side products could be detected and characterized. In this way, side products could even become part of the enumerated library and contribute to greater diversity. While analyzing the building block scope, we often observed conversions to the following recurring side products; unreacted starting material 1 and its hydrolyzed and proto-dehalogenated derivative. While we had previously confirmed the correct synthesis of the majority of all library compounds for miniSEL1 and 2, we wanted to assess whether we could also detect and annotate these combinatorial side products. For miniSEL1 74 out of 90 possible side products were identified in the MS/MS spectra (Fig. S5) and 16/45 for miniSEL2 (Fig. S6). These results highlight that SELs can, in principle, identify any structure formed during synthesis, provided that the underlying reaction pathways are known. Whether these side products will actually be productive and detectable in real screening campaigns will dependent on each individual quantity, but, notably, many of these structures would be incorrectly assigned in conventional DEL screenings and would require extensive off-DNA validation to determine the correct hit structure.

### Library synthesis and selection against CAIX

**Fig. 4:**
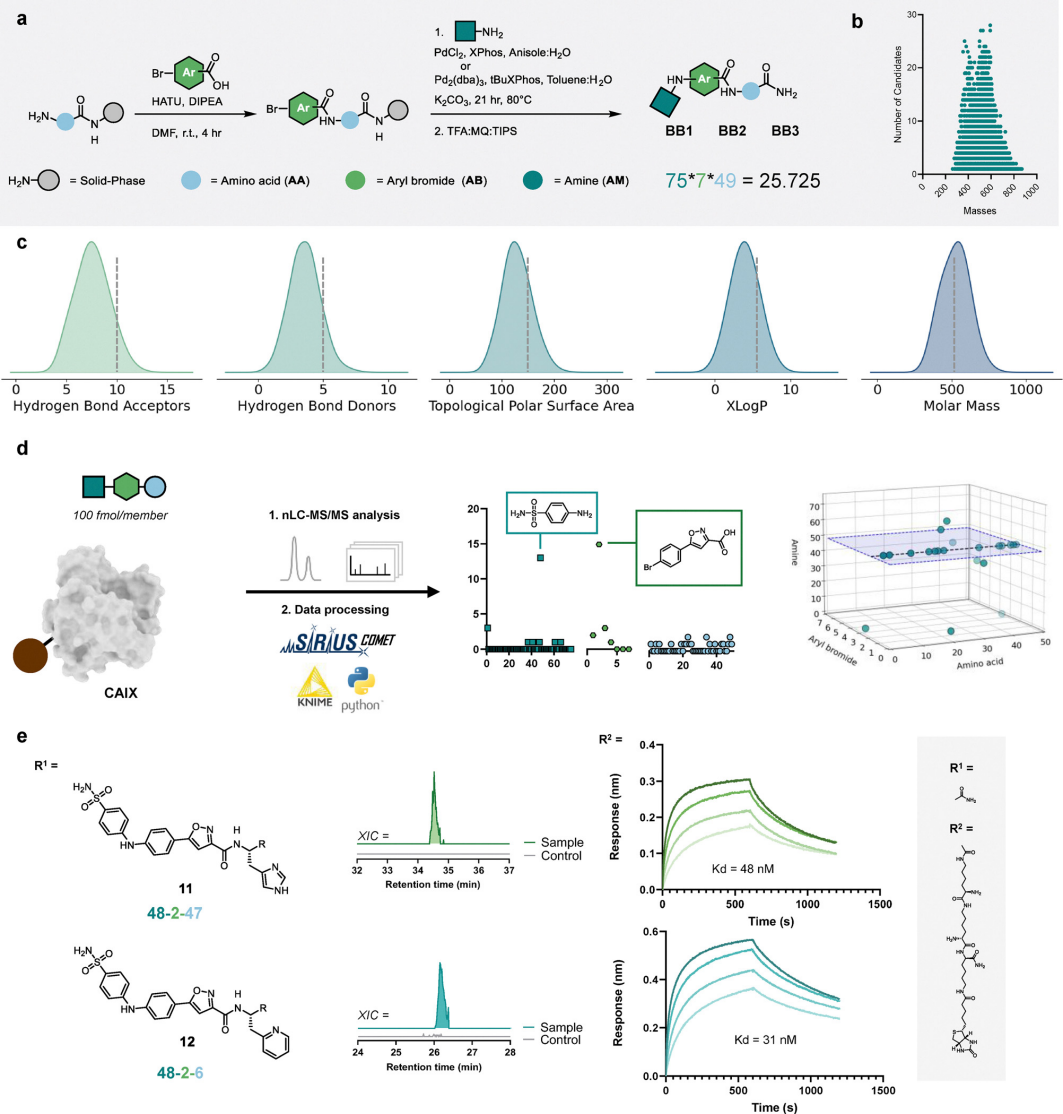
Library synthesis and the identification of ligands against CAIX (a) The SEL design consists of an amino acid and the optimized Buchwald–Hartwig cross coupling between an aryl bromide and amine. Building blocks with crude purity over 50% were selected. (b) The scatter plot shows the mass degeneracy at 5 ppm mass accuracy. (c) The distribution plot of druglike properties show that the average distribution abides Lipinski's rule of 5. (d) AS-MS results plotted based on the building block frequencies from the best ranked annotations plotted as 2D and 3D scatter plots. The *x*, *y* and *z* axis represent the three building blocks. Showing that the combination of 5-(4-Bromophenyl) isoxazole-3-carboxylic acid and 4-aminobenzenesulfonamide is significantly enriched (*p* < 0.0001, see Fig. S4), indicated by the black dotted line. (e) Structure of two selected hits. The extracted ion chromatogram using 5 ppm accuracy shows selective enrichment for CAIX compared to the streptavidin magnetic beads control. Binding affinity was determined by BLI association/dissociation curves of the biotinylated version of hit compounds.

With an established method for combinatorial synthesis and decoding of Buchwald–Hartwig based libraries, the preparation of a large SEL library was initiated. Using 49 amino acid building blocks, 7 aryl bromides and 75 amines, we assembled a SEL with 25 725 members (Fig. 4a). Depending on the type of amine, we used the respective optimized conditions for the Buchwald–Hartwig amination step. For specific exact masses the library contains up to 28 isobaric compounds but each of those features a distinct combination of building blocks, likely enabling structure annotation through MS/MS fragmentation (Fig. 4b). We computationally enumerated the entire library and visualized compliance with the Lipinski parameters: molecular weight (MW < 500 Da), *X* log *P* (> 5), hydrogen bond donors (HBD < 5), hydrogen bond acceptors (HBA < 10), and topological polar surface area (TPSA < 140) (Fig. 4c).

Finally, we wanted to validate the entire discovery workflow including affinity selection, tandem mass spectrometry and hit decoding. To this end, we performed an affinity selection experiment against CAIX. CAIX is a therapeutically relevant target for cancer treatment, particularly in hypoxic tumors, due to its role in tumor cell survival and pH regulation in the tumor microenvironment. High affinity CAIX ligands are enabling tools for radionuclide cancer therapy.^36^ Moreover, CAIX is a preferred scaffold for benchmarking library selections due to its predictable binding profile to aromatic sulfonamides.^25,36^ In addition, other members of the carbonic anhydrase family have frequently served as benchmark targets for the validation of ligand discovery strategies such as *in situ* click chemistry^37^ and direct to biology screening.^38,39^

The affinity selection was performed on biotin-CAIX, immobilized on streptavidin-coated magnetic beads and incubated with the library (using 100 fmol per member). After washing away non-binders, protein-bound hits were eluted under denaturing conditions and analyzed with our nanoLC-MS/MS SIRIUS-COMET workflow. The selection in parallel was performed on unfunctionalized magnetic streptavidin beads. Non-specific binders, appearing in both selections, were computationally removed from our analysis by performing a background subtraction. Only compounds displaying at least three-fold higher intensity in the CAIX sample were considered as hits.

After background subtraction and SIRIUS-COMET structure annotation, we obtained 21 candidate structures. When analyzing building block frequencies, we found a significant enrichment of 4-aminobenzenesulfonamide, appearing in 13 annotated structures as first ranked (Fig. 4d). Given the known affinity of 4-aminobenzenesulfonamide for CAIX, these 13 hits can be considered as real binders with high confidence. For four of the remaining eight candidates, the sulfonamide building block was still present, but proposed with a lower rank. This outcome indicates that optimizing the ranking algorithm could improve hit detection in the future. The remaining four candidates in the manual analysis showed unclear enrichment and/or overall poor structure annotation and were not considered as hits. Interestingly, the sulfonamide always occurred in combination with 5-(4-bromophenyl)isoxazole-3-carboxylic acid, indicating that this combination is important for binding (Fig. 4d). The building block enrichment (proportion of sulfonamide in hits *vs.* expected random distribution) has a strong statistical significance with a *p* value of *p* < 0.0001 (Fig. S5).

From the hits we selected two compounds for experimental binding validation (Fig. 4e). We prepared variants of both compounds (11 and 12) with a biotin tag and tested their binding *via* biolayer interferometry (BLI), which demonstrated low nanomolar binding affinities with *K*_D_ values of 48 nM and 31 nM, respectively. We resynthesized the untagged version of compound 12 to further confirm and validate the results of our affinity selection. While the nanoLC retention time and MS/MS spectra matched the original hit perfectly (Fig. S18 and S19), NMR characterization was hindered by a proto-dehalogenated side product formed during synthesis. Despite multiple attempts, this byproduct could not be separated from the target compound (Fig. S20 and S21). This difficulty illustrates a common bottleneck in combinatorial chemistry, where the transition from a mixture-based “hit” to a purified, isolated compound is not always straightforward. Altogether, the CAIX selection demonstrates that Buchwald–Hartwig aminations can be incorporated in SELs and enable discovery campaigns from large libraries.

## Discussion and conclusion

Here, we developed procedures for solid-phase Buchwald–Hartwig aminations, enabling the construction of large combinatorial libraries. We optimized reaction conditions for aromatic and aliphatic amines and tested a building block scope of 22 aryl bromides and 156 amines. Building blocks with incorporations >50% were included in a final SEL of 25 725 members. We adapted our previously developed SIRIUS-COMET software to enable the automated structure annotation of MS/MS spectra of complex samples of compounds resulting from the combinatorial Buchwald–Hartwig amination. Finally, we demonstrated the application in AS-MS/MS against CAIX, resulting in robust hit enrichment and two validated binders with nanomolar affinities (48 nM and 31 nM), highlighting the potential of SELs for discovering novel hits.

Compared to Suzuki cross couplings that we previously developed,^26^ establishing the Buchwald–Hartwig amination for combinatorial synthesis was more challenging, exhibited a narrower substrate scope and lower yields. The Buchwald–Hartwig amination reported here required tailored reaction conditions based on the type of amine, resulting in distinct reaction protocols for primary aromatic and cyclic aliphatic amines. Primary aromatic amines were generally well tolerated, while aliphatic amines showed poor conversion and probably due to their susceptibility to β-hydride elimination. Cyclic amines outperformed their acyclic counterparts. Both findings are consistent with previous reaction performed in DEL optimization.^15,40^

A remaining challenge for DELs is the identification of true hits after the selection arising from incorrectly encoded library members, due to truncations or side product formation. In contrast, SELs enable direct decoding of compounds from their own structure, thereby eliminating issues related to barcode-compound mismatches. Moreover, SELs also offer the potential to detect and characterize side products that may arise during the synthesis.

Future research will be needed to further expand the chemical space by incorporating a more diverse set of aryl bromides and amines. A significant variability in ligand performance in our optimizations would suggest that further optimization into newly developed ligands for the incorporation of primary amines,^41^ secondary amines^42,43^ and heterocycles^44–46^ would increase our substrate scope. Since we have no DNA constraints, we can likely directly apply novel reported reaction conditions to solid-support, offering a distinct advantage over DEL-based reaction optimization.

To decode isobaric compounds in larger libraries (beyond 100 000 members), additional MS/MS fragment information on the introduced C–N is required. Under the current conditions, this bond fragments only sporadically, limiting confident structural assignment for larger libraries, as information about the amine building block is missing. By further optimizing MS/MS-based fragmentation in terms of collision energy and structure annotations, we could potentially enhance the decoding accuracy.

The modest library size shown in this study still exceeds size the size of pools (typically 500–1000 members) that can be screened by classical affinity selection mass spectrometry screens, relying on identification by MS1. DELs usually achieve substantially larger library sizes. However, we are confident that our SEL platform continues expanding and multiple libraries with different scaffolds will overall contribute to a broad chemical diversity.

The selection against CAIX yielded 13 hits, where the enrichment was dependent on the combination of 4-aminobenzenesulfonamide and 5-(4-bromophenyl)isoxazole-3-carboxylic acid. CAIX is known to bind to aromatic sulfonamides, but adjacent BBs can play an important role.^36^ The dependence on these two building blocks showcase the importance of developing diverse chemistry of different library scaffolds.

From the 343 potential sulfonamide binders, only a fraction was identified. This outcome reflects the constraints arising from building-block orientation in combination with the applied washing protocol. While our washing steps efficiently remove non-binders also ligands with fast dissociation rates might be lost. Consequently, at 100 fmol per library member, the assay is biased toward the identification of low-nanomolar binders. Fine-tuning the library concentration and washing parameters could allow systematic modulation of this affinity threshold, an aspect to be explored in subsequent studies.

Metal residues in the library might pose a challenge in specific screening campaigns. The solid support based synthesis enables extensive washing after the cross-coupling step, likely removing the catalyst from the system. After acidic cleavage from the resin the library is also bulk purified on a C18 column, leading to further removal of undesired components. In this study we did not take additional steps for quantitative metal removal. We acknowledge that in specific cases the presence of palladium might represent a limitation and we suggest in these cases to perform additional resin washes using potent metal chelators.

Overall, this work expands the chemical space accessible to self-encoded libraries and illustrates how established reactions can be integrated into the SEL platform for early drug discovery workflows.

## Author contributions

Conceptualization: EvdN, ZL, QQG, SP, synthesis: EvdN, ZL, QQG, methodology: EvdN, NAH, SB, SP, assays: EvdN, software: NAH, SB, visualization: EvdN, SP, supervision: EvdN, SP, funding acquisition: SP, SB, writing – original draft: EvdN, SP, writing – review & editing: EvdN, NAH, SB, SP.

## Conflicts of interest

SP, SB, EvdN and NAH have filed a patent application for the methodology described here. The remaining authors declare no competing interests.

## Supplementary Material

CB-007-D5CB00303B-s001

## Data Availability

The raw LC-MS/MS files and the files needed to run SIRIUS:COMET have been deposited in the Zenodo database (https://doi.org/10.5281/zenodo.17632652). Source data are provided with this paper. All other data is available in the main text, the supplementary information (SI) and from the corresponding author(s) upon request. Supplementary information is available. See DOI: https://doi.org/10.1039/d5cb00303b.
