# Supplementary material for: Integration of palladium-catalyzed C–N coupling into self-encoded libraries for accelerated hit discovery
Source: RSC Chem Biol. 2026 Mar 3;7(4):630–40. doi: 10.1039/d5cb00303b (PMC12978271; doi:10.1039/d5cb00303b)
Supplement: CB-007-D5CB00303B-s001 [file CB-007-D5CB00303B-s001.pdf]

Supplementary Information (SI) for RSC Chemical Biology.
This journal is © The Royal Society of Chemistry 2026

Supplementary information for

Integration of Palladium-Catalyzed C–N Coupling into Self-Encoded Libraries for Accelerated Hit Discovery

Authors: Edith van der Nol[1,2]+, Zhenshuo Luo[1]+, Qing Qing Gao[1]+, Nils Alexander Haupt[3], Sebastian Böcker[3], Sebastian Pomplun[1,2]*

Affiliations:

[1]LACDR, Leiden University; Leiden, 2333 CC, The Netherlands

[2]Oncode Institute; Utrecht, 3521 AL, The Netherlands

[3]Chair for Bioinformatics, Institute for Computer Science, Friedrich Schiller University Jena, Jena, 07737, Germany

*Corresponding authors. Email: s.j.pomplun@lacdr.leidenuniv.nl

+These authors contributed equally to this work

Table of Contents

1. Supplementary figures: 3
2. Abbreviations 10
3. Material and Methods 11
   - 3.1 Reagents and supplies 11
   - 3.2 Instrumentation 11
   - 3.3 General procedures 12
   - 3.4 Software 13
4. Library enumeration 14
5. SIRIUS:COMET 15
6. Library synthesis 17
   - 6.1 MiniSEL1 17
   - 6.2 MiniSEL2 17
   - 6.3 SEL 18
7. Affinity selection against CAIX 19
   - 7.1 List of hit structures 19
   - 7.2 Enrichment plots 20
   - 7.3 Hit identification 20
   - 7.4 Hit resynthesis 23
   - 7.5 Hit validation 25
8. List of building blocks 26
9. Supplementary LC-MS data 30
   - 9.1 LC-MS data from the alkyl bromide scope 30
   - 9.2 LC-MS data from the aryl amine scope 30
   - 9.3 LC-MS data from the alkyl amine scope 30

1. Supplementary figures:

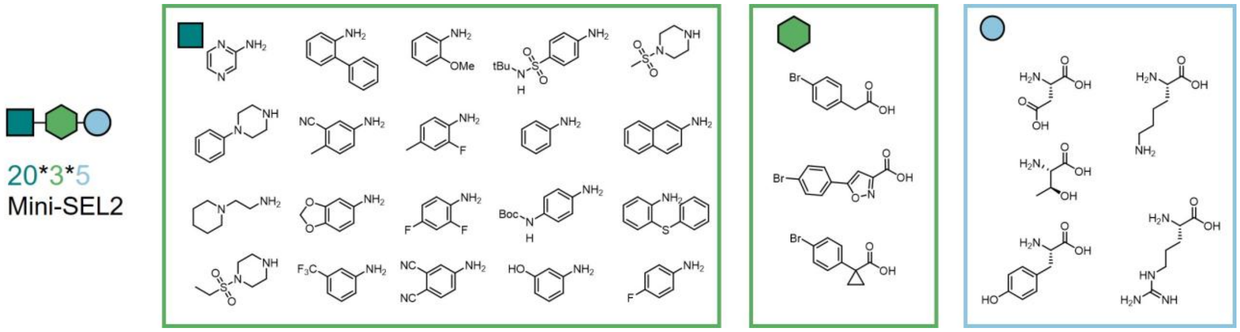

Fig. S1. Building blocks used for Mini-SEL 2

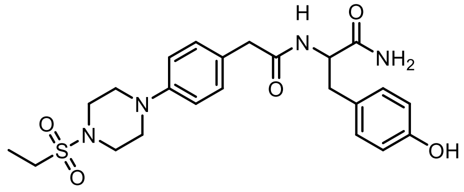

15, 25 eV:

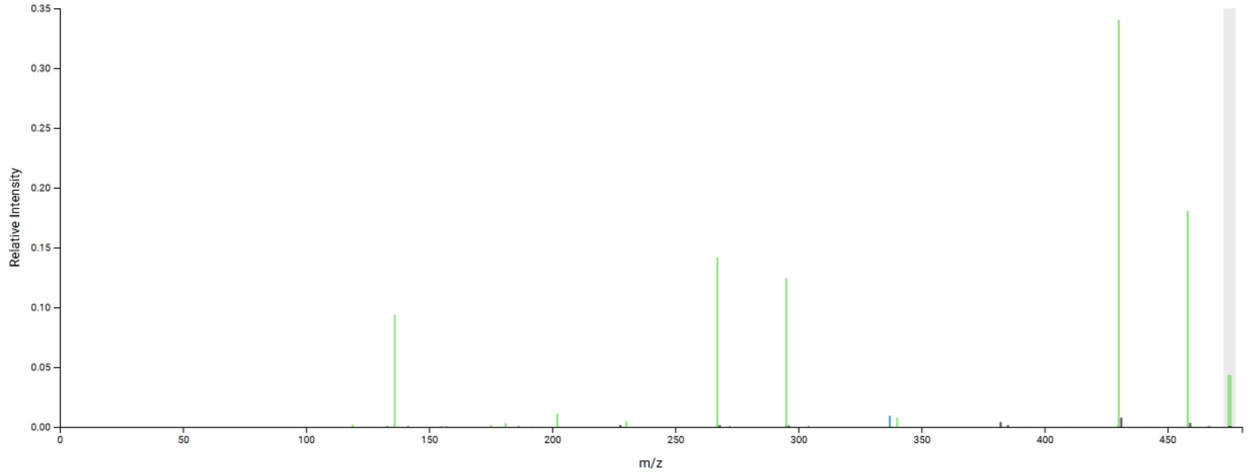

15, 25, 60 eV

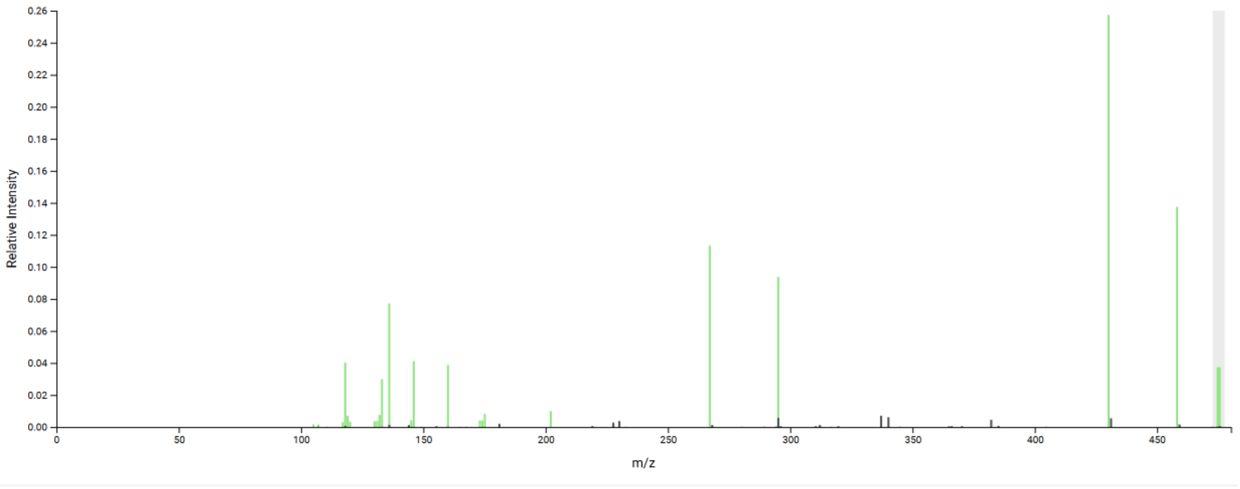

Fig. S2. Examples of fragmentation spectra using different CEs

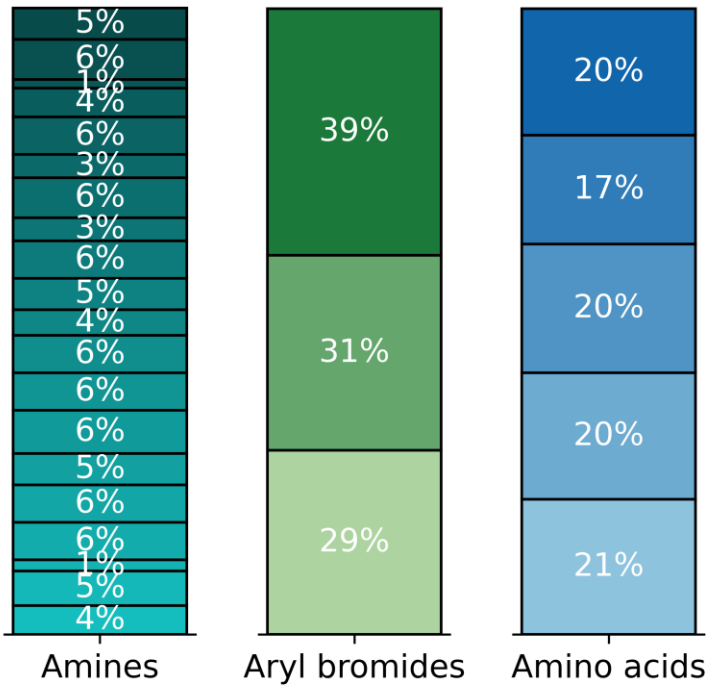

Fig. S3. Building blocks distribution of miniSEL2

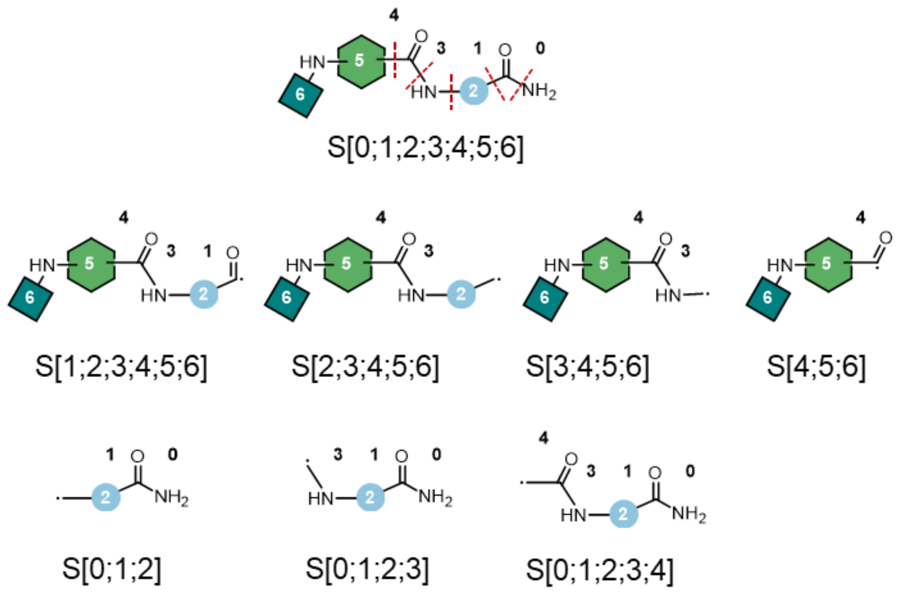

Fig. S4. Visualization of the fragment types used in COMET

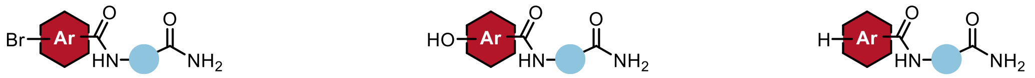

24/30 24/30 26/30

	Truncated	Hydrolyzed	Dehalogenated
**1**	C1CC1(C2=CC=C(C=C2)Br)C(=O)NCC(=O)N	NC(=O)CNC(=O)c1cc(-c2ccc(O)cc2)on1	C1CC(N(C1)C(=O)C2(CC2)C3=CC=CC=C3)C(=O)N
**2**	NC(=O)CNC(=O)C(Cc1ccc(Br)cc1)NC(=O)c1ccccc1	C1=CC=C(C=C1)C(=O)NC(CC2=CC=C(C=C2)O)C(=O)NCC(=O)N	NC(=O)CCC(NC(=O)Cc1ccccc1)C(N)=O
**3**	NC(=O)CNC(=O)C(Cc1ccc(Br)cc1)NC(=O)CC1CCCCC1	NC(=O)CNC(=O)C(Cc1ccc(O)cc1)NC(=O)CC1CCCCC1	CCC(C)C(NC(=O)C1(c2ccccc2)CC1)C(N)=O
**4**	COc1cc(CCC(=O)NC(Cc2ccc(Br)cc2)C(=O)NCC(N)=O)on1	COc1cc(CCC(=O)NC(Cc2ccc(O)cc2)C(=O)NCC(N)=O)on1	C1=CC=C(C=C1)CC(C(=O)N)NC(=O)CC2=CC=CC=C2
**5**	NC(=O)C(Cc1ccccc1)NC(=O)Cc1ccc(Br)cc1	NC(=O)C(Cc1ccccc1)NC(=O)Cc1ccc(O)cc1	NC(=O)C1CCCN1C(=O)c1cc(-c2ccccc2)on1
**6**	NC(=O)C(Cc1ccccc1)NC(=O)C1(c2ccc(Br)cc2)CC1	NC(=O)C(Cc1ccccc1)NC(=O)c1cc(-c2ccc(O)cc2)on1	NC(=O)CCC(NC(=O)C1(c2ccccc2)CC1)C(N)=O
**7**	NC(=O)C(Cc1ccccc1)NC(=O)C(Cc1ccc(Br)cc1)NC(=O)c1ccccc1	NC(=O)C(Cc1ccccc1)NC(=O)C1(c2ccc(O)cc2)CC1	CCC(C)C(NC(=O)c1cc(-c2ccccc2)on1)C(N)=O
**8**	NC(=O)C(Cc1ccccc1)NC(=O)C(Cc1ccc(Br)cc1)NC(=O)CC1CCCCC1	C1=CC=C(C=C1)CC(C(=O)N)NC(=O)C(CC2=CC=C(C=C2)O)NC(=O)C3=CC=CC=C3	NC(=O)C(Cc1ccccc1)NC(=O)C1(c2ccccc2)CC1
**9**	COc1cc(CCC(=O)NC(Cc2ccc(Br)cc2)C(=O)NC(Cc2ccccc2)C(N)=O)on1	NC(=O)C(Cc1ccccc1)NC(=O)C(Cc1ccc(O)cc1)NC(=O)CC1CCCCC1	NC(=O)CCC(NC(=O)c1cc(-c2ccccc2)on1)C(N)=O
**10**	NC(=O)CCC(NC(=O)Cc1ccc(Br)cc1)C(N)=O	COc1cc(CCC(=O)NC(Cc2ccc(O)cc2)C(=O)NC(Cc2ccccc2)C(N)=O)on1	C1=CC=C(C=C1)CC(C(=O)NCC(=O)N)NC(=O)C2=CC=CC=C2
**11**	NC(=O)CCC(NC(=O)C1(c2ccc(Br)cc2)CC1)C(N)=O	NC(=O)CCC(NC(=O)c1cc(-c2ccc(O)cc2)on1)C(N)=O	C1=CC=C(C=C1)CC(C(=O)N)NC(=O)C2=NOC(=C2)C3=CC=CC=C3
**12**	NC(=O)CCC(NC(=O)C(Cc1ccc(Br)cc1)NC(=O)c1ccccc1)C(N)=O	NC(=O)CCC(NC(=O)C1(c2ccc(O)cc2)CC1)C(N)=O	NC(=O)CNC(=O)C(Cc1ccccc1)NC(=O)CC1CCCCC1

**13**	NC(=O)CCC(NC(=O)C(Cc1ccc(Br)cc1)NC(=O)CC1CCCCC1)C(N)=O	NC(=O)CCC(NC(=O)C(Cc1ccc(O)cc1)NC(=O)c1ccccc1)C(N)=O	NC(=O)C1CCCN1C(=O)C(Cc1ccccc1)NC(=O)c1ccccc1
**14**	COc1cc(CCC(=O)NC(Cc2ccc(Br)cc2)C(=O)NC(CCC(N)=O)C(N)=O)on1	NC(=O)CCC(NC(=O)C(Cc1ccc(O)cc1)NC(=O)CC1CCCCC1)C(N)=O	COc1cc(CCC(=O)NC(Cc2ccccc2)C(=O)NCC(N)=O)on1
**15**	CCC(C)C(NC(=O)C1(c2ccc(Br)cc2)CC1)C(N)=O	COc1cc(CCC(=O)NC(Cc2ccc(O)cc2)C(=O)NC(CCC(N)=O)C(N)=O)on1	CCC(C)C(NC(=O)C(Cc1ccccc1)NC(=O)c1ccccc1)C(N)=O
**16**	CCC(C)C(NC(=O)C(Cc1ccc(Br)cc1)NC(=O)c1ccccc1)C(N)=O	CCC(C)C(NC(=O)C1(c2ccc(O)cc2)CC1)C(N)=O	NC(=O)C1CCCN1C(=O)C(Cc1ccccc1)NC(=O)CC1CCCCC1
**17**	CCC(C)C(NC(=O)C(Cc1ccc(Br)cc1)NC(=O)CC1CCCCC1)C(N)=O	CCC(C)C(NC(=O)C(Cc1ccc(O)cc1)NC(=O)c1ccccc1)C(N)=O	C1=CC=C(C=C1)CC(C(=O)NC(CCC(=O)N)C(=O)N)NC(=O)C2=CC=CC=C2
**18**	CCC(C)C(NC(=O)C(Cc1ccc(Br)cc1)NC(=O)CCc1cc(OC)no1)C(N)=O	CCC(C)C(NC(=O)C(Cc1ccc(O)cc1)NC(=O)CC1CCCCC1)C(N)=O	CCC(C)C(NC(=O)C(Cc1ccccc1)NC(=O)CC1CCCCC1)C(N)=O
**19**	NC(=O)C1CCCN1C(=O)Cc1ccc(Br)cc1	CCC(C)C(NC(=O)C(Cc1ccc(O)cc1)NC(=O)CCc1cc(OC)no1)C(N)=O	COc1cc(CCC(=O)NC(Cc2ccccc2)C(=O)N2CCCC2C(N)=O)on1
**20**	NC(=O)C1CCCN1C(=O)c1cc(-c2ccc(Br)cc2)on1	NC(=O)C1CCCN1C(=O)c1cc(-c2ccc(O)cc2)on1	C1=CC=C(C=C1)CC(C(=O)N)NC(=O)C(CC2=CC=CC=C2)NC(=O)C3=CC=CC=C3
**21**	NC(=O)C1CCCN1C(=O)C1(c2ccc(Br)cc2)CC1	NC(=O)C1CCCN1C(=O)C1(c2ccc(O)cc2)CC1	NC(=O)CCC(NC(=O)C(Cc1ccccc1)NC(=O)CC1CCCCC1)C(N)=O
**22**	NC(=O)C1CCCN1C(=O)C(Cc1ccc(Br)cc1)NC(=O)c1ccccc1	NC(=O)C1CCCN1C(=O)C(Cc1ccc(O)cc1)NC(=O)c1ccccc1	CCC(C)C(NC(=O)C(Cc1ccccc1)NC(=O)CCc1cc(OC)no1)C(N)=O
**23**	NC(=O)C1CCCN1C(=O)C(Cc1ccc(Br)cc1)NC(=O)CC1CCCCC1	NC(=O)C1CCCN1C(=O)C(Cc1ccc(O)cc1)NC(=O)CC1CCCCC1	NC(=O)C(Cc1ccccc1)NC(=O)C(Cc1ccccc1)NC(=O)CC1CCCCC1
**24**	COc1cc(CCC(=O)NC(Cc2ccc(Br)cc2)C(=O)N2CCCC2C(N)=O)on1	COc1cc(CCC(=O)NC(Cc2ccc(O)cc2)C(=O)N2CCCC2C(N)=O)on1	COc1cc(CCC(=O)NC(Cc2ccccc2)C(=O)NC(CCC(N)=O)C(N)=O)on1
**25**			NC(=O)C(Cc1ccc(N2CCN(c3ccc(F)cc3F)CC2)cc1)NC(=O)c1ccccc1
**26**			COc1cc(CCC(=O)NC(Cc2ccccc2)C(=O)NC(Cc2ccccc2)C(N)=O)on1

Fig. S5. Identified side products in Mini-SEL1

	**Truncated**	**Hydrolyzed**	**Dehalogenated**
**1**	CC(O)C(NC(=O)C1(c2ccc(Br)cc2)CC1)C(N)=O	CC(O)C(NC(=O)C1(c2ccc(O)cc2)CC1)C(N)=O	NC(=O)C(CC(=O)O)NC(=O)c1cc(-c2ccccc2)on1
**2**	N=C(N)NCCCC(NC(=O)C1(c2ccc(Br)cc2)CC1)C(N)=O	CC(O)C(NC(=O)c1cc(-c2ccc(O)cc2)on1)C(N)=O	NC(=O)C(CC(=O)O)NC(=O)C1(c2ccccc2)CC1
**3**	N=C(N)NCCCC(NC(=O)Cc1ccc(Br)cc1)C(N)=O	N=C(N)NCCCC(NC(=O)C1(c2ccc(O)cc2)CC1)C(N)=O	NCCCCC(NC(=O)C1(c2ccccc2)CC1)C(N)=O
**4**	N=C(N)NCCCC(NC(=O)c1cc(-c2ccc(Br)cc2)on1)C(N)=O	N=C(N)NCCCC(NC(=O)c1cc(-c2ccc(O)cc2)on1)C(N)=O	C1=CC=C(C=C1)CC(=O)NC(CCCN=C(N)N)C(=O)N
**5**	NC(=O)C(CC(=O)O)NC(=O)C1(c2ccc(Br)cc2)CC1		N=C(N)NCCCC(NC(=O)c1cc(-c2ccccc2)on1)C(N)=O
**6**	NCCCCC(NC(=O)Cc1ccc(Br)cc1)C(N)=O		N=C(N)NCCCC(NC(=O)C1(c2ccccc2)CC1)C(N)=O

Fig. S6. Identified side products in Mini-SEL2

a)

SEL1	# sulfonamide	# non sulfonamide	Total
After AS-MS	13	8	21
Before AS-MS	330	25374	25704
Total	343	25382	25725
p = 6.20e-20			

b)

```
####### SEL #######

library_size <- 49 * 7 * 75

strcts_w_substrct <- 49 * 7

compounds_after_selection <- 21

found_binders <- 13


structs_wo_substrct <- library_size – strcts_w_substrct

nonHits_w_substrct <- strcts_w_substrct - found_binders

hits_wo_substrct <- compounds_after_selection - found_binders

nonHits_wo_subsrct <- library_size - compounds_after_selection - nonHits_w_substrct


data <- data.frame(

"sulfonamide containing structures" = c(found_binders, nonHits_w_substrct),

"structures without sulfonamide" = c(hits_wo_substrct, nonHits_wo_subsrct),

row.names=c("After AS-MS", " Before AS-MS")

)

fisher.test(data)$p.value
```

Fig. S7. P value calculations

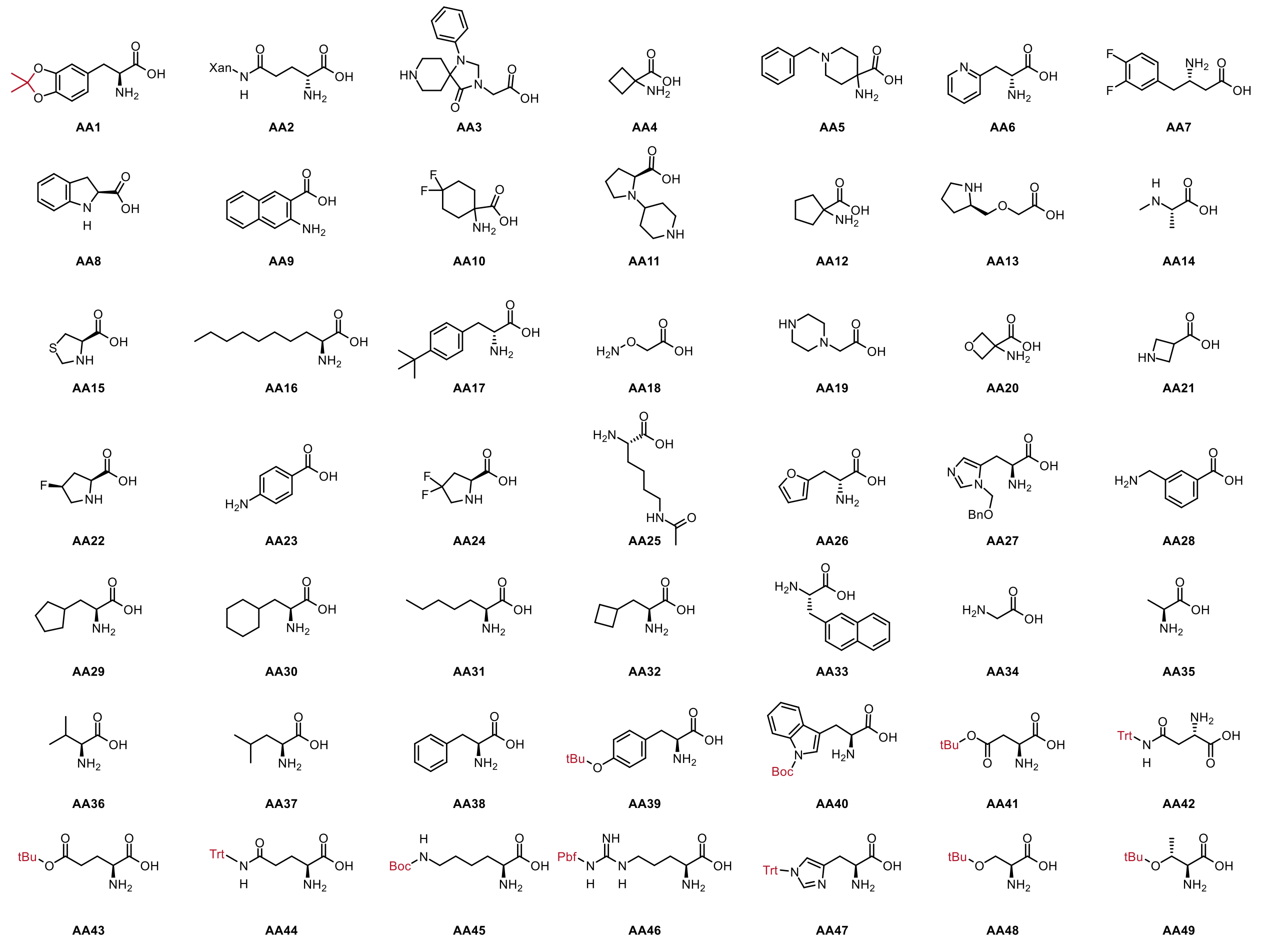

Fig. S8. Amino acid building blocks used to synthesize the SEL

Fmoc protected variants of the amino acids were used in the library synthesis. The protecting groups indicated in red are removed upon cleavage.

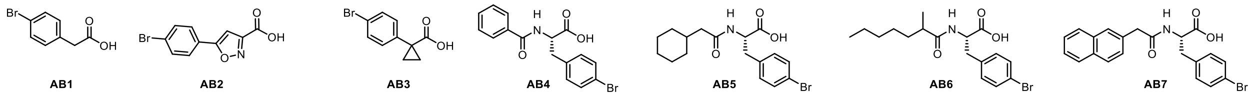

Fig. S9. Aryl bromide building blocks used to synthesize the SEL

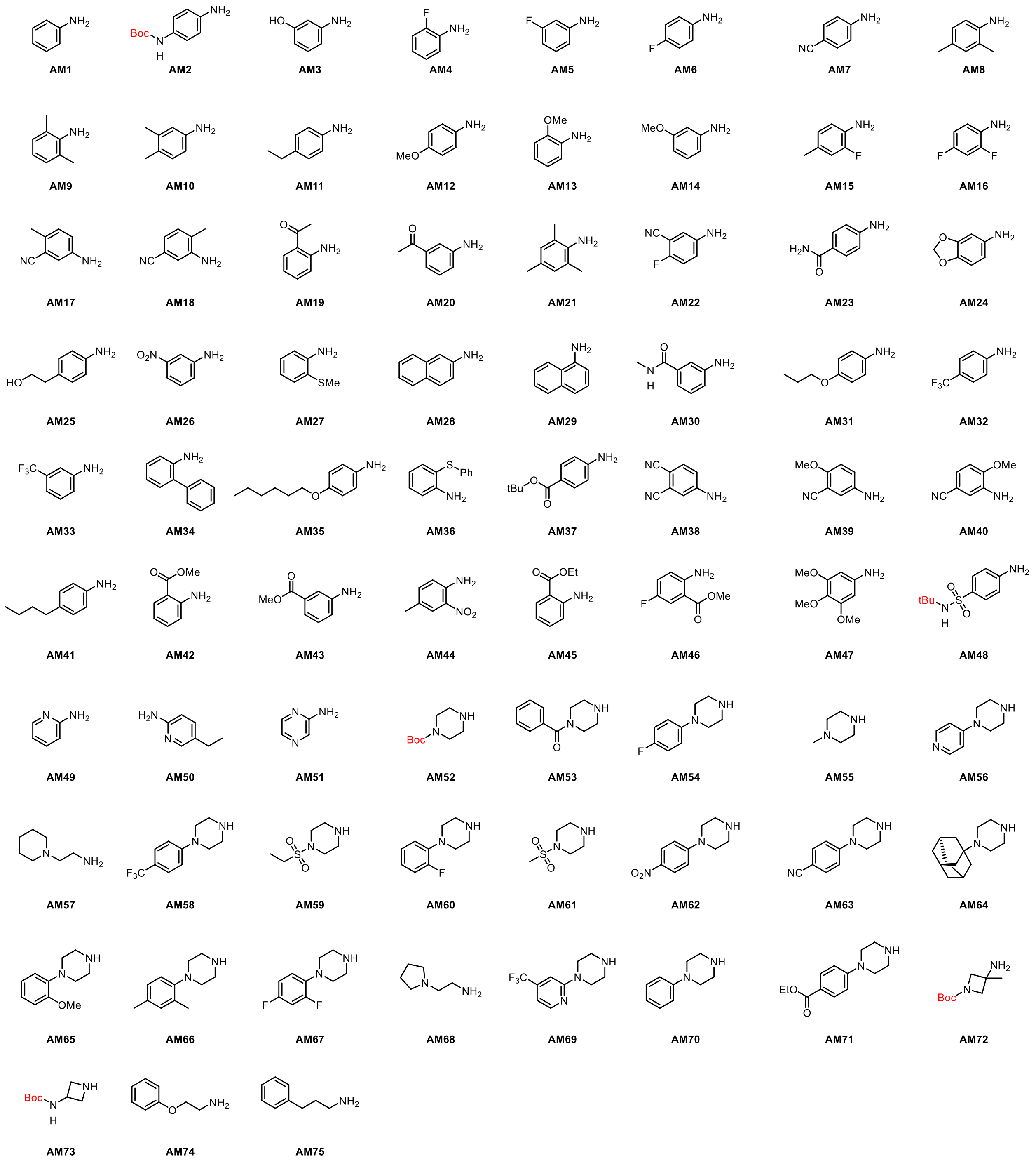

Fig. S10. Amine building blocks used to synthesize the SEL.
The protecting groups indicated in red are removed upon cleavage.

2. Abbreviations

APC	Allylpalladium(II) chloride dimer
AS	Affinity Selection
AS-MS/MS	Affinity Selection-Tandem Mass Spectrometry
BB	Building block
BLI	Biolayer interferometry
Boc	*tert*-butoxycarbonyl
BSA	Bovine serum albumine
CAIX	Carbonic anhydrase IX
DCM	Dichloromethane
DEL	DNA-encoded library
DIPEA	Di-isopropylethylamine
DMF	Dimethylformamide
DMSO	Dimethylsulfoxide
Et	Ethyl
Eq	Equivalent
FA	Formic acid
FBS	Fetal bovine serum
Fmoc	Fluorenylmethoxycarbonyl
HATU	Hexafluorophosphate Azabenzotriazole Tetramethyl Uronium
HTS	High-Throughput Screening
iPr	Isopropyl
LC-MS	Liquid Chromatography-Mass Spectrometry
LC-MS/MS	Liquid Chromatography-Tandem Mass Spectrometry
Me	Methyl
MeCN	Acetonitrile
MeOH	Methanol
MW	Molecular weight
MQ	MilliQ
Pbf	2,2,4,6,7-Pentamethyl-2,3-dihydrobenzofuran-5-sulfonyl
PBS	Phosphate-buffered saline
Ppm	Parts per million
r.t.	Room temperature
SA	Streptavidin
SEL	Self-encoded library
t-Bu	*tert*-Butyl
TFA	Trifluoroacetic acid
TIPS	Triisopropylsilane
TPSA	Topological polar surface area
Trt	Trityl
Tween-20	Polysorbate 20

3. Material and Methods

3.1 Reagents and supplies

Chemicals: Reagents and solvents were purchased from Sigma-Aldrich (Merck), Fisher Scientific, BLDpharm, Fluorochem, Carl Roth and VWR and were used without further purification unless stated otherwise. Fmoc-protected amino acid building blocks were partially purchased from Chemspace. Tentagel® S NH2 90 µm (S30902) and TentaGel® M NH_2 30 µm (M30352) were purchased from Rapp-Polymere.

Materials for affinity selection: Dynabeads MyOne Streptavidin T1 were purchased from ThermoFisher Scientific. Biotinylated Human Carbonic Anhydrase IX (38-414), His,Avitag (CA9-H82E3) and Human Carbonic Anhydrase IX (38-414), His,Avitag (CA9-H5226) were purchased from ACROBiosystems. A KingFisher™ Duo Prime Purification System was used to perform the affinity selection experiments. The protocols were developed with BindIt 4.1 Software.

3.2 Instrumentation

LC-MS analysis: Compound purity was determined by LC–MS, using the LCMS-2020 system (Shimadzu) with a Gemini 3 µm C18 110 Å column (50 × 3 mm) using the following parameters: flow rate = 0.55 mL/min, scan range = 160-800 m/z, column temperature (°C) = 40. The following gradient of 10−90% MeCN/H_2O (0.1% formic acid) over 15 min and measuring UV absorbance at 254 nm was used, unless stated otherwise. Compounds were dissolved in H_2O:MeCN:*t*-BuOH (1:1:1) before injection.

LC-MS/MS analysis: Analysis was performed on an Vanquish™ Neo UHPLC system (Thermo Scientific) connected to an Orbitrap Exploris 240 mass spectrometer (Thermo Fisher Scientific). Samples were run on a Double nanoViper PepMap Neo column (2 µm particle size, 15 cm x 75 µm, Thermo Fisher Scientific, DNV75150PN) following a PepMap Neo Trap Cartridge (5 µm C18 300 µm X 5 mm). The standard nano-LC method was run without temperature control and a flow rate of 250 nL/min with the following gradient: 3% solvent B ramping linearly to 60% B in A over 60 min, from 60% B to 95% B over 6 min and at 95% B for 5 min, with solvent A = water (0.1% FA), and solvent B = 80% acetonitrile, 20% water (0.1% FA). Positive spray ionization was set at 1900 V. The following parameters were used for MS1 collection: resolution = 120.000, scan range = 250-875 m/z, maximum injection times = 300 ms, RF Lens = 80%, microscans = 1, AGC target = standard, Ion Transfer Tube Temp (°C) = 280, Charge State = +1. For the data dependent MS/MS event the following settings were used: resolution = 15000, number of Dependent Scans= 20, isolation window (m/z) = 1.5, intensity threshold = 1E5, MIPS mode = small molecule, absolute collision energy = 15, 25 eV, AGC Target (%) = 50, microscans = 1, RF Lens(%) = 70, dynamic exclusion = on (exclude after n times = 3, Exclusion duration (s) = 15, excluding isotopes, 10 ppm mass tolerance).

Purification: The automatic flash chromatography was performed on a Biotage Selekt System with pre-packed flash cartridges (Biotage® Sfär Bio C18 - Duo 300 Å 20 µm). MQ 0.1% TFA (buffer A) and MeCN 0.1% TFA (buffer B) were used as mobile phase.

3.3 General procedures

General procedure A: Manual solid phase synthesis (SPS):

TentaGel S NH_2 resin (90 µm, 0.26 mmol/g, 1.0 eq.) was functionalized with a Rink linker using the following protocol before attaching other building blocks.

TentaGel S NH_2 resin (90 µm, 0.26 mmol/g, 1.0 eq.) was loaded onto a fritted syringe and swelled for 5 min in DMF. A solution of Fmoc-protected amino-acid (3.0 eq), HATU (0.4 M, 2.98 eq) and DIPEA (9.0 eq) in DMF was added to the resin. After 20 min the resin was washed with DMF (5x) and Fmoc deprotection was performed by washing with piperidine (20% in DMF, 1x) before incubating with piperidine (20% in DMF) for 10 min. The resin was washed with DMF (5x) before adding the next Fmoc-protected amino-acid. The protocol was repeated until completion of the synthesis.

The compounds were cleaved of the resin by incubating at room temperature for 1 hour with a solution of TFA:H_2O:TIPS (92.5:5:2.5) and washed with TFA:H_2O:TIPS (92.5:5:2.5). The volume was reduced by evaporating the TFA solution with a N_2 stream. The reaction mixture was dissolved in H_2O:MeCN:*t*-BuOH (1:1:1) before injection into a LC-MS.

General procedure B: Buchwald-Hartwig reaction with aromatic amines

K_2CO_3 (2.76 mg, 20 µmol, 2 eq.), XPhos (4.77 mg, 10 µmol, 1.0 eq.), amine (20 µmol, 2.0 eq.) and $Pd(OAc)_2$ (1.22 mg, 5 µmol, 0.5 eq.) were dissolved in anisole:H_2O (9:1, 300 µL) and added to the TentaGel S NH_2 resin (10 µmol, 1.0 eq.). The reaction was performed in an 1.5 mL eppendorf and purged with N_2 prior to sealing the Eppendorf tubes. The reaction was stirred at 80°C for 21h. The resin was washed with DMF (5 x 2 mL) and DCM (5 x 2 mL) before cleaving with a solution of TFA:H_2O:TIPS (92.5:5:2.5) for 1h and washed once with a solution of TFA:H_2O:TIPS (92.5:5:2.5).

General procedure C: Buchwald-Hartwig reaction with aliphatic amines

K_2CO_3 (2.76 mg, 20 µmol, 2.0 eq), tBuXPhos (3.40 mg, 8 µmol, 0.8 eq.), amine (20 µmol, 2.0 eq.), and $Pd_2(dba)_3$ (1.83 mg, 2 µmol, 0.2 eq.) were dissolved in toluene:H_2O (9:1, 300 µL) and added to the TentaGel S NH_2 resin (10 µmol, 1.0 eq.). The reaction was performed in an 1.5 mL eppendorf and purged with N_2 prior to sealing the Eppendorf tubes. The reaction was stirred at 80°C for 18h. The resin was washed with DMF (5 x 2 mL) and DCM (5 x 2 mL) before cleaving with a solution of TFA:H_2O:TIPS (92.5:5:2.5) for 1h and washed once with a solution of TFA:H_2O:TIPS (92.5:5:2.5).

Affinity selection: A KingFisher™ Duo Prime Purification System was used to perform the affinity selection experiments. The protocols were developed with BindIt 4.1 Software. MyOne Streptavidin T1 Dynabeads (1 mg) were washed with 3x 1 mL 10% FBS, 1x PBS, 0.02% Tween-20. The beads were incubated with biotinylated CAIX (150 pmol) in 10% FBS, 1x PBS, 0.02% Tween-20 for 1h at 10°C. The beads were washed with 2x 1 mL 10% FBS, 1x PBS, 0.02% Tween-20, 400 µM d-biotin and 1x 1 mL 10% FBS, 1x PBS, 0.02% Tween-20 before incubating with the library (100 µL, 100 fmol/member) in 10% FBS, 1x PBS for 1h at 10°C. The beads were washed with 5x 1 mL of 1x PBS and subsequently eluted with 2x MeCN:MQ (1:1) 0.1%FA (100 µL per well). An overview of the King Fisher protocol as described in Fig. S8.

	General	Beginning of step		Mixing/Heating		End of step		
	Volume (µL)	Release time (s)	Release speed	Mixing time (min)	Temp	Mixing speed	Collect count	Collect time (s)
Bead uptake	100	-	-	5	r.t.	Bottom mix	5	10
Bead washing (3x)	1000	30	Medium	3	r.t.	Medium	5	10
Protein incubation	100	30	Medium	60	10 ºC	Medium	5	10
Biotin blocking (2x)	1000	30	Medium	10	r.t.	Medium	5	10
Bead washing	1000	30	Medium	3	r.t.	Medium	5	10
Library Incubation	1000	30	Medium	60	10 ºC	Medium	5	10
Bead washing (5x)	1000	30	Medium	0.5	r.t.	Medium	5	10
Elution (2x)	100	30	Medium	3	r.t.	Medium	5	10

Fig. S11. Overview of the KingFisher program used for affinity selection.

Sample preparation after AS: Samples from the affinity selection procedure were lyophilized and resuspended in 50 µL MQ 0.1%FA. The StageTips were prepared as described by Rappsilber et al. using C18 material from Empore SPE 47 mm discs (66883-U, Merck). The StageTips were pre-conditioned with 200 µL MeOH, 200 µL of 0.1% (v/v) FA in MeCN and 200 µL of 0.1% (v/v) FA in MQ, respectively by centrifuging for 3 min at 300 rcf. The samples were then loaded on the StageTips and washed with 200 µL of 0.1% (v/v) FA in MQ. Compounds were eluted by adding 200 µL of 0.1% (v/v) FA in MeCN:MQ (7:3). The samples were lyophilized before resuspending in 25 µL 0.1% (v/v) FA in UPLC-MS grade water. The samples were centrifuged for 5 min at 15.000 rpm. Afterwards, 23 µL was transferred to an LC-MS vial and 20 µL was injected into the LC-MS/MS system.

Biolayer interferometry (BLI):

Purified biotinylated compounds were dissolved to 1 µM in 1x PBS, 0.02% Tween-20, 1 mg/ml BSA (0.1% (w/v)) (kinetic buffer) and used for immobilization onto streptavidin Octet SA Biosensors (SATORIUS). Biolayer interferometry (BLI) assays were performed in 96 well plates (GreinerBio-One, polypropylene, flat-bottom, chimney well) using an Octet R4 system (SATORIUS). Wells were filled with 200 µL with kinetic buffer, compound solution or CAIX solution.

Biotinylated compound was immobilized onto the streptavidin biosensor for 60 s. Sensors were then dipped into kinetic buffer for 60 s, CAIX solution (1 µM, 500 nM, 250 nM, 125 nM) for 600 s and into kinetic buffer for 600s. Measurements were carried out at 30 °C.

3.4 Software

KNIME: KNIME 5.5.1 software was used for library enumeration and molecular property calculations. The following extensions were installed RDkit Nodes version 5.2.1. andCDK version 1.5.6. The KNIME workflow is available through Zenodo (10.5281/zenodo.17632652). The workflow and code involved are described in section 4.

Proteowizard (MSconvert)[1]**:** Raw files (.raw) from the Orbitrap Exploris 240 mass spectrometer were converted to .mzML files using MSConvert. The following settings were used: output format= mzML, binary encoding precision= 64-bit, write index= yes, use zlib compression= yes, TPP compatibility= yes, package in gzip= no, use numpress linear compression= no, use numpress short logging float compression= no. use numpress positive integer compression= no, combine ion mobility scans= no, SIM as spectra= no, SRM as spectra= no, filter= peak picking, Algorithm= Vendor, MS level= 1-2.

MZmine[2]**:** MZmine 4.5.0 was used to perform a background subtraction using a three-fold intensity threshold with samples measured against streptavidin-coated magnetic beads. The mzwizard feature was used using the following settings:

HPLC - Smoothing= yes. Stable ionization across samples= yes. Crop retention time= 0.00-78.00 min. Max peaks in chromatography= 15. Minimum consecutive scans= 4. Approximate feature FWMN= 0.10 min. RT tolerance (intra-sample)= 0.08 min. RT tolerance (sample-to-sample)= 0.40 min.

Orbitrap - Ion mode= positive with absolute intensity. Noise threshold: MS1= 1.0E5, MS2= 0. Minimum feature height= 3.0E5. m/z tolerance (scan-to-scan)= 0.0010 m/z or 5.0 ppm. m/z tolerance (intra-sample)= 0.0015 m/z or 5.0 ppm. m/z tolerance (sample-to-sample)= 0.0015 m/z or 5.0 ppm.

Filters - Original feature list= remove. Min samples per aligned feature= max of 1 sample or 0.0%. Only keep features with 13C= no.

Annotation - Local compound database search= no. Annotate lipids= no.

The generated feature list was used in the Feature list blank subtraction using the following parameters: Minimum # detection in blanks= 1. Quantification= height. Ratio type= maximum. Fold charge increase= 300%. The subtracted feature list was subsequently exported to SIRIUS.

4. Library enumeration

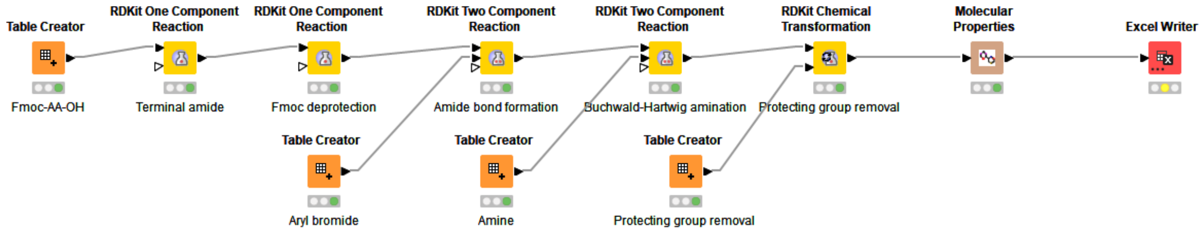

Fig. S12. Overview of the KNIME workflow used to enumerate the library and to calculate the molecular properties.

Table S1. The reaction SMARTS used for library enumeration

Reaction	Reaction SMARTS
COOH -> CONH2	[#8;A;X1H0-,X2H1:4][#6;A;X3:2]=[O:1]>>[#7;A;X3:3][#6;X3:2]=[O;X1:1]
Fmoc-deprotection	[#7:17]-[#6:16](=[O:18])-[#8:15]-[#6:14]-[#6:1]-1-[c:5]2[c:9][c:8][c:7][c:6][c:4]2-[c:3]2[c:10][c:11][c:12][c:13][c:2]-12>>[#7:17].[O:18]=[#6:16]-[#8:15]-[#6:14]-[#6:1]-1-[c:2]2[c:13][c:12][c:11][c:10][c:3]2-[c:4]2[c:6][c:7][c:8][c:9][c:5]-12
Amide bond formation	[#7;A;X3;H2,H1;!$(NC=O);!$(NC=CC=O);!$(NC=S);!$(NC=N)!$(N-S):3].[#8;A;X1H0-,X2H1][#6;A;X3:2]=[O:1]>>[#7;A;X3:3][#6;X3:2]=[O;X1:1]
Buchwald-Hartwig amination	Br[c:3].[#6:1][N;!H0;!$(NS(=O)=O);!$(NC([C;H3])([C;H3])[C;H3]);!$(NC=O):2]>>[#6:1][N:2][c:3]
Trt	[N:13][C:14]([c:12]1[c:1][c:2][c:3][c:4][c:5]1)([c:11]1[c:6][c:7][c:8][c:9][c:10]1)[c:15]1[c:16][c:17][c:18][c:19][c:20]1>>[N;A;X3,H2:13]
Trt	[n:13][C:14]([c:12]1[c:1][c:2][c:3][c:4][c:5]1)([c:11]1[c:6][c:7][c:8][c:9][c:10]1)[c:15]1[c:16][c:17][c:18][c:19][c:20]1>>[n;A;X3,H1:13]
Boc	[N:3]-[#6:4](=[O:10])-[#8:5][C:6]([#6:7])([#6:8])[#6:9]>>[N;A;X3:3]
Boc	[n:3]-[#6:4](=[O:10])-[#8:5][C:6]([#6:7])([#6:8])[#6:9]>>[n;A;X3;H1:3]
OtBu	[#6;H3:5][C:2]([#6;H3:4])([#6;H3:3])[#8:1]>>[#8:1]
NtBu	[#6;H3:5][C:2]([#6;H3:4])([#6;H3:3])[#7:1]>>[#7:1]
Pbf	[#6:11]-[#8:10]-[c:9]1[c:8][c:6](-[#6:7])[c:5]([c:14](-[#6:15])[c:12]1-[#6:13])[S:2]([#7:1])(=[O:3])=[O:4]>>[#7:1]
AA1	[#6:6][C:5]1([#6:7])[#8:4]-[c:3]2[c:2][c:1][c:11][c:10][c:9]2-[#8:8]1>>[#8:4]-[c:3]1[c:2][c:1][c:11][c:10][c:9]1-[#8:8]

5. SIRIUS:COMET

COMET software is available through GitHub (https://github.com/sirius-ms/comet). Here we used COMET v1.1.0-SNAPSHOT. Enumerated libraries generated by the above KNIME workflow were imported into COMET as custom structure databases using the GUI. Spectra files were imported as .mgf files that were generated from the described MZmine workflow.

The COMET filter can be accessed through the general filter dialog window of the GUI. For its application, we used the following settings (also depicted in Fig. S10): scaffold formula: left blank, fallback adducts= $[M+H]^+$. MS1 mass accuracy (ppm) = 5 ppm, considered fragment types = S[2;3;4;5;6], S[3;4;5;6],S[0;1;2], S[4;5;6], S[0;1;2;3], S[5;6], S[0;1;2;3;4], minimum number of matching peaks = 1, number of considered peaks = 5, number of allowed hydrogen shifts = 1, MS2 mass accuracy (ppm) = 5. The specification of such fragment types is illustrated in Fig. S4. For each library, a file containing information about all the library's building blocks has to be provided in COMET. The .csv file named "SEL_CustomDB_BBs.csv" was used to analyze AS-MS/MS data and is available through Zenodo (10.5281/zenodo.17632652)

After applying COMET, the remaining features are annotated using EPIMETHEUS. Molecular formula generation was performed using formula database search in the imported custom library using $[M+H]^+$ as fall back adduct, all other settings were left to default. After fingerprint prediction (score threshold enabled), the imported custom library was used for structure database search. Structure candidates were ranked according to EPIMETHEUS.

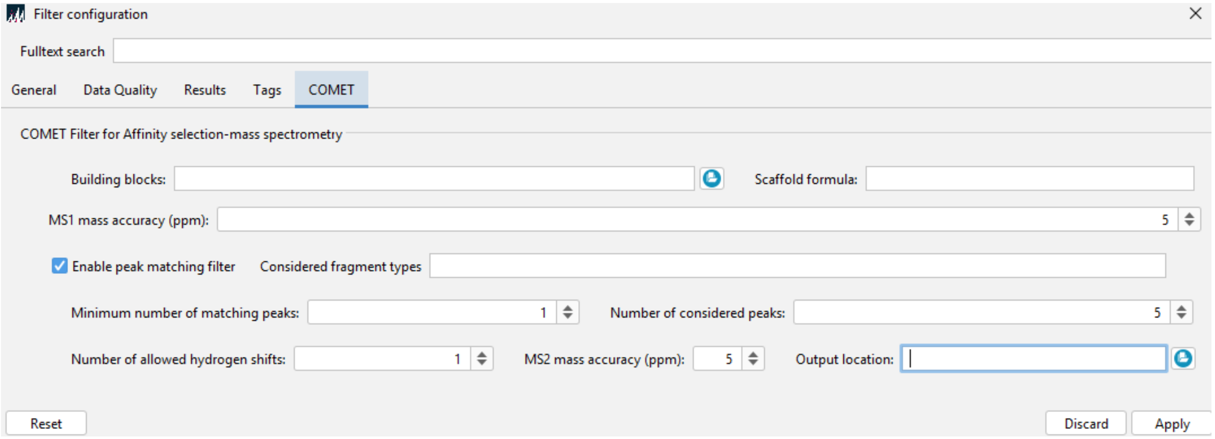

Fig. S13. Overview of parameters used for COMET

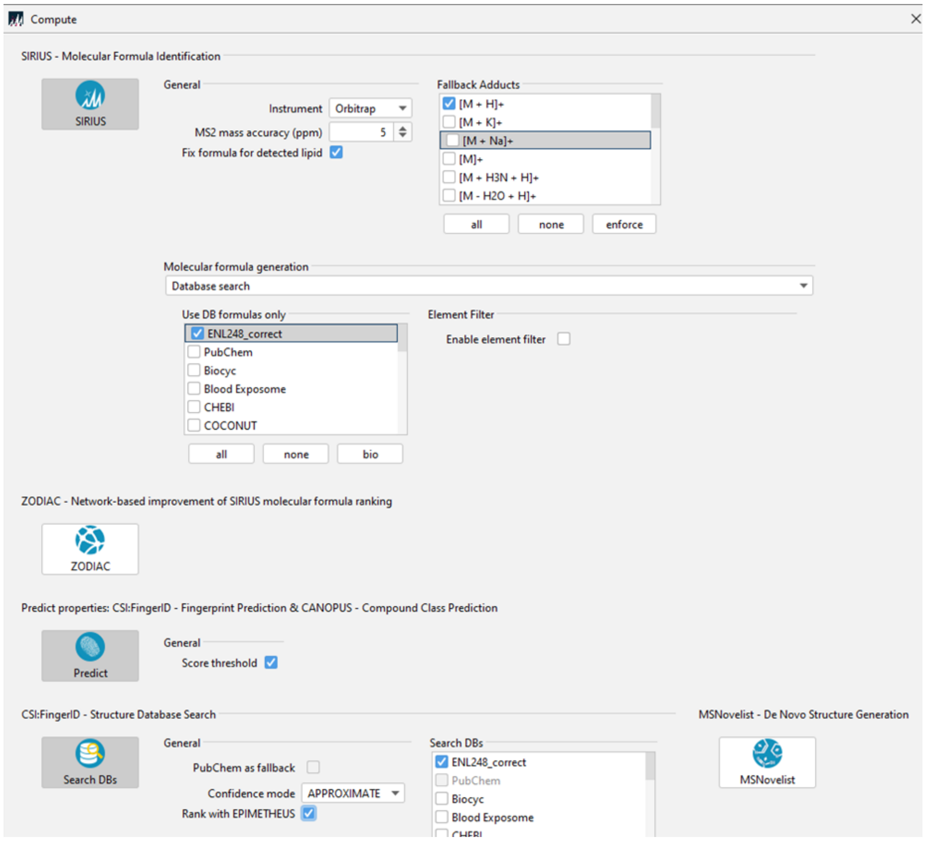

Fig. S14. Settings used for the structure annotation with EPIMETHEUS

6. Library synthesis

6.1 MiniSEL1

TentaGel S NH_2 resin (90 µm, 0.26 mmol/g loading, 385 mg, 100 µmol, 1.0 eq.) was transferred to a 5 mL fritted syringe and was subsequently swelled with DMF for 10 min. A solution of Fmoc-Rink-OH (539 mg, 1 mmol, 10.0 eq), 0.4M HATU (0.8 mmol, 2 mL, 8.0 eq.) and DIPEA (400 µL, 2.3 mmol, 23 eq) in DMF was added to the resin and reacted for 20 min. The resin was washed with DMF (5 x 3 mL) and 20% piperidine in DMF (1x, 2 mL) before incubating with 20% piperidine in DMF for 10 min.

The resin was divided over 5 x 2 mL fritted syringes. A solution of Fmoc-protected amino acid (200 µmol, 10.0 eq), HATU (400 µL, 0.4 M, 160 µmol, 8.0 eq) and DIPEA (80 µL, 460 µmol, 23.0 eq) in DMF was added to the resin (20 µmol). After 20 min the resin was combined in a 10 mL fritted syringe and was washed with DMF (5 x 2 mL) and 20% piperidine in DMF (1x, 2 mL) before incubating with 20% piperidine in DMF for 10 min. The resin was washed with DMF (5 x 2 mL) before adding the next Fmoc-protected amino-acid.

The resin was divided over 6 x 2 mL fritted syringes. A solution of aryl bromide (167 µmol, 10.0 eq), HATU (334 µL, 0.4 M, 133 µmol, 8.0 eq) and DIPEA (67 µL, 383 µmol, 23.0 eq) in DMF was added to the resin (16.67 µmol) and reacted for 20 min. The 3 syringes that reacted with Fmoc-Phe(4-Br)-OH were washed with DMF (5 x 3 mL) and 20% piperidine in DMF (1x, 2 mL) before incubating with 20% piperidine in DMF for 10 min. The syringes were coupled with benzoic acid, cyclohexane acetic acid or 3-(3-Methoxy-5-isoxazolyl)propanoic acid (167 µmol, 10.0 eq), HATU (334 µL, 0.4 M, 133 µmol, 8.0 eq) and DIPEA (67 µL, 383 µmol, 23.0 eq) in DMF was added to the resin (16.67 µmol) and reacted for 20 min. The resin from all 6 syringes were combined in a 10 mL fritted syringe and was washed with DMF (5 x 2 mL).

The resin was divided over 10 x 1.5 mL Eppendorf tubes. The Buchwald–Hartwig amination was conducted following general procedure B or C, selected based on the amine used.

The resin was incubated for 1.5 hour with a solution of TFA:H_2O:TIPS (92.5:5:2.5) and washed once with a solution of TFA:H2O:TIPS (92.5:5:2.5). TFA was evaporated and the library was purified with using reverse phase column chromatography with a stepwise gradient 00-70-100% MeCN. The sample was lyophilized and diluted with MQ 0.1%FA to a concentration of 30 µM and measured at 100 fmol/member via LC-MS/MS.

6.2 MiniSEL2

TentaGel S NH_2 resin (90 µm, 0.26 mmol/g loading, 769 mg, 200 µmol, 1.0 eq.) was transferred to a 10 mL fritted syringe and was subsequently swelled with DMF for 10 min. A solution of Fmoc-Rink-OH (1079 mg, 2.0 mmol, 10.0 eq), HATU (1.6 mmol, 0.4M, 4 mL, 8.0 eq.) and DIPEA (800 µL, 4.6 mmol, 23 eq) in DMF was added to the resin and reacted for 20 min. The resin was washed with DMF (5 x 5 mL) and 20% piperidine in DMF (1x, 5 mL) before incubating with 20% piperidine in DMF for 10 min.

The resin was divided over 5 x 2 mL fritted syringes. A solution of Fmoc-protected amino acid (400 µmol, 10.0 eq), HATU (800 µL, 0.4 M, 320 µmol, 8.0 eq) and DIPEA (160 µL, 920 µmol, 23.0 eq) in DMF was added to the resin (40 µmol). After 20 min the resin was combined in a 10 mL fritted syringe and was washed with DMF (5 x 5 mL) and 20% piperidine in DMF (1x, 5 mL) before incubating with 20% piperidine in DMF for 10 min. The resin was washed with DMF (5 x 5 mL) before adding the next Fmoc-protected amino-acid.

The resin was divided over 3 x 2 mL fritted syringes. A solution of aryl bromide (667 μmol, 10.0 eq), HATU (1.34 mL, 0.4 M, 534 μmol, 8.0 eq) and DIPEA (267 μL, 1534 μmol, 23.0 eq) in DMF was added to the resin (66.7μmol) and reacted for 20 min.

The resin was divided over 20 x 1.5 mL Eppendorf tubes The Buchwald–Hartwig amination was conducted following general procedure B or C, selected based on the amine used.

The resin was incubated for 1.5 hour with a solution of TFA:H_2O:TIPS (92.5:5:2.5) and washed once with a solution of TFA:H2O:TIPS (92.5:5:2.5). TFA was evaporated and the library was purified with using reverse phase column chromatography with a stepwise gradient 00-70-100% MeCN. The sample was lyophilized and diluted with MQ 0.1%FA to a concentration of 30 μM and measured at 100 fmol/member via LC-MS/MS.

6.3 SEL

TentaGel M NH_2 (30 μm, 0.30 mmol/g loading, 1.27 g, 380 μmol, 1.0 eq.) was transferred to a 10 mL fritted syringe and was subsequently swelled with DMF for 10 min. A solution of Fmoc-Rink-OH (2.05 g, 3.8 mmol, 10.0 eq), HATU (3.04 mmol, 0.4 M, 7.6 mL, 8.0 eq.) and DIPEA (1.52 mL, 8.74 mmol, 23 eq) in DMF was added to the resin and reacted for 2h. The resin was washed with DMF (5 x 5 mL) and 20% piperidine in DMF (1x, 5 mL) before incubating with 20% piperidine in DMF for 10 min.

The resin was divided over 49 x 2 mL fritted syringes. A solution of Fmoc-protected amino acid (38.75 μmol, 5.0 eq), HATU (77.5 μL, 0.4 M, 31 μmol, 4.0 eq) and DIPEA (15.5 μL, 89 μmol, 11.5eq) in DMF was added to the resin (7.75 μmol). After 20 min the resin was combined in a 10 mL fritted syringe and was washed with DMF (5 x 5 mL) and 20% piperidine in DMF (1x, 5 mL) before incubating with 20% piperidine in DMF for 10 min. The resin was washed with DMF (5 x 5 mL) before adding the next Fmoc-protected amino-acid.

The resin was divided over 7 x 2 mL fritted syringes. A solution of aryl bromide (271 μmol, 5.0 eq), HATU (543 μL, 0.4 M, 217 μmol, 4.0 eq) and DIPEA (109 μL, 624 μmol, 11.5 eq) in DMF was added to the resin (54.3 μmol) and reacted for 20 min. The 3 syringes that reacted with Fmoc-Phe(4-Br)-OH were washed with DMF (5 x 3 mL) and 20% piperidine in DMF (1x, 2 mL) before incubating with 20% piperidine in DMF for 10 min. The syringes were coupled with benzoic acid, cyclohexane acetic acid, 2-methylheptanoic acid or 2-(naphthalen-2-yl)acetic acid (271 μmol, 5.0 eq), HATU (543 μL, 0.4 M, 217 μmol, 4.0 eq) and DIPEA (109 μL, 624 μmol, 11.5 eq) in DMF was added to the resin (54.3 μmol) and reacted for 20 min. The resin from all 7 syringes were combined in a 10 mL fritted syringe and was washed with DMF (5 x 5 mL).

The resin was divided over 75 x 1.5 mL Eppendorf tubes. The Buchwald–Hartwig amination was conducted following general procedure B or C, selected based on the amine used.

The resin was incubated for 1.5 hour with a solution of TFA:H_2O:TIPS (92.5:5:2.5) and washed once with a solution of TFA:H2O:TIPS (92.5:5:2.5). TFA was evaporated and the library was purified with using reverse phase column chromatography with a stepwise gradient 00-70-100% MeCN. The sample was lyophilized and diluted with DSMO to a concentration of 50 mM and used in affinity selection experiments as described in section 3.3.

7. Affinity selection against CAIX

7.1 List of hit structures

Table S2. Identified binders for CAIX after AS-MS

	SMILES
1	CC(C)C(NC(=O)c1cc(-c2ccc(Nc3ccc(S(N)(=O)=O)cc3)cc2)on1)C(N)=O
2	CC(O)C(NC(=O)c1cc(-c2ccc(Nc3ccc(S(N)(=O)=O)cc3)cc2)on1)C(N)=O
3	NC(=O)C(CCC(=O)O)NC(=O)c1cc(-c2ccc(Nc3ccc(S(N)(=O)=O)cc3)cc2)on1
4	NC(=O)C(CO)NC(=O)c1cc(-c2ccc(Nc3ccc(S(N)(=O)=O)cc3)cc2)on1
5	NC(=O)C(Cc1c[nH]cn1)NC(=O)c1cc(-c2ccc(Nc3ccc(S(N)(=O)=O)cc3)cc2)on1
6	NC(=O)C(Cc1ccccn1)NC(=O)c1cc(-c2ccc(Nc3ccc(S(N)(=O)=O)cc3)cc2)on1
7	NC(=O)C1(NC(=O)c2cc(-c3ccc(Nc4ccc(S(N)(=O)=O)cc4)cc3)on2)CCC1
8	NC(=O)C1CC(F)(F)CN1C(=O)c1cc(-c2ccc(Nc3ccc(S(N)(=O)=O)cc3)cc2)on1
9	NC(=O)C1CC(F)CN1C(=O)c1cc(-c2ccc(Nc3ccc(S(N)(=O)=O)cc3)cc2)on1
10	NC(=O)C1CN(C(=O)c2cc(-c3ccc(Nc4ccc(S(N)(=O)=O)cc4)cc3)on2)C1
11	NC(=O)CCC(NC(=O)c1cc(-c2ccc(Nc3ccc(S(N)(=O)=O)cc3)cc2)on1)C(N)=O
12	NC(=O)CN1CCN(C(=O)c2cc(-c3ccc(Nc4ccc(S(N)(=O)=O)cc4)cc3)on2)CC1
13	NC(=O)COCC1CCCN1C(=O)c1cc(-c2ccc(Nc3ccc(S(N)(=O)=O)cc3)cc2)on1

List of hit structures identified after performing AS-MS in duplicates on CAIX. Using COMET filter with a least matching 1 out of the 5 biggest peaks and 5 ppm accuracy, 13 unique structures containing building block **AM48** were identified.

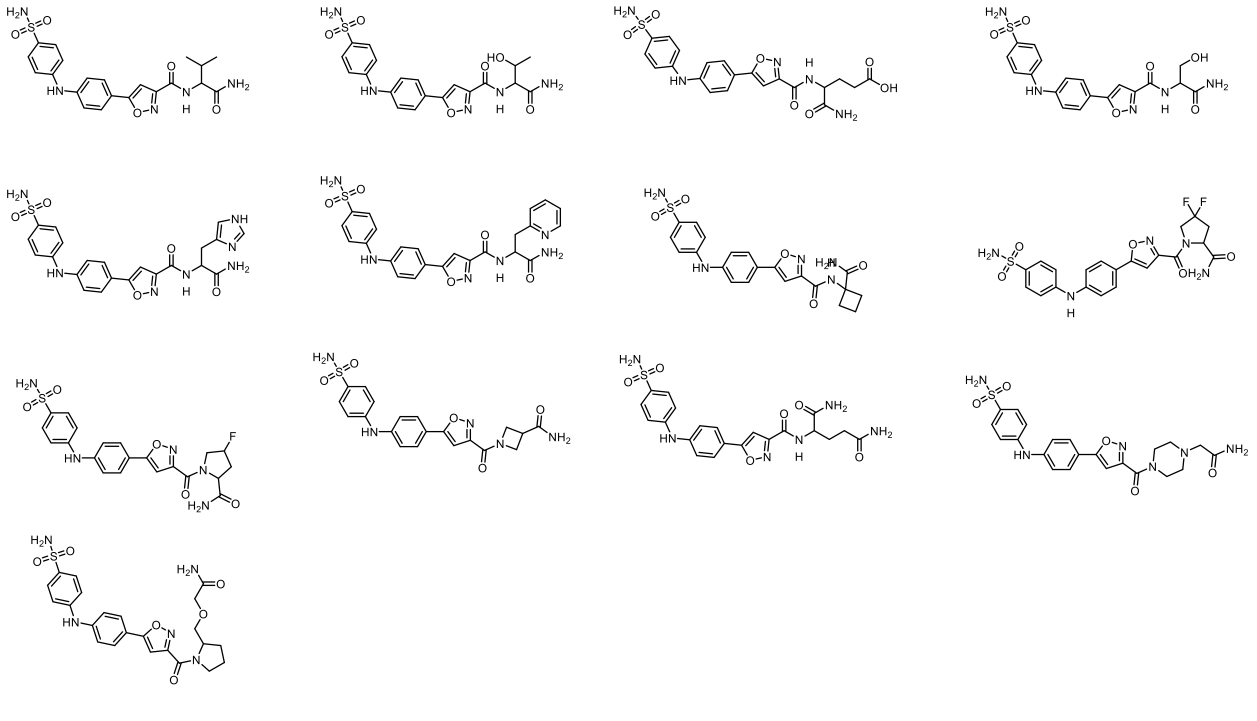

Fig. S15. Structures of the 13 hit compounds found after AS-MS against CAIX with SEL.

7.2 Enrichment plots

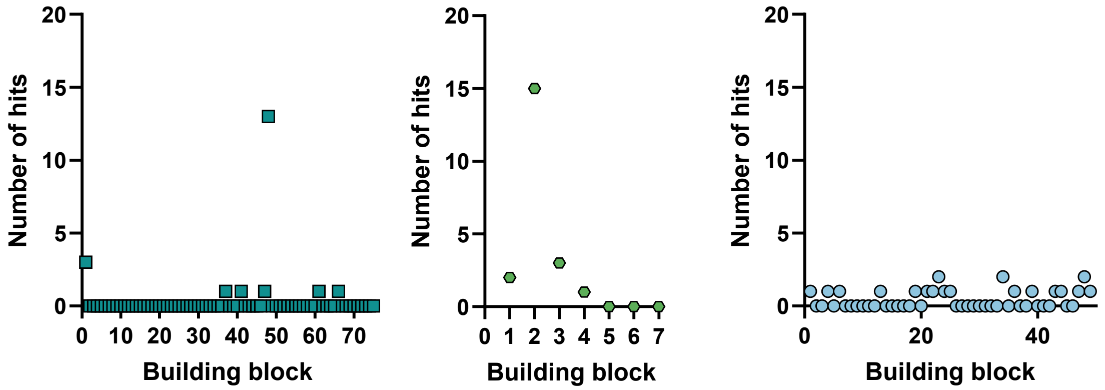

Fig. S16. Building block analysis after affinity selection with SEL against CAIX.
The top ranked structures by EPIMETHEUS were taken and deconstructed into the respective building blocks.

7.3 Hit identification

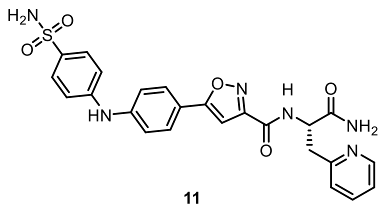

11

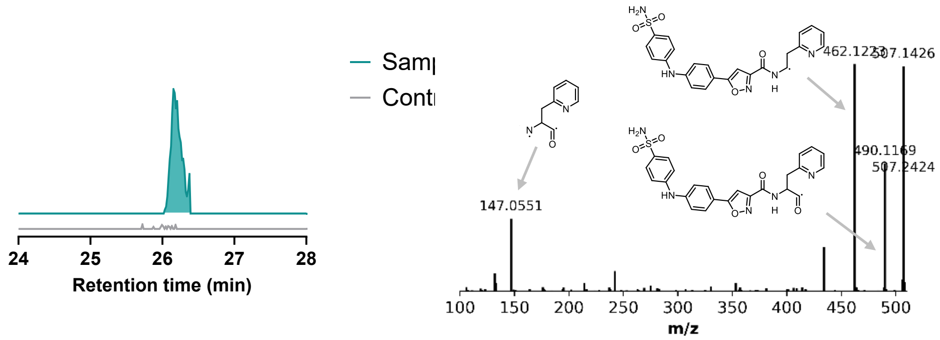

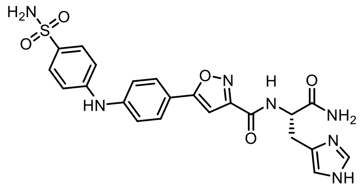

12

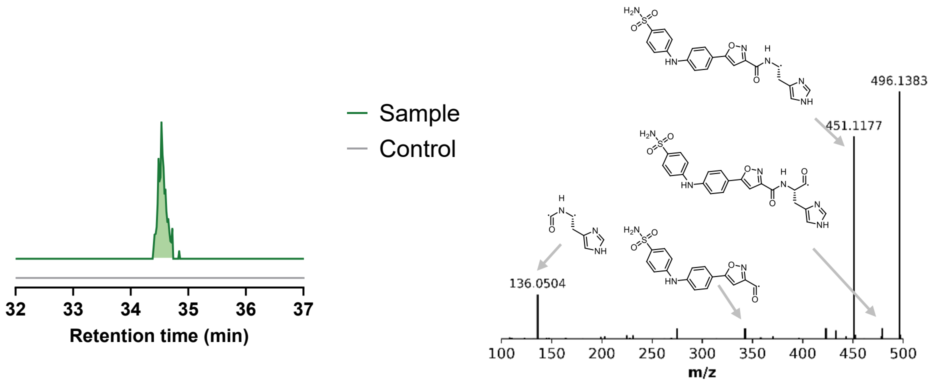

Fig. S17. Hits identified using COMET
Chemical structures of hit **11** and **12**, with the extracted ion chromatogram (EIC) and LC-MS/MS chromatogram from the affinity selection of SEL against CAIX.

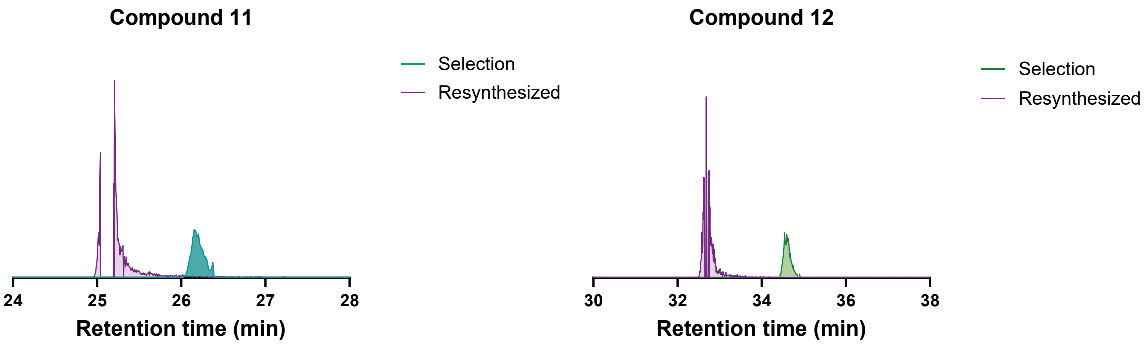

Fig. S18. Retention time comparison of the XIC traces after selection and the resynthesized compounds.

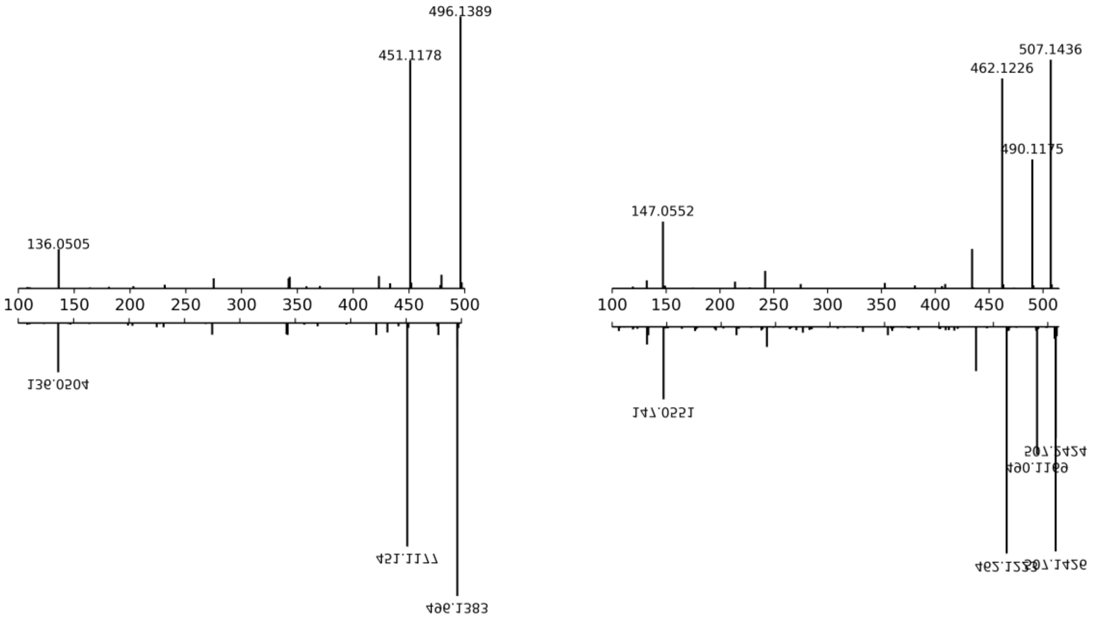

Fig. S19. Comparison of LC-MS/MS fragmentation after selection and the resynthesized compounds.

Top spectrum corresponds to the identified hit after affinity selection. The bottom spectrum corresponds to the resynthesized hit.

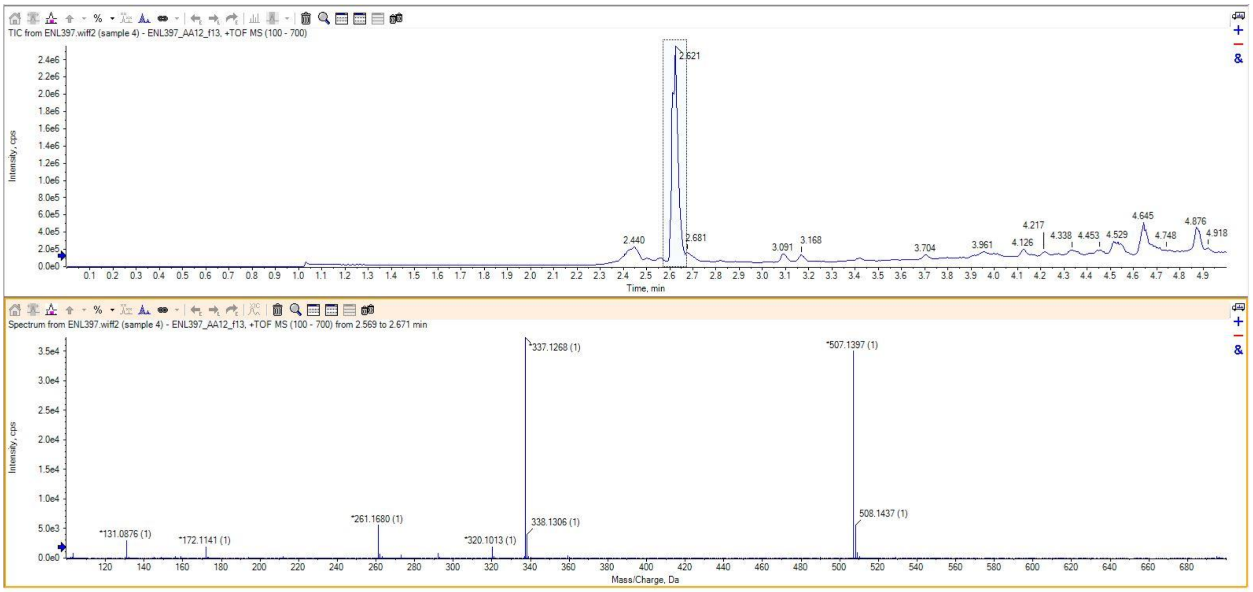

Fig. S20. LC-MS traces of resynthesized compound 12

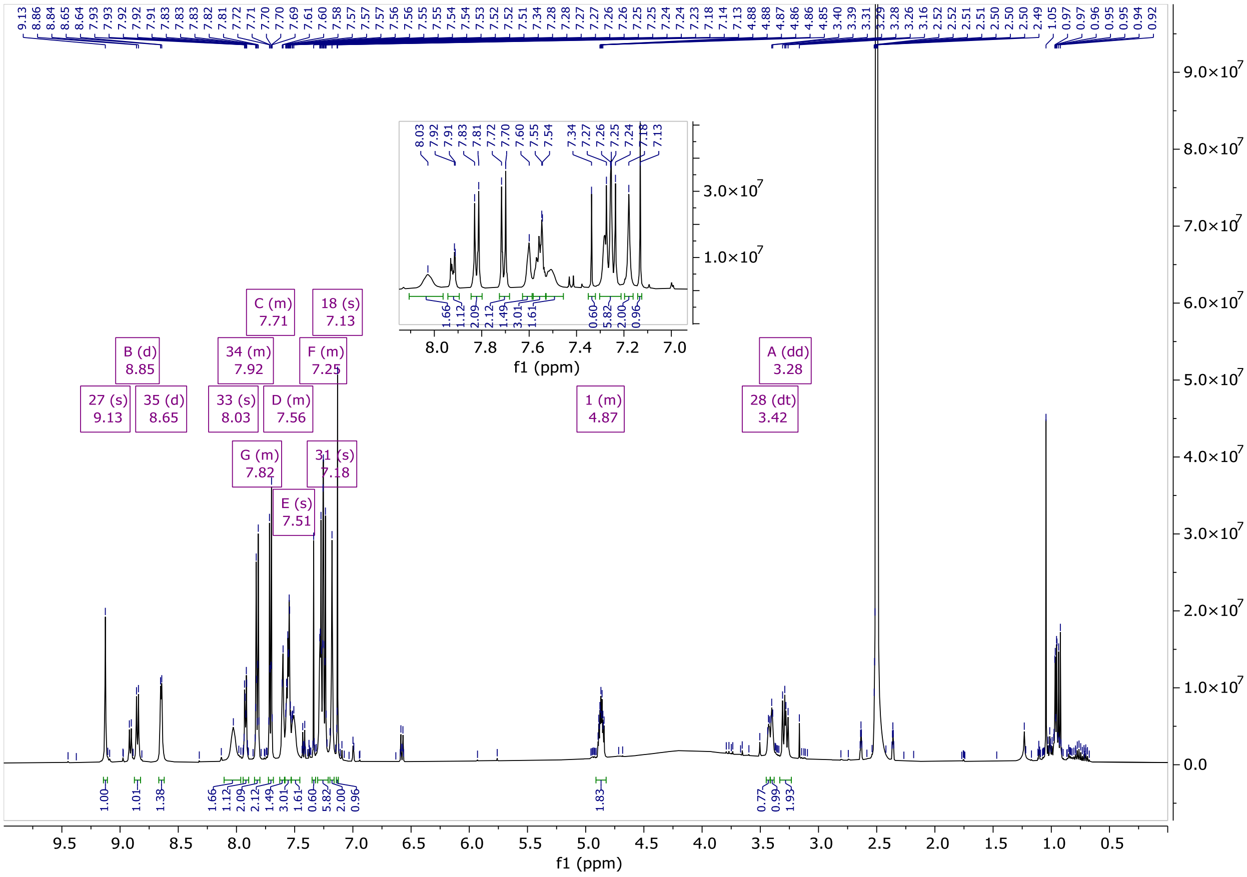

Fig. S21. NMR of resynthesized compound 12 (500 MHz, DMSO)

7.4 Hit resynthesis

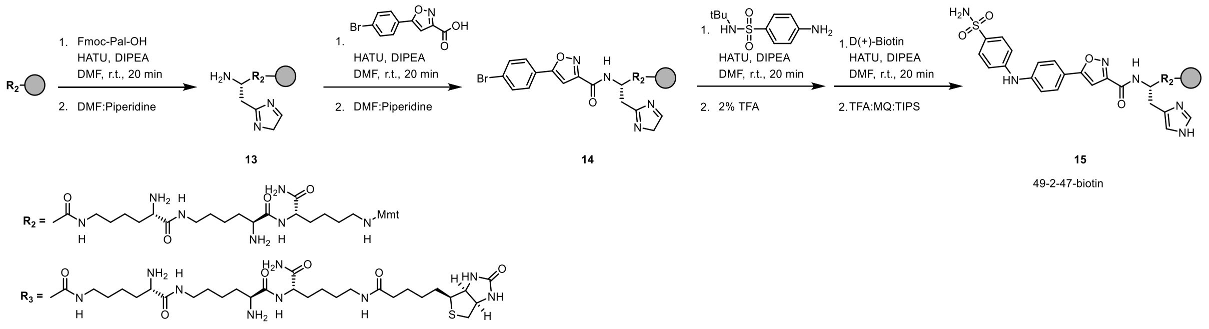

Product **15** was synthesized on a 100 µmol scale using reagent quantities described in general procedure A: Manual solid phase synthesis (SPS), as described in section 3.3 by respectively coupling Fmoc-Lys(Mmt)-OH (r.t., on.), Boc-Lys(Fmoc)-OH (2x)(2h, 70 ºC), Fmoc-His-OH (r.t., on.) and 5-(4-Bromophenyl)isoxazole-3-carboxylic acid (4h, 70 ºC)

The Buchwald-Hartwig cross coupling with 4-Amino-N-(tert-butyl)benzenesulfonamide was performed according to general procedure B.

The compound was cleaved of the resin by incubating at room temperature for 1 hour with a solution of TFA:H_2O:TIPS (92.5:5:2.5) and washed with TFA:H_2O:TIPS (92.5:5:2.5). The volume was reduced by evaporating the TFA solution with a N_2 stream. The crude mixture was purified by reverse phase column chromatography (C18, 00-35% MQ:MeCN) and product 15 was isolated in 9% yield (10.2 mg, 9.2 µmol, 52% purity). MS (ESI): m/z calculated for $C_{50}H_{71}N_{15}O_{10}S_2$ [M+H] = 1106.5028, found = 1106.60

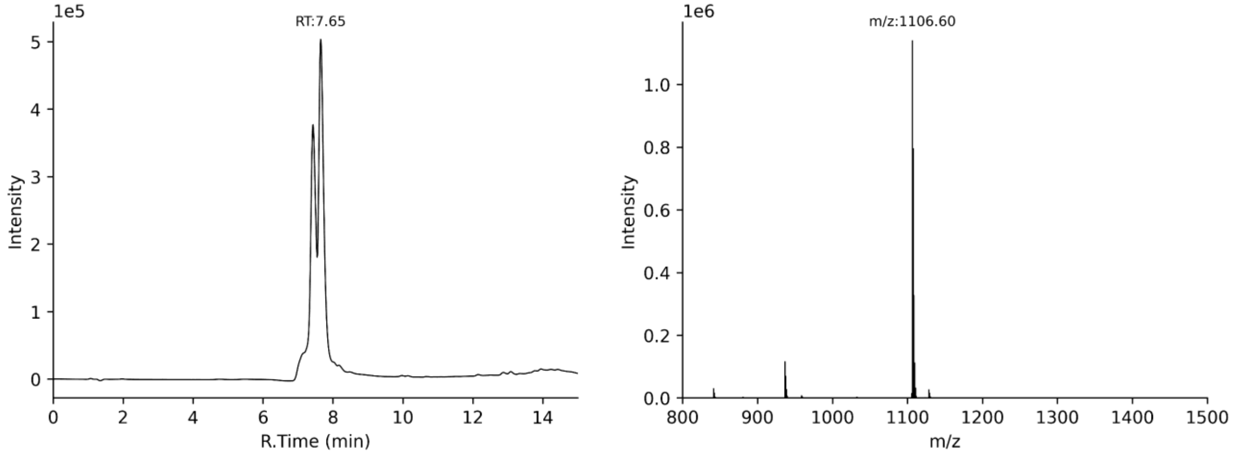

Fig. S22. LC-MS chromatogram from purified compound 15.

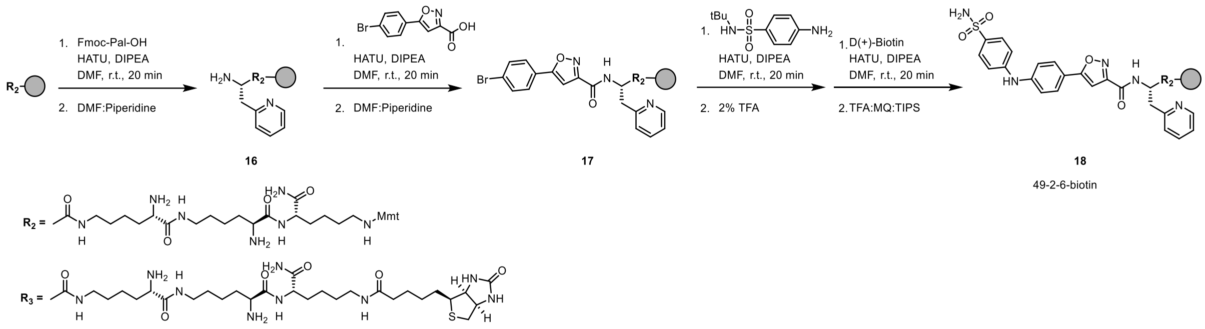

Product **18** was synthesized on a 100 µmol scale using reagent quantities described in general procedure A: Manual solid phase synthesis (SPS), as described in section 3.3 by respectively coupling Fmoc-Lys(Mmt)-OH (r.t., on.), Boc-Lys(Fmoc)-OH (2x)(2h, 70 ºC), Fmoc-Pal-OH (r.t., on.) and 5-(4-Bromophenyl)isoxazole-3-carboxylic acid (4h, 70 ºC)

The Buchwald-Hartwig cross coupling with 4-Amino-N-(tert-butyl)benzenesulfonamide was performed according to general procedure B.

The compound was cleaved of the resin by incubating at room temperature for 1 hour with a solution of TFA:H_2O:TIPS (92.5:5:2.5) and washed with TFA:H_2O:TIPS (92.5:5:2.5). The volume was reduced by evaporating the TFA solution with a N_2 stream. The crude mixture was purified by reverse phase column chromatography (C18, 00-35% MQ:MeCN) and product x was isolated in 5% yield (5.7. mg, 5.1 µmol, >95% purity). MS (ESI): m/z calculated for $C_{52}H_{72}N_{14}O_{10}S_2$ [M+H] = 1117.5075, found = 1117.60

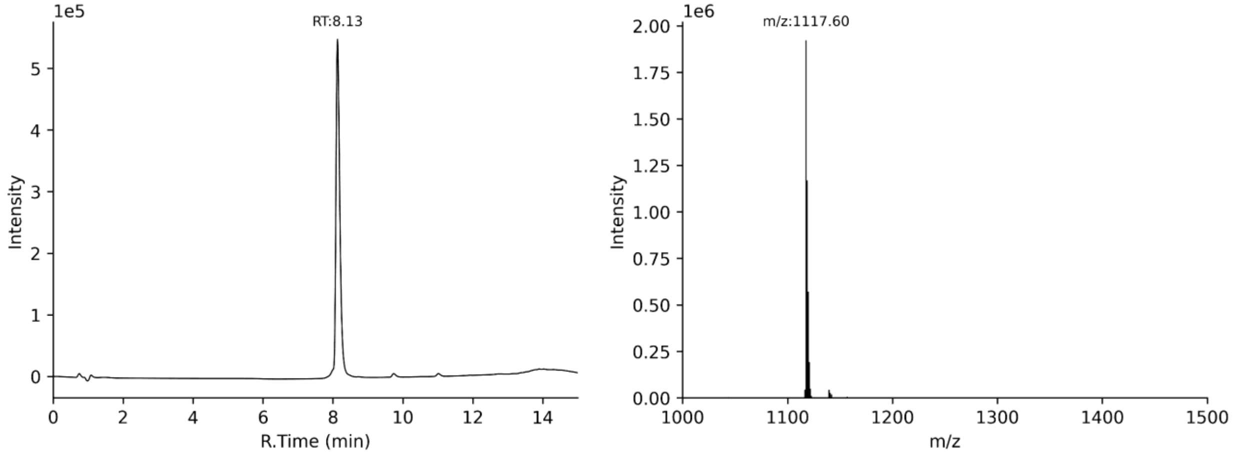

Fig. S23. LC-MS chromatogram from purified compound 18.

7.5 Hit validation

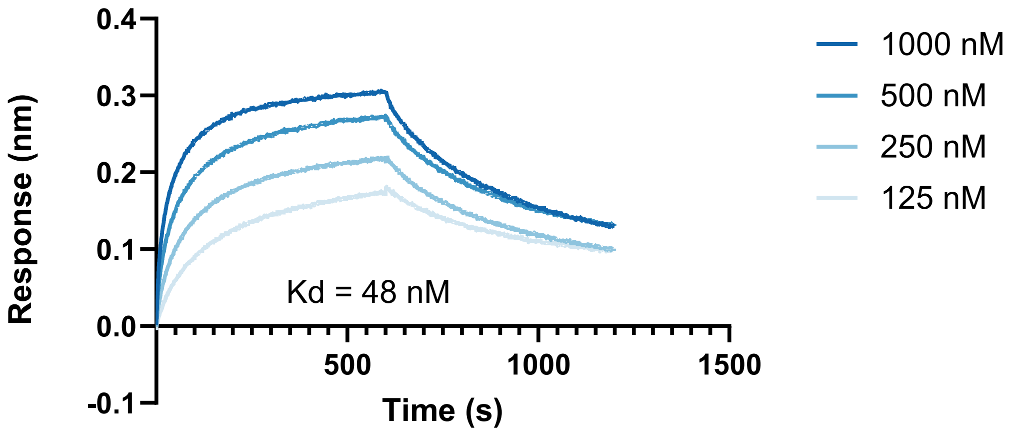

Fig. S24. Biolayer interferometry association and dissociation curves for compound 11.
The biolayer interferometry experiment was performed according to the general procedure describe in section 3.3

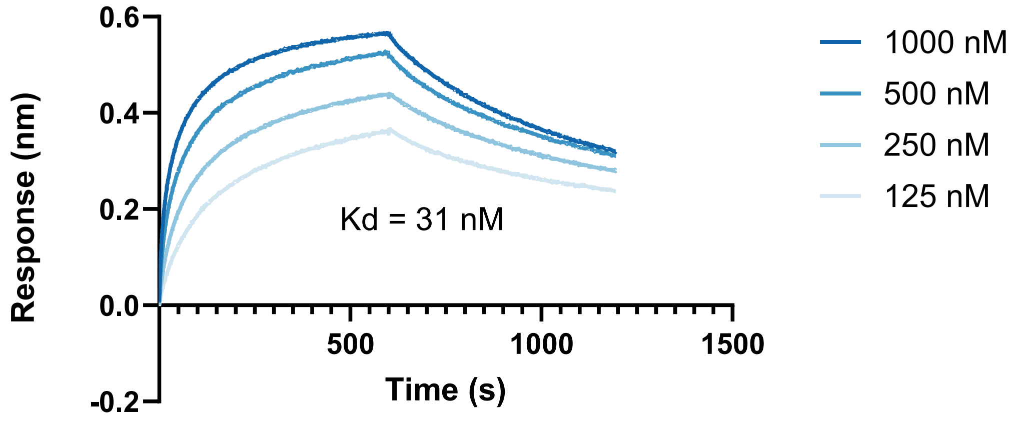

Fig. S25. Biolayer interferometry association and dissociation curves for compound 12.
The biolayer interferometry experiment was performed according to the general procedure describe in section 3.3

8. List of building blocks

Table S3. Overview of amino acids used in SEL

Amino acid (AA)	CAS	SMILES
AA1	852288-18-7	O=C(O)[C@@H](NC(OCC1c2c(c3c1cccc3)cccc2)=O)Cc4cc5c(OC(C)(C)O5)cc4
AA2	1313054-52-2	O=C(N[C@@H](C(O)=O)CCC(NC(c1c2cccc1)c3c(O2)cccc3)=O)OCC4c5c(c6c4cccc6)cccc5
AA3	215190-27-5	C1CN(CCC12C(=O)N(CN2c3ccccc3)CC(=O)O)C(=O)OCC4c5ccccc5c6ccccc46
AA4	885951-77-9	C1CC(C1)(C(=O)O)NC(=O)OCC2c3ccccc3c4ccccc24
AA5	368866-30-2	C1CN(CCC1(C(=O)O)NC(=O)OCC2c3ccccc3c4ccccc24)CC5=CC=CC=C5
AA6	185379-39-9	C3(c1ccccc1c2ccccc23)COC(=O)N[C@H](Cc4ccccn4)C(=O)O
AA7	270063-55-3	C3(c1ccccc1c2ccccc23)COC(=O)N[C@@H](Cc4cc(c(cc4)F)F)CC(=O)O
AA8	198560-38-2	C1C(N(c2ccccc21)C(=O)OCC3c4ccccc4c5ccccc35)C(=O)O
AA9	372159-75-6	c1ccc2cc(c(cc2c1)C(=O)O)NC(=O)OCC3c4ccccc4c5ccccc35
AA10	1986905-26-3	C1CC(CCC1(C(=O)O)NC(=O)OCC2c3ccccc3c4ccccc24)(F)F
AA11	204318-02-5	OC(=O)[C@@H]1CCCN1C1CCN(CC1)C(=O)OCC1c2ccccc2c3ccccc13
AA12	117322-30-2	C1CCC(C1)(C(=O)O)NC(=O)OCC2c3ccccc3c4ccccc24
AA13	1335206-44-4	C1C[C@@H](N(C1)C(=O)OCC2c3ccccc3c4ccccc24)COCC(=O)O
AA14	84000-07-7	C[C@@H](C(=O)O)N(C)C(=O)OCC1c2ccccc2c3ccccc13
AA15	133054-21-4	C1[C@H](N(CS1)C(=O)OCC2c3ccccc3c4ccccc24)C(=O)O
AA16	193885-59-5	CCCCCCCC[C@@H](C(=O)O)NC(=O)OCC1c2ccccc2c3ccccc13
AA17	252049-14-2	CC(C)(C)c1ccc(cc1)C[C@H](C(=O)O)NC(=O)OCC2c3ccccc3c4ccccc24
AA18	123106-21-8	C3(c1ccccc1c2ccccc23)COC(=O)NOCC(=O)O
AA19	180576-05-0	C1CN(CCN1CC(=O)O)C(=O)OCC2c3ccccc3c4ccccc24
AA20	1380327-56-9	C1C(CO1)(C(=O)O)NC(=O)OCC2c3ccccc3c4ccccc24
AA21	193693-64-0	C1C(CN1C(=O)OCC2c3ccccc3c4ccccc24)C(=O)O
AA22	203866-19-7	C1[C@@H](CN([C@@H]1C(=O)O)C(=O)OCC2c3ccccc3c4ccccc24)F
AA23	185116-43-2	C3(c1ccccc1c2ccccc23)COC(=O)NC4=CC=C(C=C4)C(=O)O
AA24	203866-21-1	C1[C@H](N(CC1(F)F)C(=O)OCC2c3ccccc3c4ccccc24)C(=O)O
AA25	159766-56-0	CC(=O)NCCCC[C@@H](C(=O)O)NC(=O)OCC1c2ccccc2c3ccccc13
AA26	220497-85-8	C3(c1ccccc1c2ccccc23)COC(=O)N[C@H](CC4=CC=CO4)C(=O)O
AA27	84891-19-0	c1ccc(cc1)COCn2cncc2C[C@@H](C(=O)O)NC(=O)OCC3c4ccccc4c5ccccc35
AA28	155369-11-2	C3(c1ccccc1c2ccccc23)COC(=O)NCC4=CC(=CC=C4)C(=O)O
AA29	371770-32-0	C1CCC(C1)C[C@@H](C(=O)O)NC(=O)OCC2c3ccccc3c4ccccc24
AA30	135673-97-1	C1CCC(CC1)C[C@@H](C(=O)O)NC(=O)OCC2c3ccccc3c4ccccc24
AA31	1197020-22-6	CCCCC[C@@H](C(=O)O)NC(=O)OCC1c2ccccc2c3ccccc13
AA32	478183-62-9	C1CC(C1)C[C@@H](C(=O)O)NC(=O)OCC2c3ccccc3c4ccccc24

AA33	112883-43-9	c1ccc2cc(ccc2c1)C[C@@H](C(=O)O)NC(=O)OCC3c4ccccc4c5ccccc35
AA34	29022-11-5	OC(CNC(OCC1C(C=CC=C2)=C2C3=C1C=CC=C3)=O)=O
AA35	35661-39-3	OC([C@H](C)NC(OCC1C(C=CC=C2)=C2C3=C1C=CC=C3)=O)=O
AA36	71989-37-2	OC([C@H](C(C)C)NC(OCC1C(C=CC=C2)=C2C3=C1C=CC=C3)=O)=O
AA37	35661-60-0	OC([C@H](CC(C)C)NC(OCC1C(C=CC=C2)=C2C3=C1C=CC=C3)=O)=O
AA38	35661-40-6	OC([C@H](CC1=CC=CC=C1)NC(OCC2C(C=CC=C3)=C3C4=C2C=CC=C4)=O)=O
AA39	71989-36-1	OC([C@H](CC1=CC=C(OC(C)(C)C)C=C1)NC(OCC2C(C=CC=C3)=C3C4=C2C=CC=C4)=O)=O
AA40	143432-06-0	OC([C@H](CC1=CC(C=CC=C2)=C2N1C(OC(C)(C)C)=O)NC(OCC3C(C=CC=C4)=C4C5=C3C=CC=C5)=O)=O
AA41	71989-14-5	OC([C@H](CC(OC(C)(C)C)=O)NC(OCC1C(C=CC=C2)=C2C3=C1C=CC=C3)=O)=O
AA42	132388-59-1	OC([C@H](CC(NC(C1=CC=CC=C1)(C2=CC=CC=C2)C3=CC=CC=C3)=O)NC(OCC4C(C=CC=C5)=C5C6=C4C=CC=C6)=O)=O
AA43	71989-18-9	OC([C@H](CCC(OC(C)(C)C)=O)NC(OCC1C(C=CC=C2)=C2C3=C1C=CC=C3)=O)=O
AA44	132327-80-1	OC([C@H](CCC(NC(C1=CC=CC=C1)(C2=CC=CC=C2)C3=CC=CC=C3)=O)NC(OCC4C(C=CC=C5)=C5C6=C4C=CC=C6)=O)=O
AA45	71989-27-0	OC([C@H](CCCCNC(OC(C)(C)C)=O)NC(OCC1C(C=CC=C2)=C2C3=C1C=CC=C3)=O)=O
AA46	154445-77-9	OC([C@H](CCCNC(NS(=O)(C1=C(C)C(C)=C(OC(C)(C)C2)C2=C1C)=O)=N)NC(OCC3C(C=CC=C4)=C4C5=C3C=CC=C5)=O)=O
AA47	114356-91-7	OC([C@H](CC1=CN(C(C2=CC=CC=C2)(C3=CC=CC=C3)C4=CC=CC=C4)C=N1)NC(OCC5C(C=CC=C6)=C6C7=C5C=CC=C7)=O)=O
AA48	71989-33-8	OC([C@H](COC(C)(C)C)NC(OCC1C(C=CC=C2)=C2C3=C1C=CC=C3)=O)=O
AA49	71989-34-9	OC([C@H]([C@@H](C)OC(C)(C)C)NC(OCC1C(C=CC=C2)=C2C3=C1C=CC=C3)=O)=O

Table S4. Overview of aryl bromides used in SEL

Aryl bromide (AB)	CAS	SMILES
AB1	1878-68-8	OC(CC1=CC=C(C=C1)Br)=O
AB2	33282-23-4	BrC1=CC=C(C2=CC(C(O)=O)=NO2)C=C1
AB3	345965-52-8	BrC1=CC=C(C2(CC2)C(O)=O)C=C1
AB4	198561-04-5, 65-85-0	OC([C@H](CC1=CC=C(Br)C=C1)NC(C2=CC=CC=C2)=O)=O
AB5	198561-04-5, 5292-21-7	O=C(CC1CCCCC1)N[C@@H](CC2=CC=C(Br)C=C2)C(O)=O
AB6	198561-04-5, 1188-02-9	O=C(O)[C@@H](NC(C(CCCCC)C)=O)CC(C=C1)=CC=C1Br

AB7	198561-04-5, 581-96-4	O=C(CC1=CC(C=CC=C2)=C2C=C1)N[C@@H](CC3=CC=C(Br)C=C3)C(O)=O

Table S5. Overview of amines used in SEL

Amine (AM)	CAS	SMILES
AM1	62-53-3	NC1=CC=CC=C1
AM2	71026-66-9	NC1=CC=C(NC(OC(C)(C)C)=O)C=C1
AM3	591-27-5	NC1=CC=CC(O)=C1
AM4	348-54-9	NC1=C(F)C=CC=C1
AM5	372-19-0	NC1=CC(F)=CC=C1
AM6	371-40-4	NC1=CC=C(F)C=C1
AM7	873-74-5	NC1=CC=C(C#N)C=C1
AM8	95-68-1	NC1=CC=C(C)C=C1C
AM9	87-62-7	NC1=C(C)C=CC=C1C
AM10	95-64-7	NC1=CC=C(C)C(C)=C1
AM11	589-16-2	NC1=CC=C(CC)C=C1
AM12	104-94-9	NC1=CC=C(OC)C=C1
AM13	90-04-0	NC1=C(OC)C=CC=C1
AM14	536-90-3	NC1=CC=CC(OC)=C1
AM15	452-80-2	NC1=C(F)C=C(C)C=C1
AM16	367-25-9	NC1=CC=C(F)C=C1F
AM17	50670-64-9	NC1=CC=C(C)C(C#N)=C1
AM18	60710-80-7	NC1=C(C)C=CC(C#N)=C1
AM19	551-93-9	NC1=C(C(C)=O)C=CC=C1
AM20	551-93-9	NC1=CC=CC(C(C)=O)=C1
AM21	99-03-6	NC1=C(C)C=C(C)C=C1C
AM22	53312-81-5	NC1=CC=C(F)C(C#N)=C1
AM23	2835-68-9	NC1=CC=C(C(N)=O)C=C1
AM24	14268-66-7	NC1=CC=C(OCO2)C2=C1
AM25	104-10-9	NC1=CC=C(CCO)C=C1
AM26	99-09-2	NC1=CC=CC([N+]([O-])=O)=C1
AM27	2987-53-3	NC1=CC=CC=C1SC
AM28	91-59-8	NC1=CC=C(C=CC=C2)C2=C1
AM29	134-32-7	NC1=C(C=CC=C2)C2=CC=C1
AM30	25900-61-2	NC1=CC(C(NC)=O)=CC=C1
AM31	4469-80-1	NC1=CC=C(OCCC)C=C1
AM32	455-14-1	NC1=CC=C(C(F)(F)F)C=C1
AM33	98-16-8	NC1=CC=CC(C(F)(F)F)=C1
AM34	90-41-5	NC1=C(C2=CC=CC=C2)C=CC=C1
AM35	39905-57-2	NC1=CC=C(OCCCCCC)C=C1

AM36	1134-94-7	NC1=CC=CC=C1SC2=CC=CC=C2
AM37	18144-47-3	NC1=CC=C(C(OC(C)(C)C)=O)C=C1
AM38	56765-79-8	NC1=CC(C#N)=C(C#N)C=C1
AM39	214623-57-1	NC1=CC(C#N)=C(OC)C=C1
AM40	60979-25-1	NC1=CC(C#N)=CC=C1OC
AM41	104-13-2	NC1=CC=C(CCCC)C=C1
AM42	134-20-3	NC1=CC=CC=C1C(OC)=O
AM43	09/10/4518	NC1=CC=CC(C(OC)=O)=C1
AM44	89-62-3	NC1=C([N+]([O-])=O)C=C(C)C=C1
AM45	87-25-2	NC1=CC=CC=C1C(OCC)=O
AM46	319-24-4	NC1=CC=C(F)C=C1C(OC)=O
AM47	24313-88-0	NC1=CC(OC)=C(OC)C(OC)=C1
AM48	209917-48-6	NC1=CC=C(S(NC(C)(C)C)(=O)=O)C=C1
AM49	504-29-0	NC1=CC=CC=N1
AM50	19842-07-0	NC1=CC=C(CC)C=N1
AM51	5049-61-6	NC1=CN=CC=N1
AM52	57260-71-6	CC(OC(N1CCNCC1)=O)(C)C
AM53	56227-55-5	O=C(C1=CC=CC=C1)N2CCNCC2
AM54	2252-63-3	FC(C=C1)=CC=C1N2CCNCC2
AM55	109-01-3	CN1CCNCC1
AM56	1008-91-9	N1(C2=CC=NC=C2)CCNCC1
AM57	27578-60-5	NCCN1CCCCC1
AM58	30459-17-7	FC(C(C=C1)=CC=C1N2CCNCC2)(F)F
AM59	62937-96-6	O=S(CC)(N1CCNCC1)=O
AM60	1011-15-0	FC1=CC=CC=C1N2CCNCC2
AM61	55276-43-2	CS(N1CCNCC1)(=O)=O
AM62	6269-89-2	O=[N+](C1=CC=C(N2CCNCC2)C=C1)[O-]
AM63	68104-63-2	N#CC1=CC=C(N2CCNCC2)C=C1
AM64	19984-46-4	N1(C2(CC(C3)C4)C[C@H]4C[C@H]3C2)CCNCC1
AM65	35386-24-4	COC1=CC=CC=C1N2CCNCC2
AM66	1013-76-9	CC1=CC(C)=CC=C1N2CCNCC2
AM67	115761-79-0	FC1=CC=C(N2CCNCC2)C(F)=C1
AM68	7154-73-6	NCCN1CCCC1
AM69	118708-88-6	FC(C1=CC(N2CCNCC2)=NC=C1)(F)F
AM70	92-54-6	C1(N2CCNCC2)=CC=CC=C1
AM71	80518-57-6	CCOC(C1=CC=C(N2CCNCC2)C=C1)=O
AM72	1158758-77-0	NC1(CN(C1)C(OC(C)(C)C)=O)C
AM73	217806-26-3	CC(OC(NC1CNC1)=O)(C)C
AM74	1758-46-9	C1=CC=C(C=C1)OCCN
AM75	2038-57-5	C1=CC=C(C=C1)CCCN

9. Supplementary LC-MS data

9.1 LC-MS data from the alkyl bromide scope

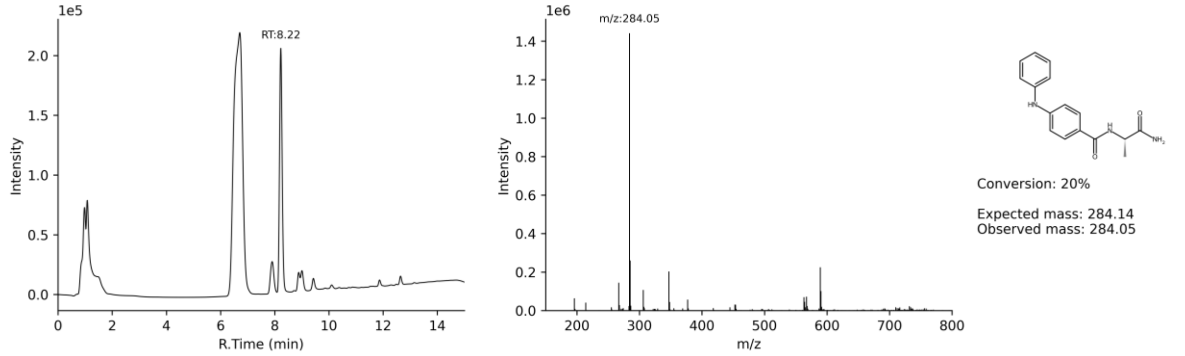

Fig. S26. LC-MS chromatogram of compound 5a.

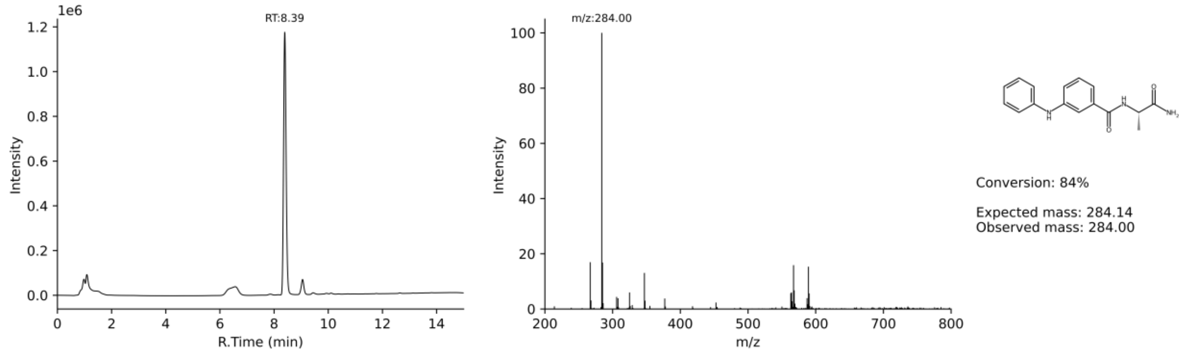

Fig. S27. LC-MS chromatogram of compound 5b.

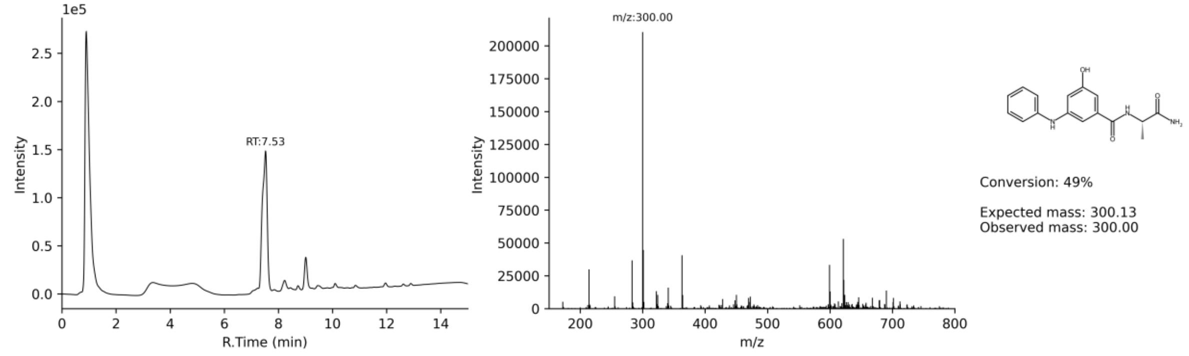

Fig. S28. LC-MS chromatogram of compound 5c.

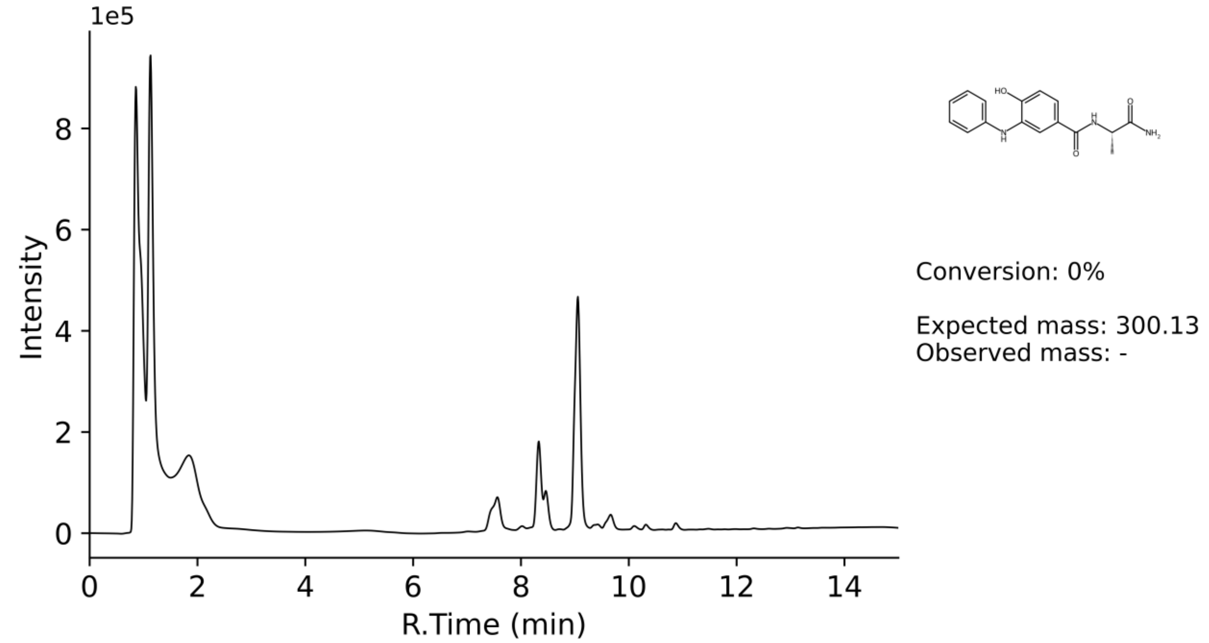

Fig. S29. LC-MS chromatogram of compound 5d.

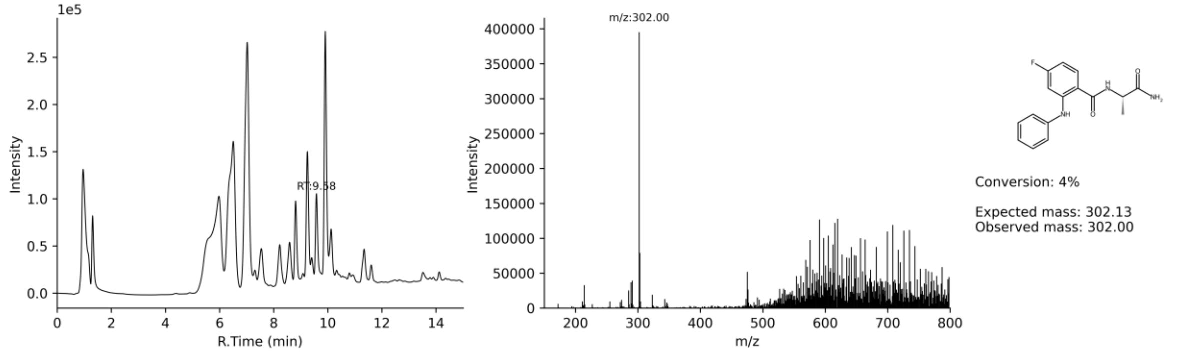

Fig. S30. LC-MS chromatogram of compound 5e.

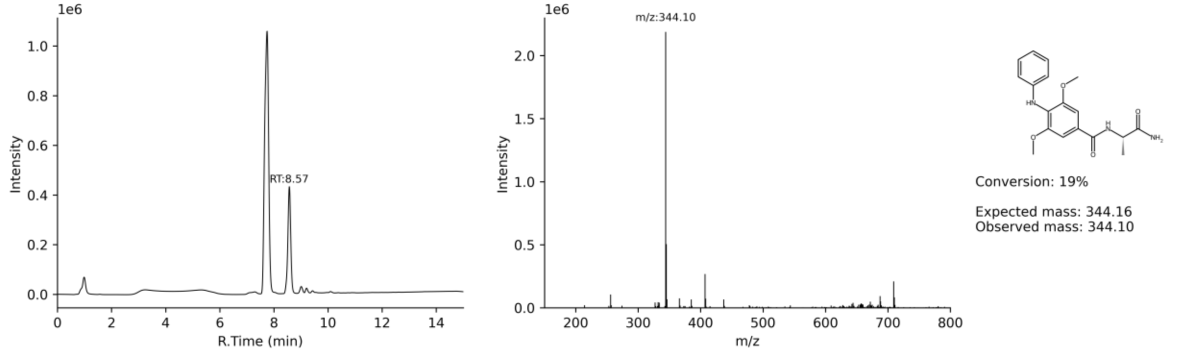

Fig. S31. LC-MS chromatogram of compound 5f.

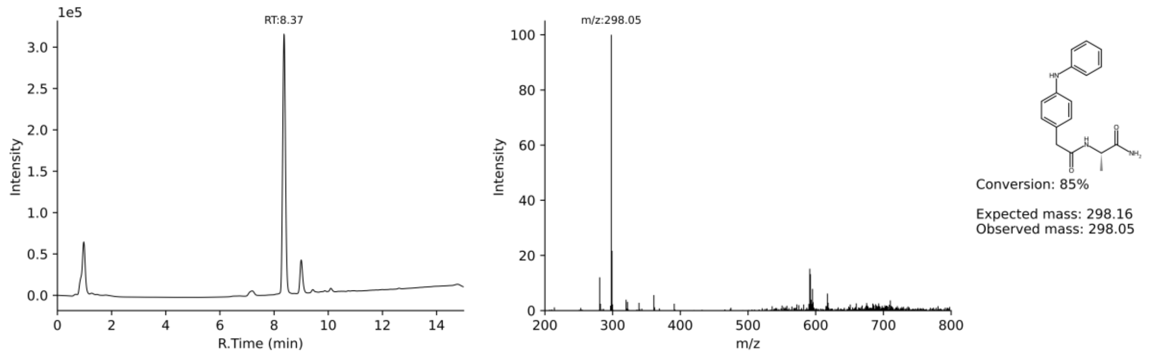

Fig. S32. LC-MS chromatogram of compound 5g.

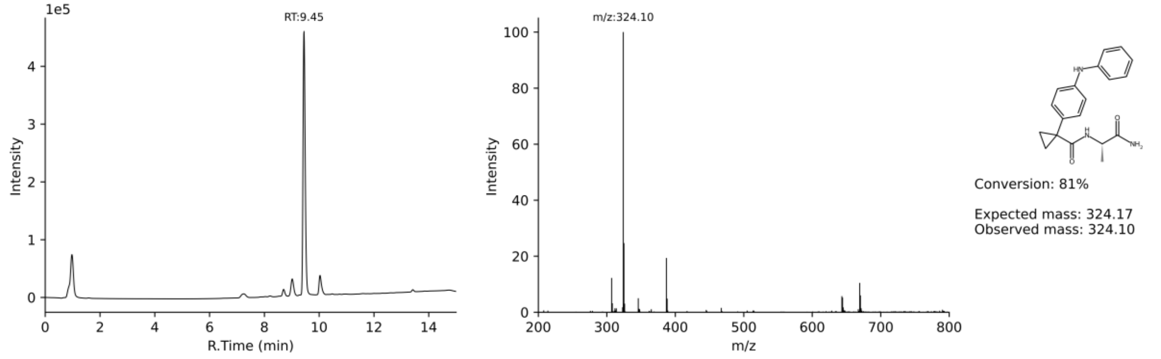

Fig. S33. LC-MS chromatogram of compound 5h.

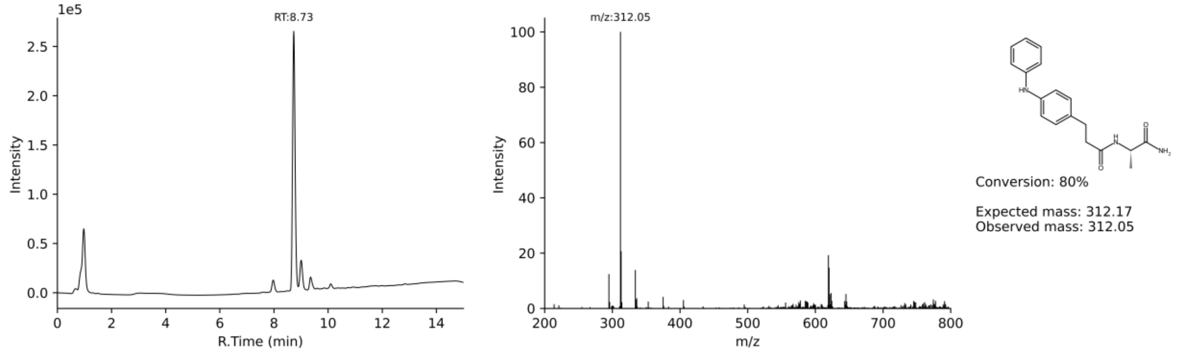

Fig. S34. LC-MS chromatogram of compound 5i.

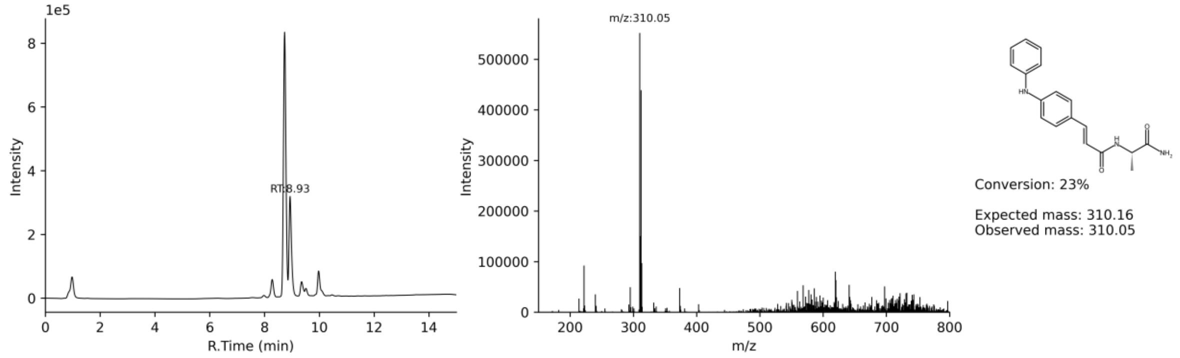

Fig. S35. LC-MS chromatogram of compound 5j.

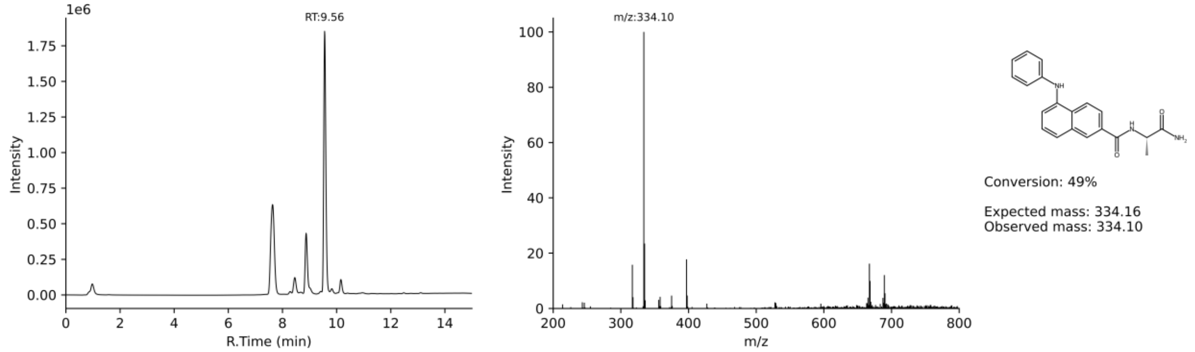

Fig. S36. LC-MS chromatogram of compound 5k.

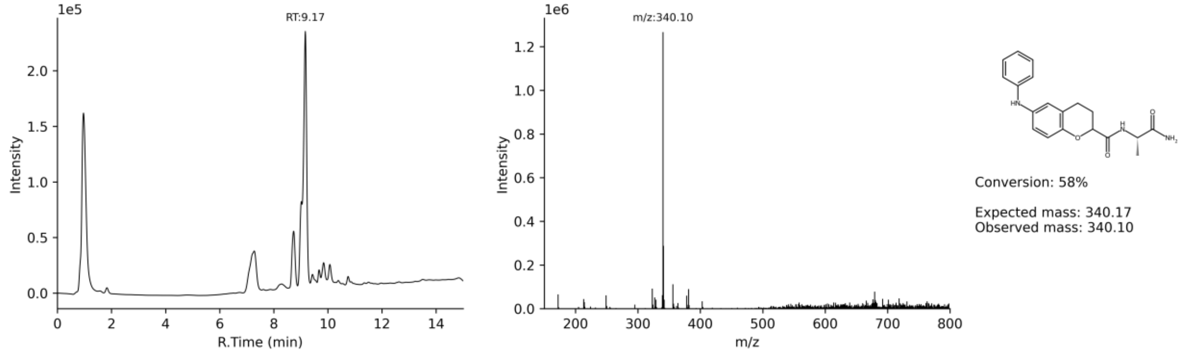

Fig. S37. LC-MS chromatogram of compound 5l.

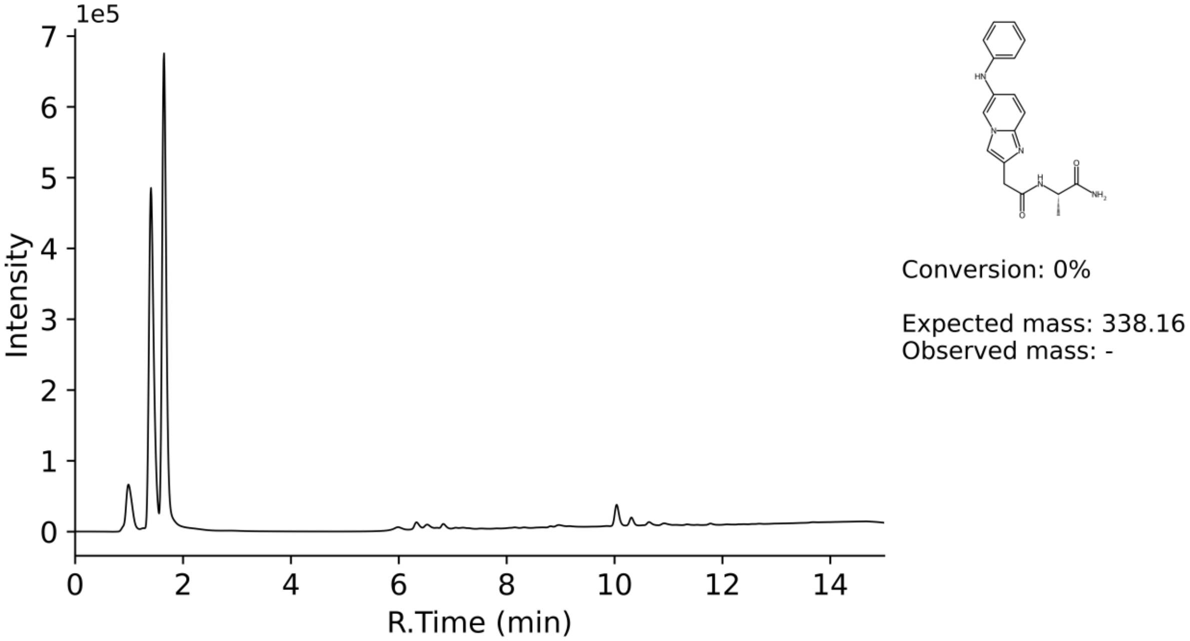

Fig. S38. LC-MS chromatogram of compound 5m.

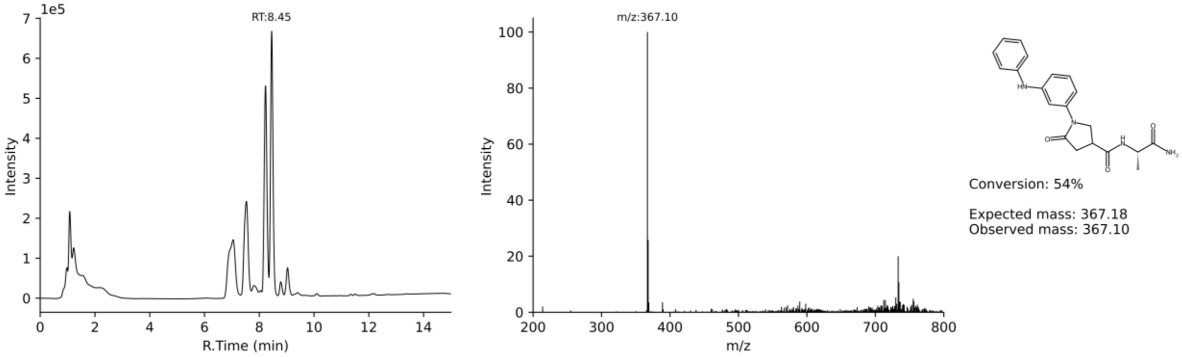

Fig. S39. LC-MS chromatogram of compound 5n.

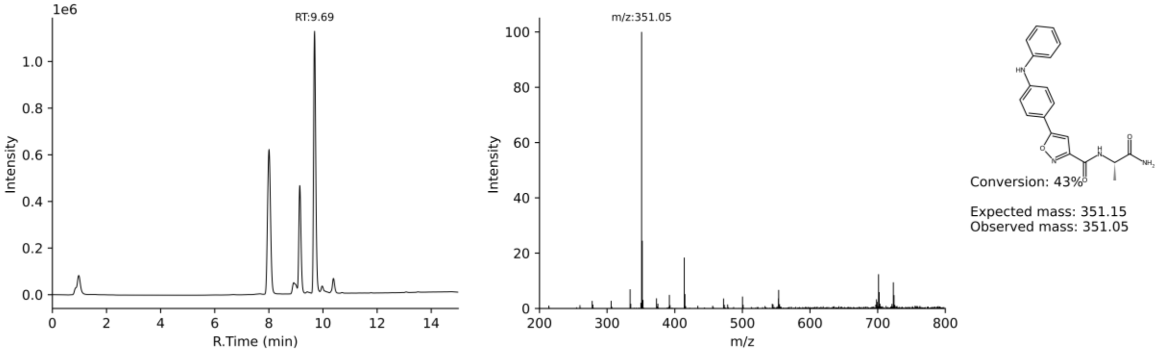

Fig. S40. LC-MS chromatogram of compound 5o.

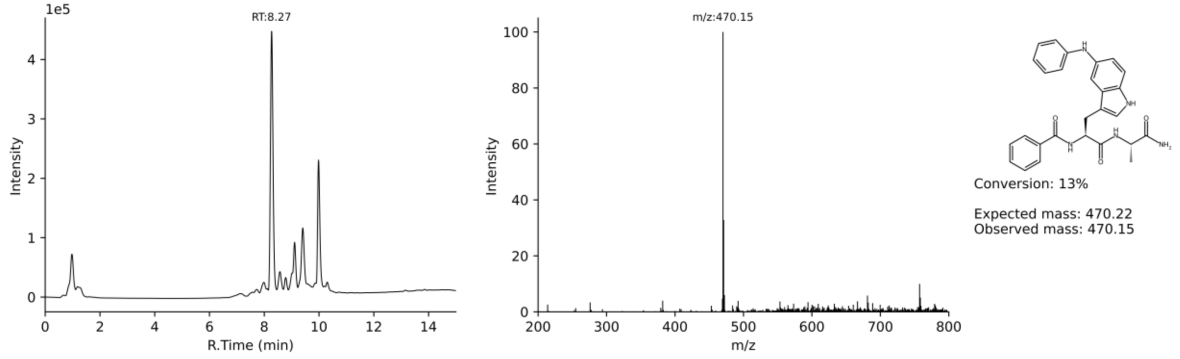

Fig. S41. LC-MS chromatogram of compound 5p.

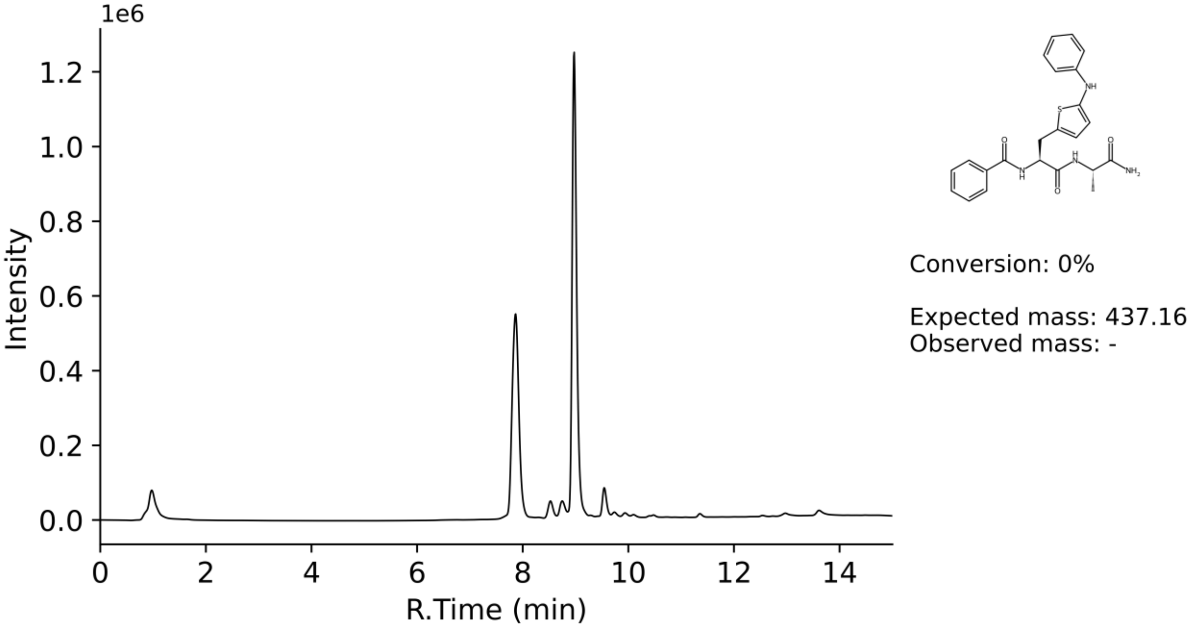

Fig. S42. LC-MS chromatogram of compound 5q.

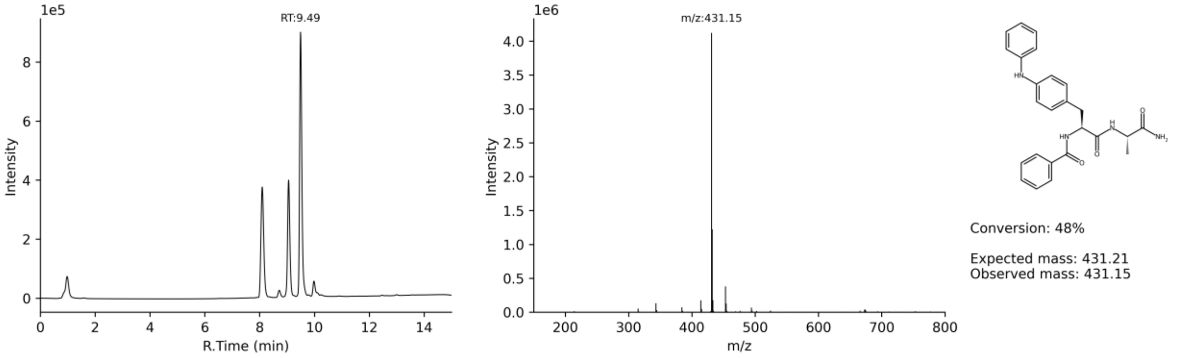

Fig. S43. LC-MS chromatogram of compound 5r.

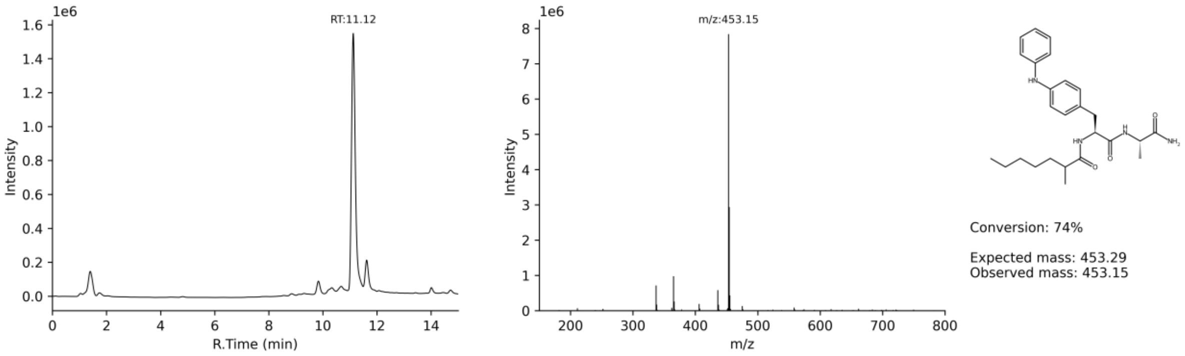

Fig. S44. LC-MS chromatogram of compound 5s.

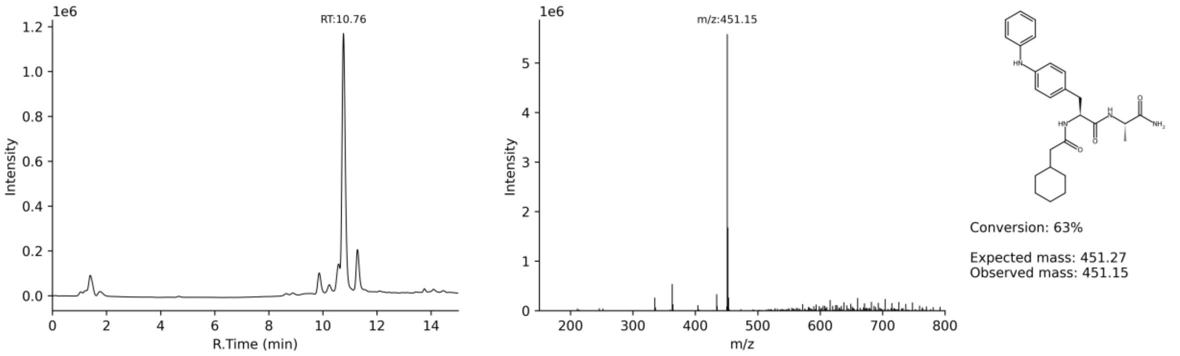

Fig. S45. LC-MS chromatogram of compound 5t.

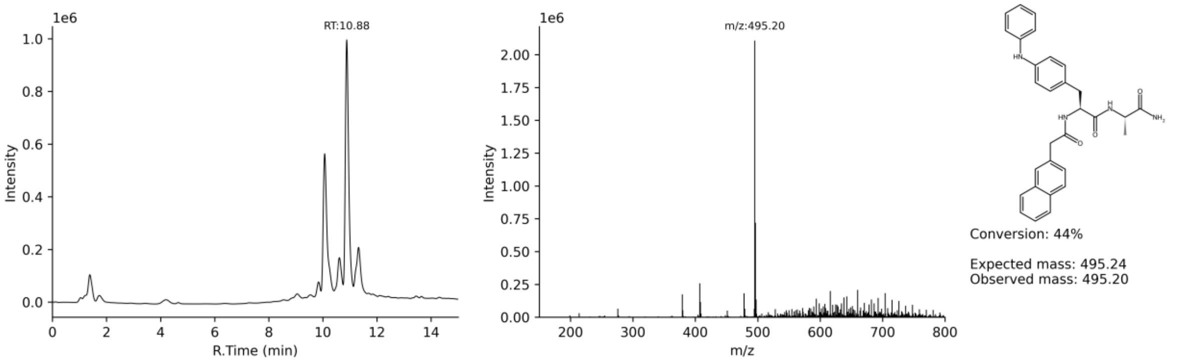

Fig. S46. LC-MS chromatogram of compound 5u.

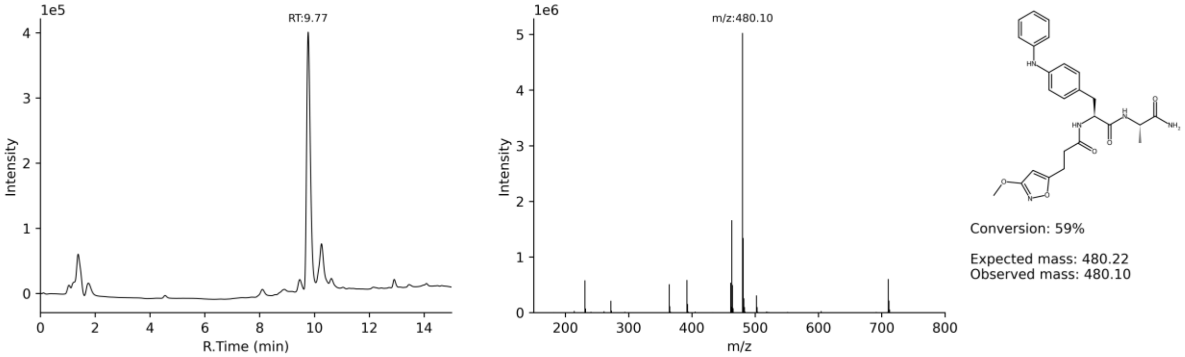

Fig. S47. LC-MS chromatogram of compound 5v.

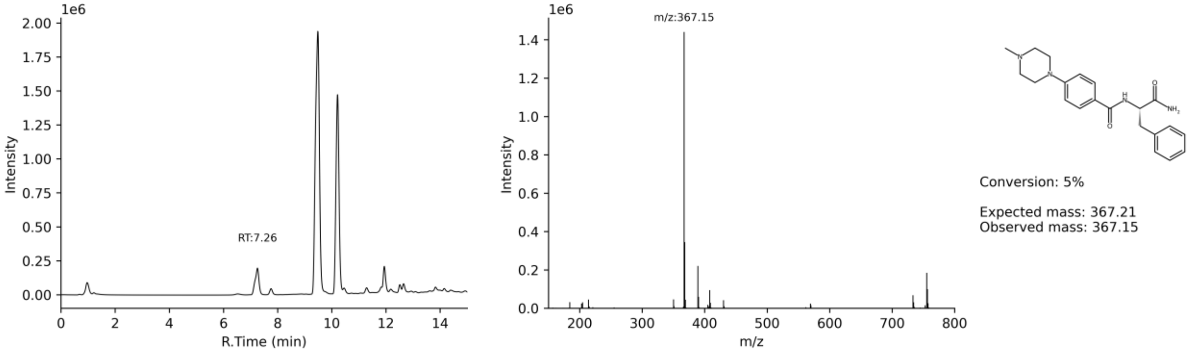

Fig. S48. LC-MS chromatogram of compound 6a.

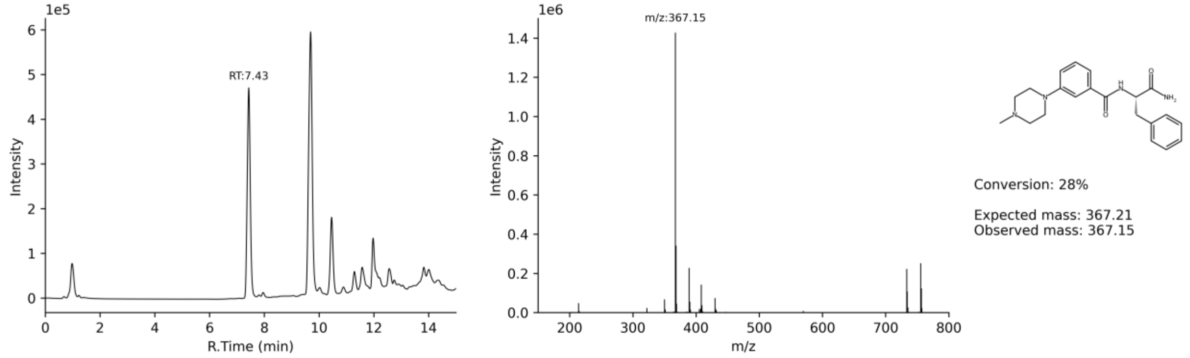

Fig. S49. LC-MS chromatogram of compound 6b.

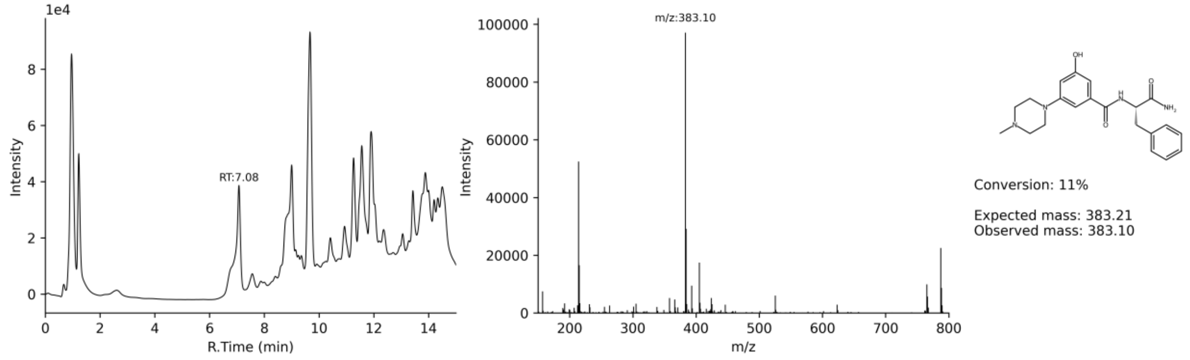

Fig. S50. LC-MS chromatogram of compound 6c.

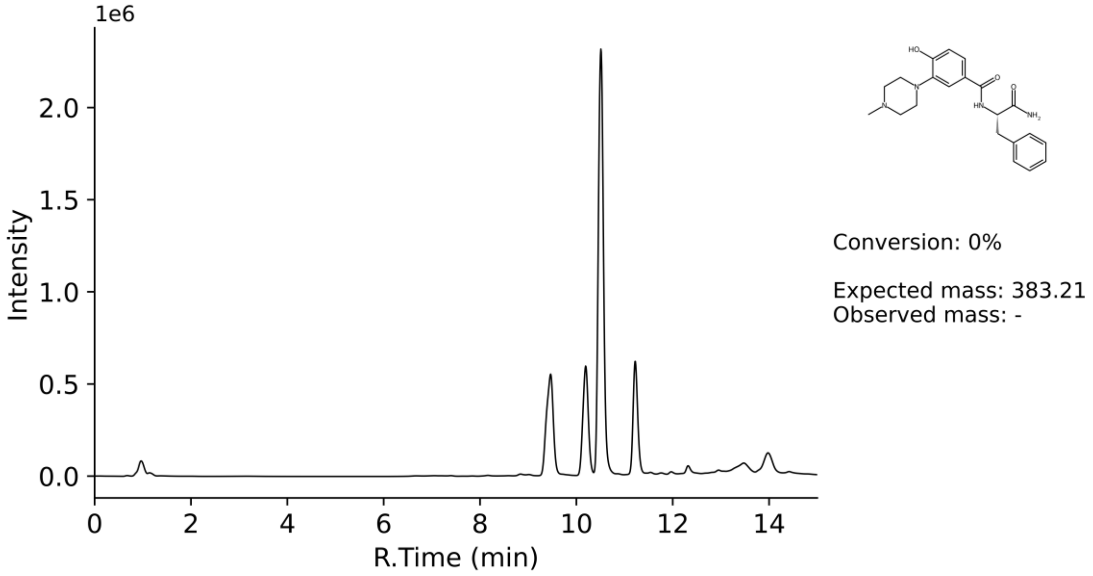

Fig. S51. LC-MS chromatogram of compound 6d.

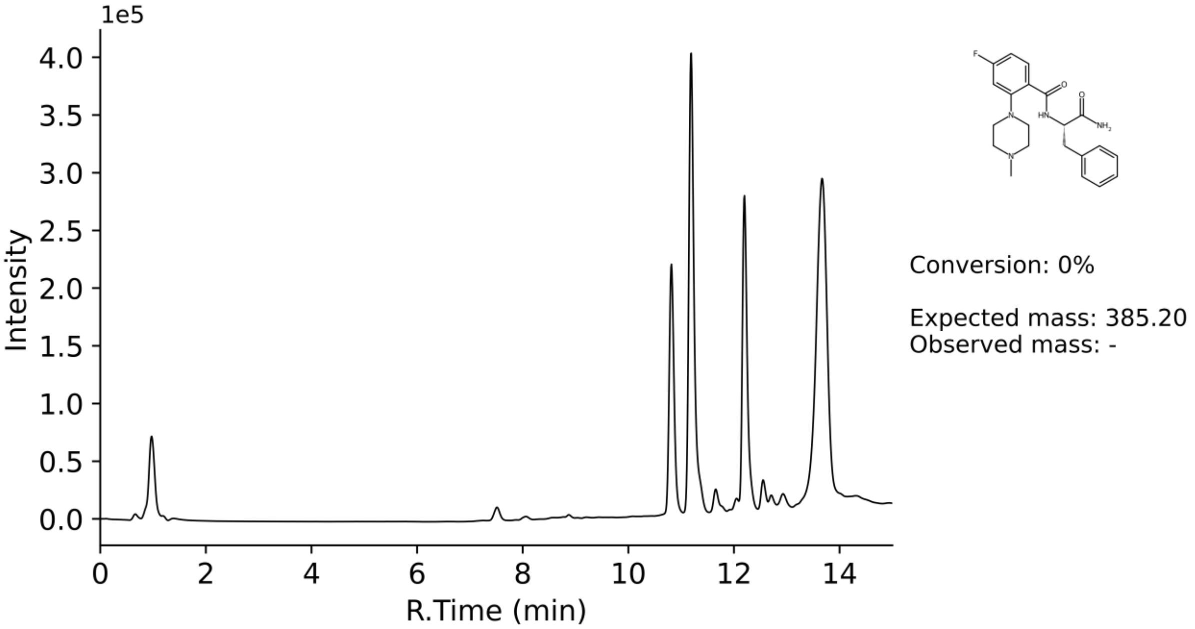

Fig. S52. LC-MS chromatogram of compound 6e.

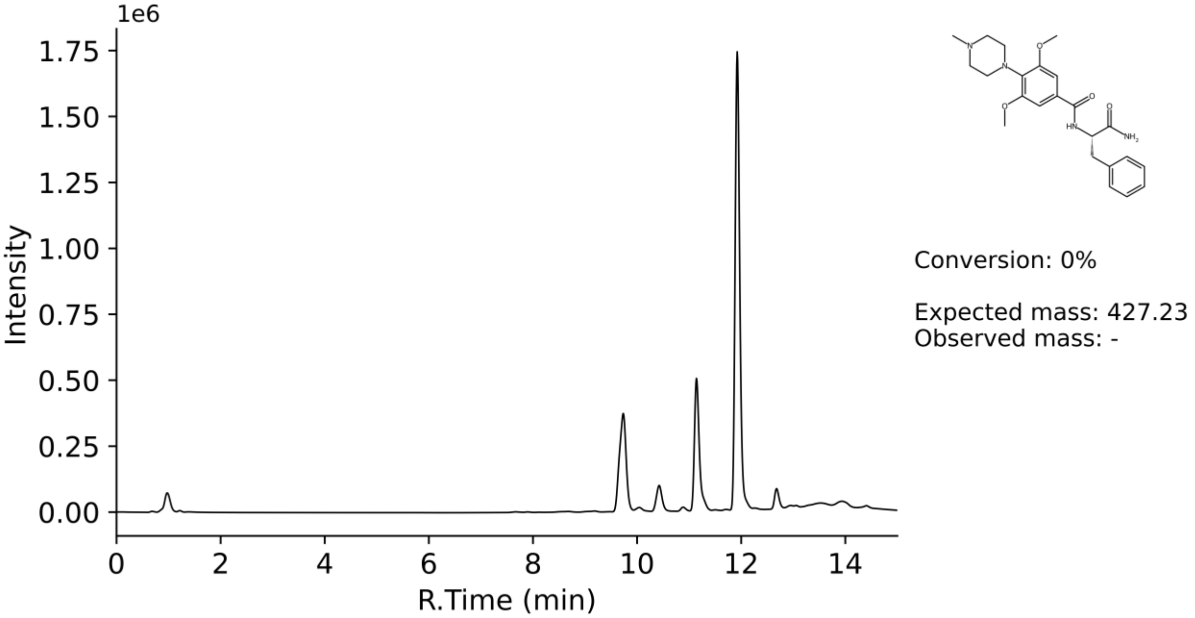

Fig. S53. LC-MS chromatogram of compound 6f.

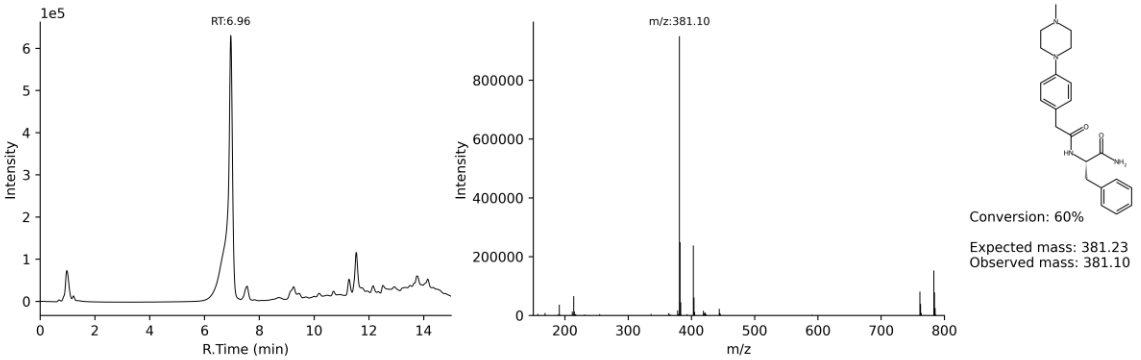

Fig. S54. LC-MS chromatogram of compound 6g.

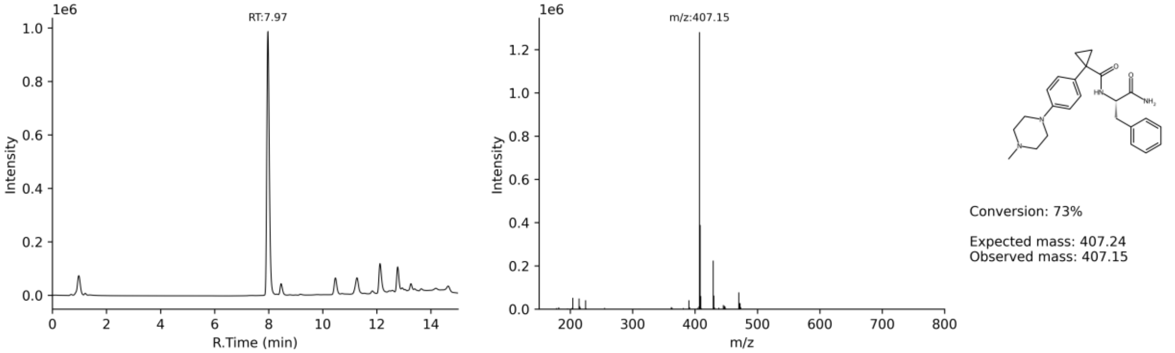

Fig. S55. LC-MS chromatogram of compound 6h.

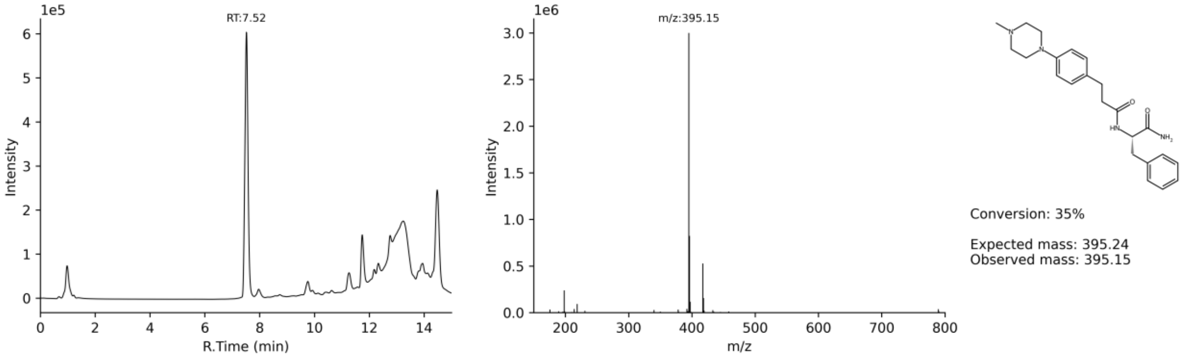

Fig. S56. LC-MS chromatogram of compound 6i.

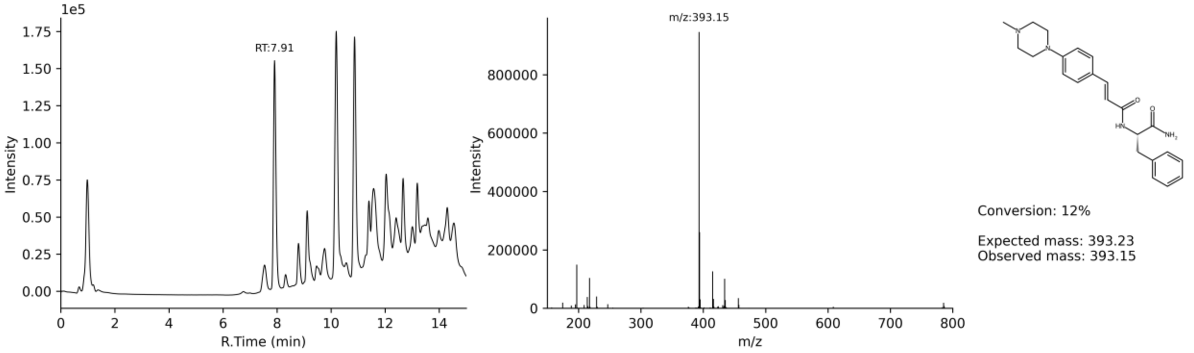

Fig. S57. LC-MS chromatogram of compound 6j.

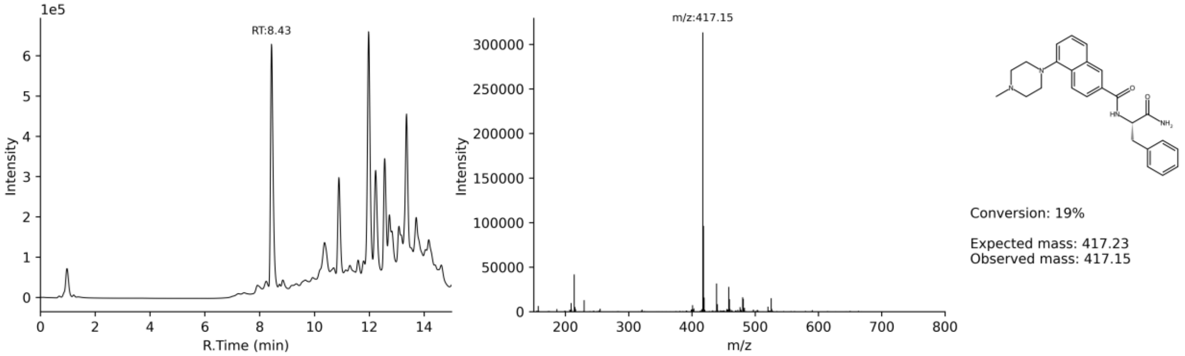

Fig. S58. LC-MS chromatogram of compound 6k.

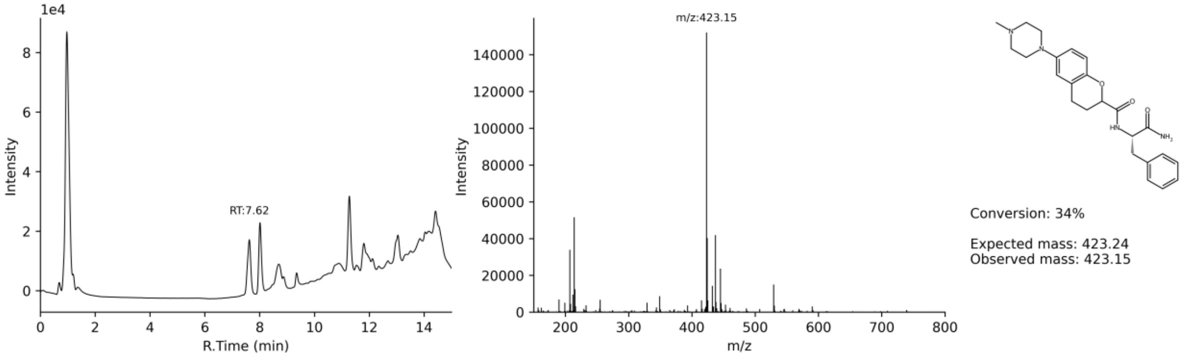

Fig. S59. LC-MS chromatogram of compound 6l.

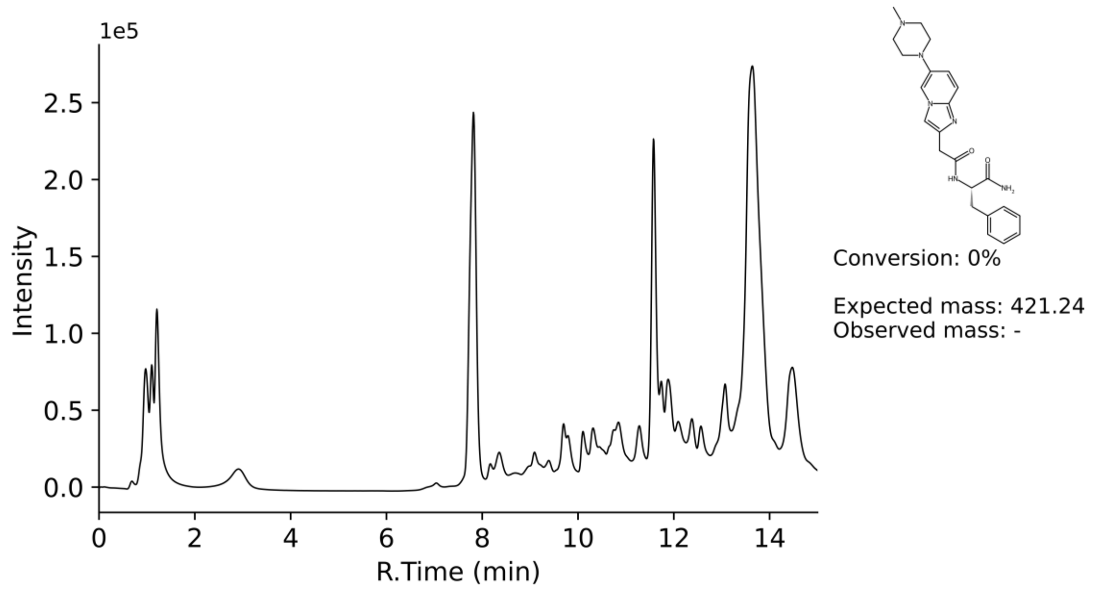

Fig. S60. LC-MS chromatogram of compound 6m.

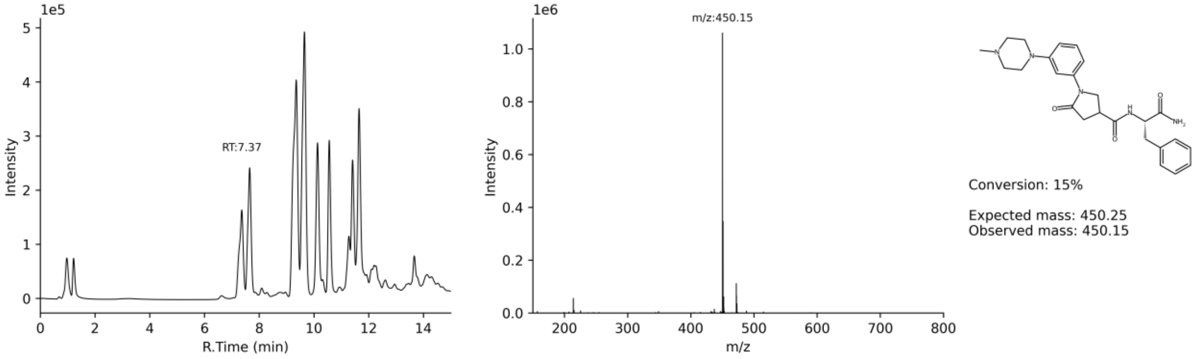

Fig. S61. LC-MS chromatogram of compound 6n.

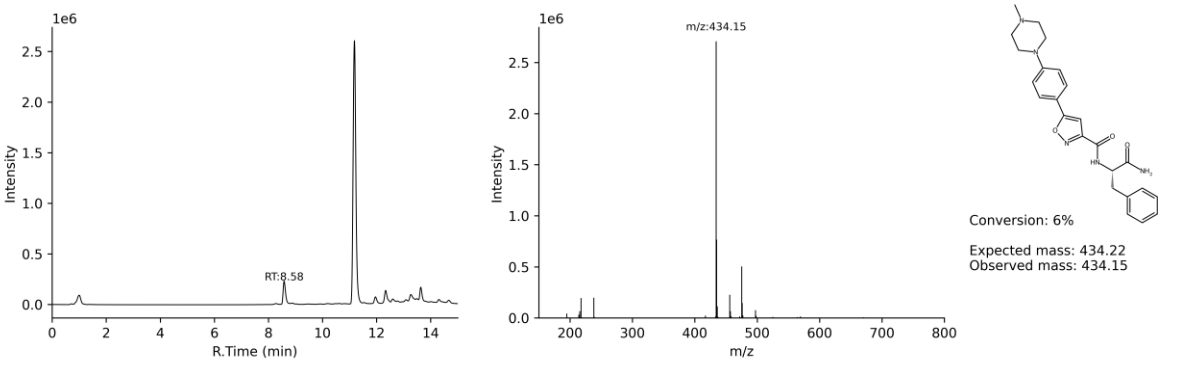

Fig. S62. LC-MS chromatogram of compound 6o.

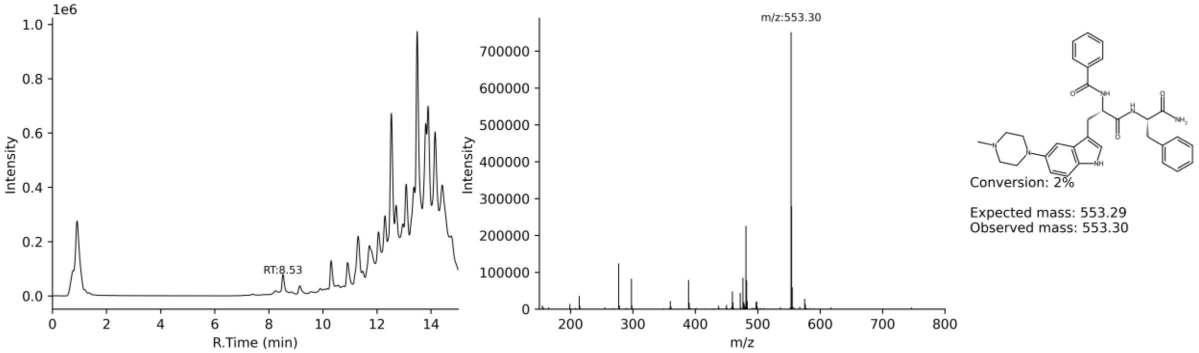

Fig. S63. LC-MS chromatogram of compound 6p.

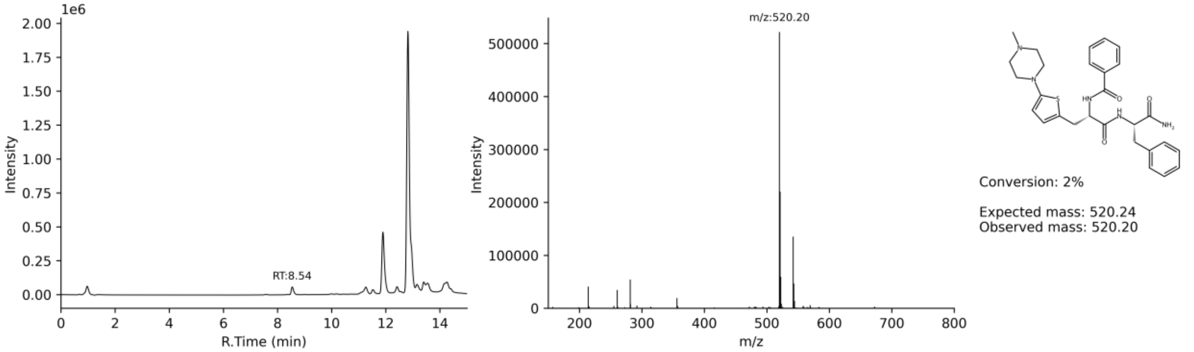

Fig. S64. LC-MS chromatogram of compound 6q.

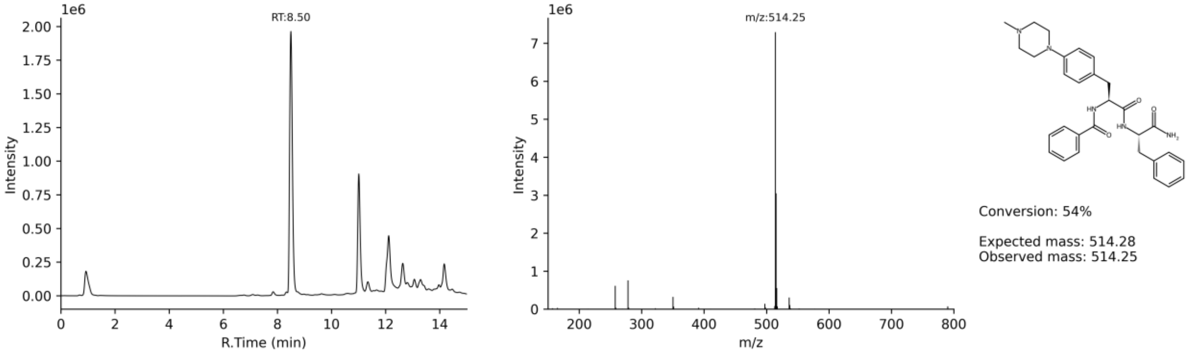

Fig. S65. LC-MS chromatogram of compound 6r.

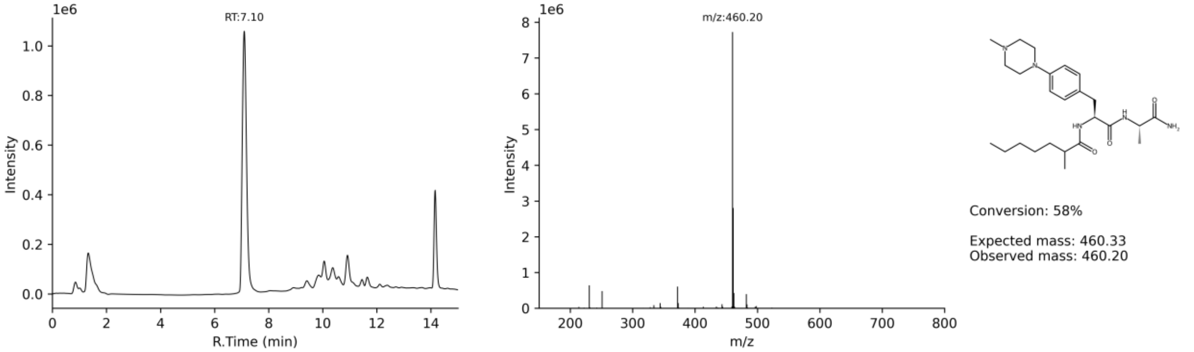

Fig. S66. LC-MS chromatogram of compound 6s.

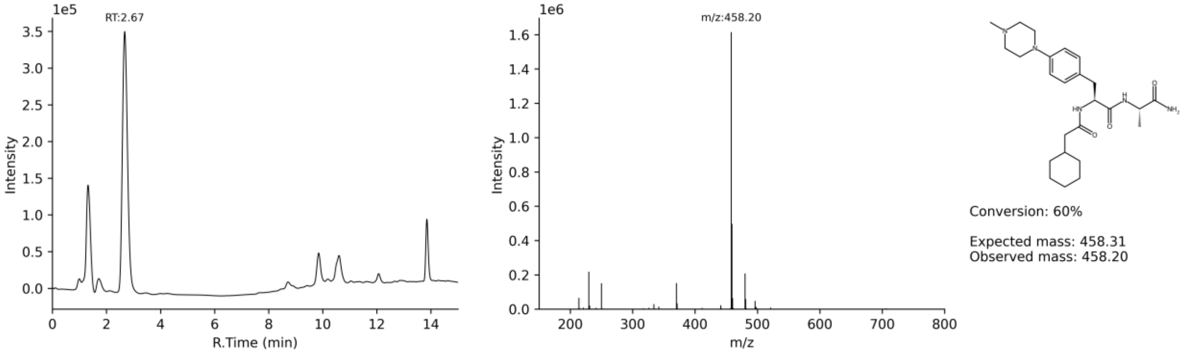

Fig. S67. LC-MS chromatogram of compound 6t.

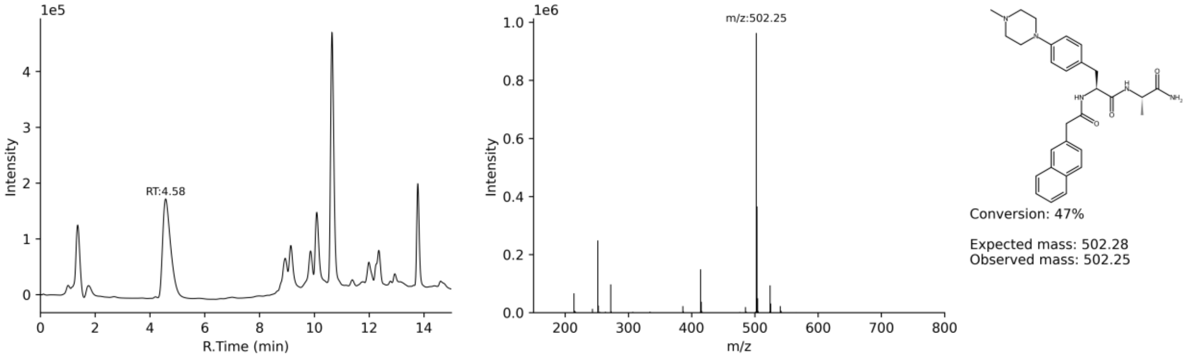

Fig. S68. LC-MS chromatogram of compound 6u.

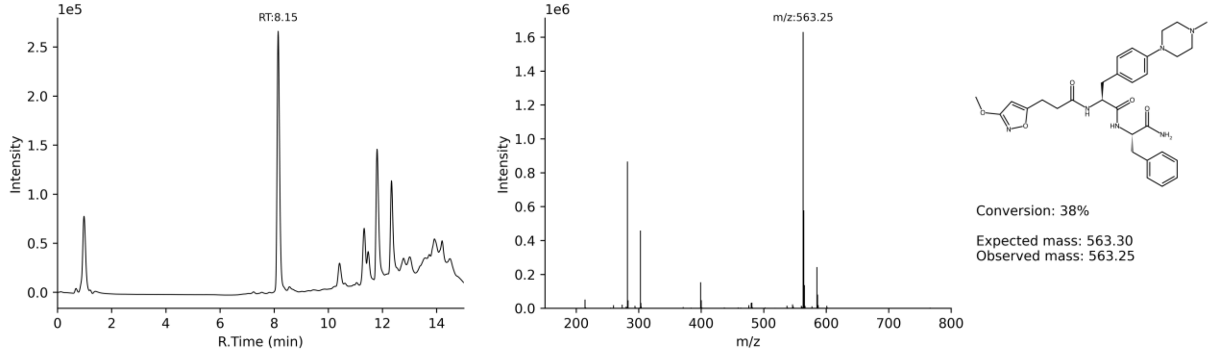

Fig. S69. LC-MS chromatogram of compound 6v.

9.2 LC-MS data from the amine scope

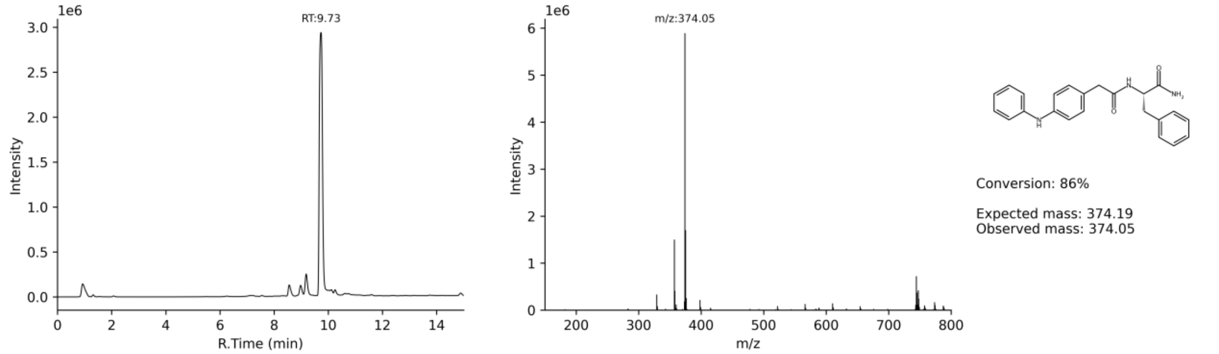

Fig. S70. LC-MS chromatogram of compound 7a.

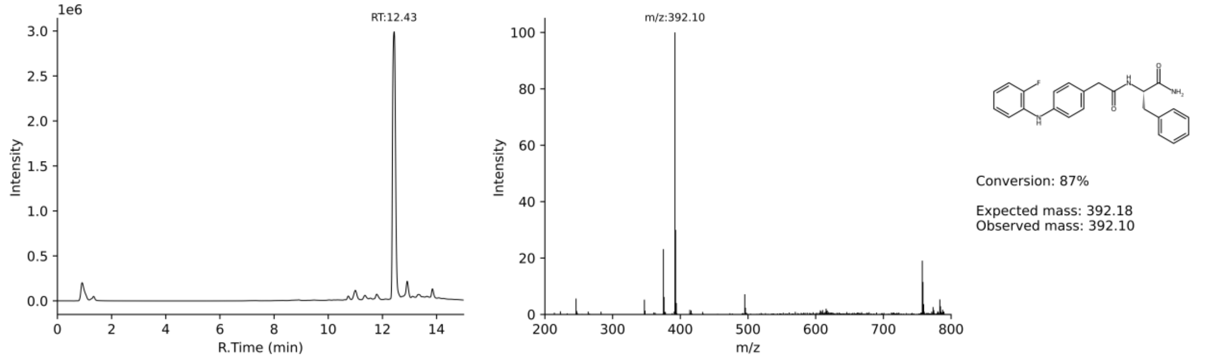

Fig. S71. LC-MS chromatogram of compound 7b.

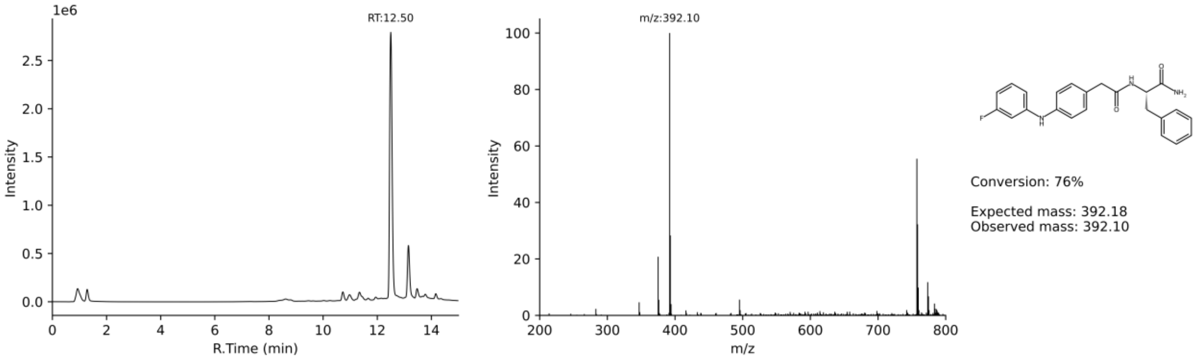

Fig. S72. LC-MS chromatogram of compound 7c.

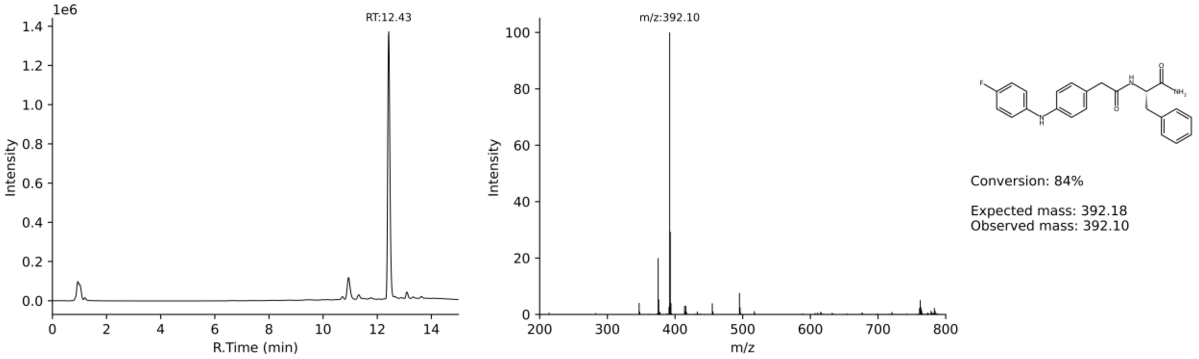

Fig. S73. LC-MS chromatogram of compound 7d.

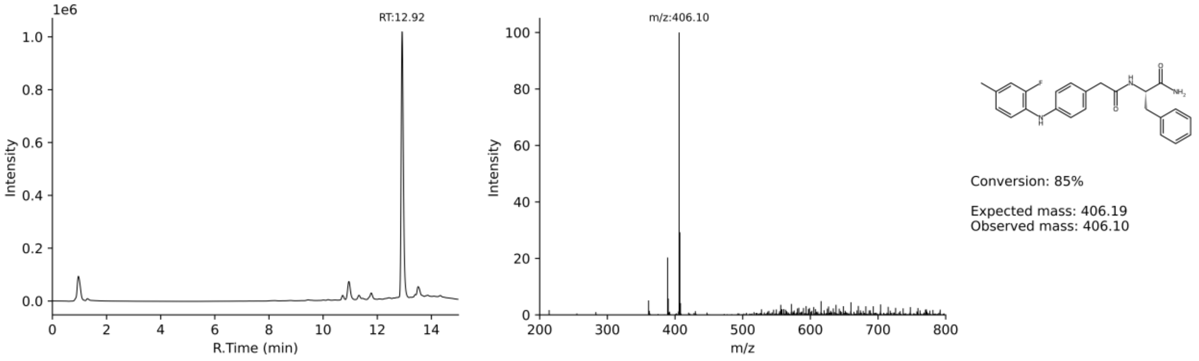

Fig. S74. LC-MS chromatogram of compound 7e.

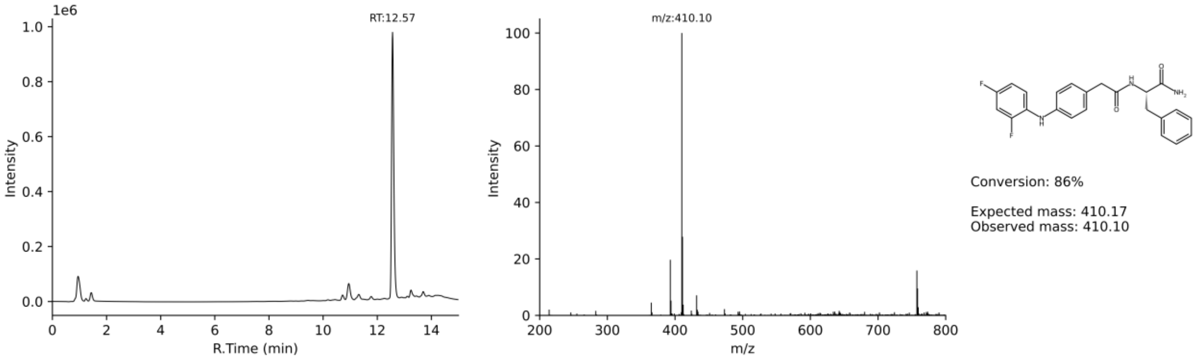

Fig. S75. LC-MS chromatogram of compound 7f.

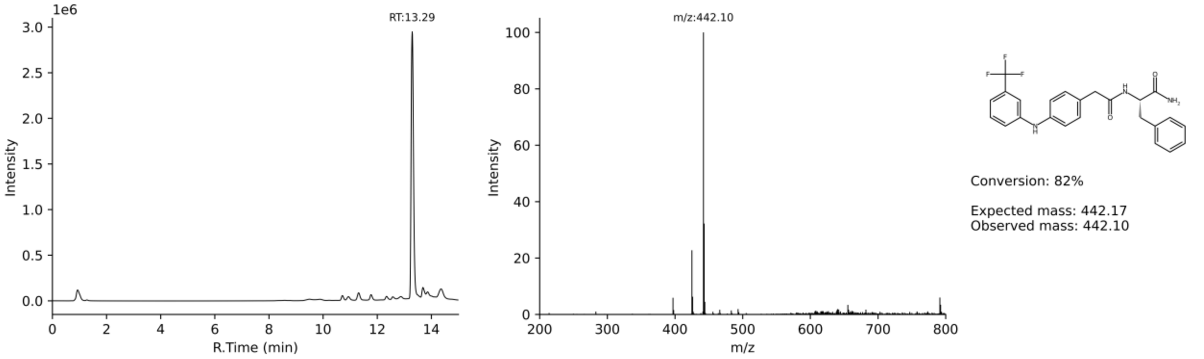

Fig. S76. LC-MS chromatogram of compound 7g.

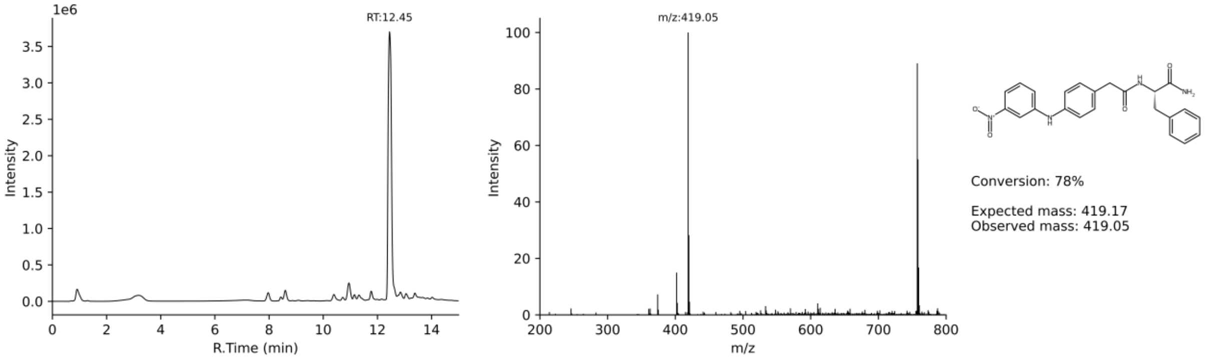

Fig. S77. LC-MS chromatogram of compound 7h.

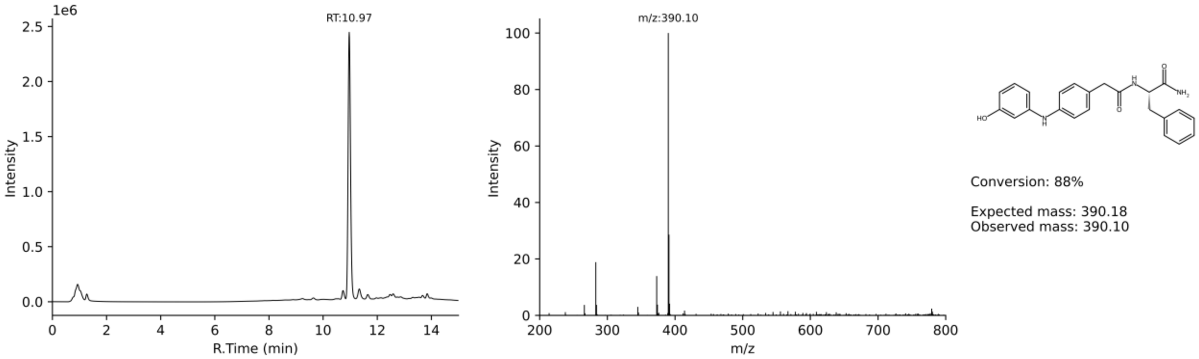

Fig. S78. LC-MS chromatogram of compound 7i.

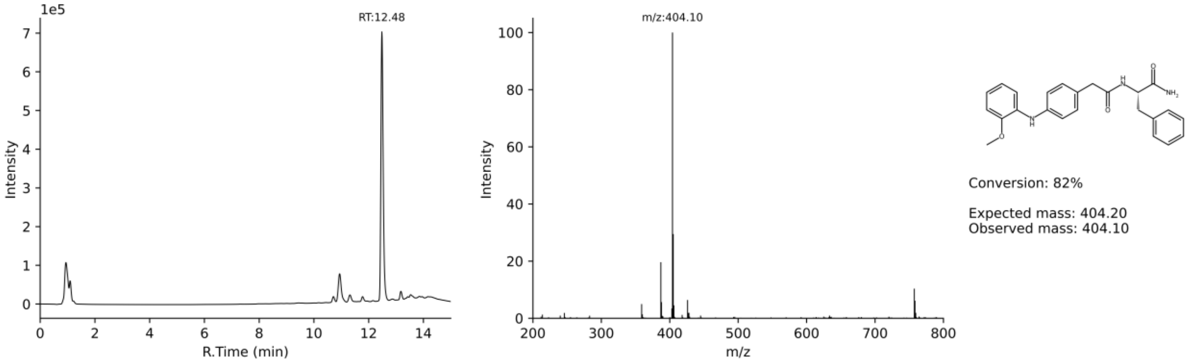

Fig. S79. LC-MS chromatogram of compound 7j.

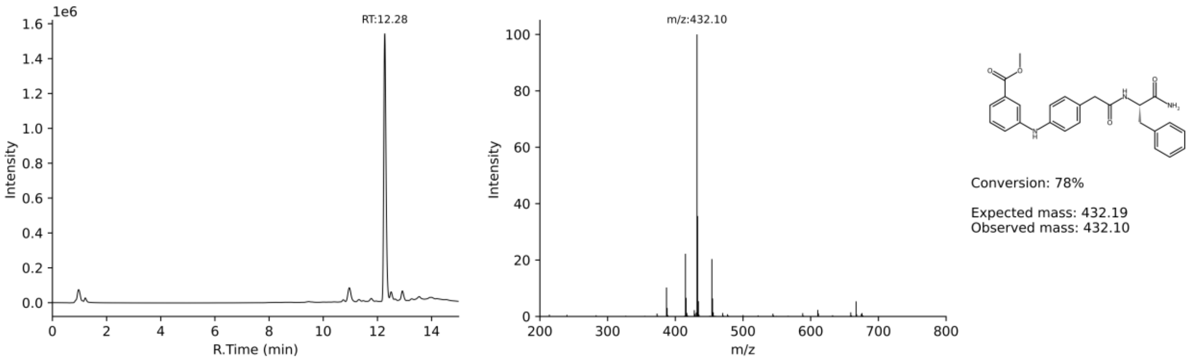

Fig. S80. LC-MS chromatogram of compound 7k.

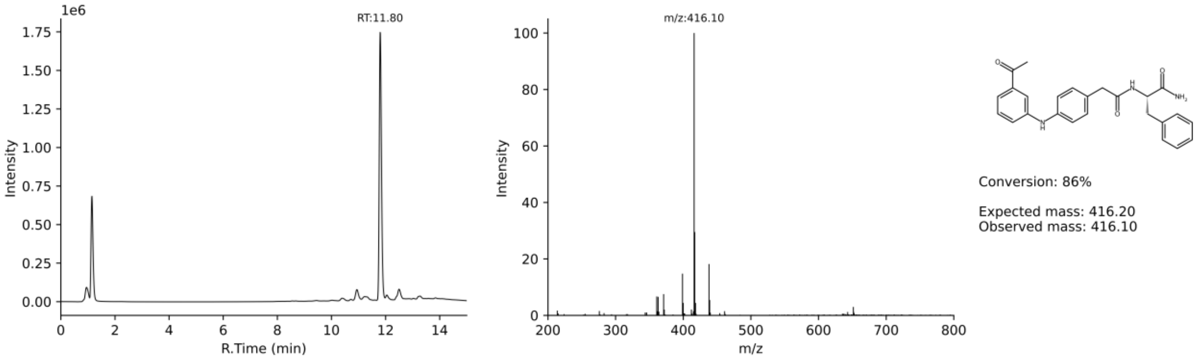

Fig. S81. LC-MS chromatogram of compound 7l.

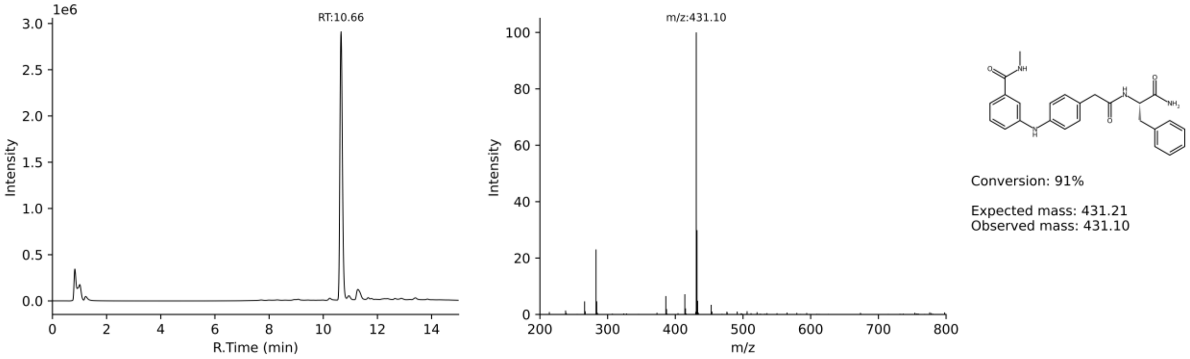

Fig. S82. LC-MS chromatogram of compound 7m.

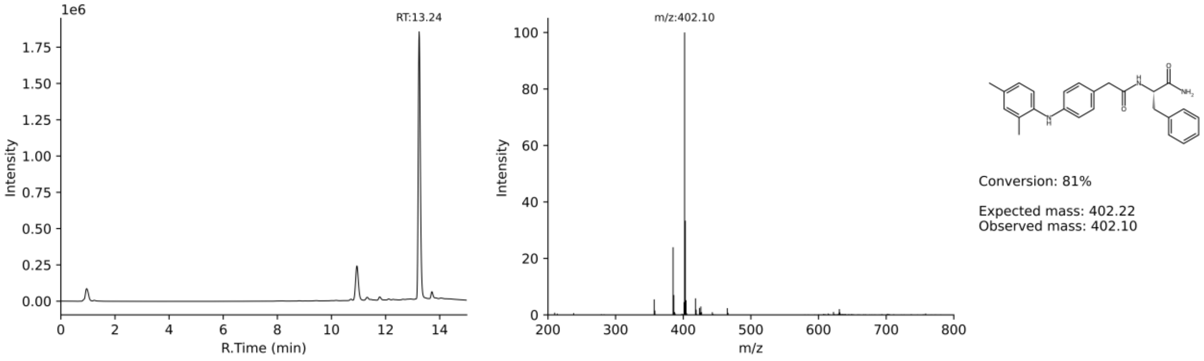

Fig. S83. LC-MS chromatogram of compound 7n.

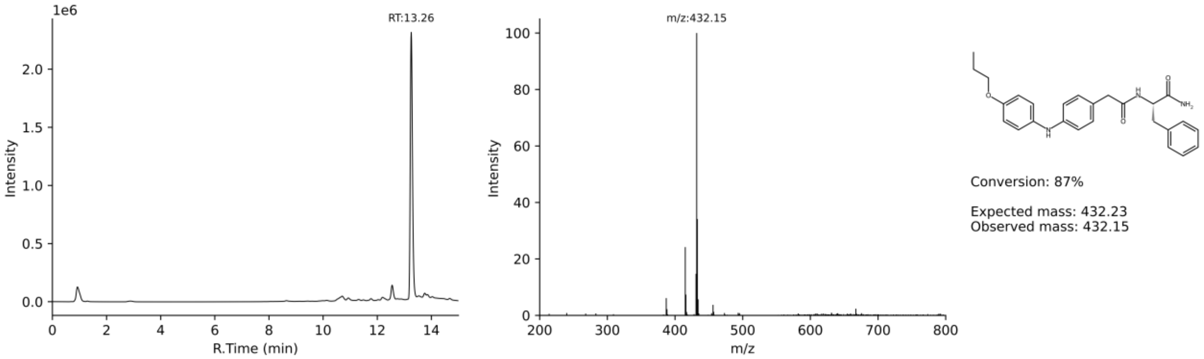

Fig. S84. LC-MS chromatogram of compound 7o.

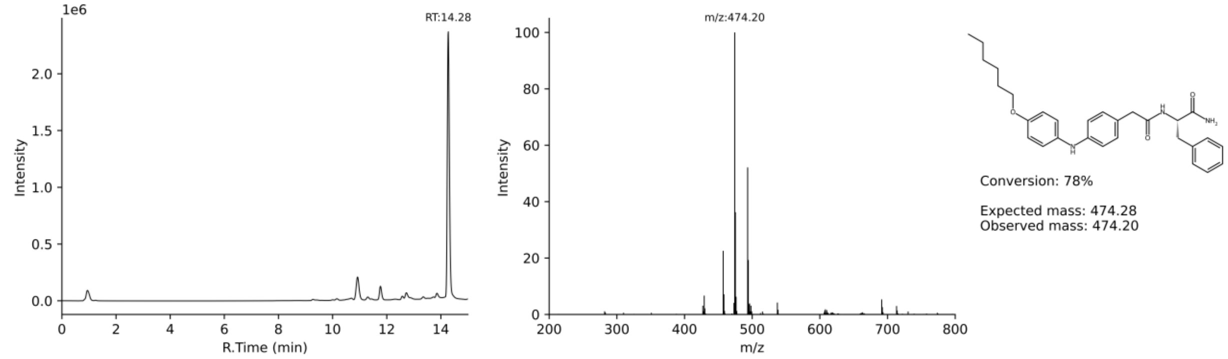

Fig. S85. LC-MS chromatogram of compound 7p.

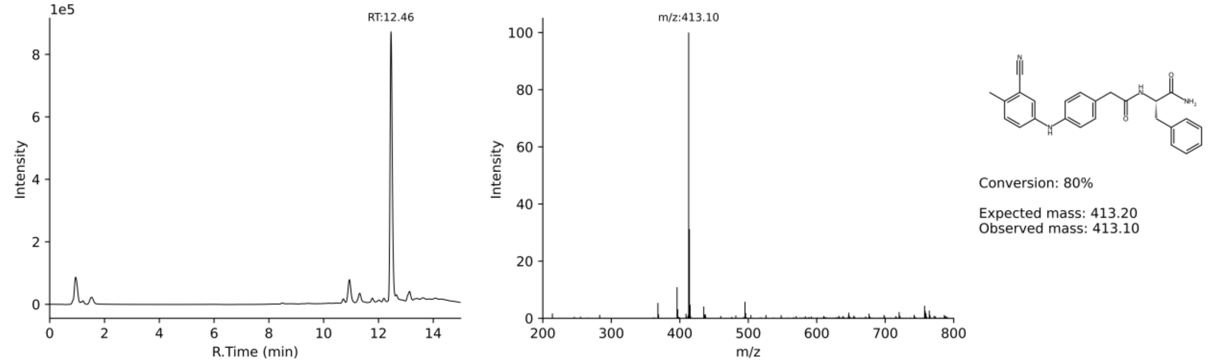

Fig. S86. LC-MS chromatogram of compound 7q.

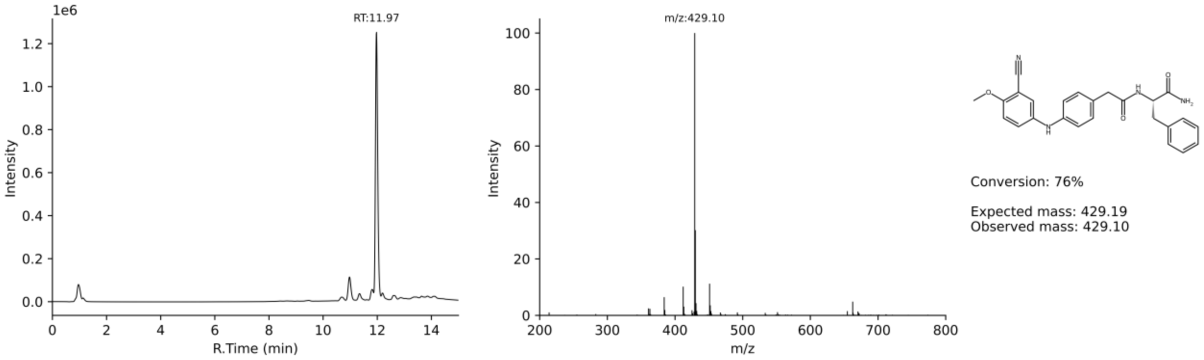

Fig. S87. LC-MS chromatogram of compound 7r.

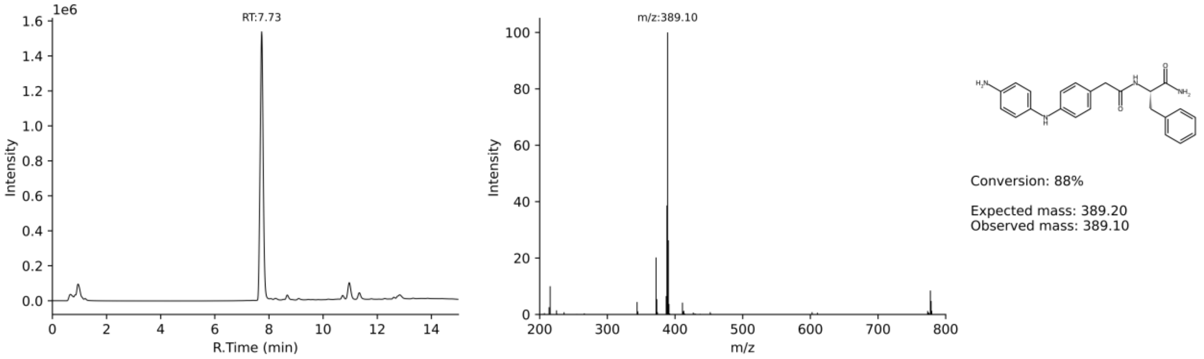

Fig. S88. LC-MS chromatogram of compound 7s.

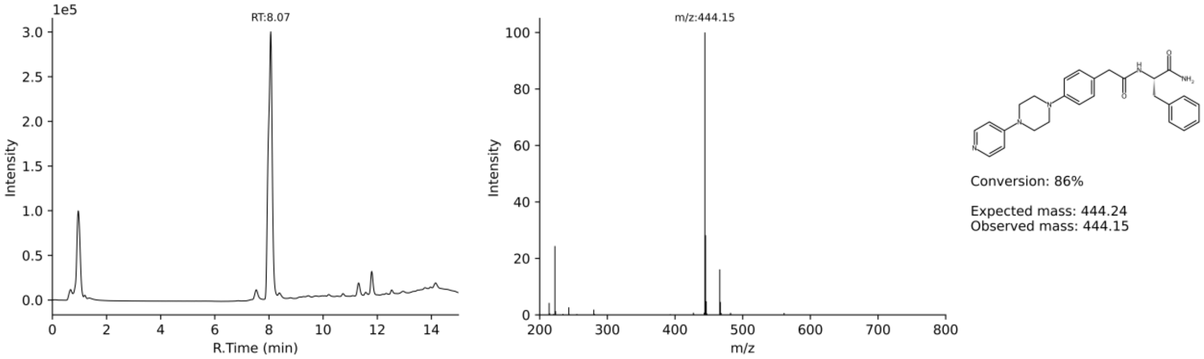

Fig. S89. LC-MS chromatogram of compound 7t.

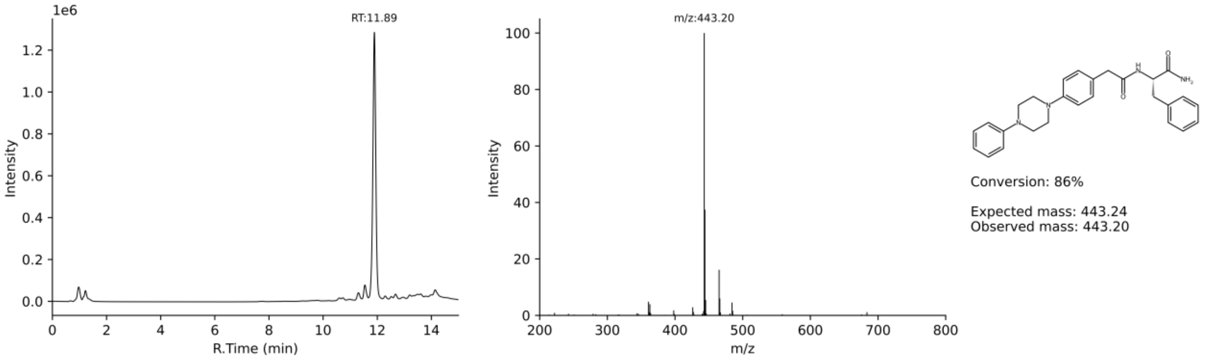

Fig. S90. LC-MS chromatogram of compound 7u.

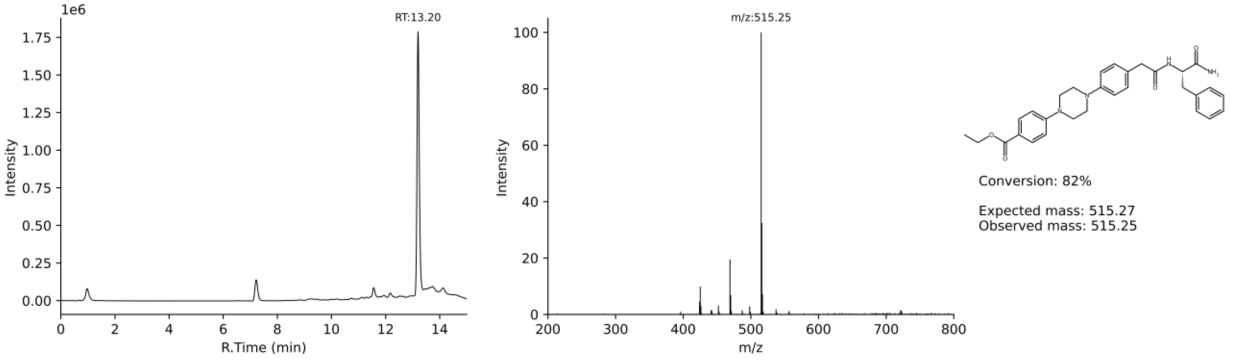

Fig. S91. LC-MS chromatogram of compound 7v.

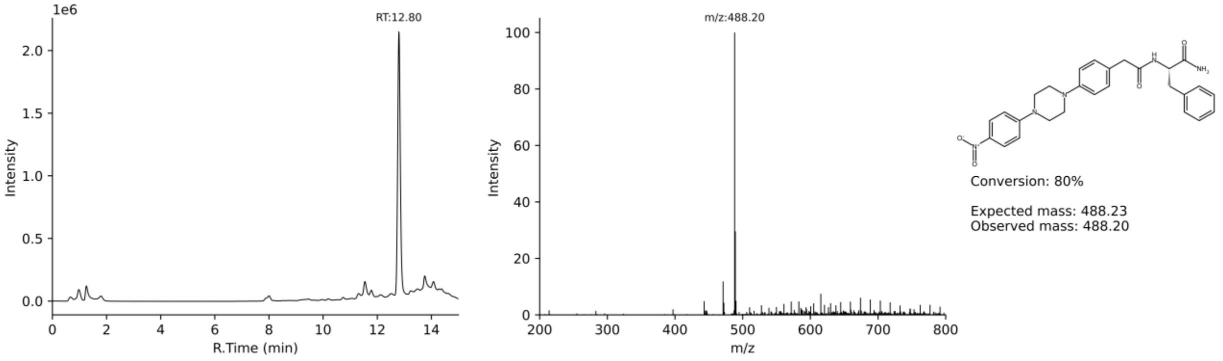

Fig. S92. LC-MS chromatogram of compound 7w.

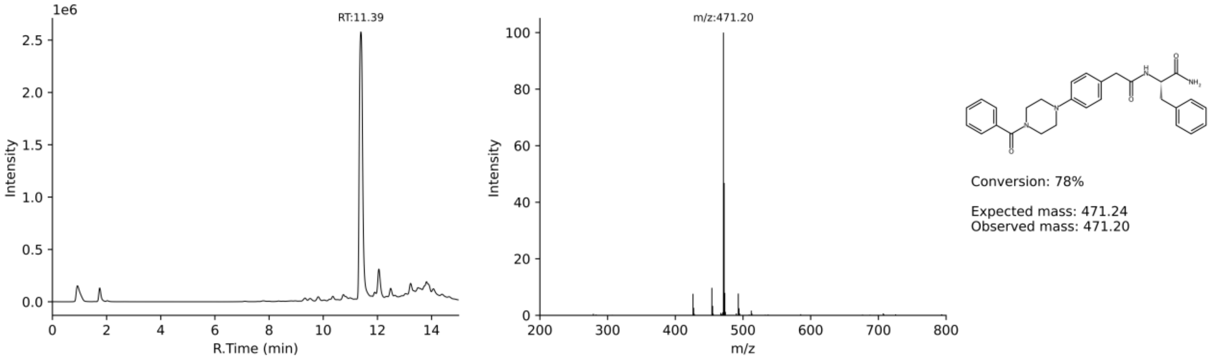

Fig. S93. LC-MS chromatogram of compound 7x.

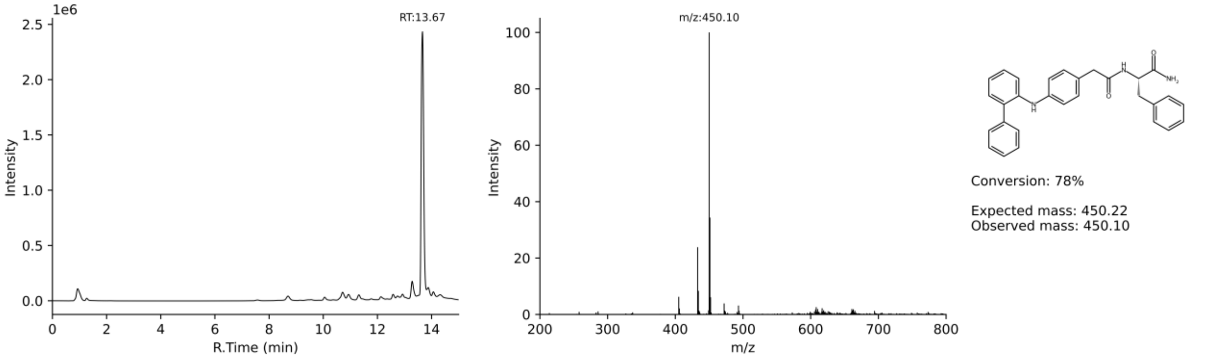

Fig. S94. LC-MS chromatogram of compound 7y.

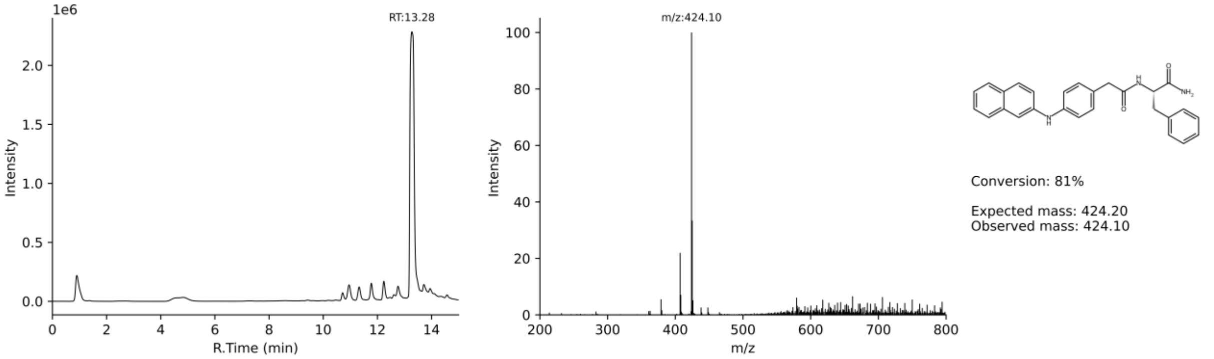

Fig. S95. LC-MS chromatogram of compound 7z.

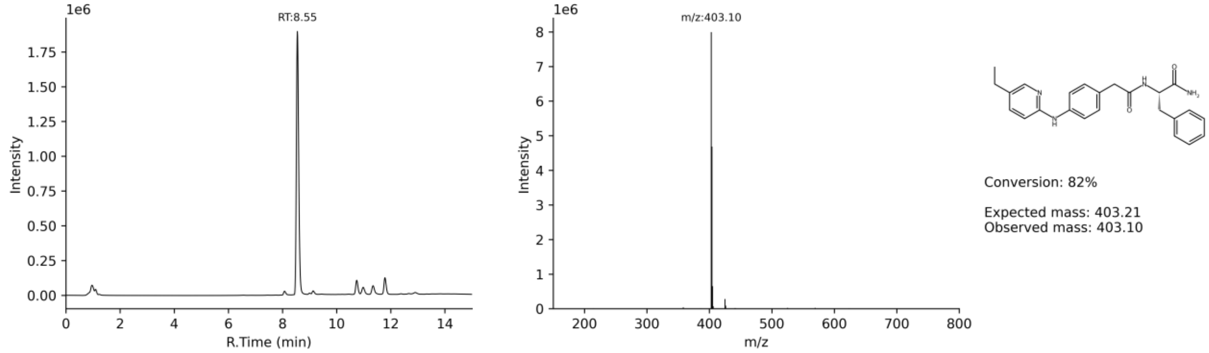

Fig. S96. LC-MS chromatogram of compound 7aa.

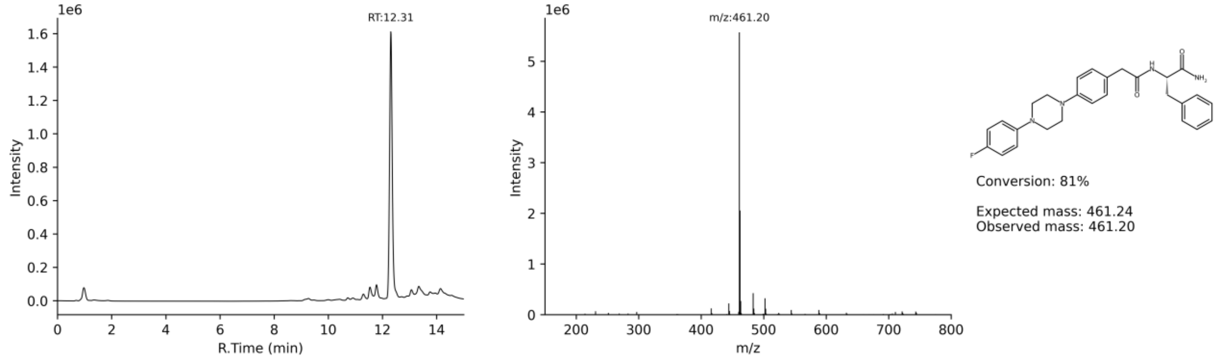

Fig. S97. LC-MS chromatogram of compound 7ab.

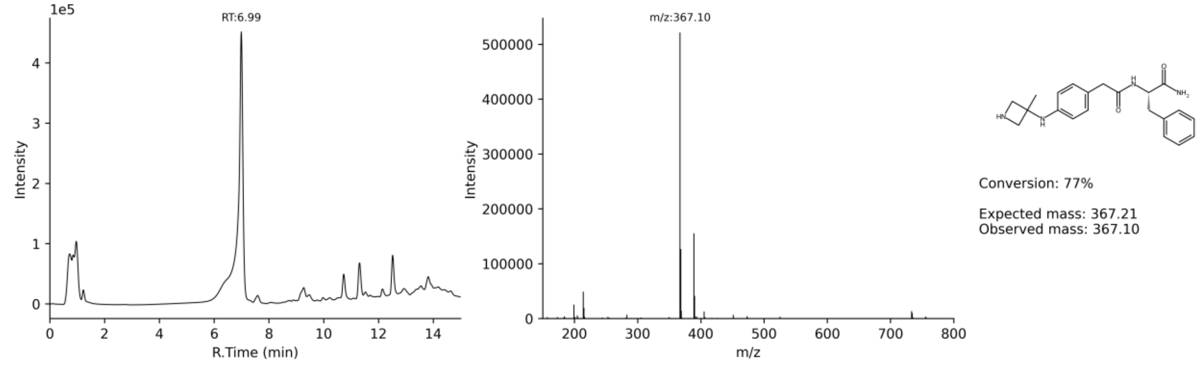

Fig. S98. LC-MS chromatogram of compound 7ac.

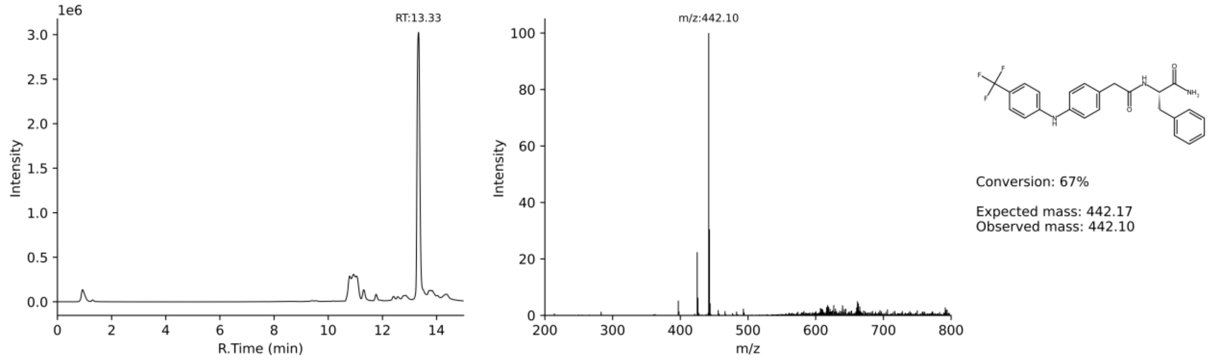

Fig. S99. LC-MS chromatogram of compound 8a.

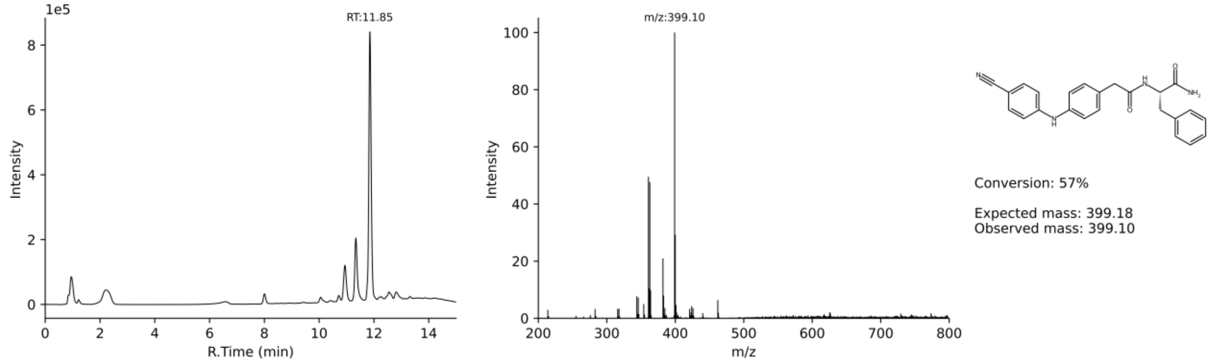

Fig. S100. LC-MS chromatogram of compound 8b.

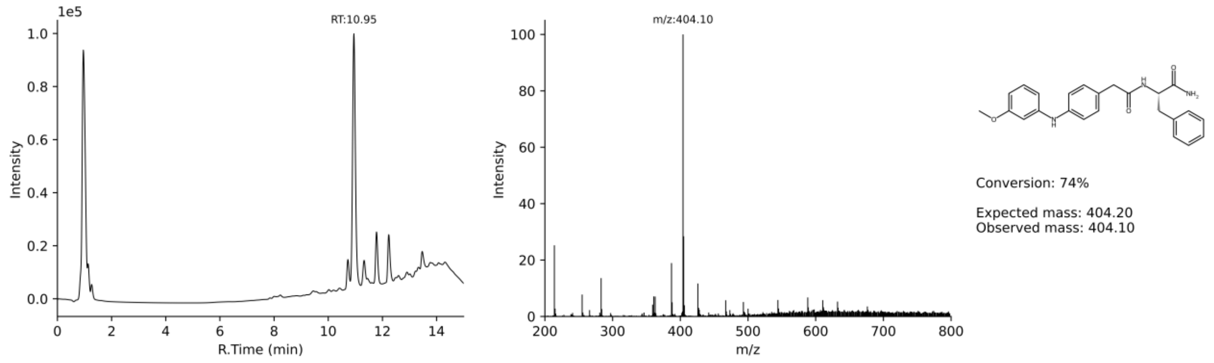

Fig. S101. LC-MS chromatogram of compound 8c.

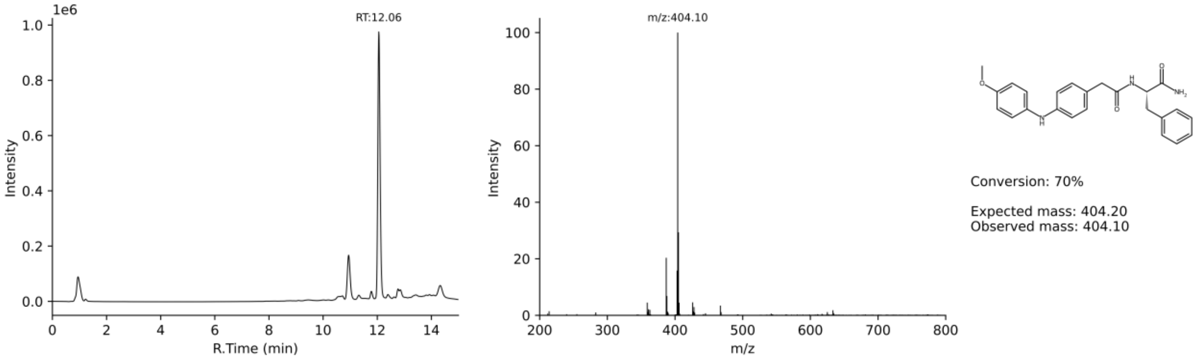

Fig. S102. LC-MS chromatogram of compound 8d.

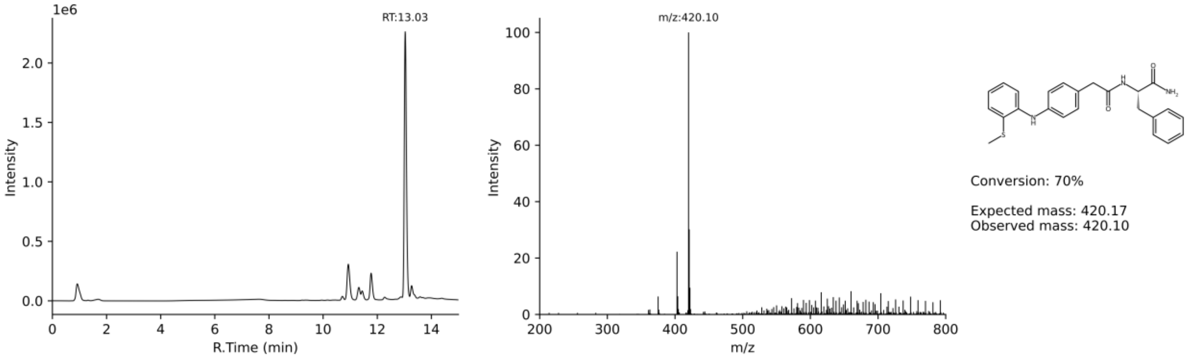

Fig. S103. LC-MS chromatogram of compound 8e.

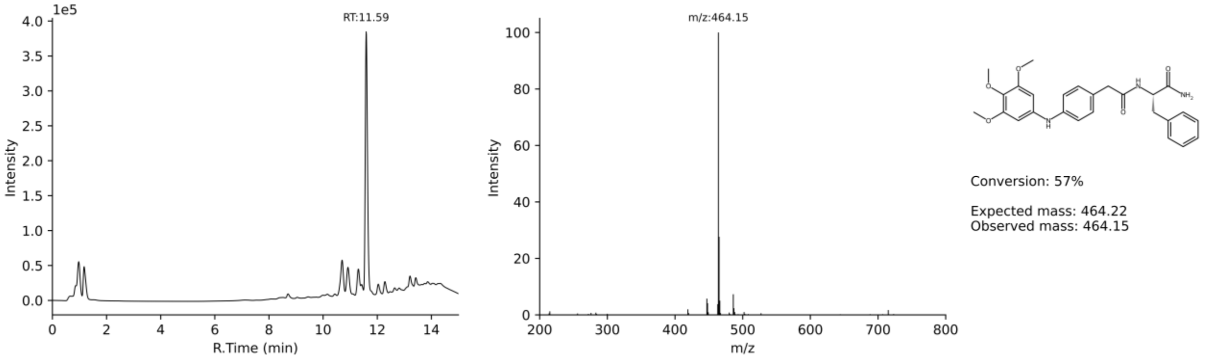

Fig. S104. LC-MS chromatogram of compound 8f.

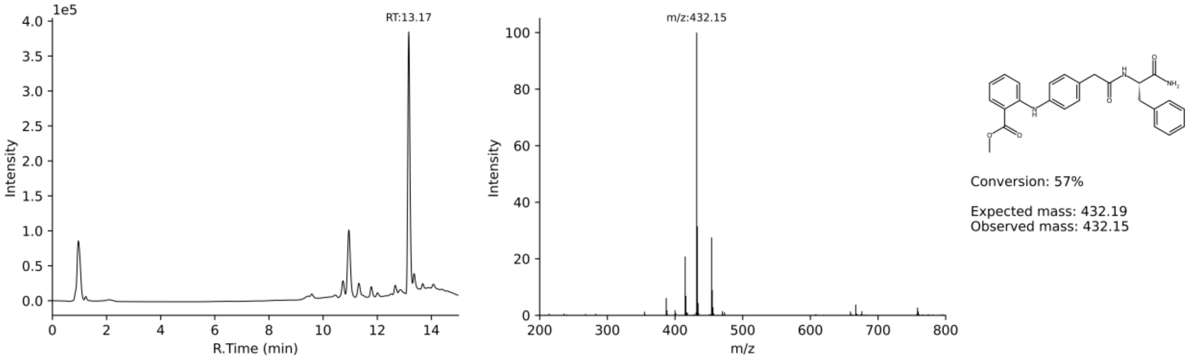

Fig. S105. LC-MS chromatogram of compound 8g.

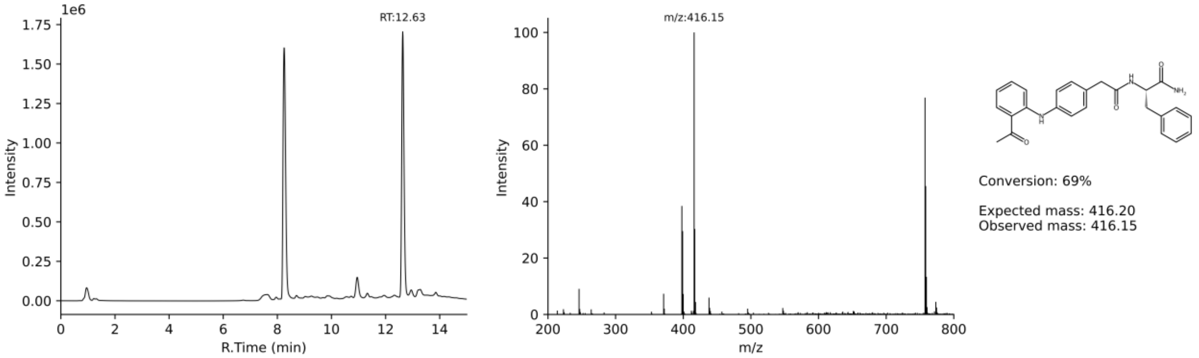

Fig. S106. LC-MS chromatogram of compound 8h.

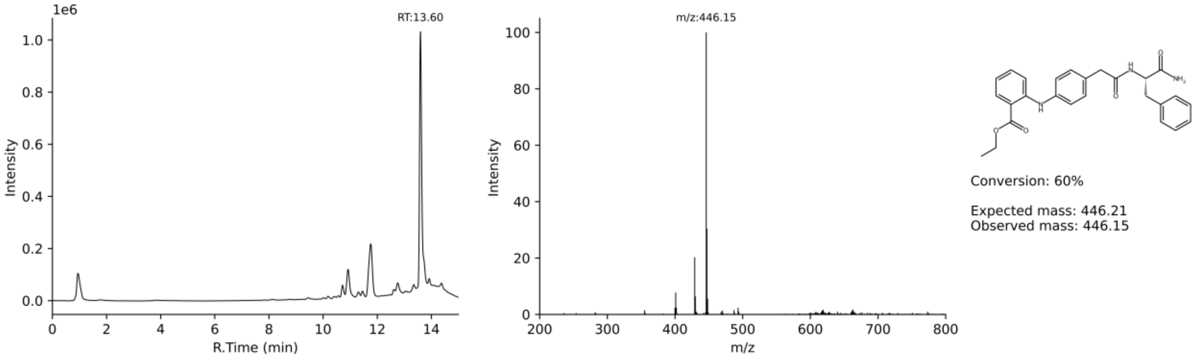

Fig. S107. LC-MS chromatogram of compound 8i.

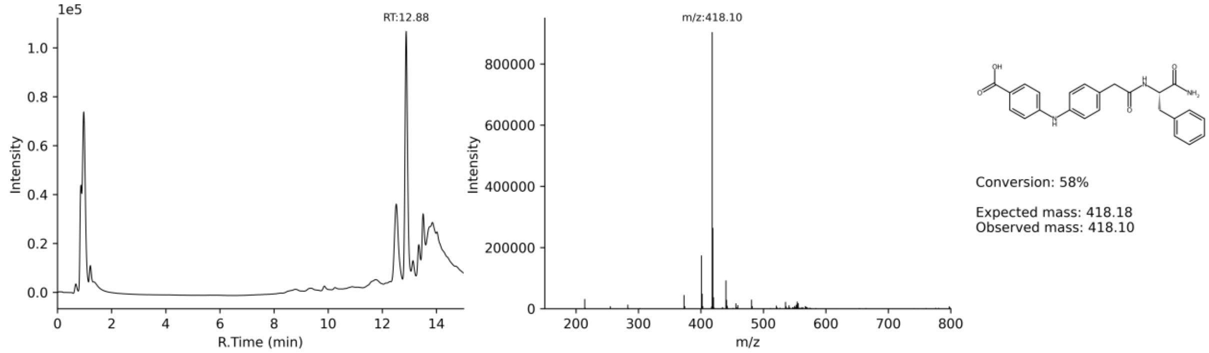

Fig. S108. LC-MS chromatogram of compound 8j.

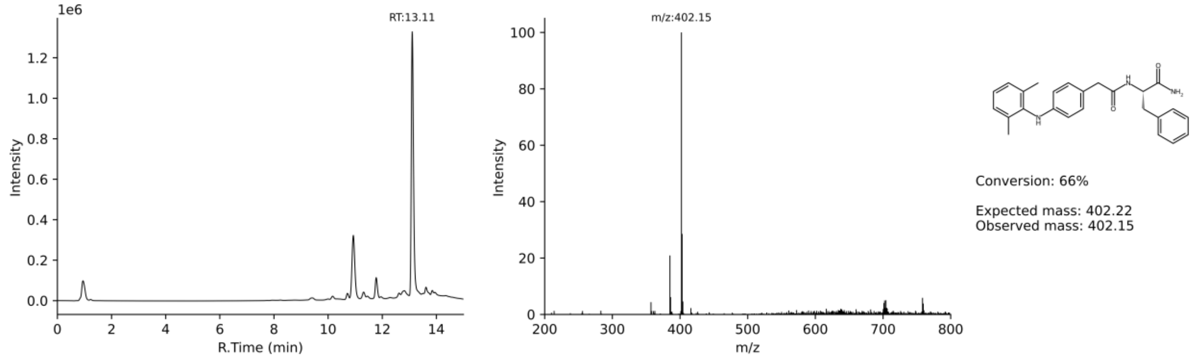

Fig. S109. LC-MS chromatogram of compound 8k.

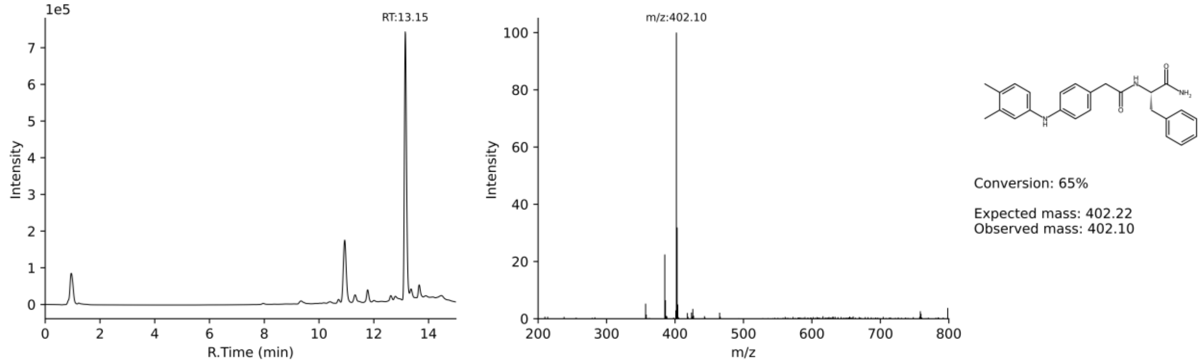

Fig. S110. LC-MS chromatogram of compound 8l.

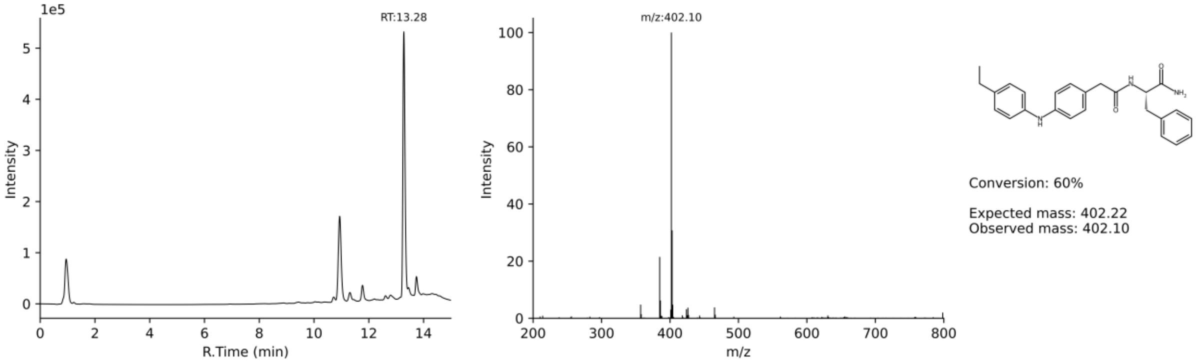

Fig. S111. LC-MS chromatogram of compound 8m.

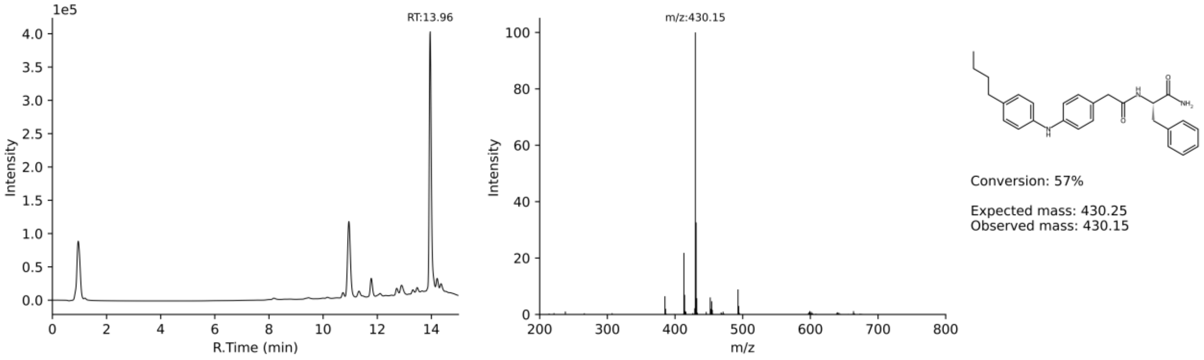

Fig. S112. LC-MS chromatogram of compound 8n.

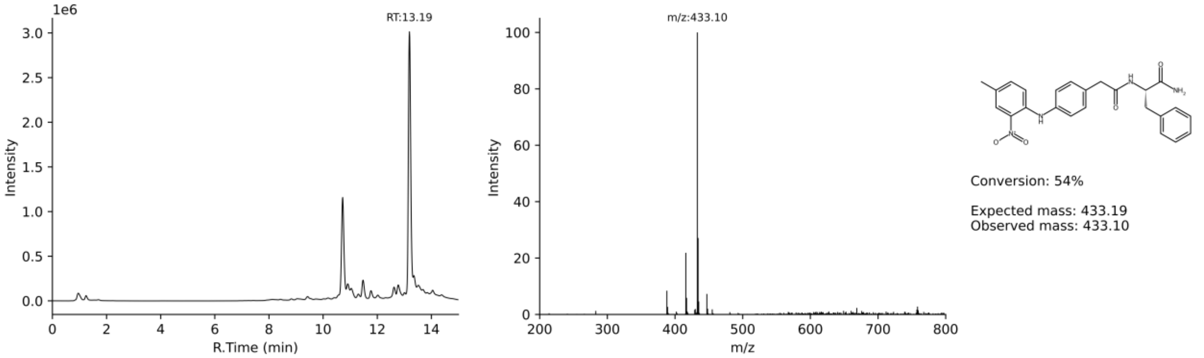

Fig. S113. LC-MS chromatogram of compound 8o.

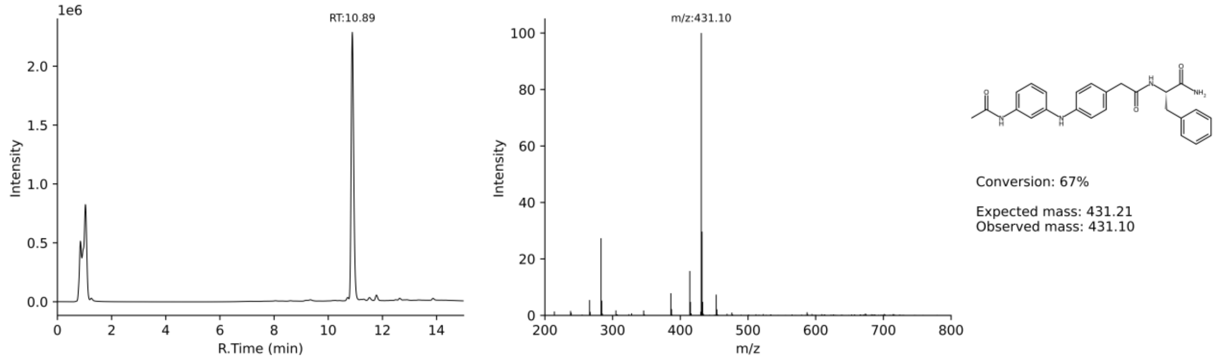

Fig. S114. LC-MS chromatogram of compound 8p.

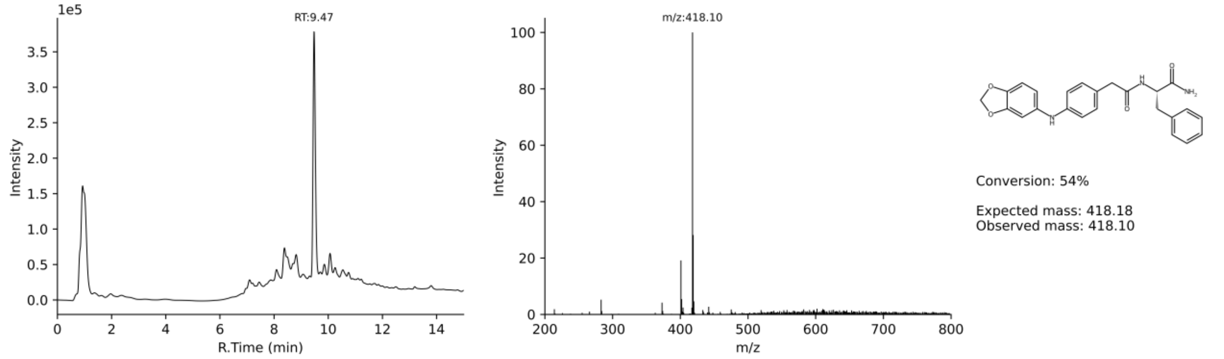

Fig. S115. LC-MS chromatogram of compound 8q.

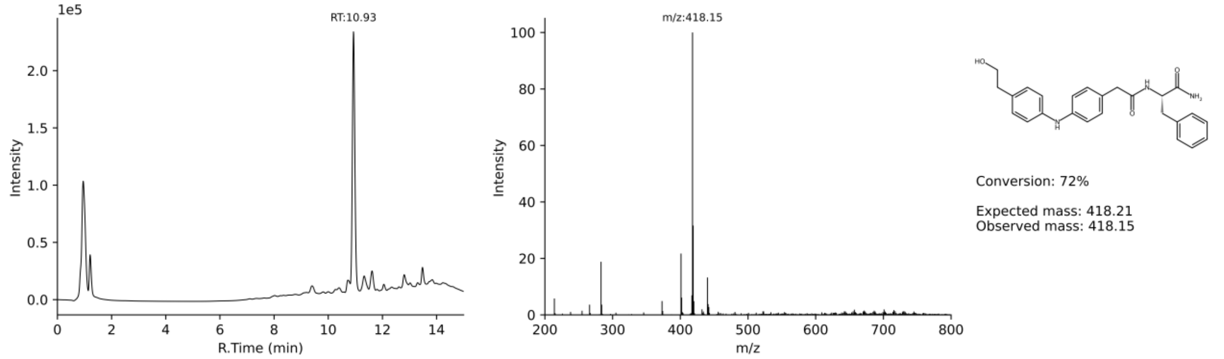

Fig. S116. LC-MS chromatogram of compound 8r.

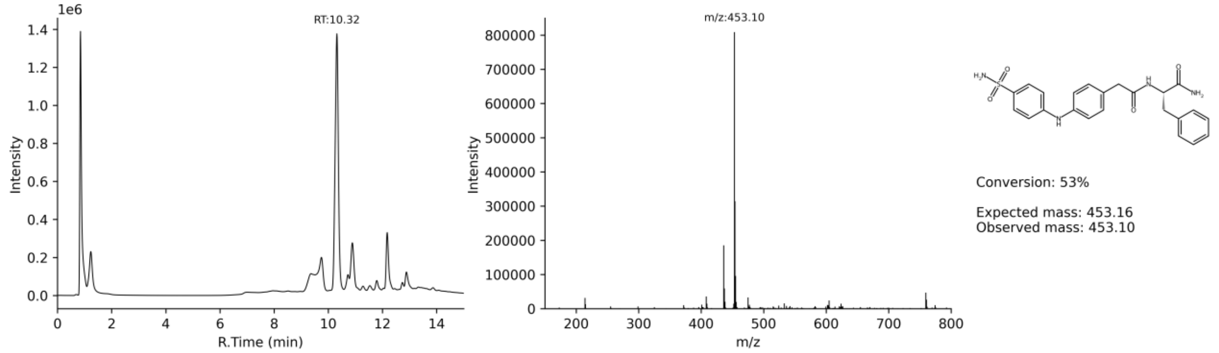

Fig. S117. LC-MS chromatogram of compound 8s.

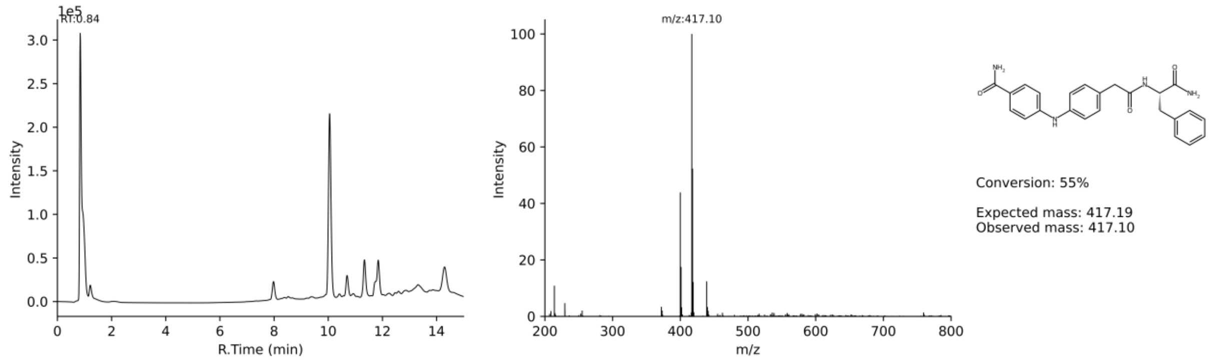

Fig. S118. LC-MS chromatogram of compound 8t.

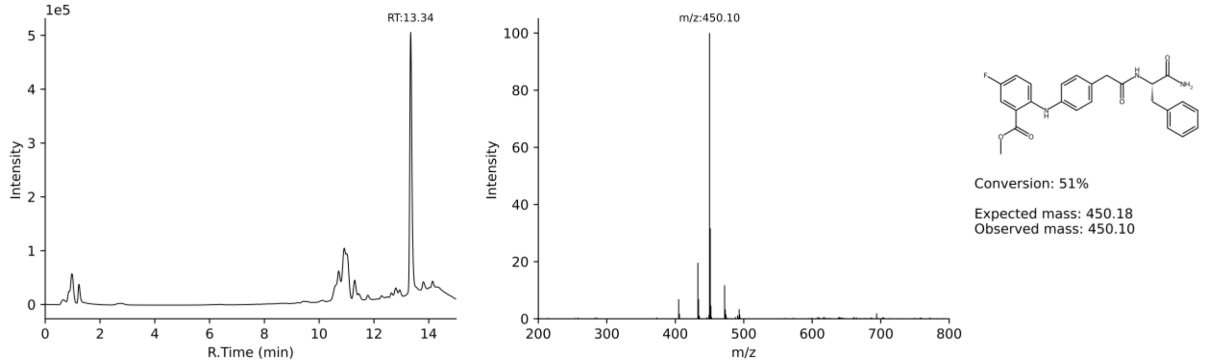

Fig. S119. LC-MS chromatogram of compound 8u.

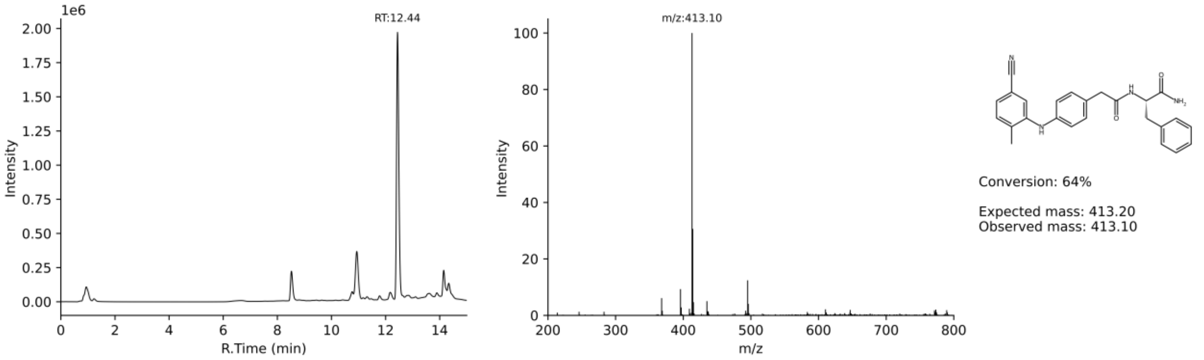

Fig. S120. LC-MS chromatogram of compound 8v.

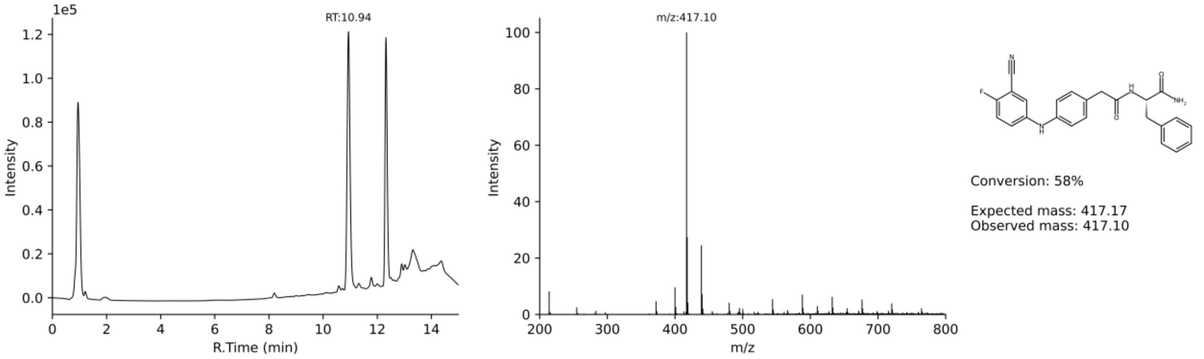

Fig. S121. LC-MS chromatogram of compound 8w.

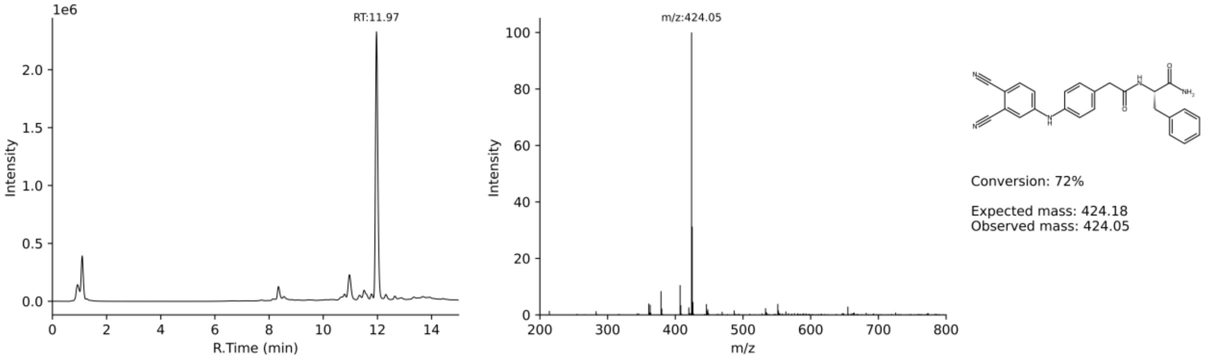

Fig. S122. LC-MS chromatogram of compound 8x.

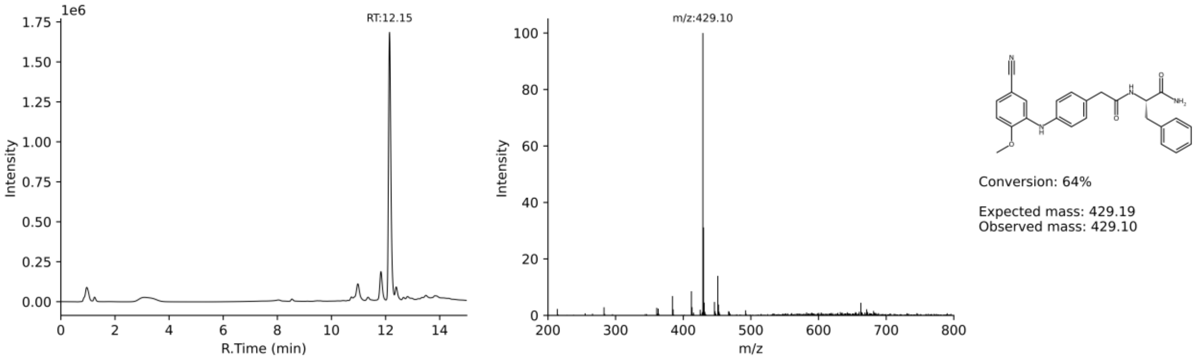

Fig. S123. LC-MS chromatogram of compound 8y.

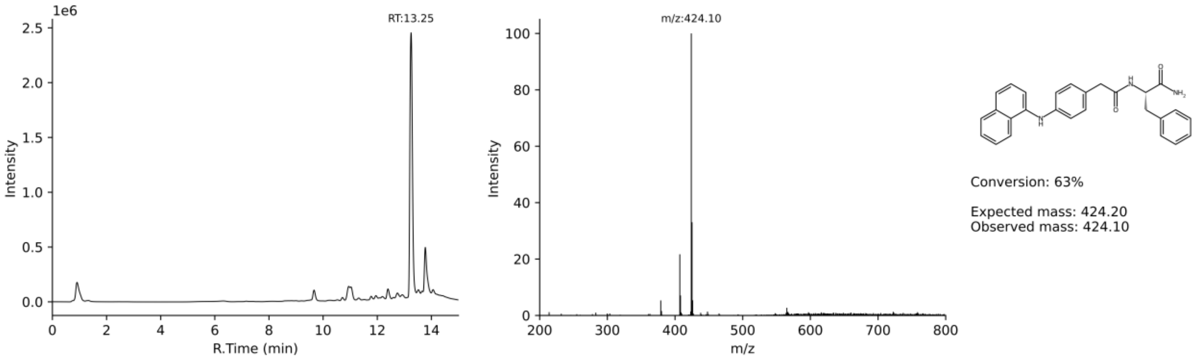

Fig. S124. LC-MS chromatogram of compound 8z.

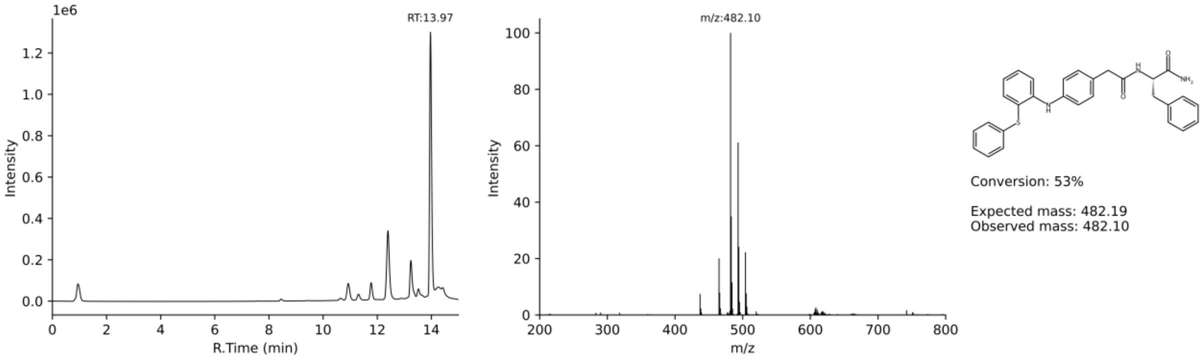

Fig. S125. LC-MS chromatogram of compound 8aa.

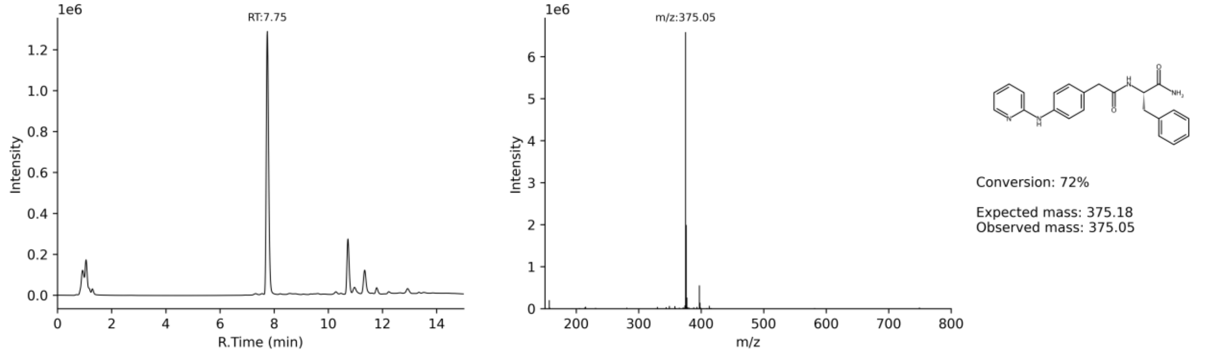

Fig. S126. LC-MS chromatogram of compound 8ab.

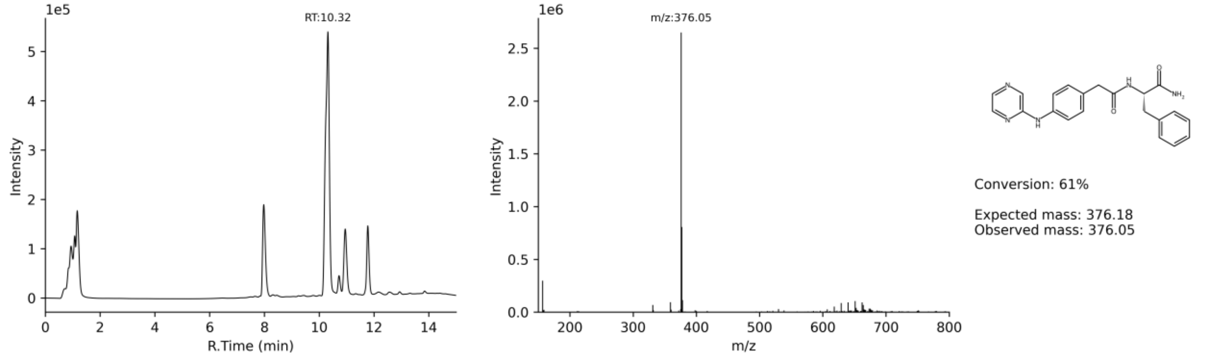

Fig. S127. LC-MS chromatogram of compound 8ac.

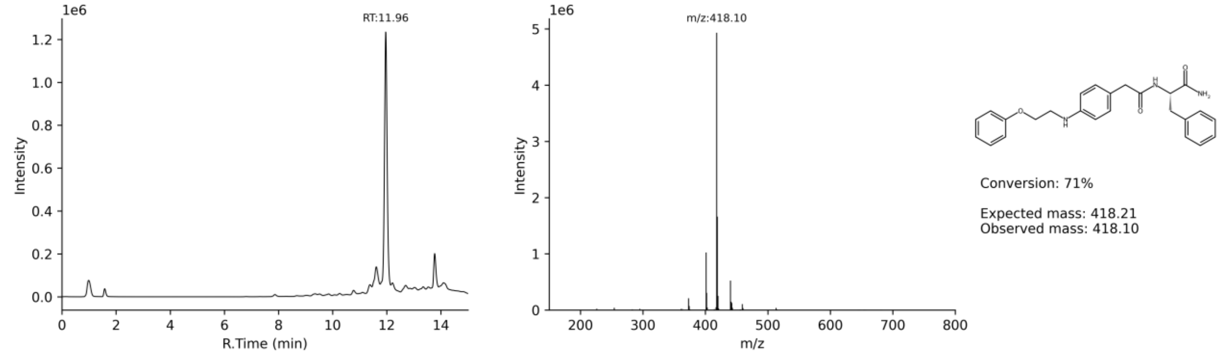

Fig. S128. LC-MS chromatogram of compound 8ad.

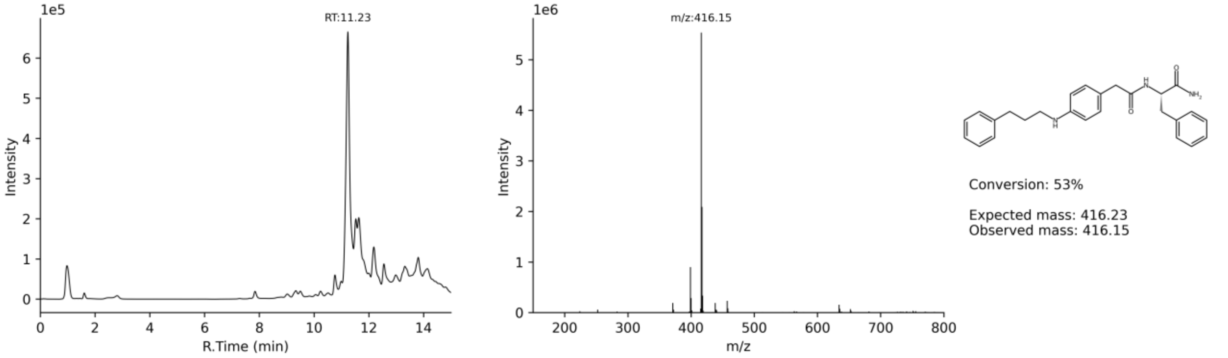

Fig. S129. LC-MS chromatogram of compound 8ae.

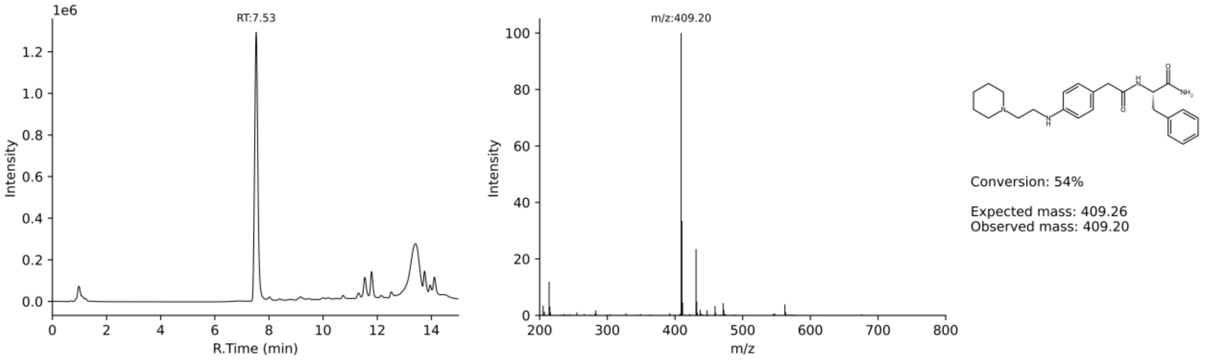

Fig. S130. LC-MS chromatogram of compound 8af.

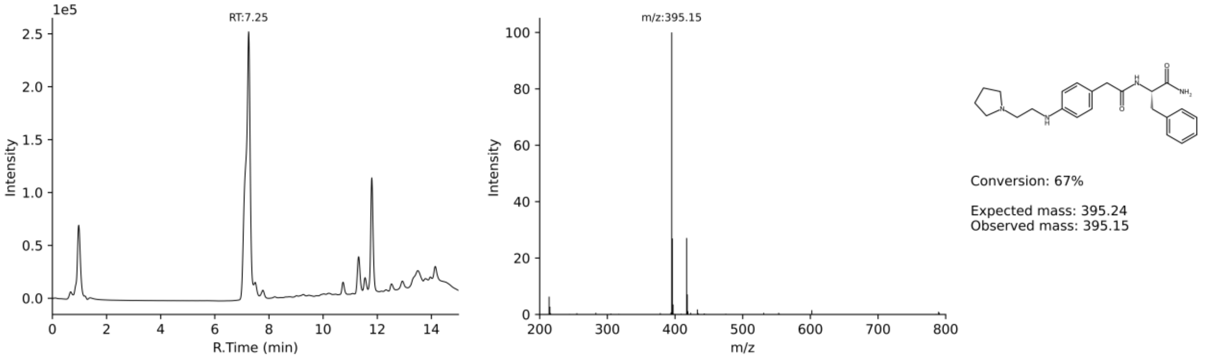

Fig. S131. LC-MS chromatogram of compound 8ag.

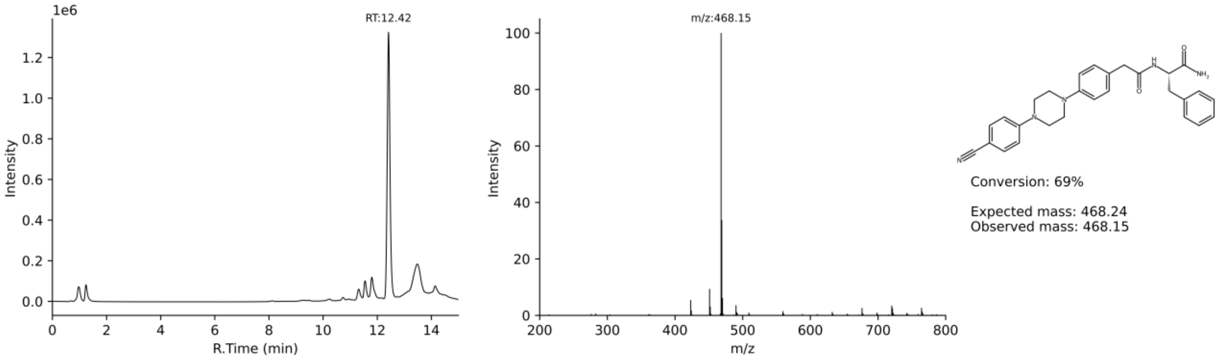

Fig. S132. LC-MS chromatogram of compound 8ah.

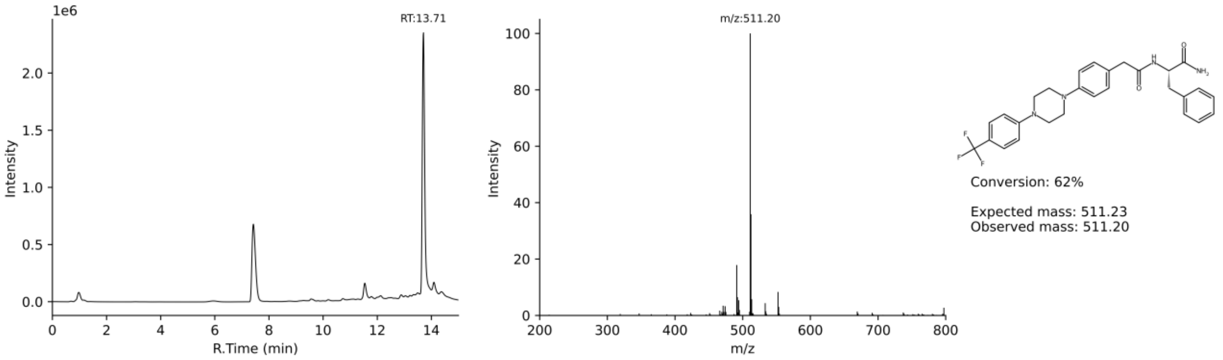

Fig. S133. LC-MS chromatogram of compound 8ai.

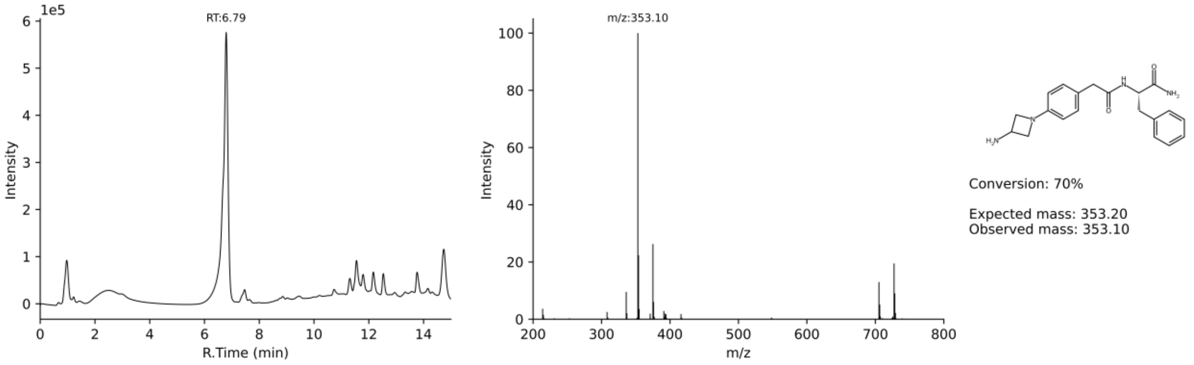

Fig. S134. LC-MS chromatogram of compound 8aj.

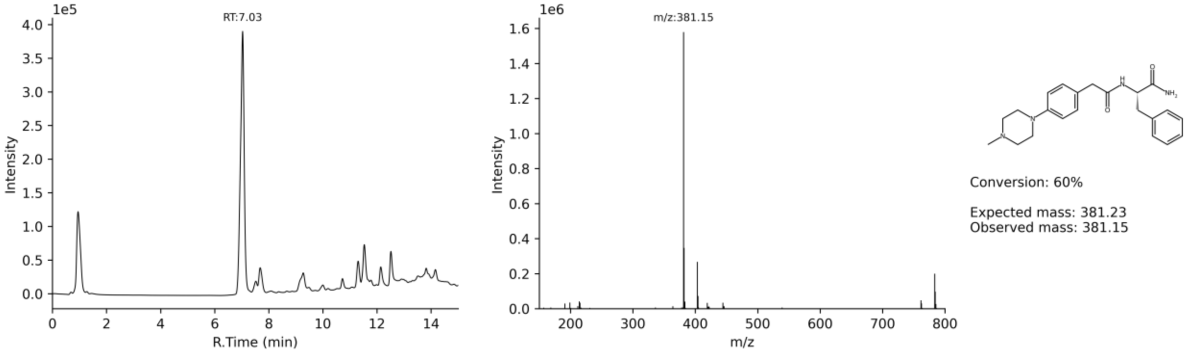

Fig. S135. LC-MS chromatogram of compound 8ak.

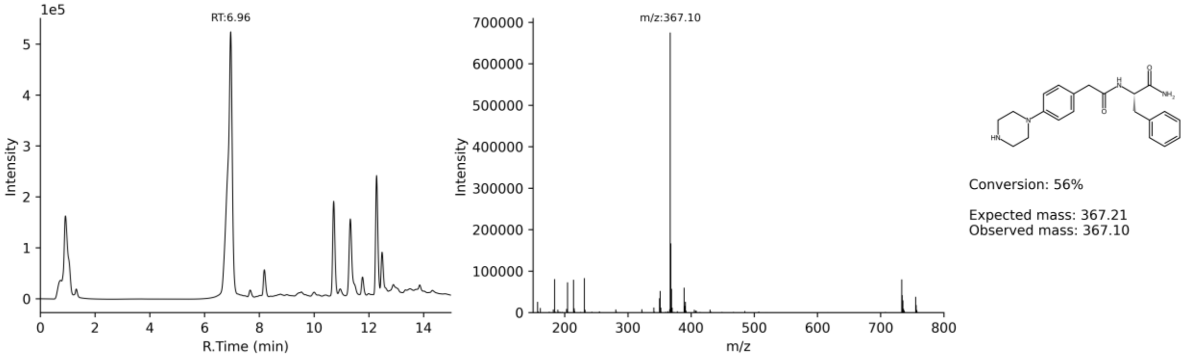

Fig. S136. LC-MS chromatogram of compound 8al.

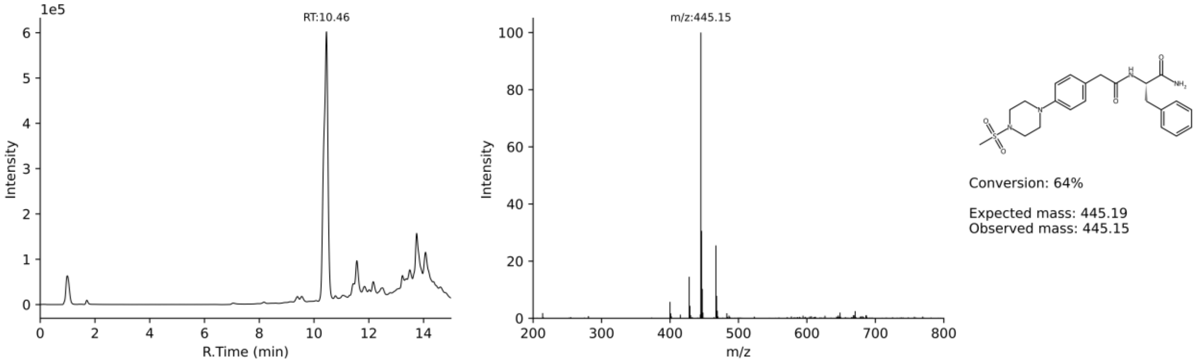

Fig. S137. LC-MS chromatogram of compound 8am.

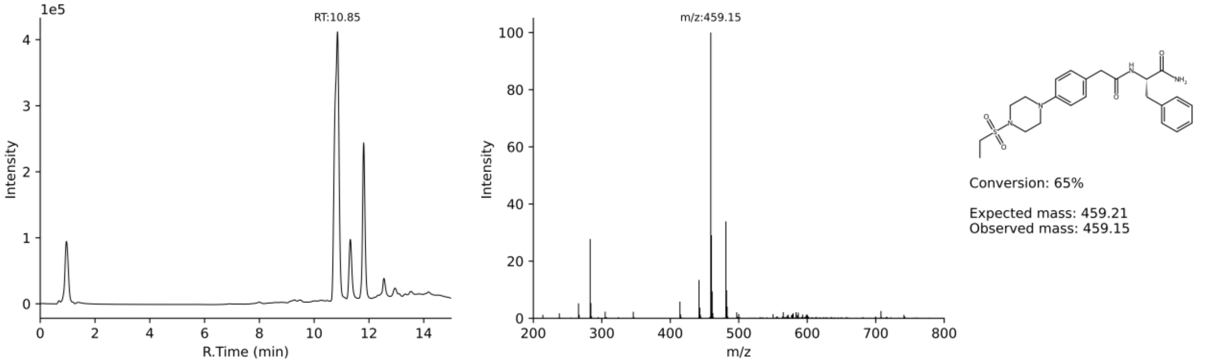

Fig. S138. LC-MS chromatogram of compound 8an.

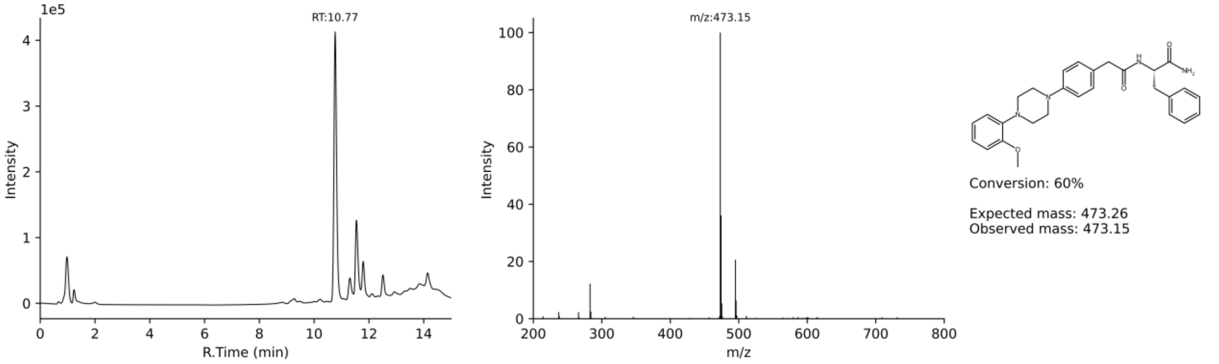

Fig. S139. LC-MS chromatogram of compound 8ao.

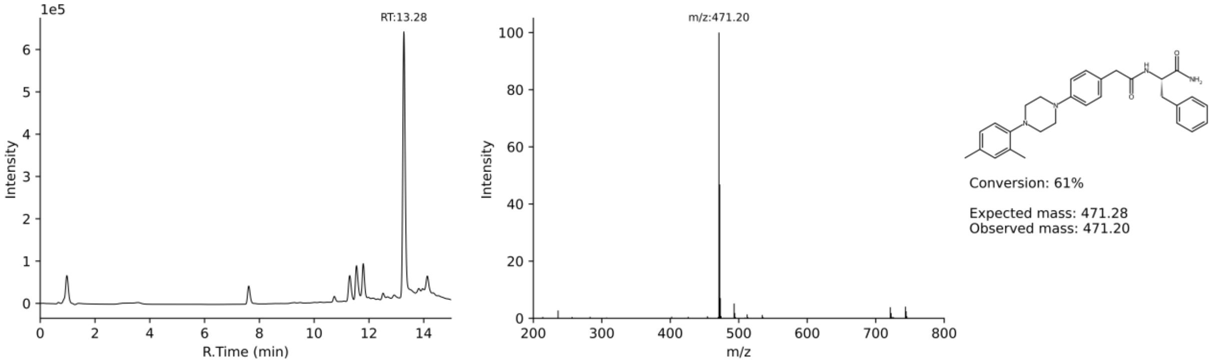

Fig. S140. LC-MS chromatogram of compound 8ap.

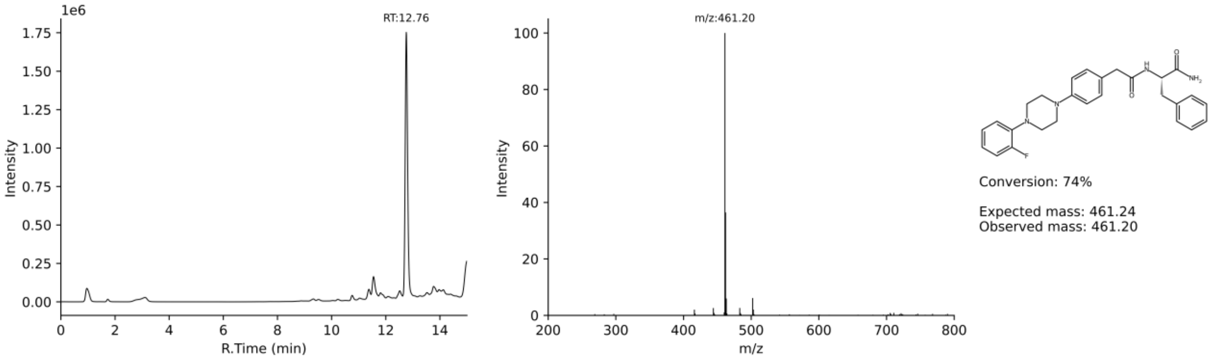

Fig. S141. LC-MS chromatogram of compound 8aq.

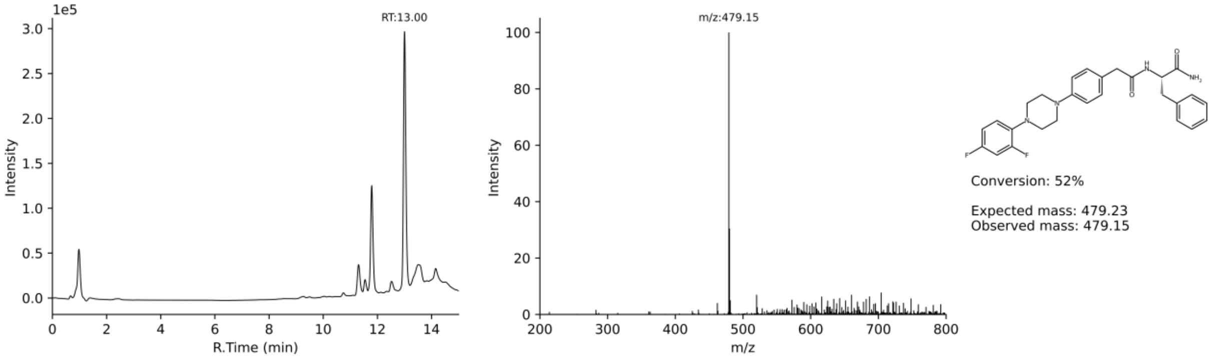

Fig. S142. LC-MS chromatogram of compound 8ar.

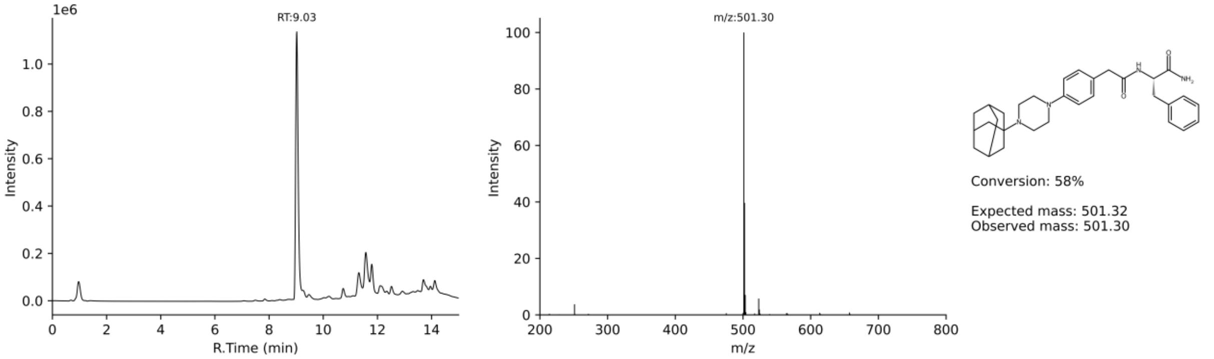

Fig. S143. LC-MS chromatogram of compound 8as.

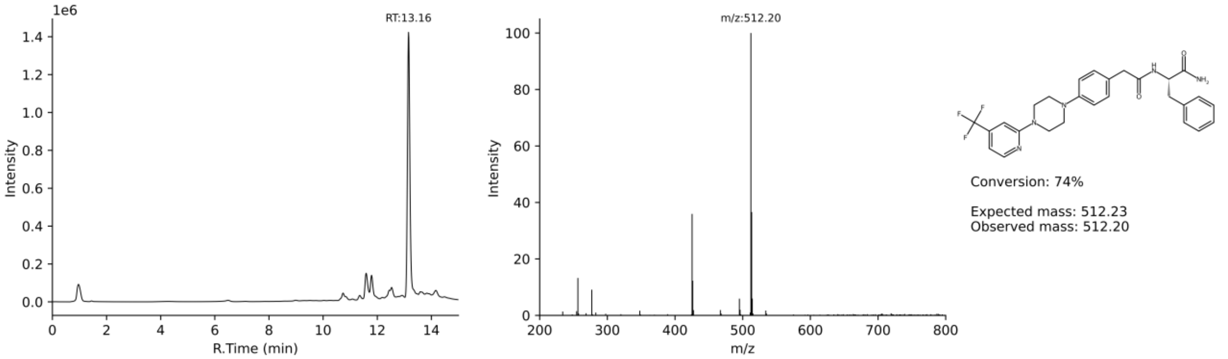

Fig. S144. LC-MS chromatogram of compound 8at.

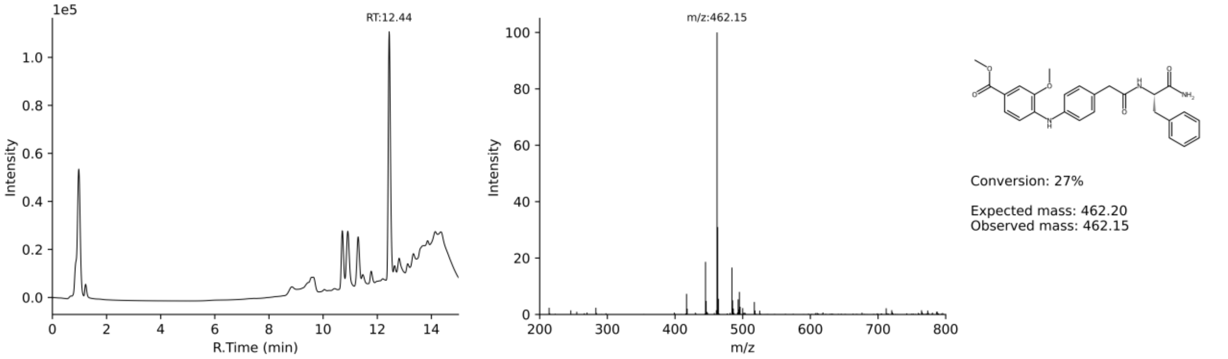

Fig. S145. LC-MS chromatogram of compound 9a.

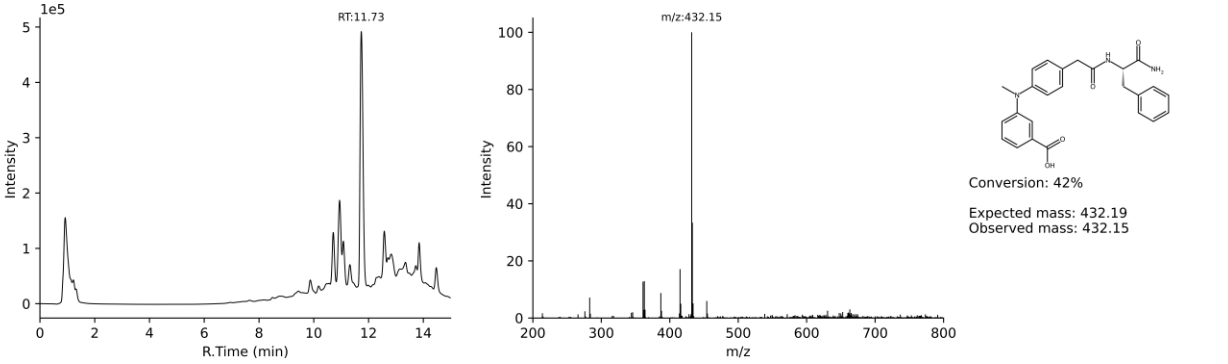

Fig. S146. LC-MS chromatogram of compound 9b.

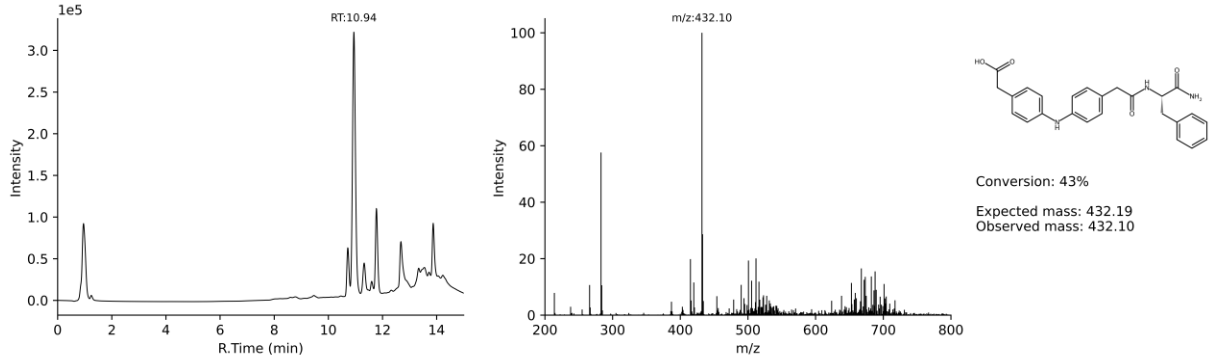

Fig. S147. LC-MS chromatogram of compound 9c.

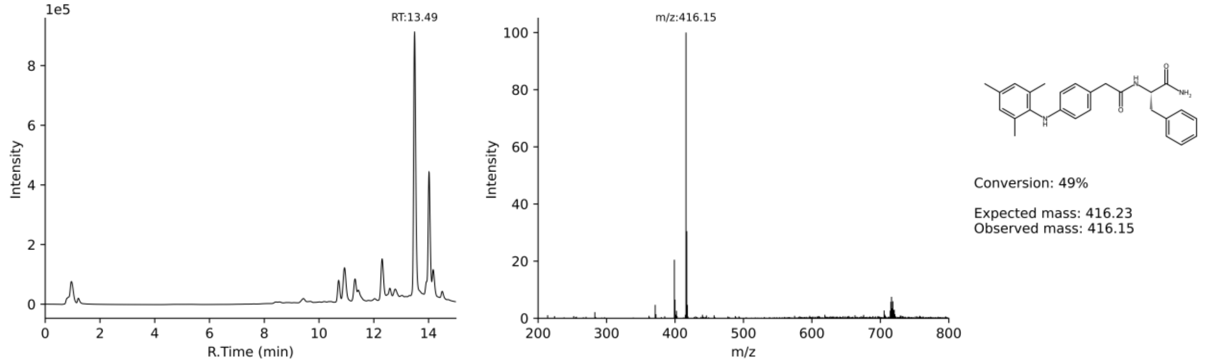

Fig. S148. LC-MS chromatogram of compound 9d.

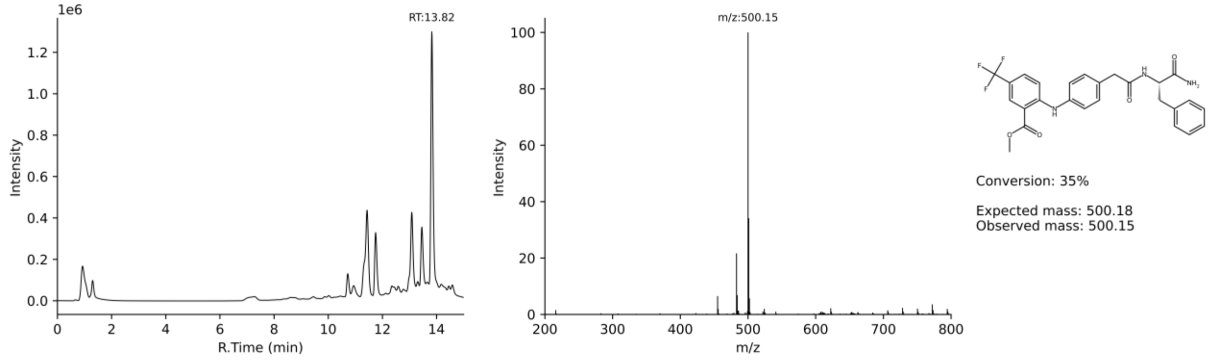

Fig. S149. LC-MS chromatogram of compound 9e.

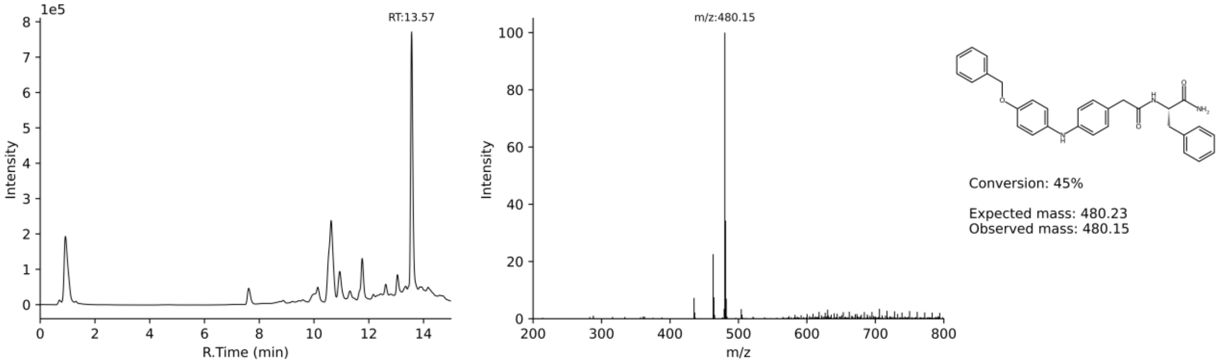

Fig. S150. LC-MS chromatogram of compound 9f.

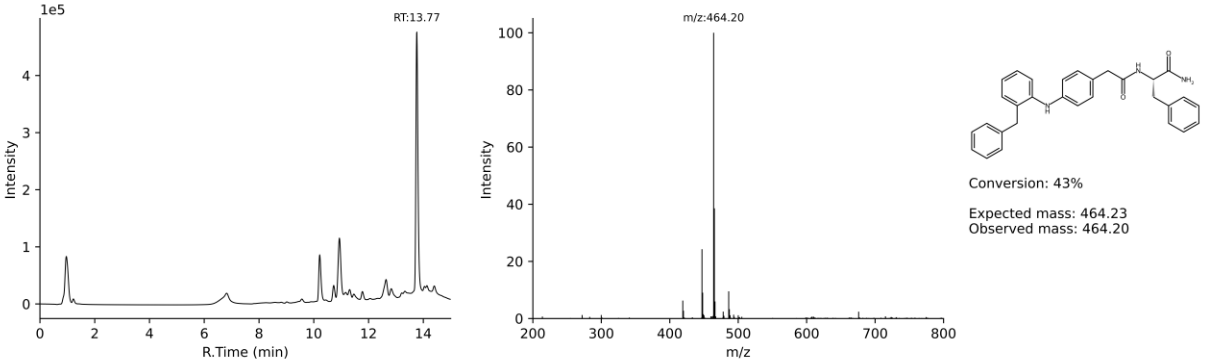

Fig. S151. LC-MS chromatogram of compound 9g.

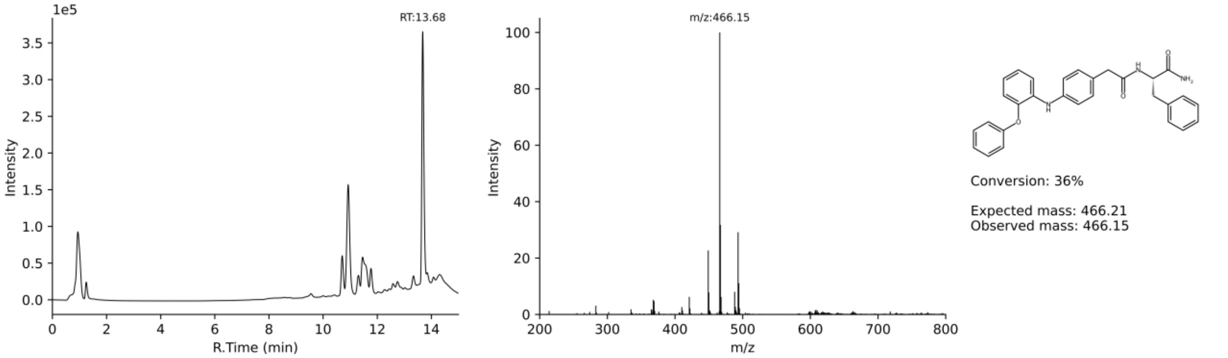

Fig. S152. LC-MS chromatogram of compound 9h.

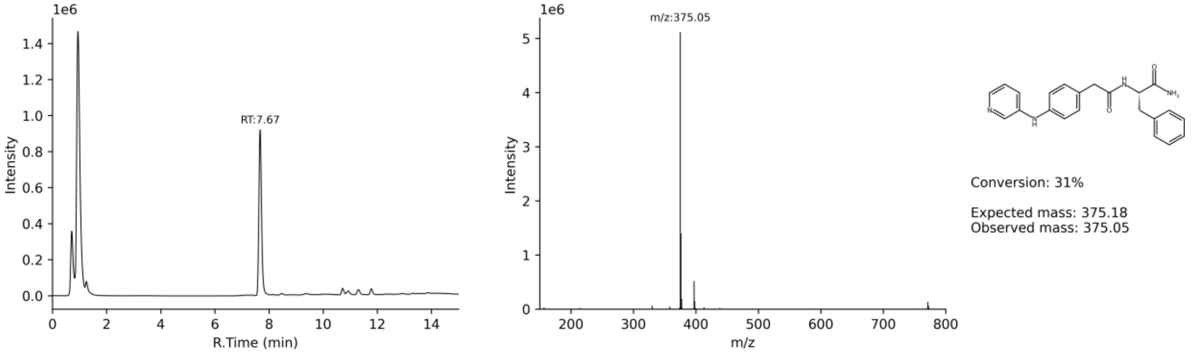

Fig. S153. LC-MS chromatogram of compound 9i.

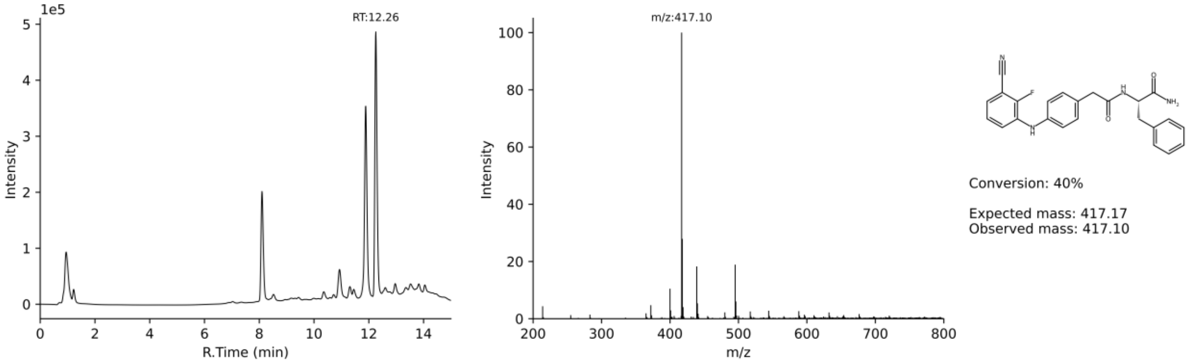

Fig. S154. LC-MS chromatogram of compound 9j.

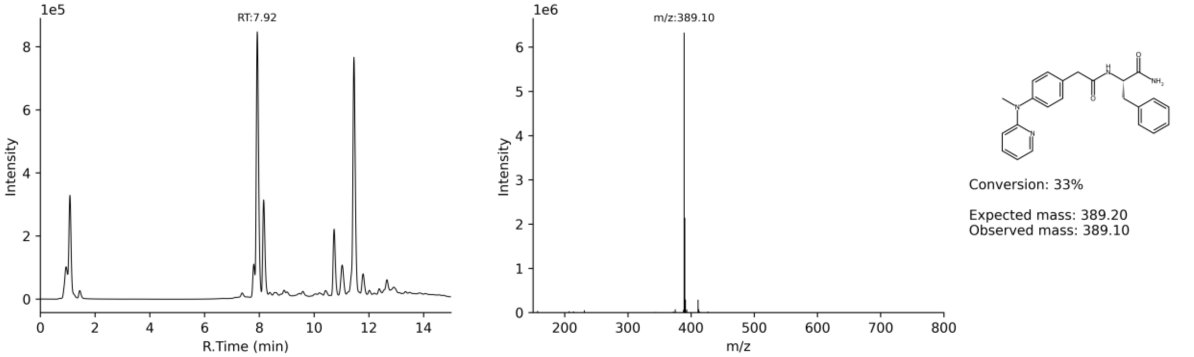

Fig. S155. LC-MS chromatogram of compound 9k.

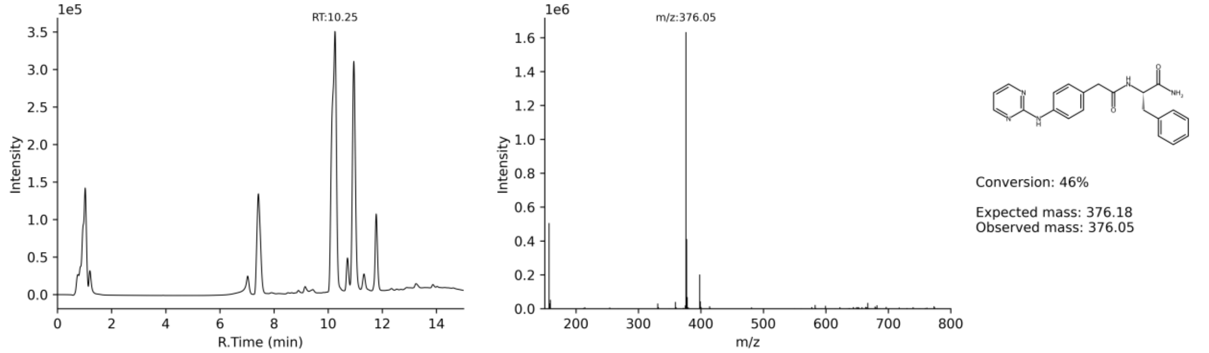

Fig. S156. LC-MS chromatogram of compound 9l.

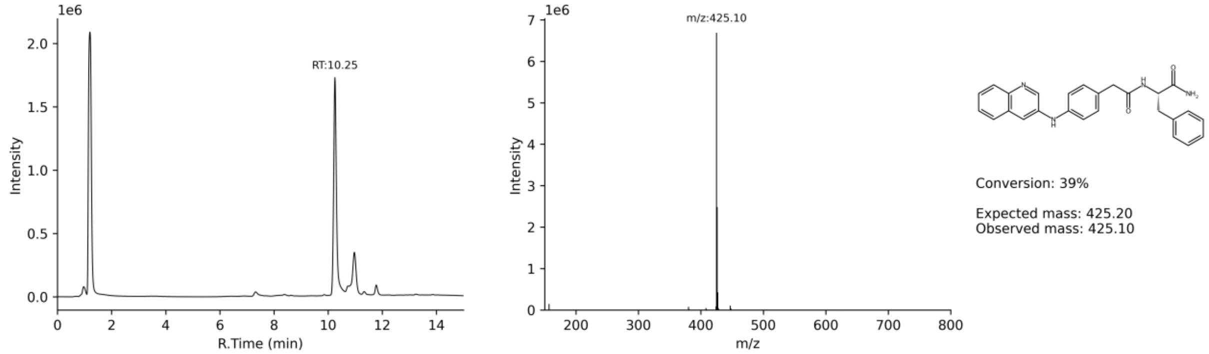

Fig. S157. LC-MS chromatogram of compound 9m.

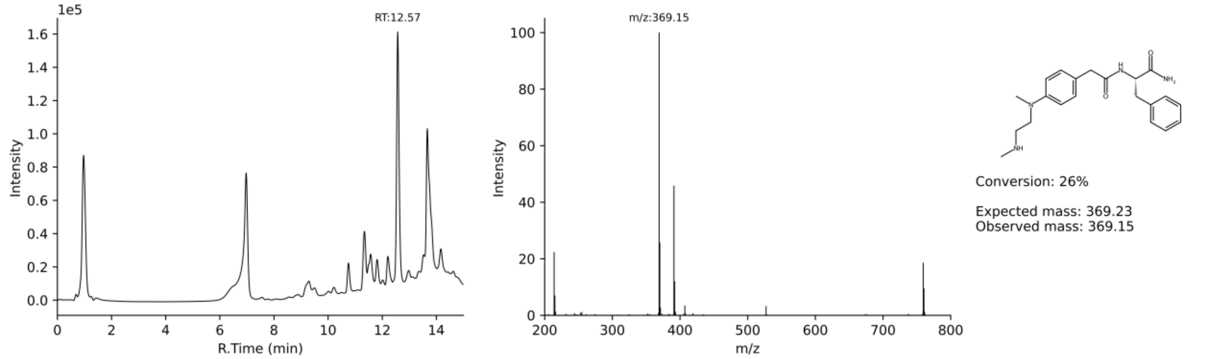

Fig. S158. LC-MS chromatogram of compound 9n.

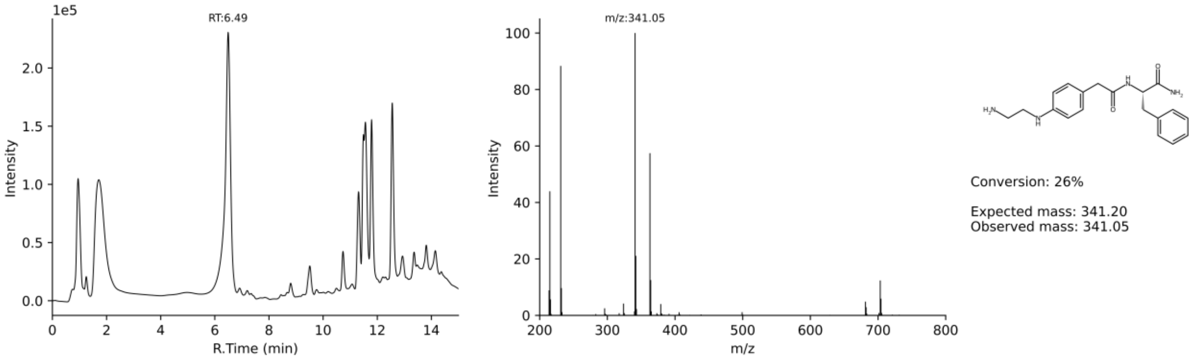

Fig. S159. LC-MS chromatogram of compound 9o.

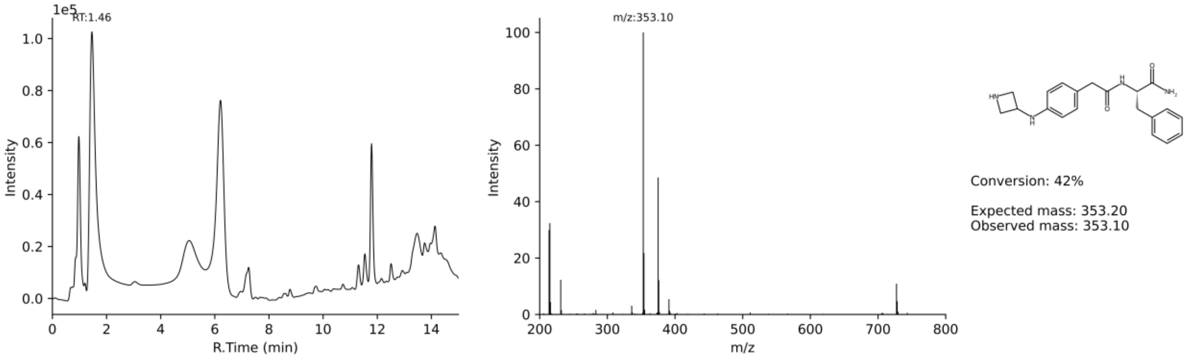

Fig. S160. LC-MS chromatogram of compound 9p.

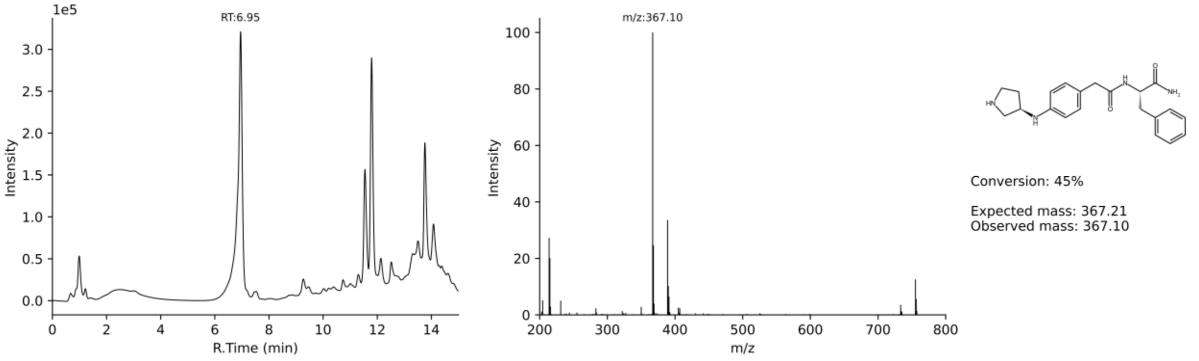

Fig. S161. LC-MS chromatogram of compound 9q.

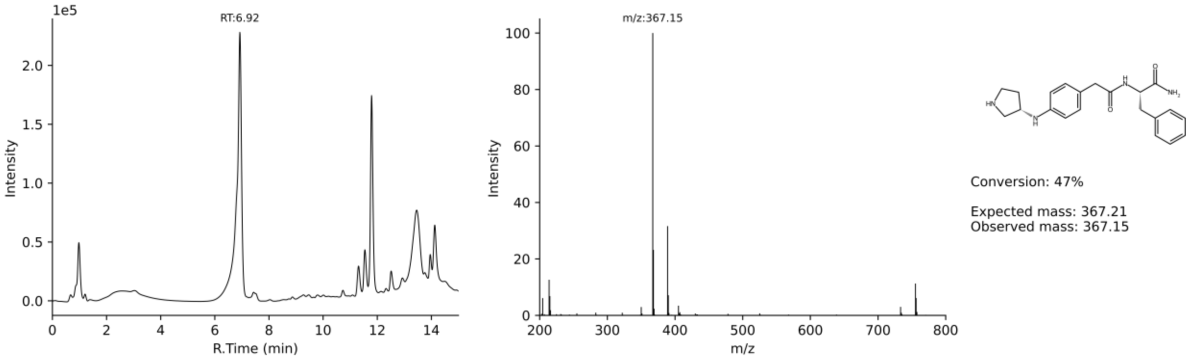

Fig. S162. LC-MS chromatogram of compound 9r.

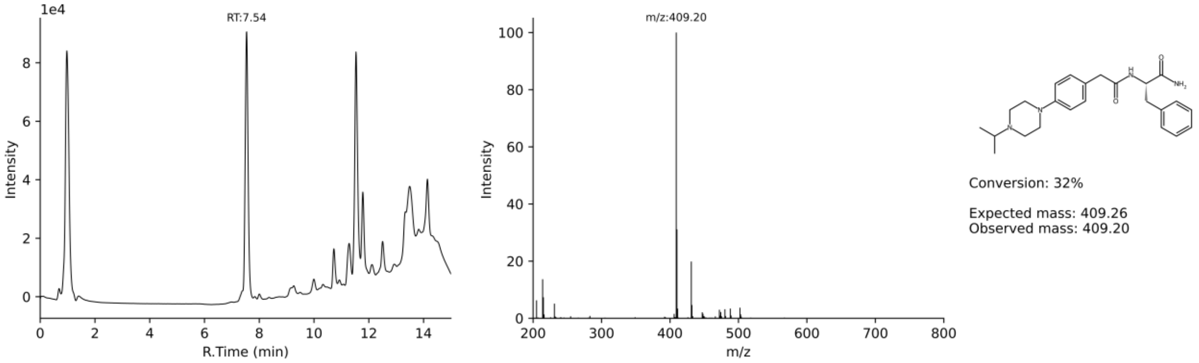

Fig. S163. LC-MS chromatogram of compound 9s.

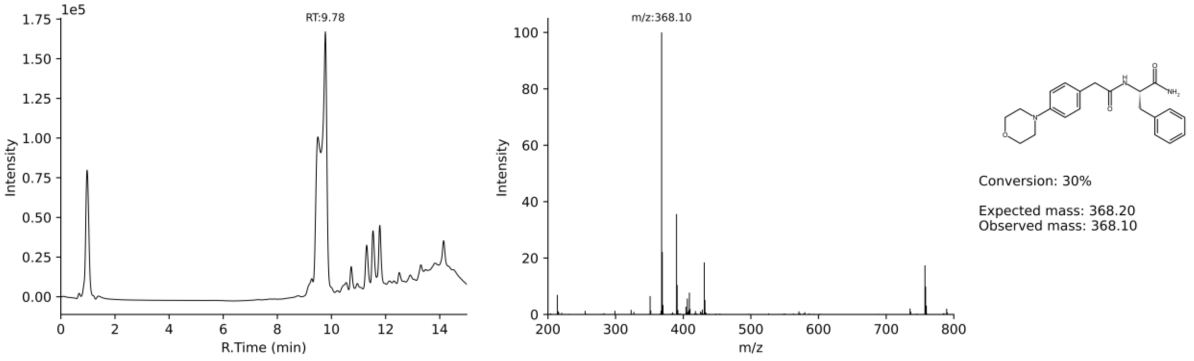

Fig. S164. LC-MS chromatogram of compound 9t.

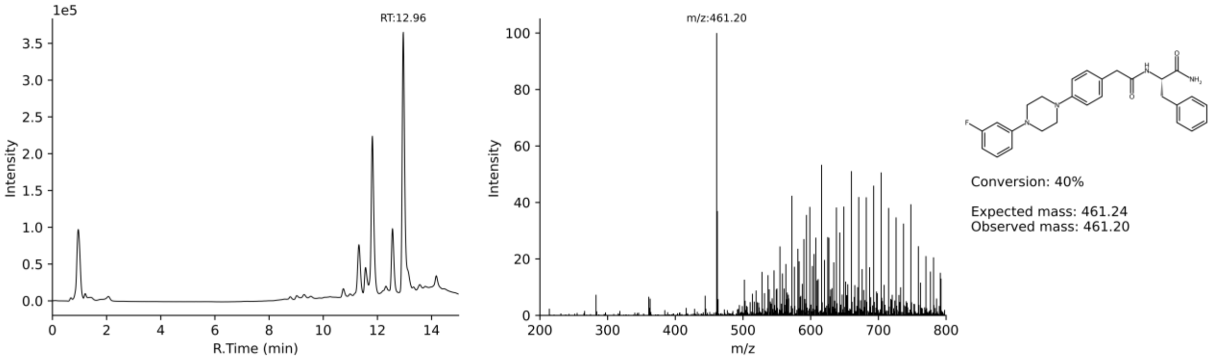

Fig. S165. LC-MS chromatogram of compound 9u.

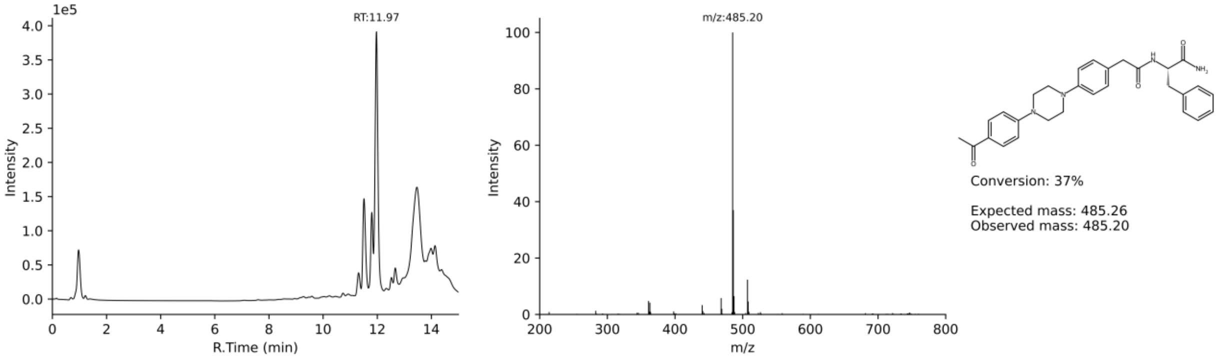

Fig. S166. LC-MS chromatogram of compound 9v.

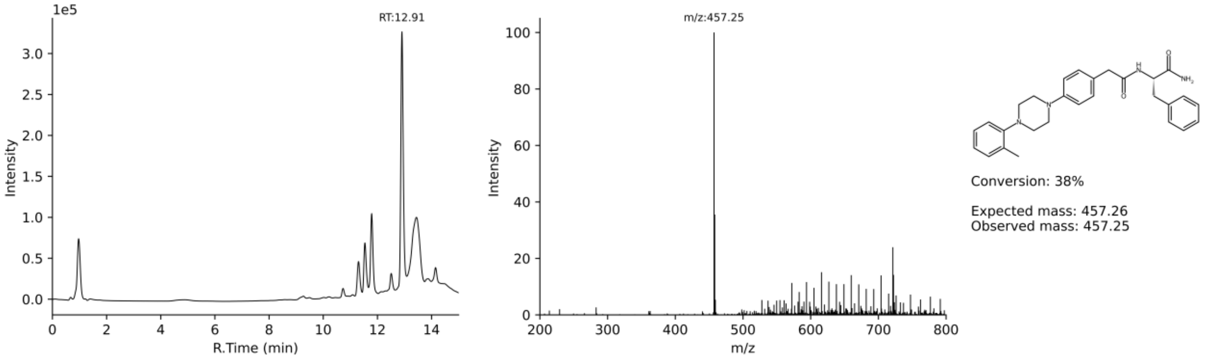

Fig. S167. LC-MS chromatogram of compound 9w.

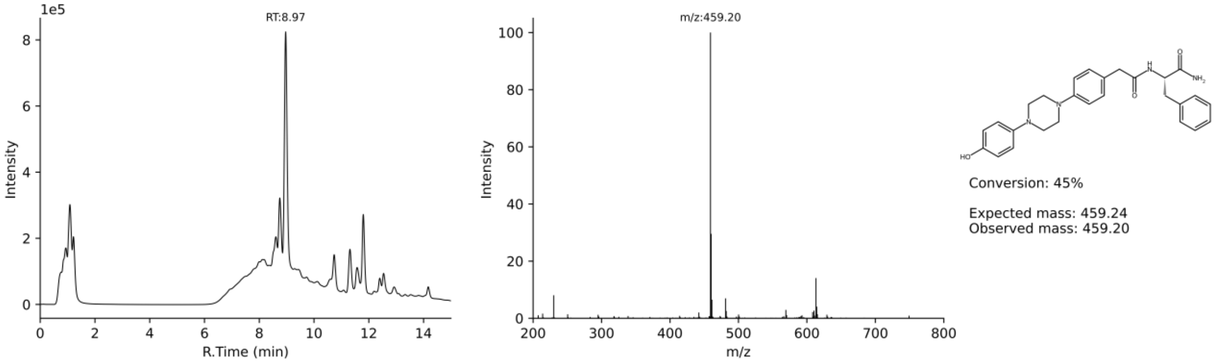

Fig. S168. LC-MS chromatogram of compound 9x.

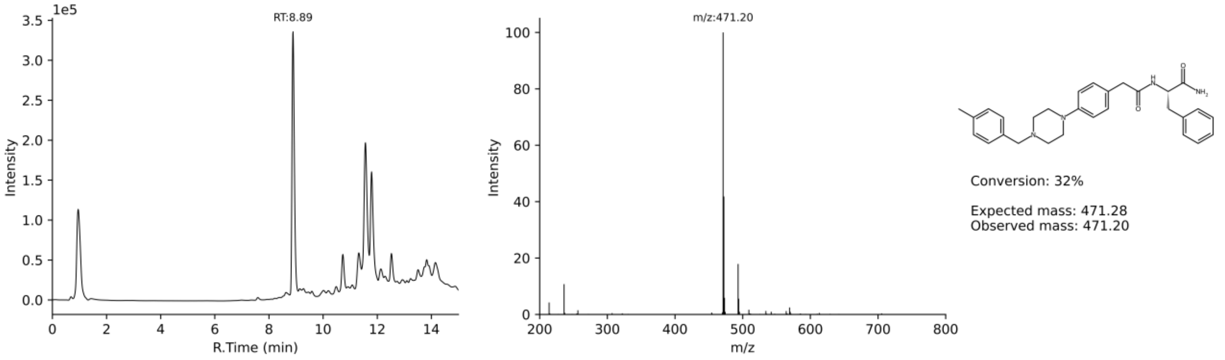

Fig. S169. LC-MS chromatogram of compound 9y.

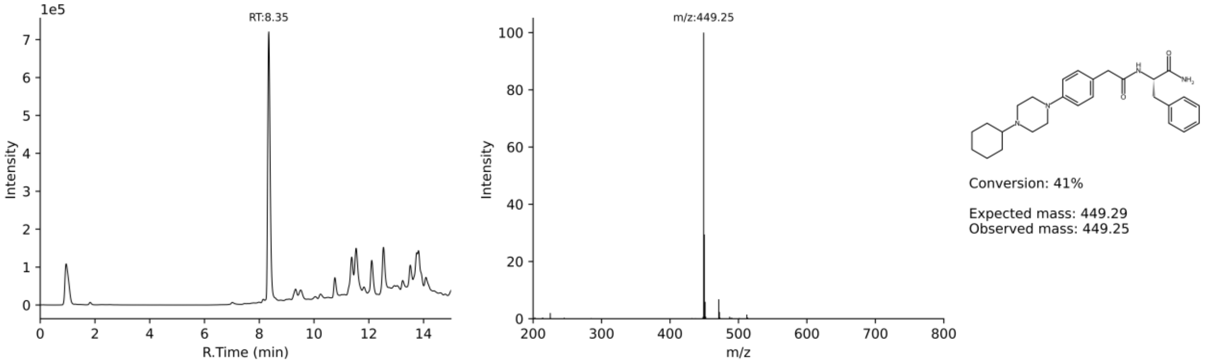

Fig. S170. LC-MS chromatogram of compound 9z.

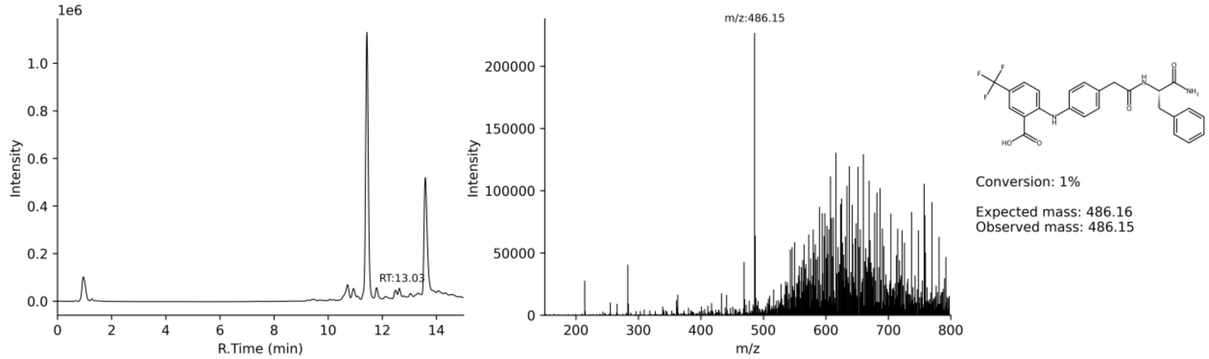

Fig. S171. LC-MS chromatogram of compound 10a.

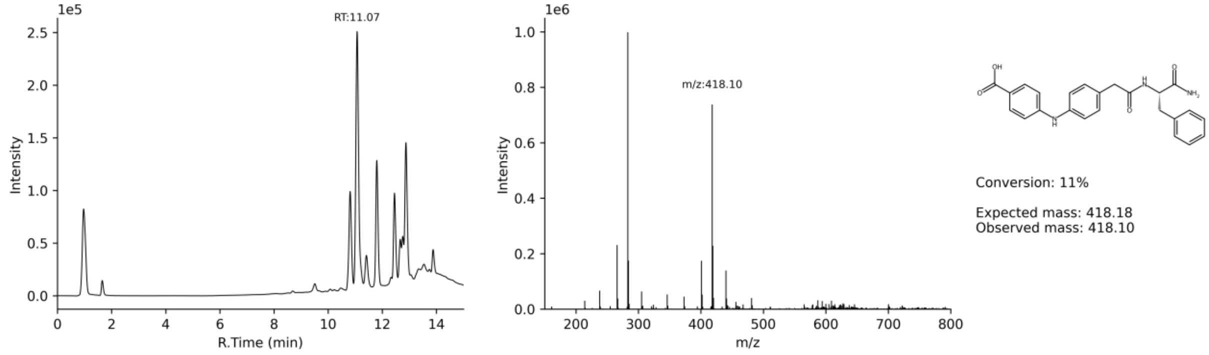

Fig. S172. LC-MS chromatogram of compound 10b.

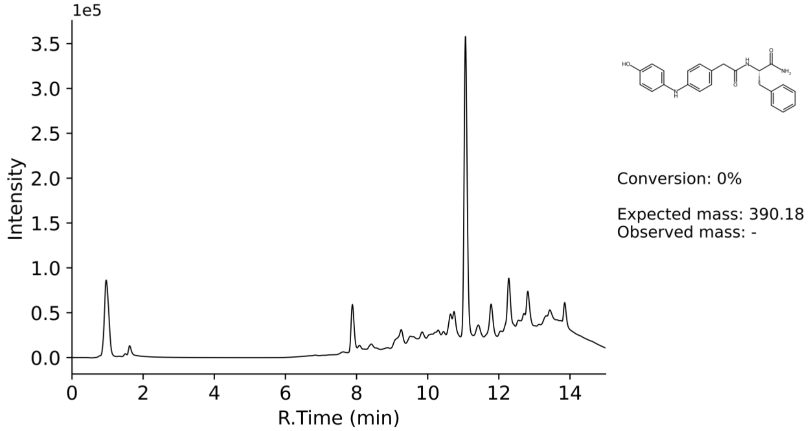

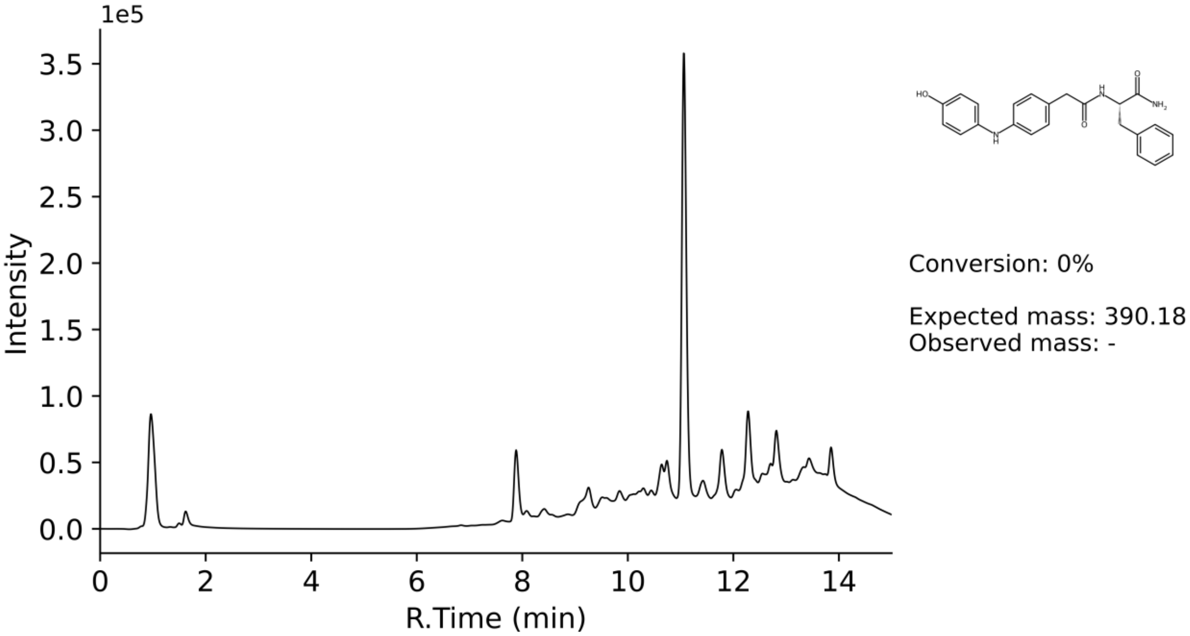

Fig. S173. LC-MS chromatogram of compound 10c.

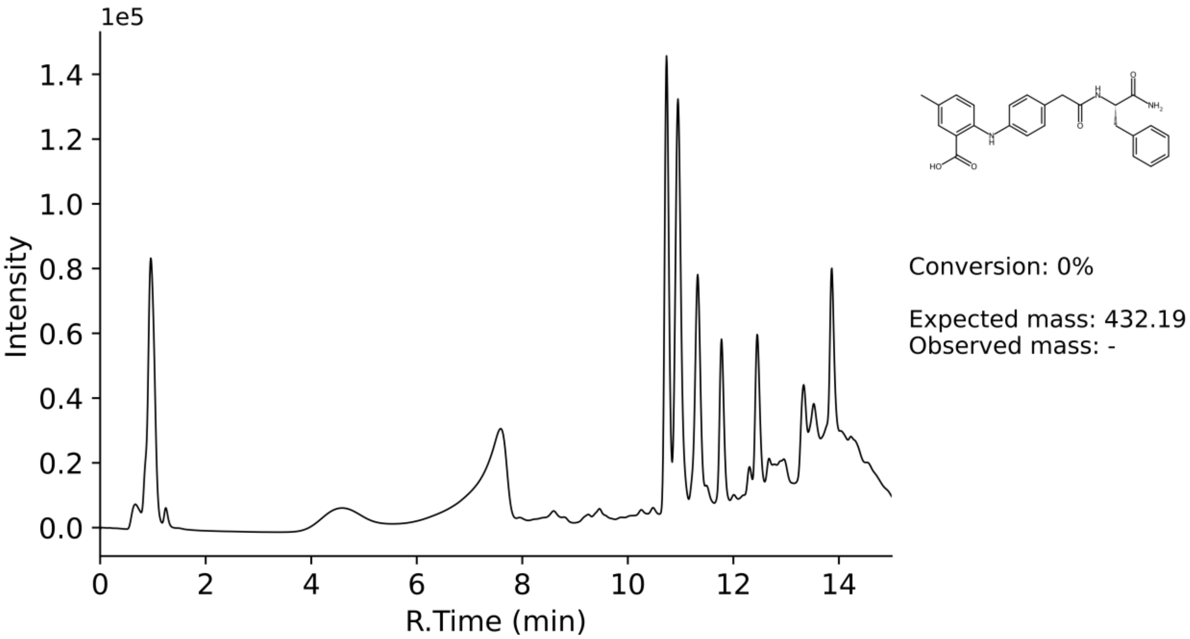

Fig. S174. LC-MS chromatogram of compound 10d.

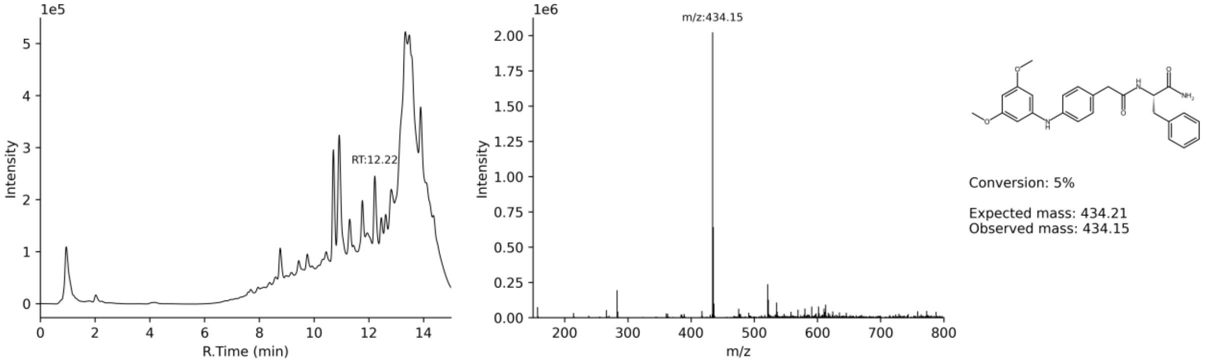

Fig. S175. LC-MS chromatogram of compound 10e.

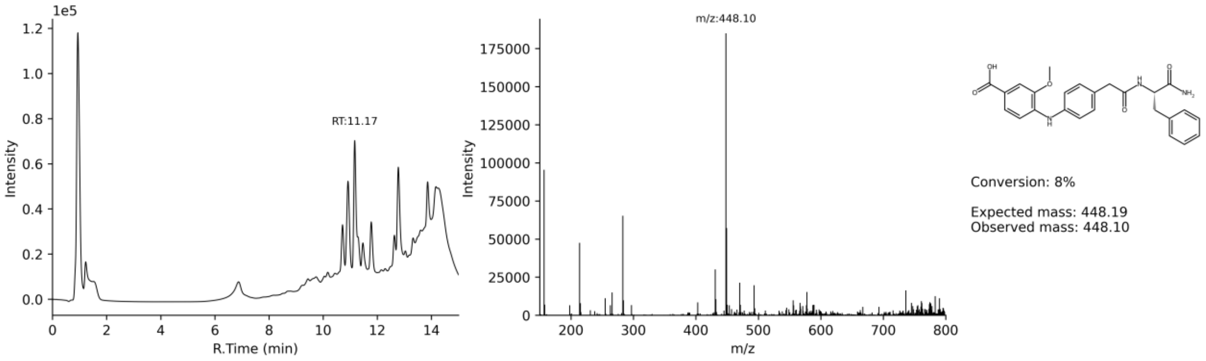

Fig. S176. LC-MS chromatogram of compound 10f.

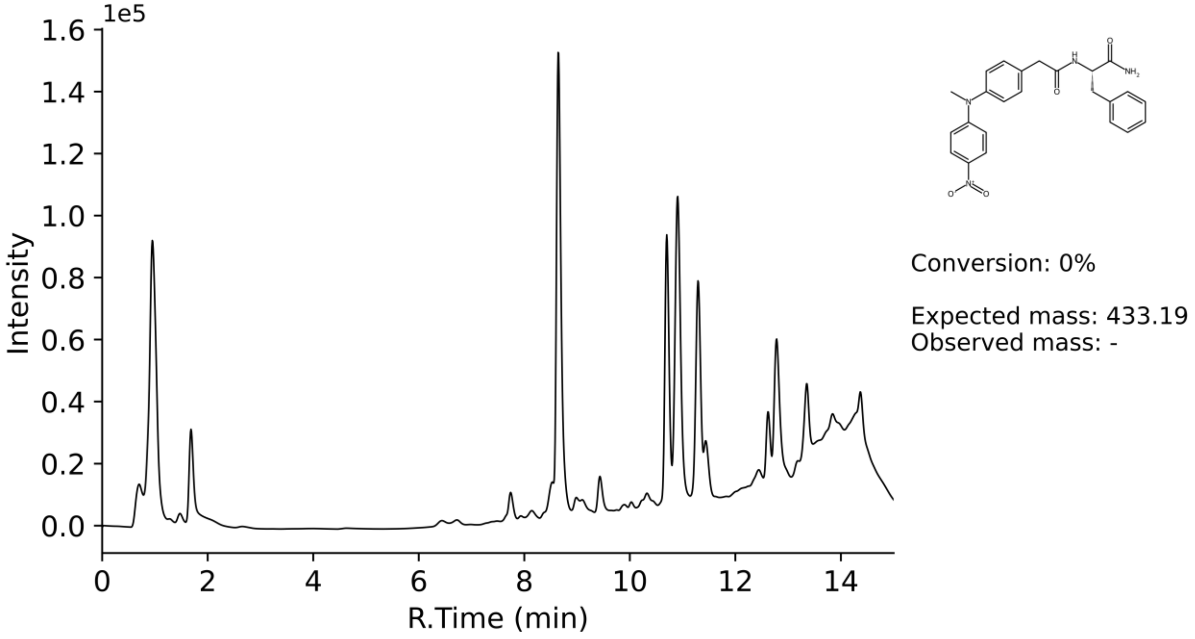

Fig. S177. LC-MS chromatogram of compound 10g.

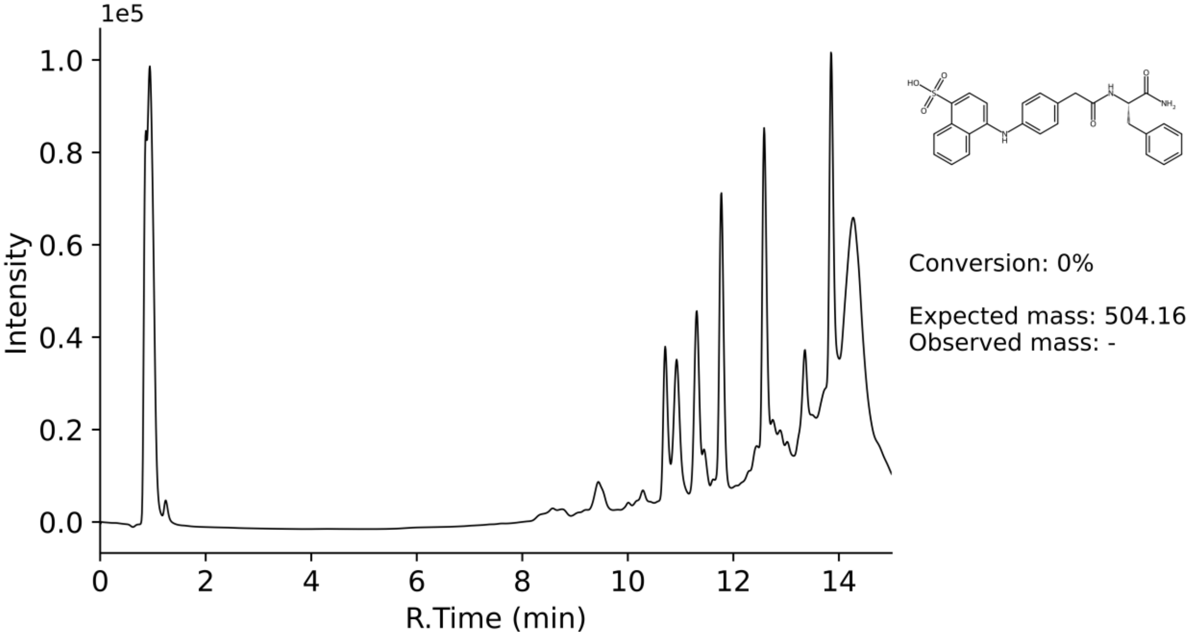

Fig. S178. LC-MS chromatogram of compound 10h.

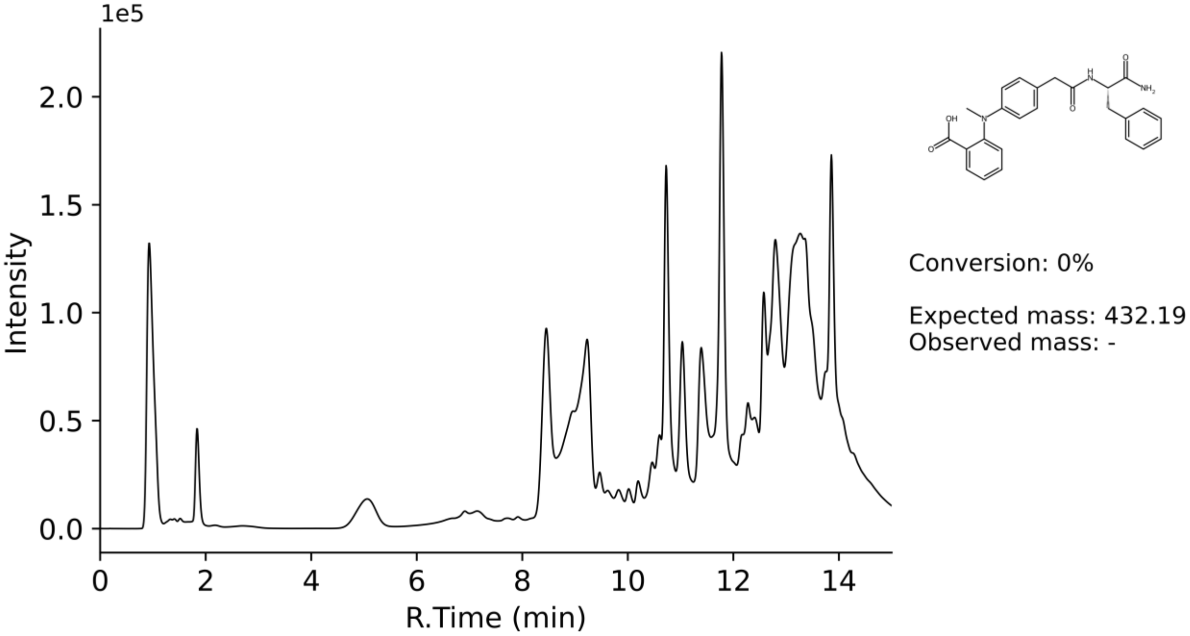

Fig. S179. LC-MS chromatogram of compound 10i.

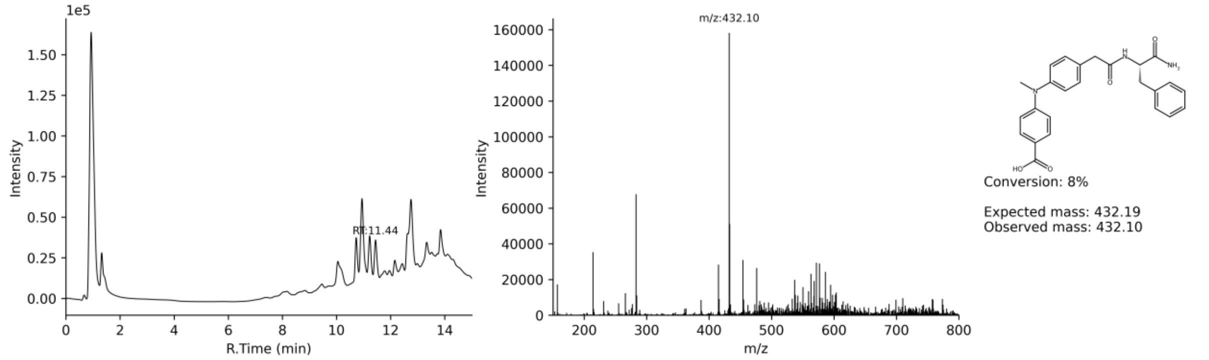

Fig. S180. LC-MS chromatogram of compound 10j.

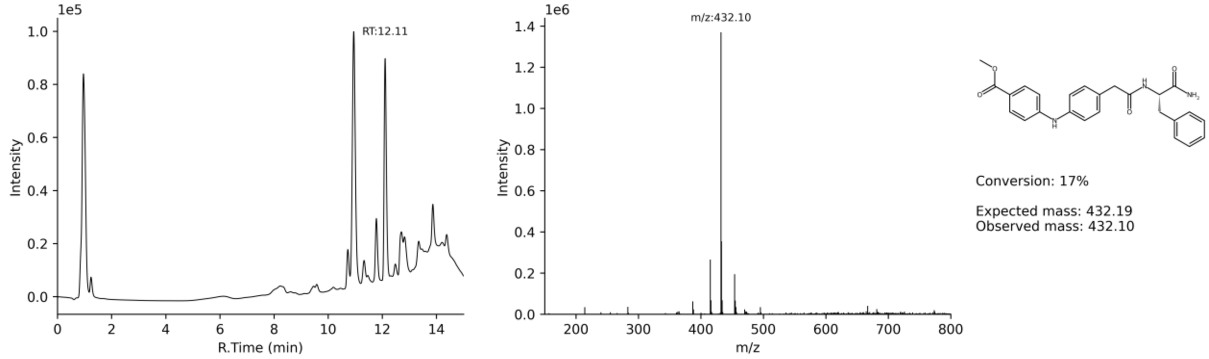

Fig. S181. LC-MS chromatogram of compound 10k.

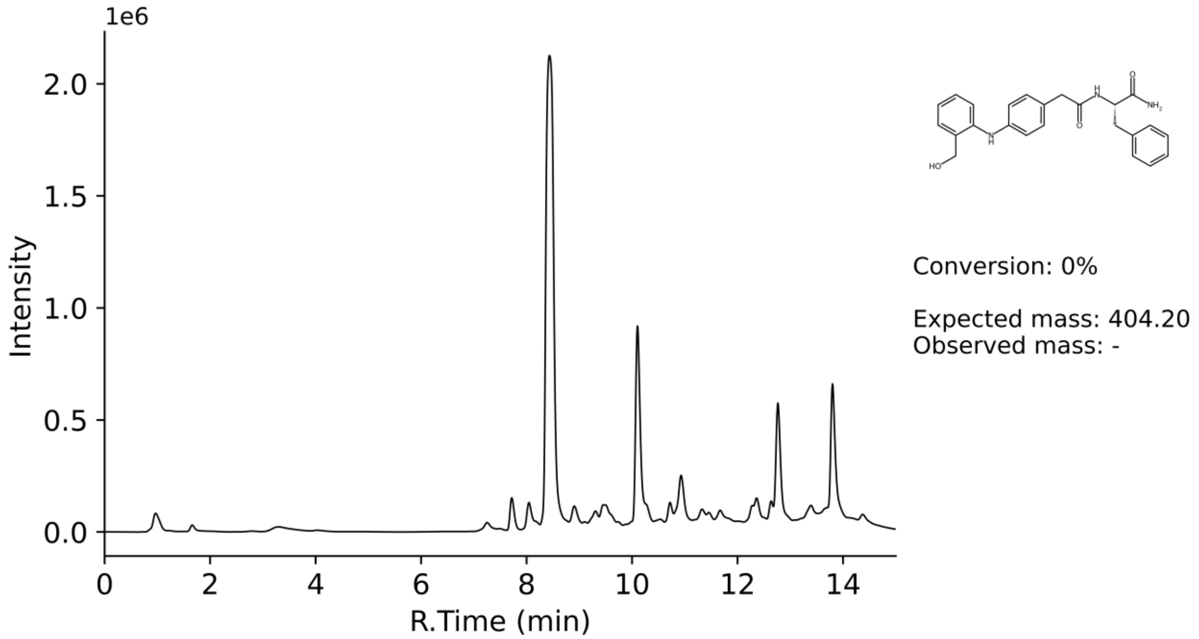

Fig. S182. LC-MS chromatogram of compound 10l.

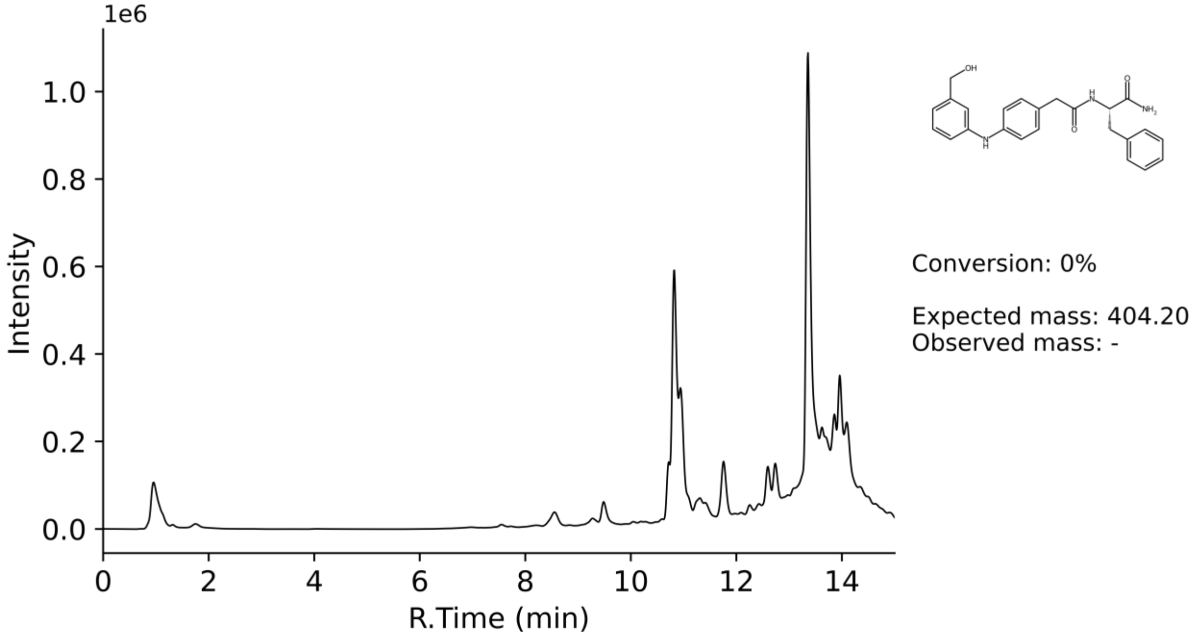

Fig. S183. LC-MS chromatogram of compound 10m.

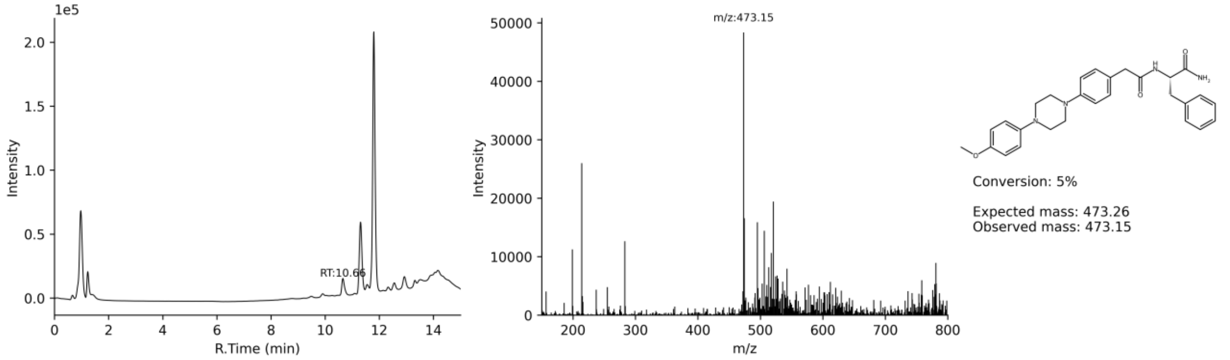

Fig. S184. LC-MS chromatogram of compound 10n.

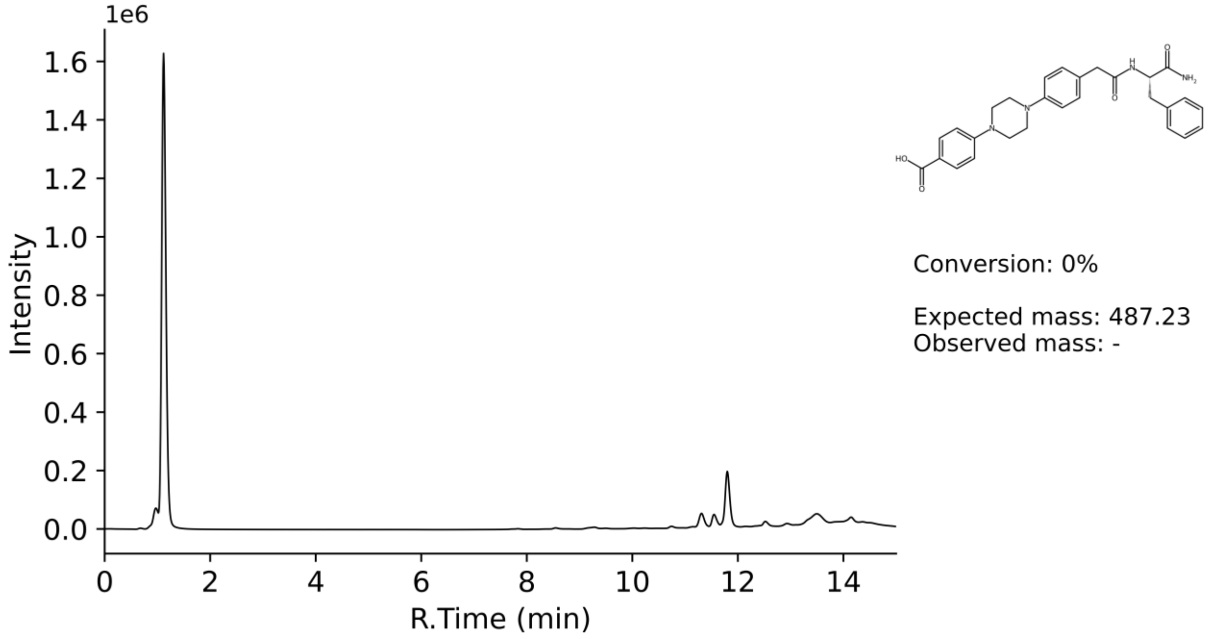

Fig. S185. LC-MS chromatogram of compound 10o.

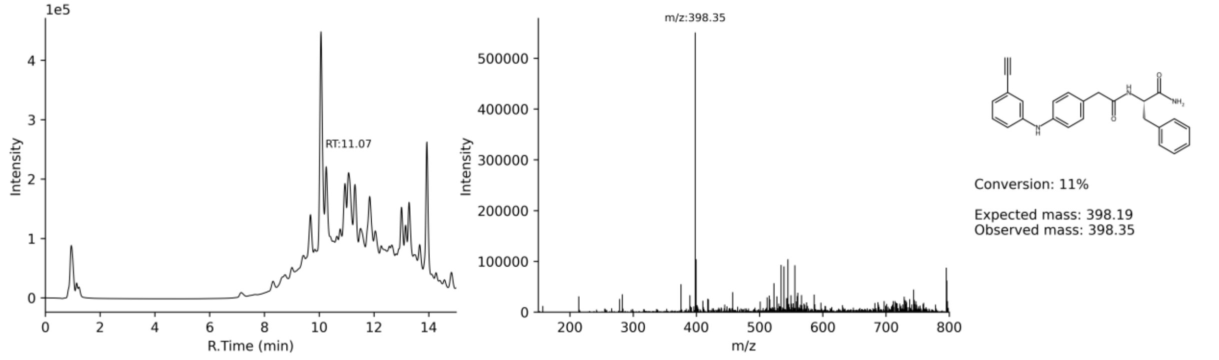

Fig. S186. LC-MS chromatogram of compound 10p.

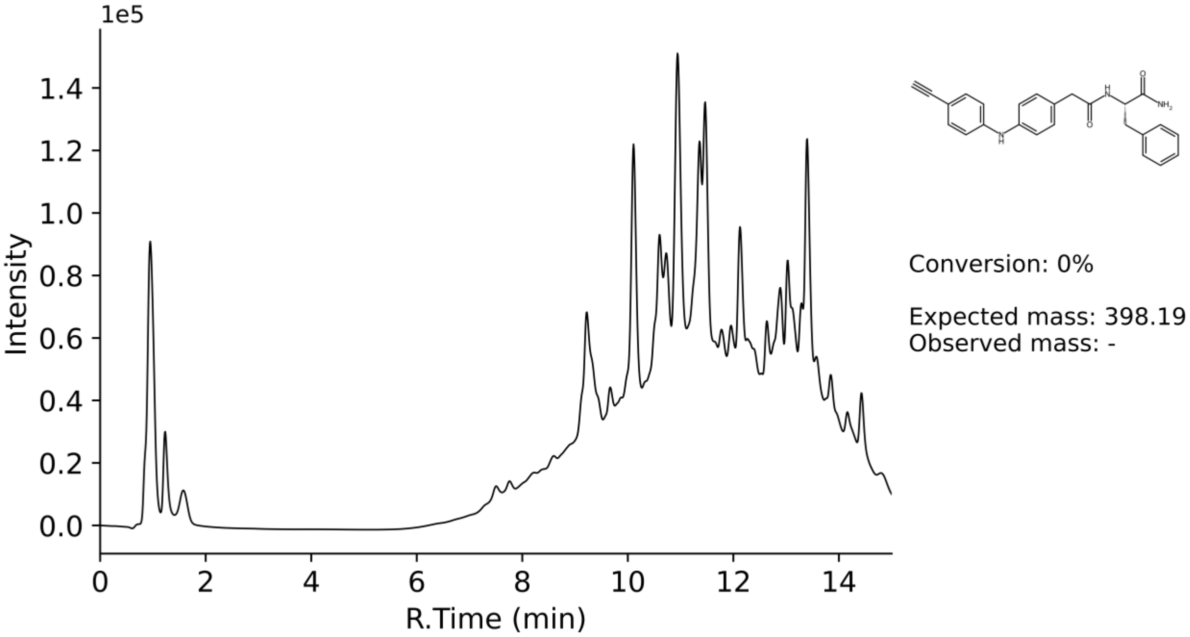

Fig. S187. LC-MS chromatogram of compound 10q.

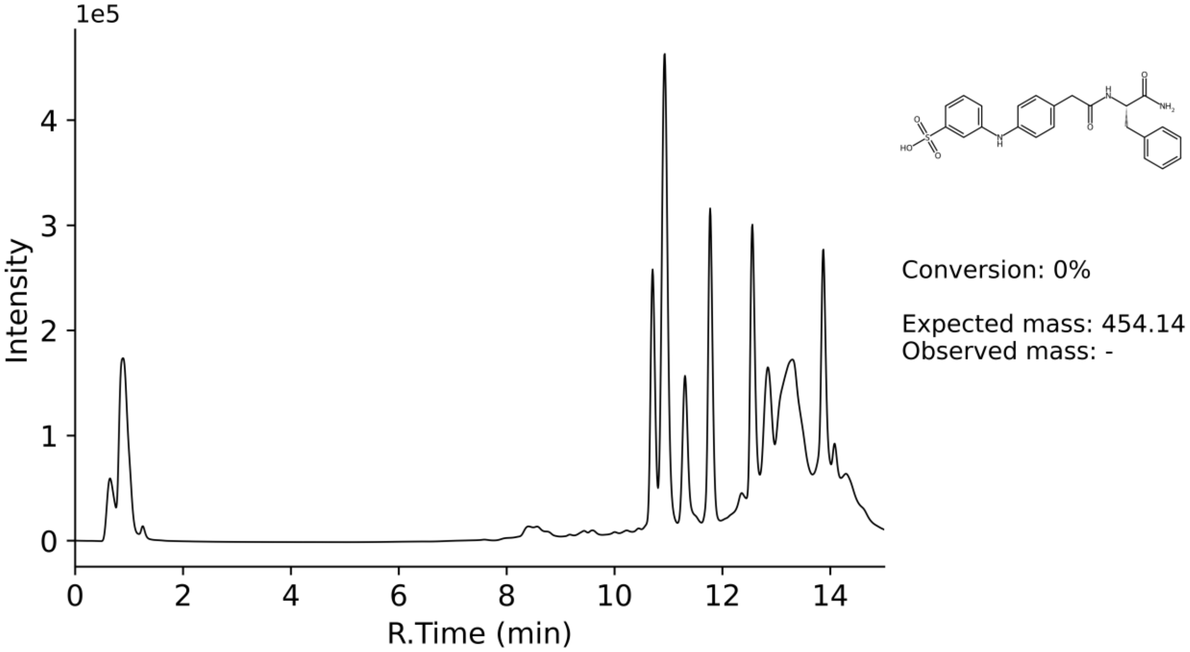

Fig. S188. LC-MS chromatogram of compound 10r.

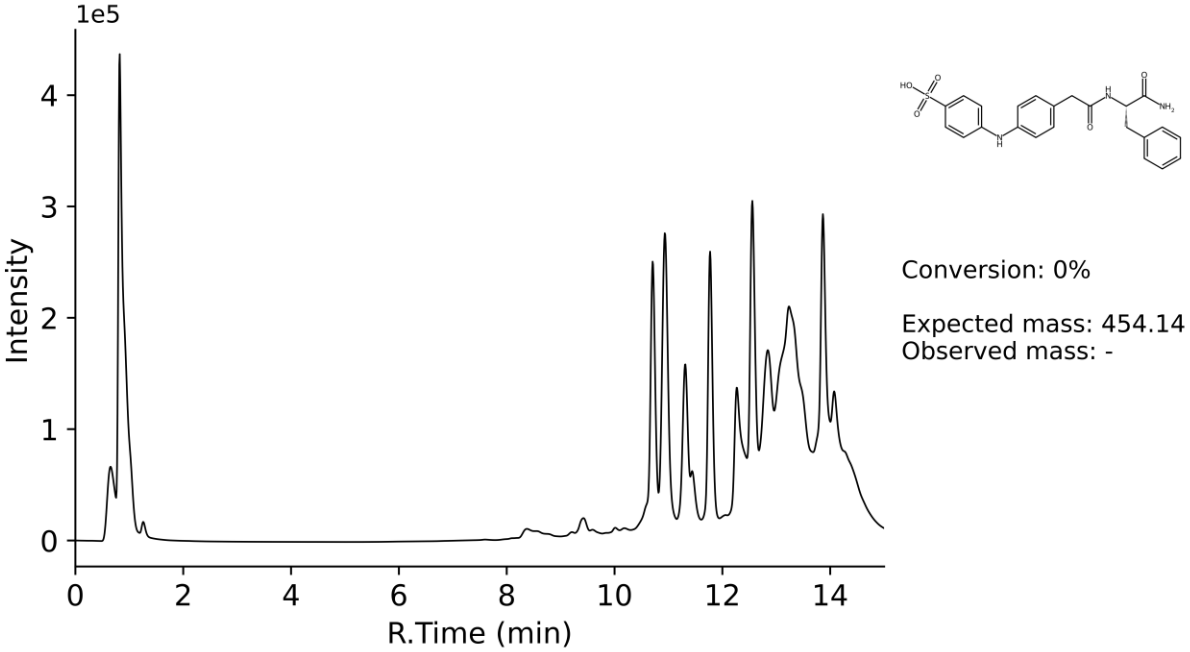

Fig. S189. LC-MS chromatogram of compound 10s.

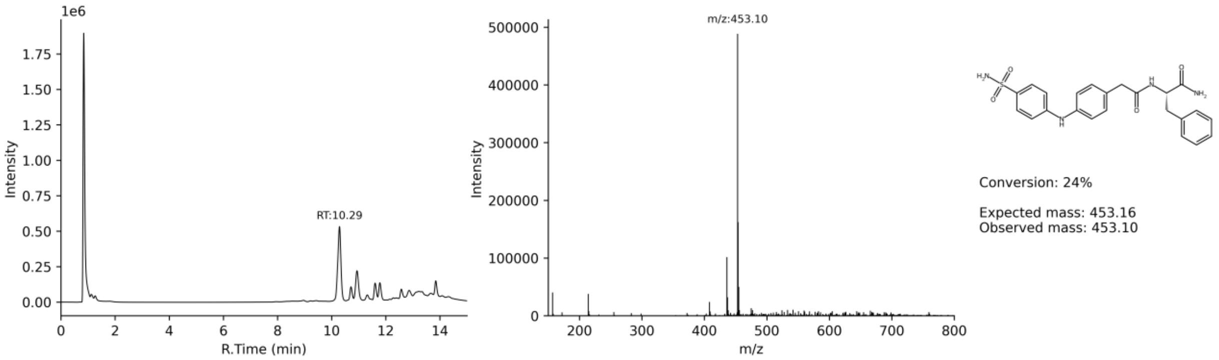

Fig. S190. LC-MS chromatogram of compound 10t.

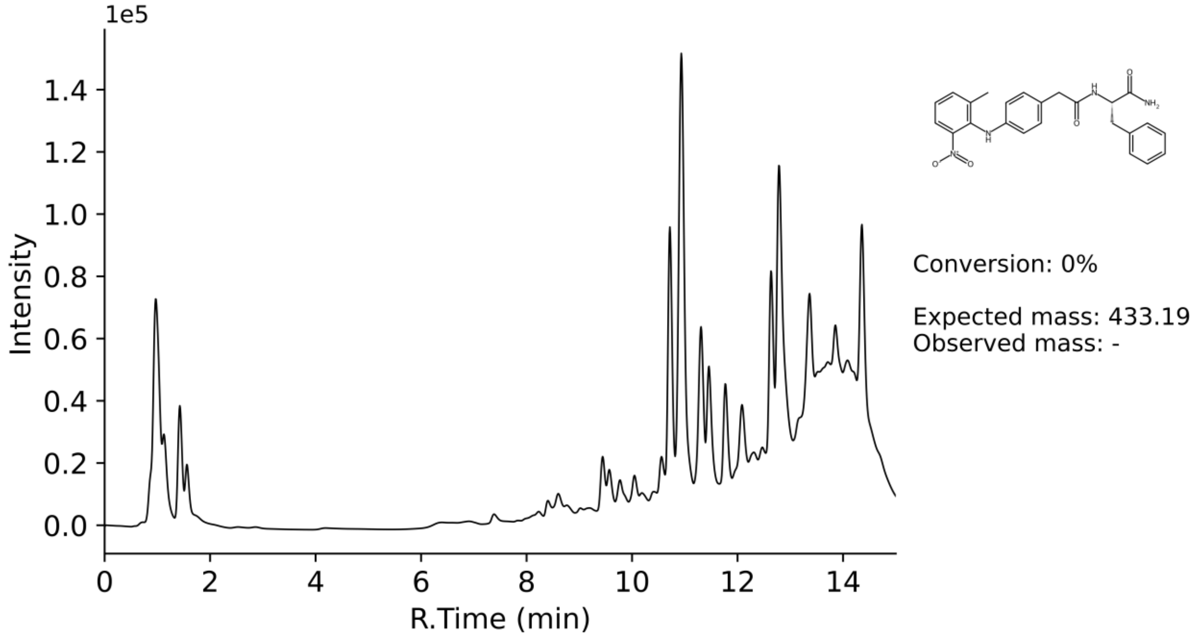

Fig. S191. LC-MS chromatogram of compound 10u.

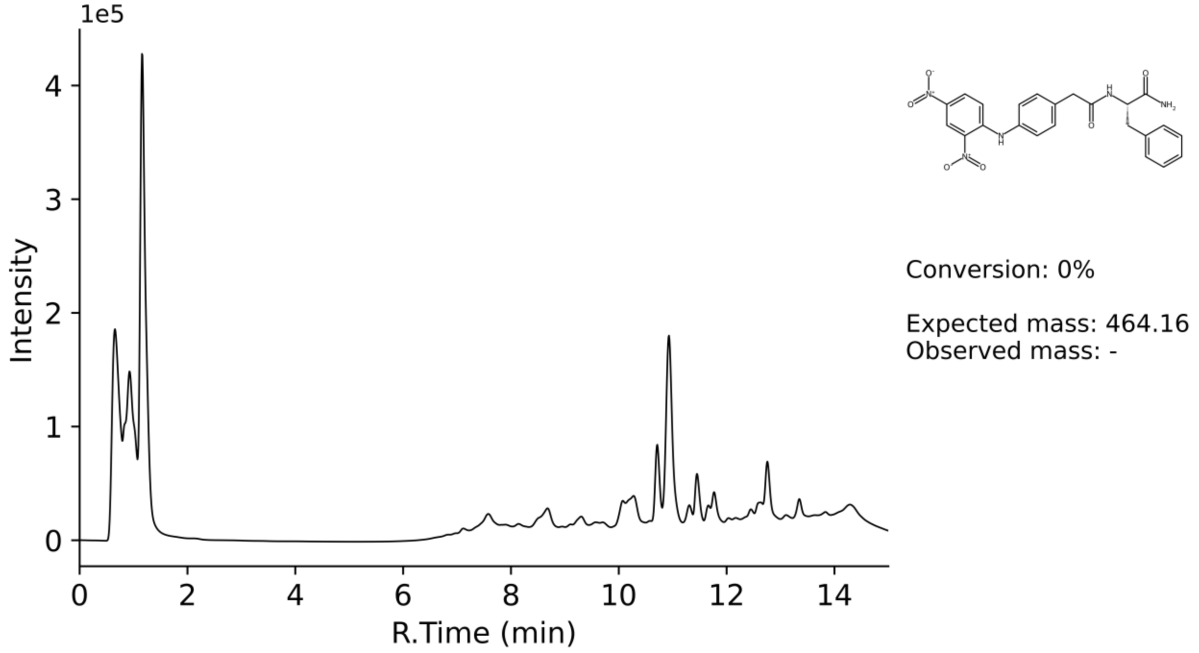

Fig. S192. LC-MS chromatogram of compound 10v.

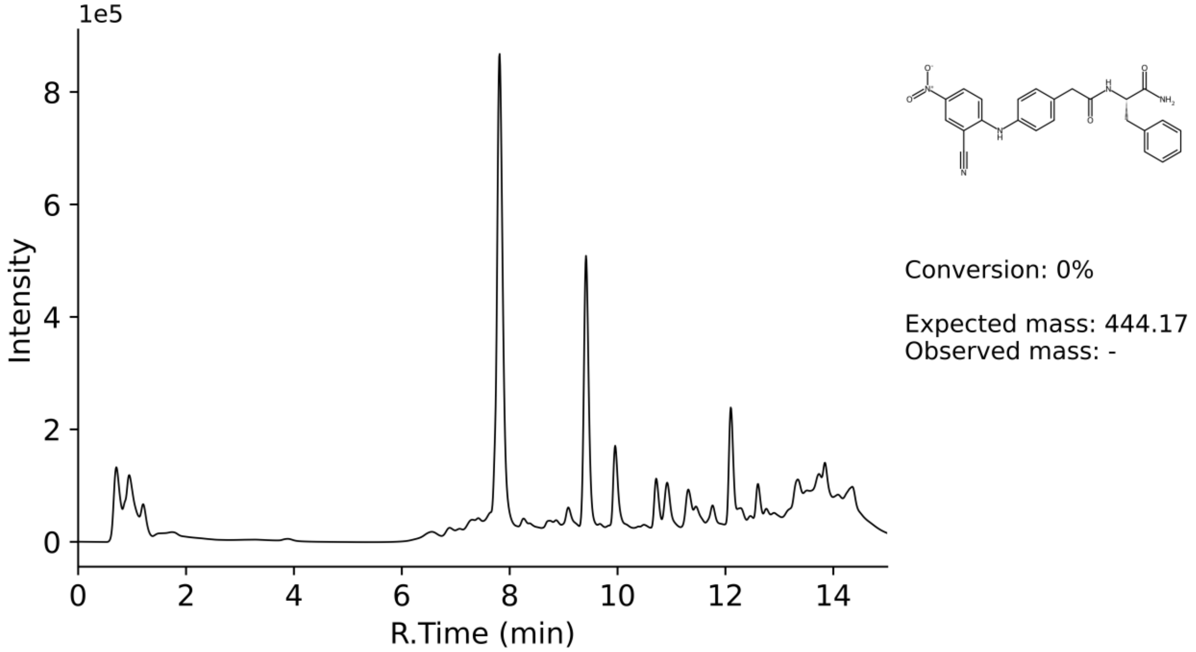

Fig. S193. LC-MS chromatogram of compound 10w.

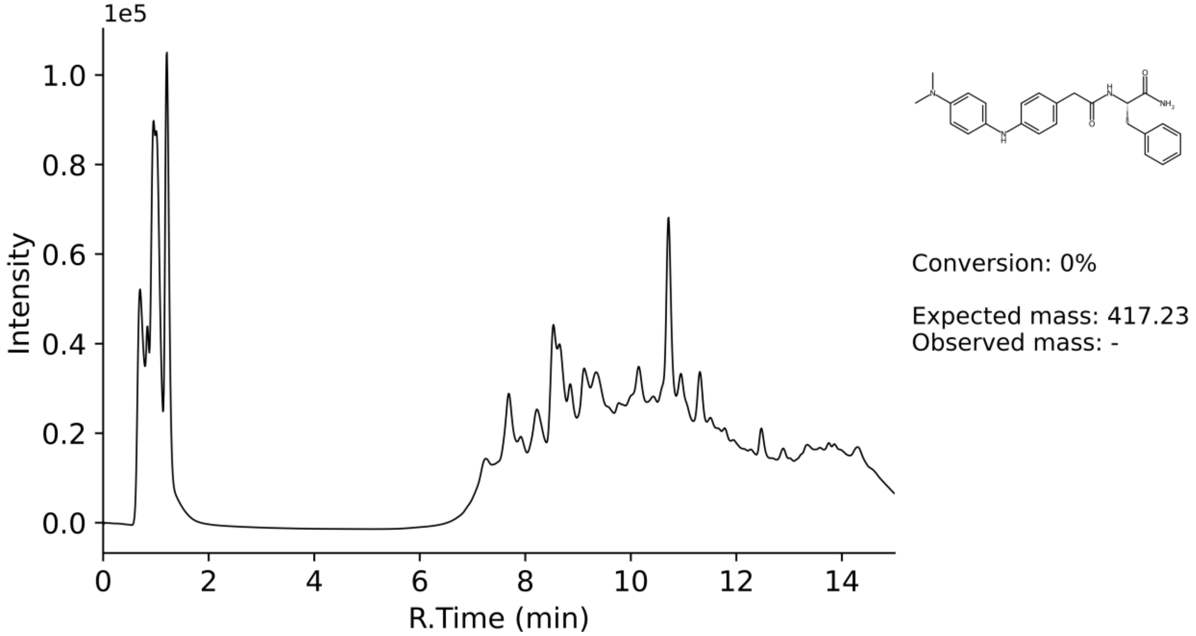

Fig. S194. LC-MS chromatogram of compound 10x.

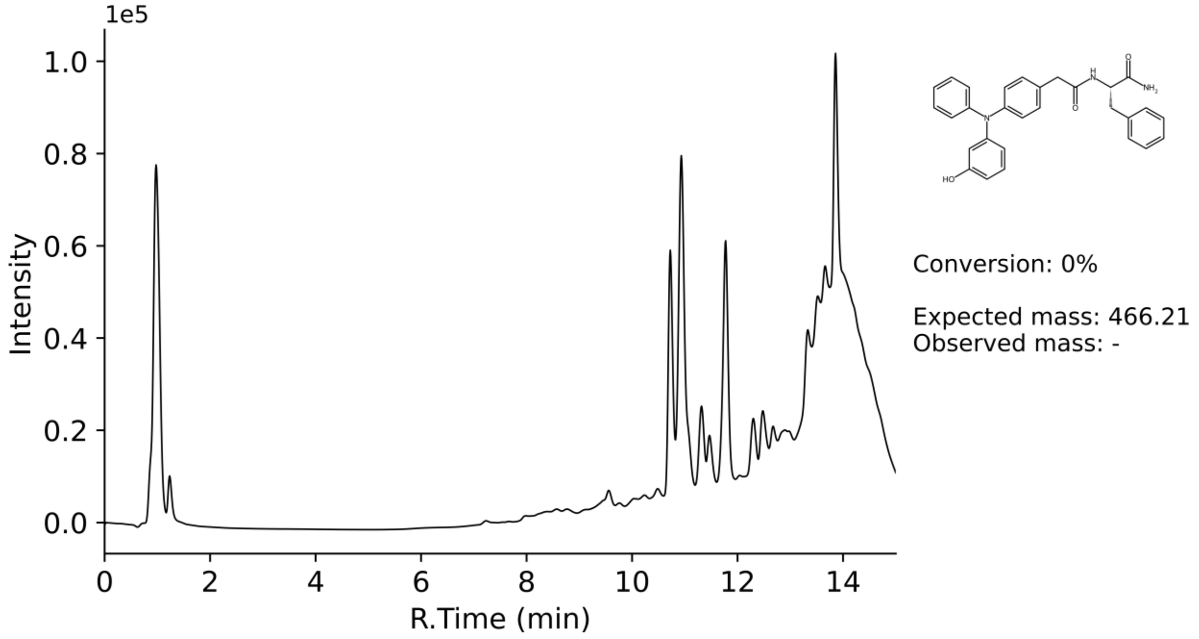

Fig. S195. LC-MS chromatogram of compound 10y.

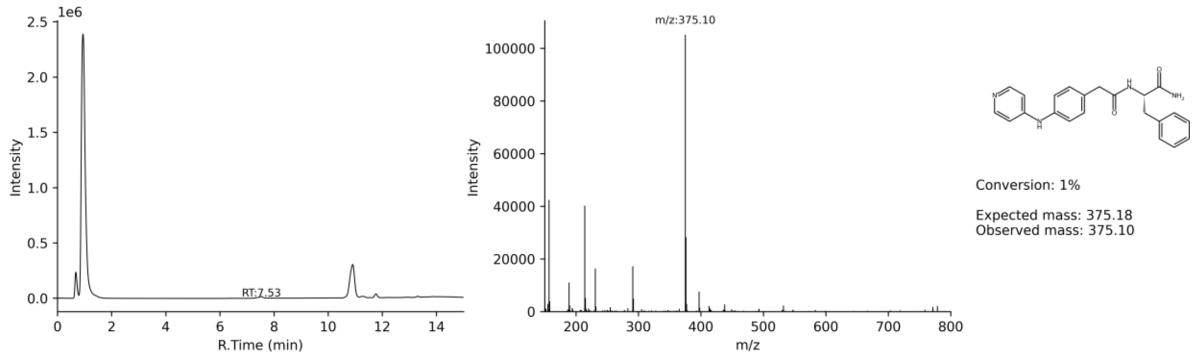

Fig. S196. LC-MS chromatogram of compound 10z.

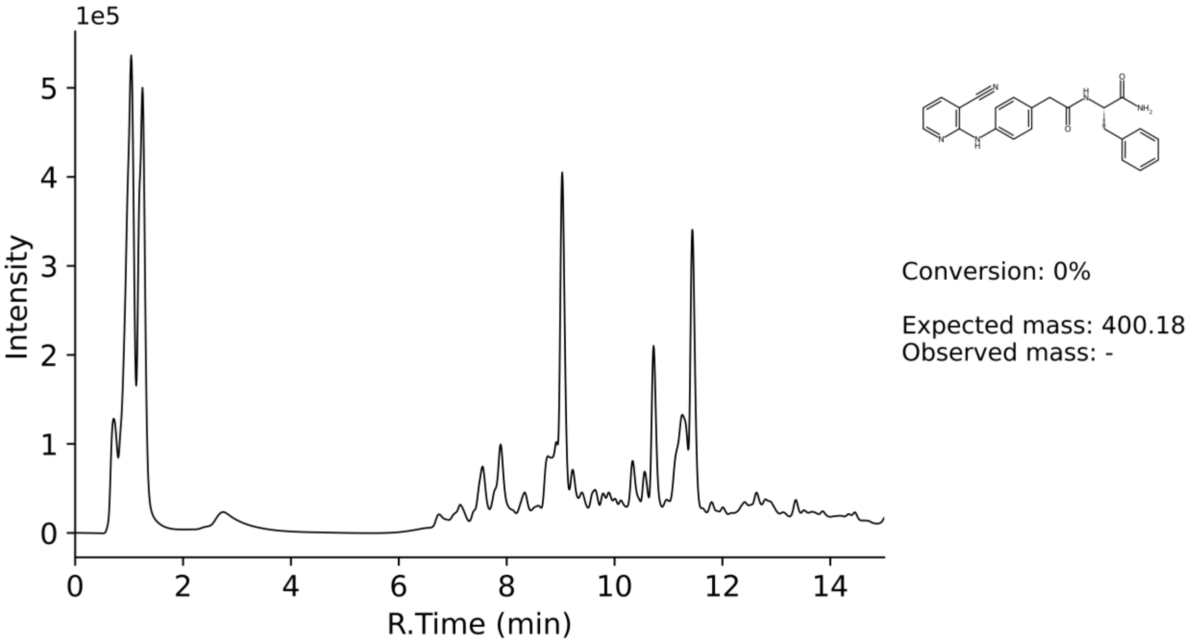

Fig. S197. LC-MS chromatogram of compound 10aa.

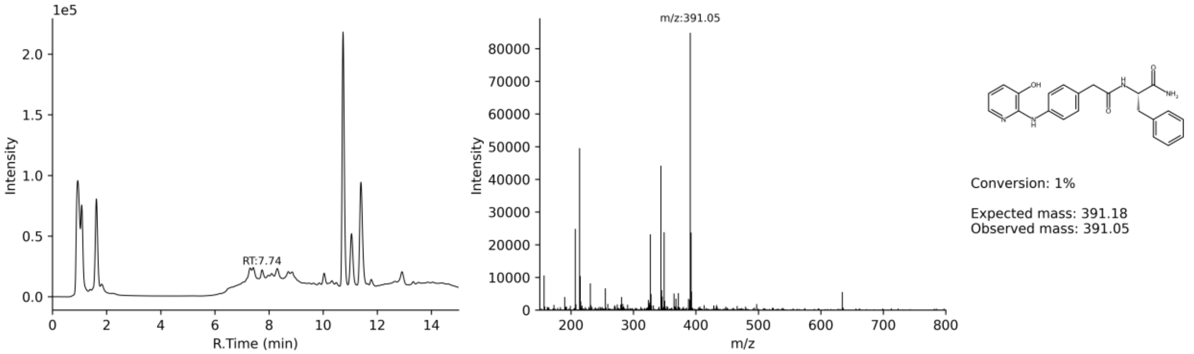

Fig. S198. LC-MS chromatogram of compound 10ab.

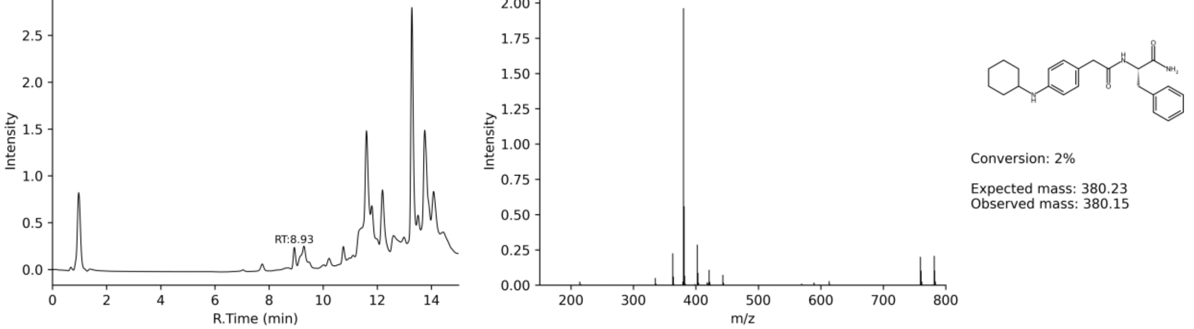

Fig. S199. LC-MS chromatogram of compound 10ac.

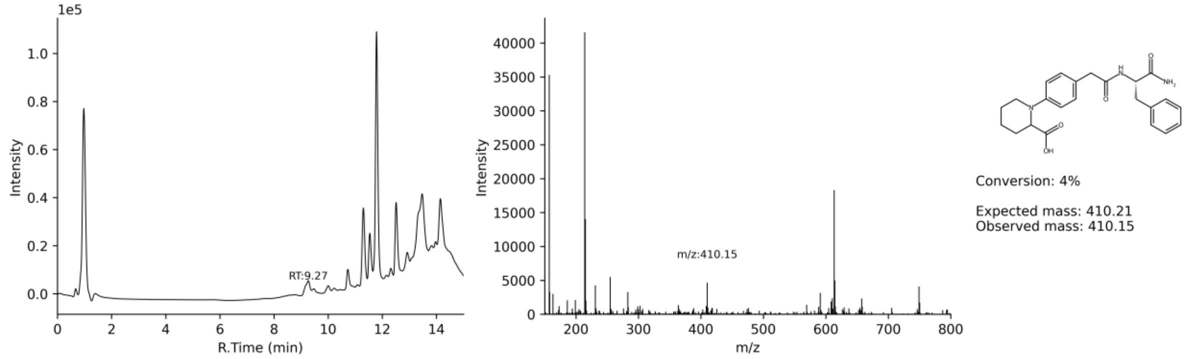

Fig. S200. LC-MS chromatogram of compound 10ad.

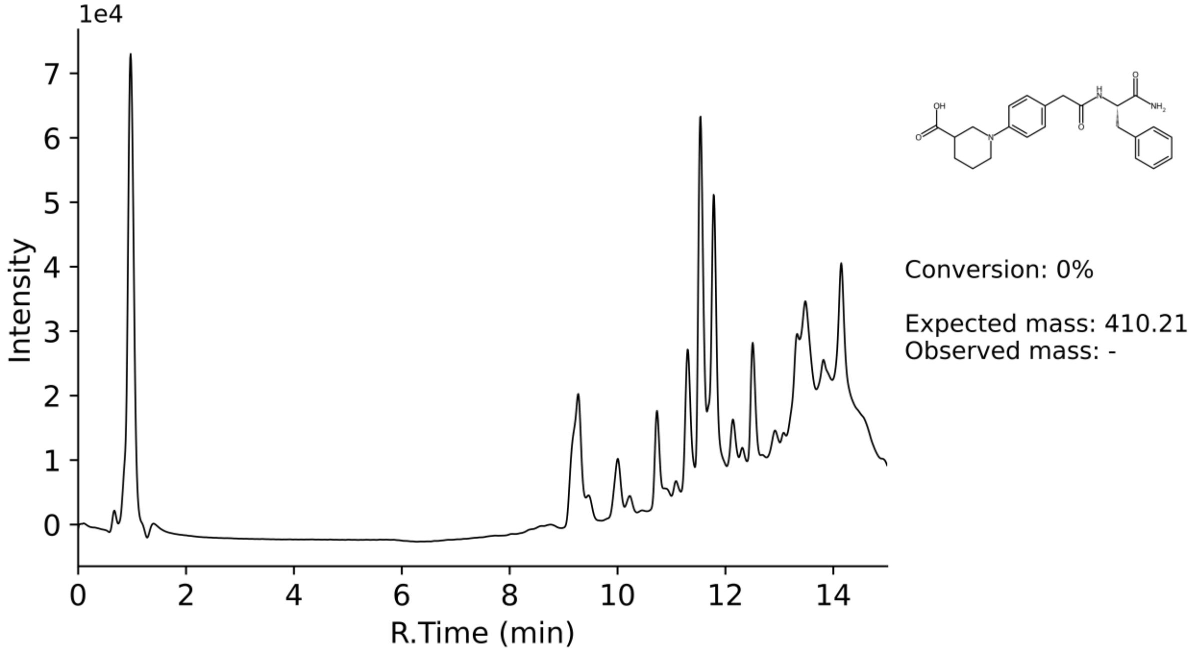

Fig. S201. LC-MS chromatogram of compound 10ae.

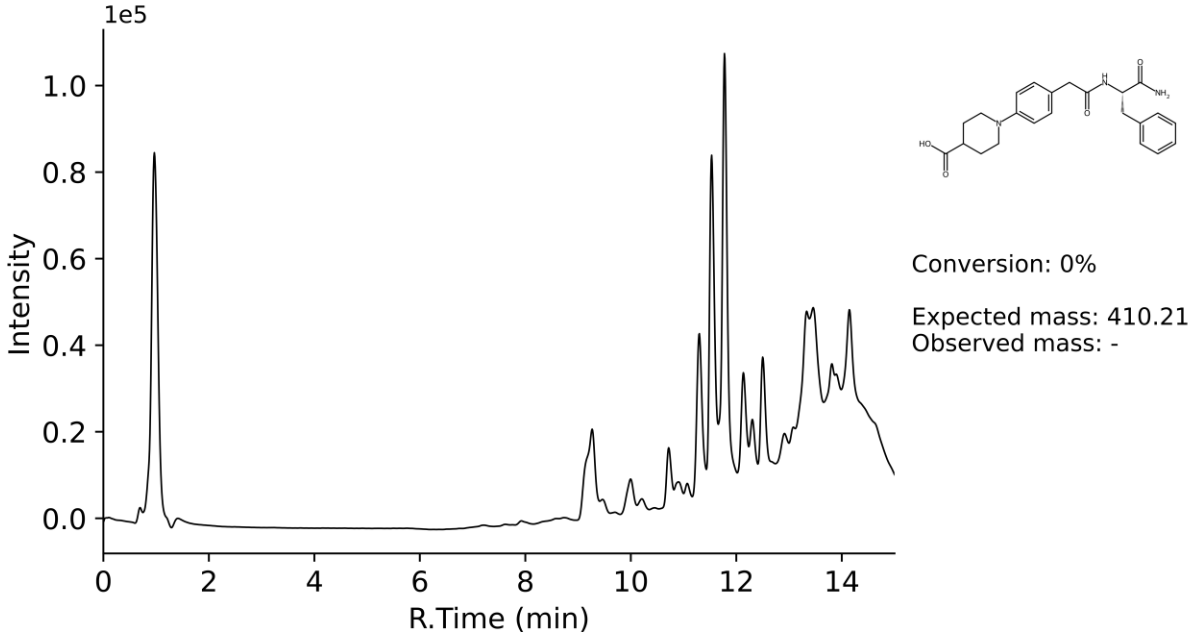

Fig. S202. LC-MS chromatogram of compound 10af.

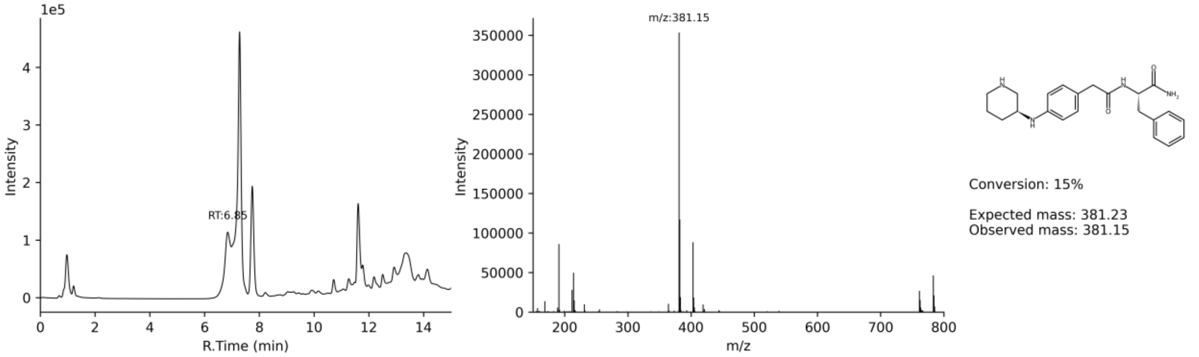

Fig. S203. LC-MS chromatogram of compound 10ag.

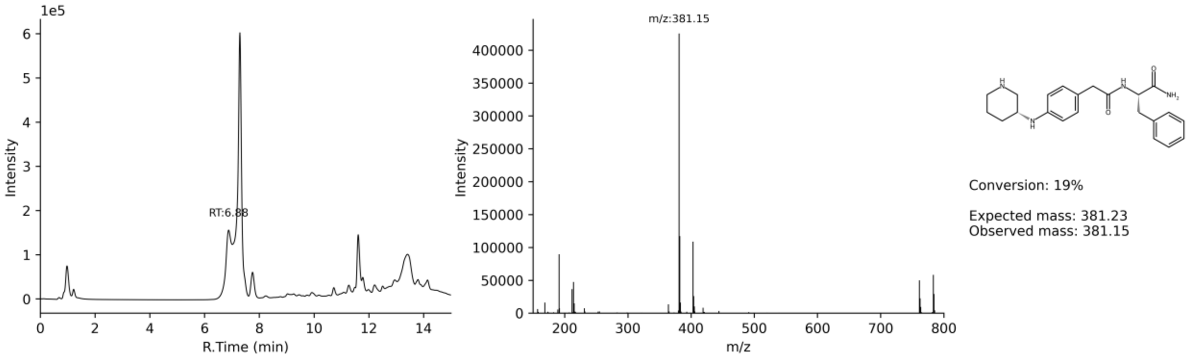

Fig. S204. LC-MS chromatogram of compound 10ah.

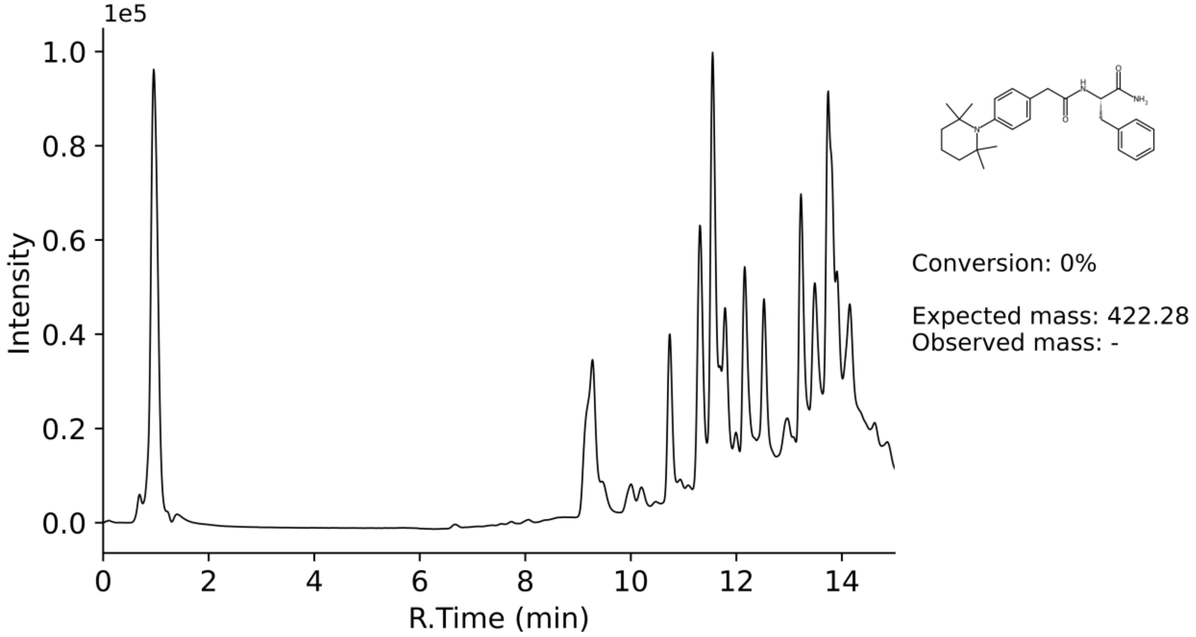

Fig. S205. LC-MS chromatogram of compound 10ai.

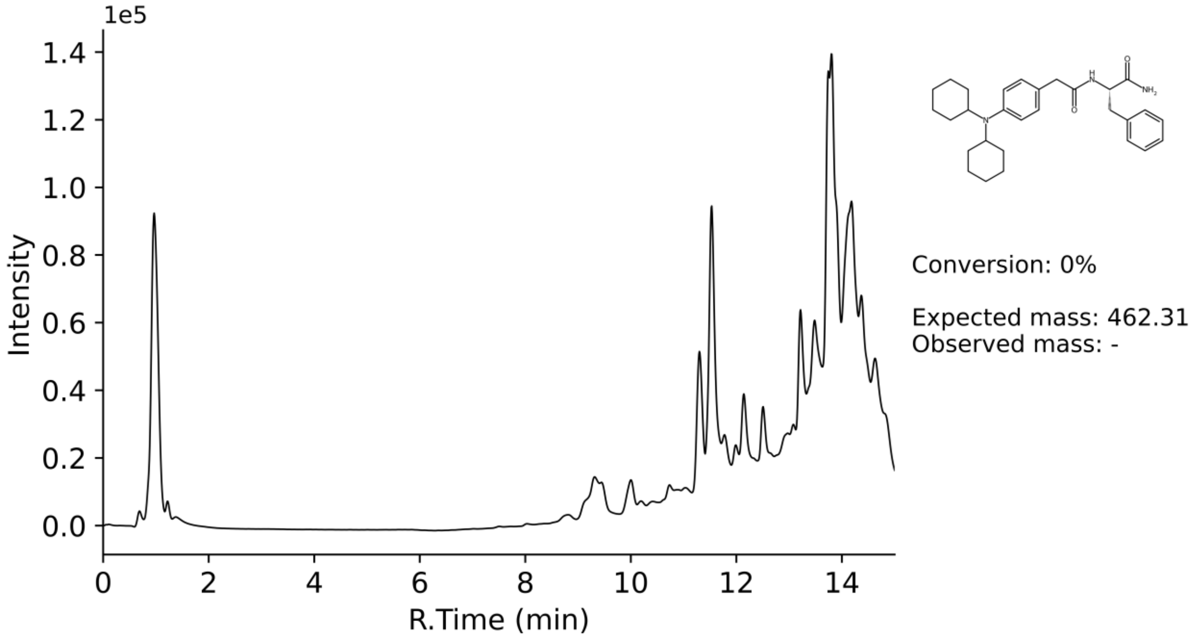

Fig. S206. LC-MS chromatogram of compound 10aj.

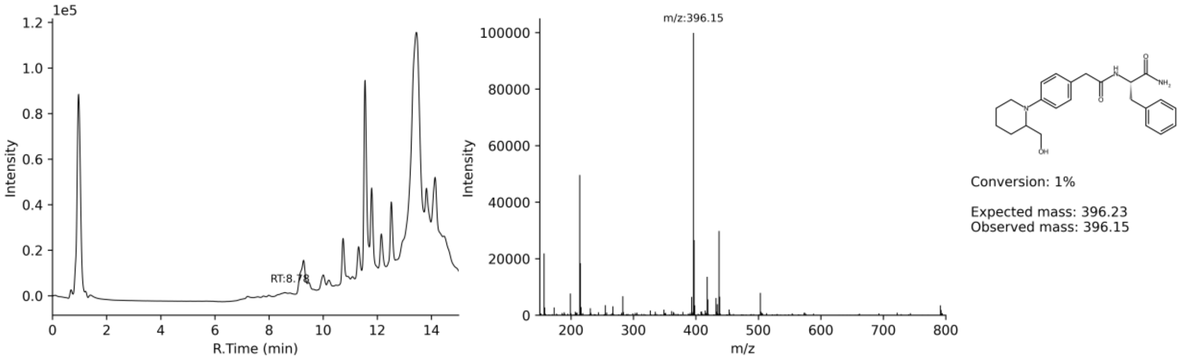

Fig. S207. LC-MS chromatogram of compound 10ak.

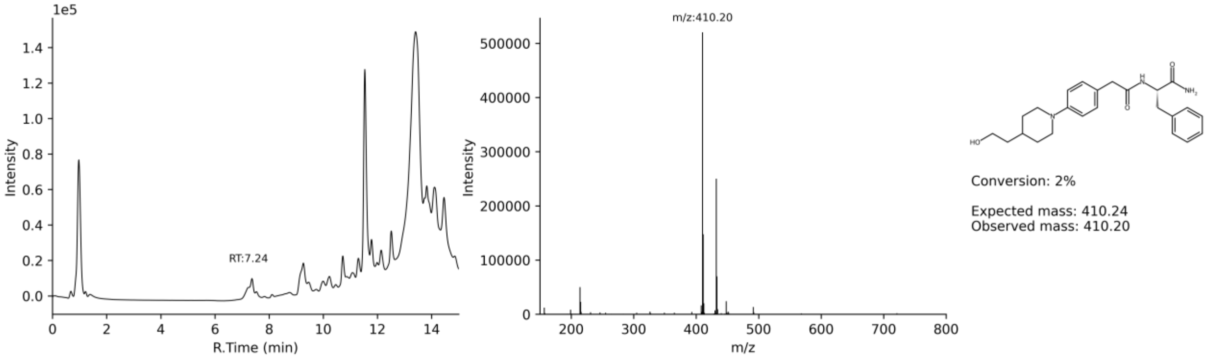

Fig. S208. LC-MS chromatogram of compound 10al.

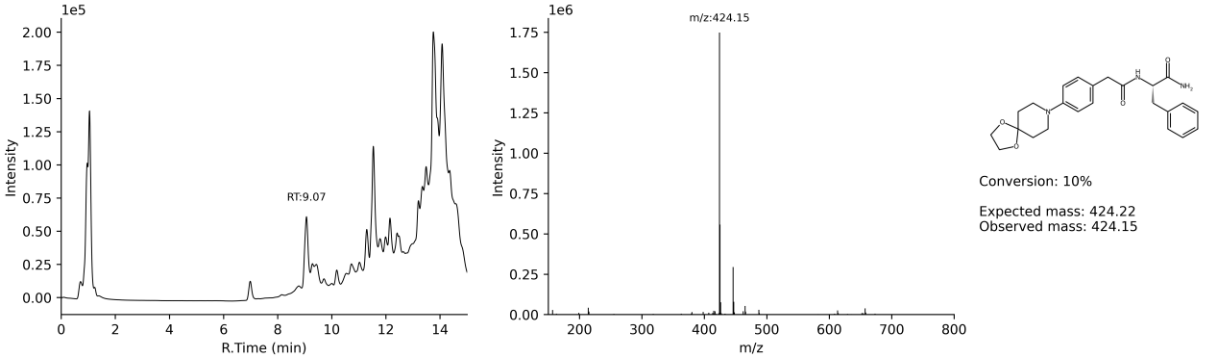

Fig. S209. LC-MS chromatogram of compound 10am.

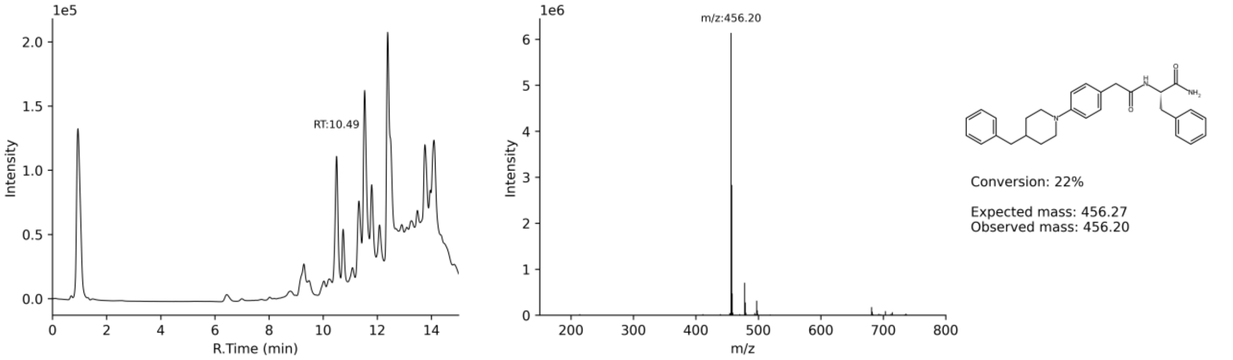

Fig. S210. LC-MS chromatogram of compound 10an.

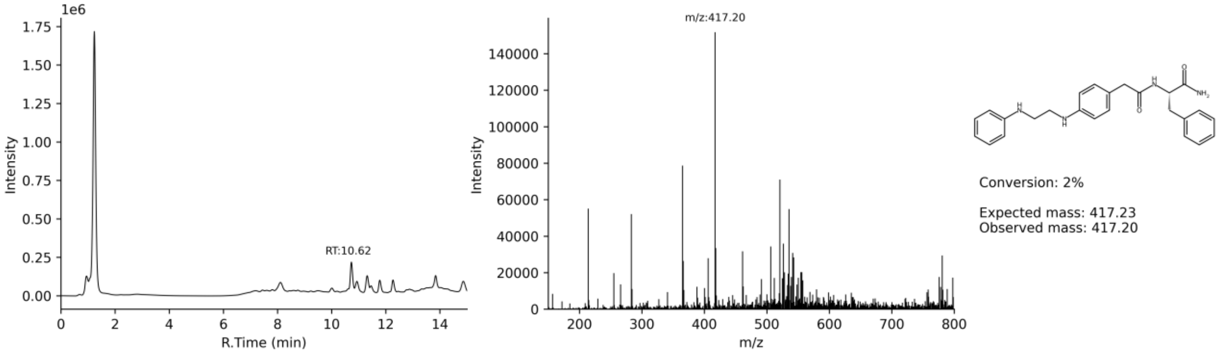

Fig. S211. LC-MS chromatogram of compound 10ao.

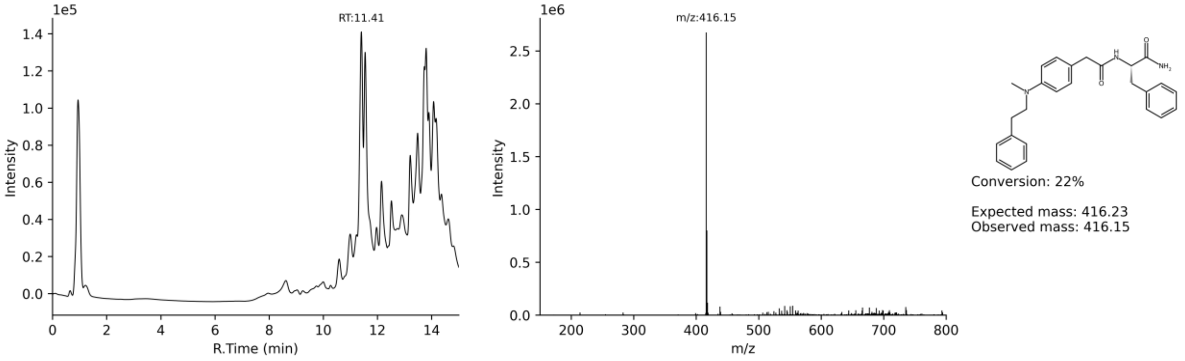

Fig. S212. LC-MS chromatogram of compound 10ap.

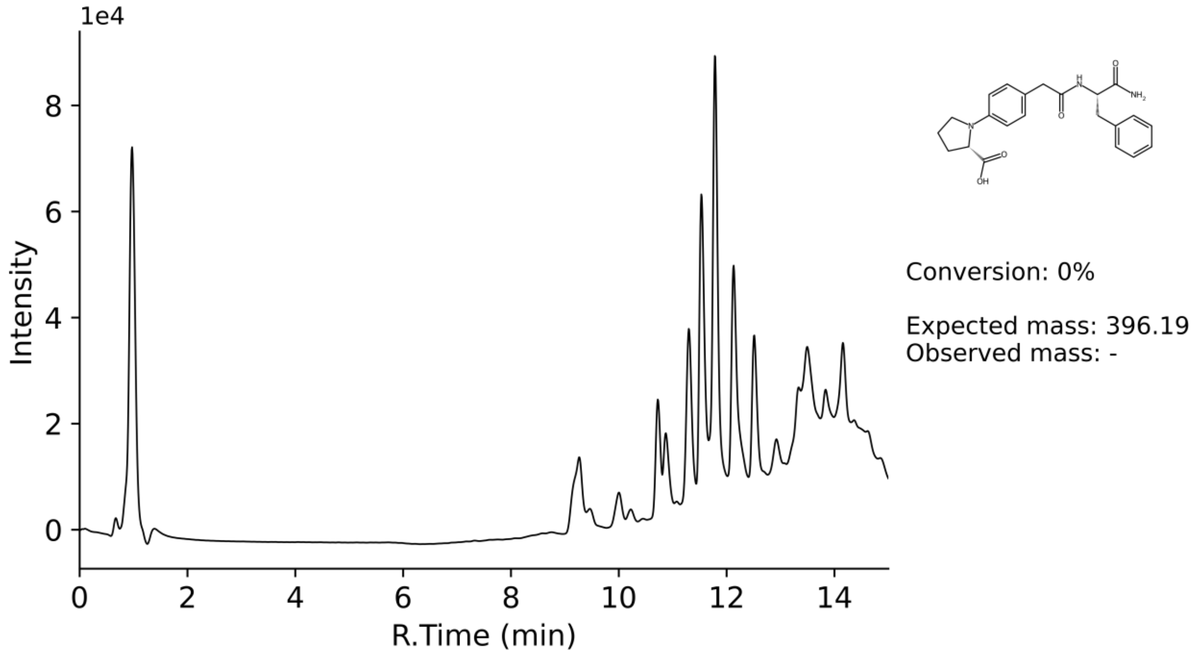

Fig. S213. LC-MS chromatogram of compound 10aq.

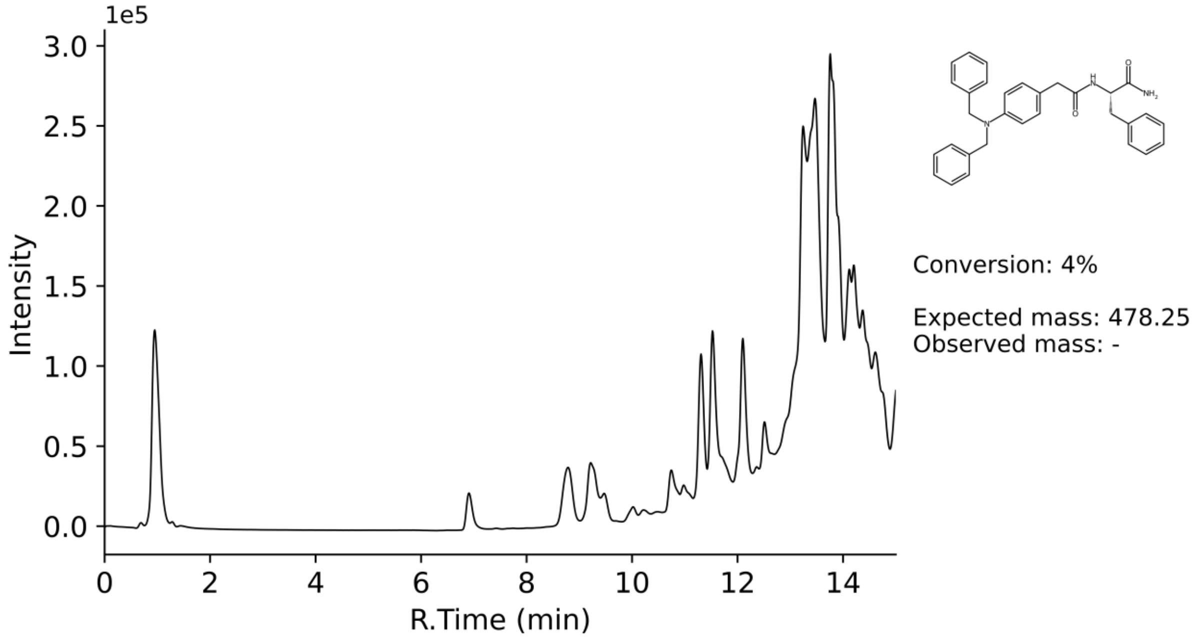

Fig. S214. LC-MS chromatogram of compound 10ar.

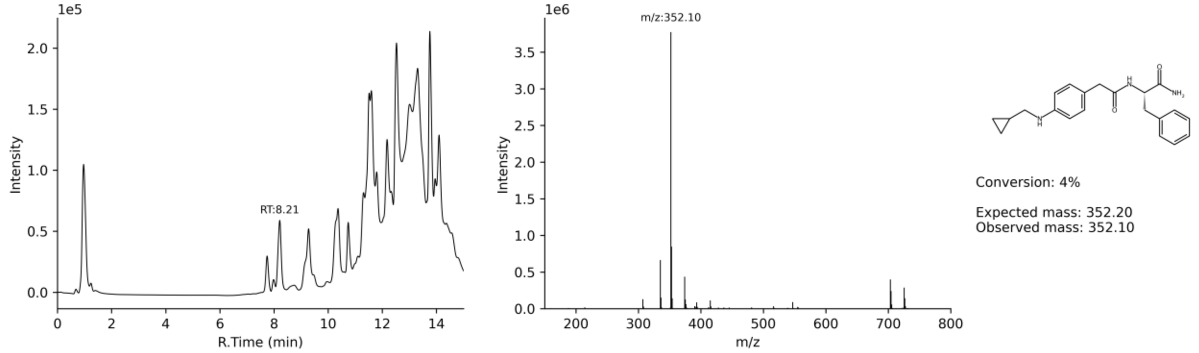

Fig. S215. LC-MS chromatogram of compound 10as.

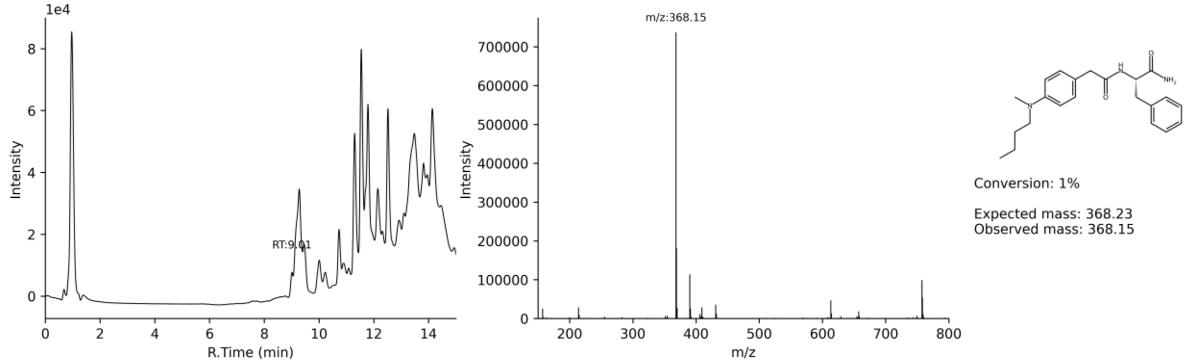

Fig. S216. LC-MS chromatogram of compound 10at.

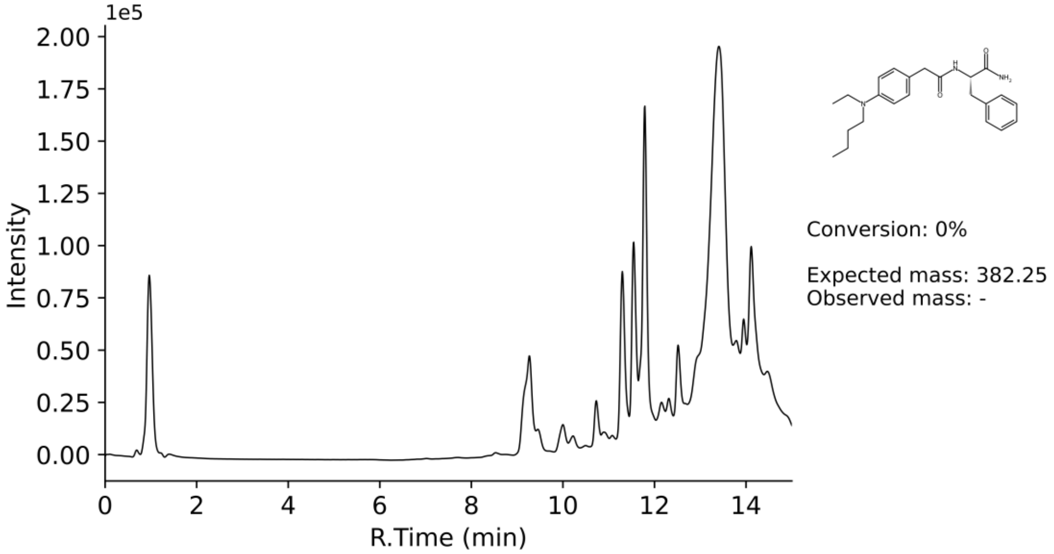

Fig. S217. LC-MS chromatogram of compound 10au.

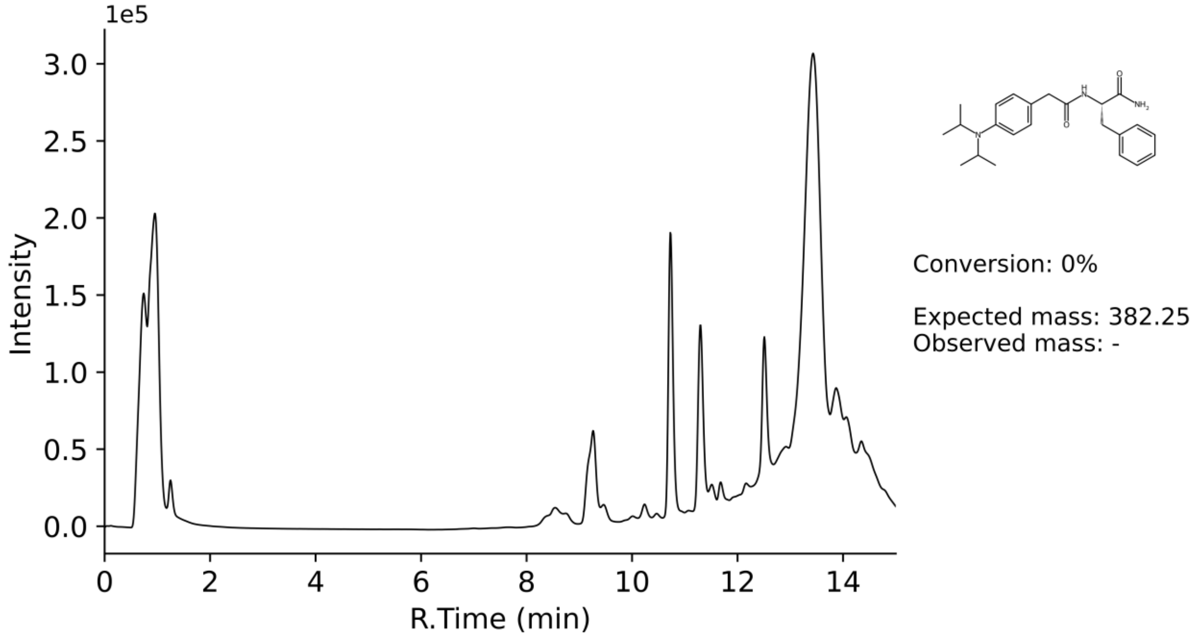

Fig. S218. LC-MS chromatogram of compound 10av.

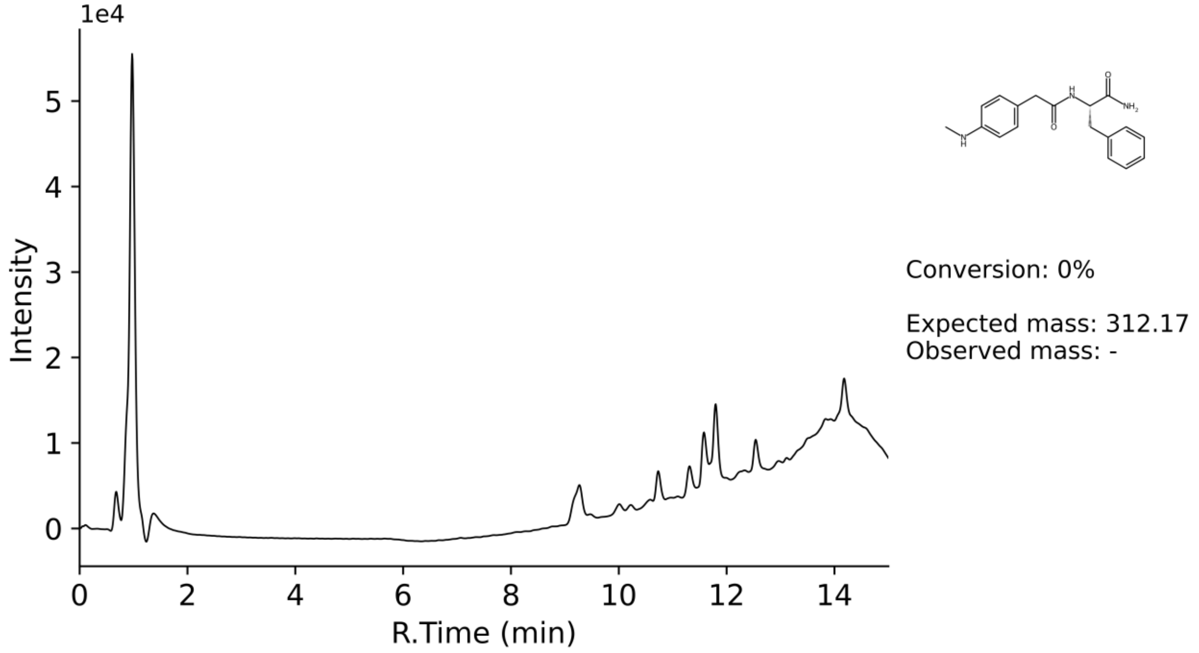

Fig. S219. LC-MS chromatogram of compound 10aw.

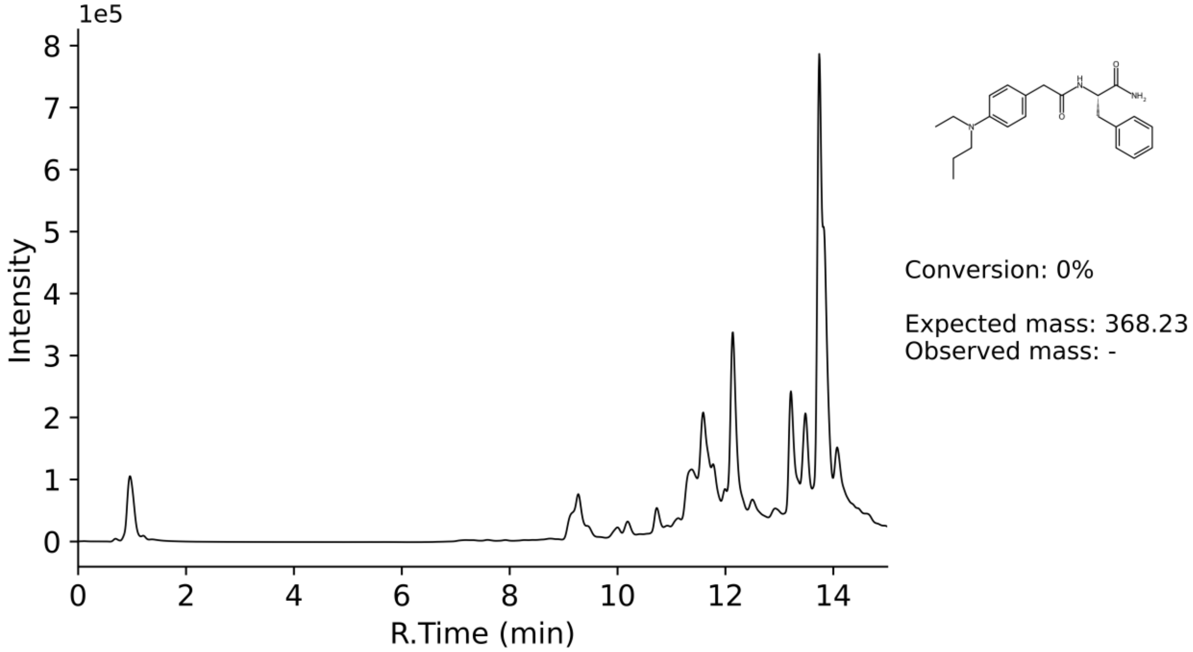

Fig. S220. LC-MS chromatogram of compound 10ax.

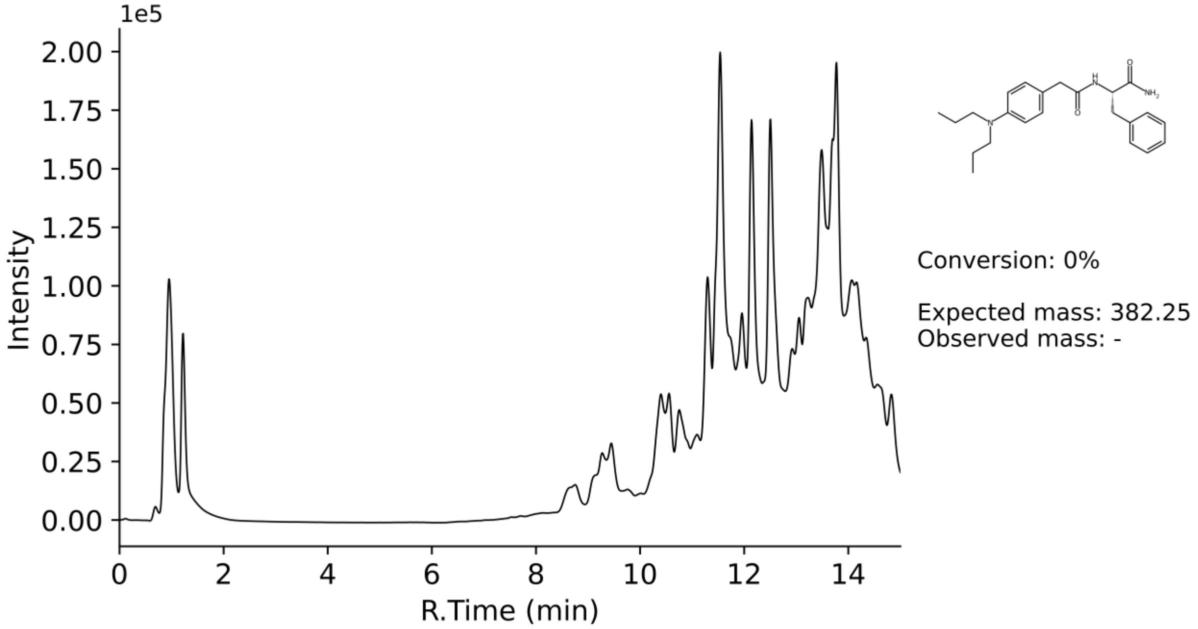

Fig. S221. LC-MS chromatogram of compound 10ay.

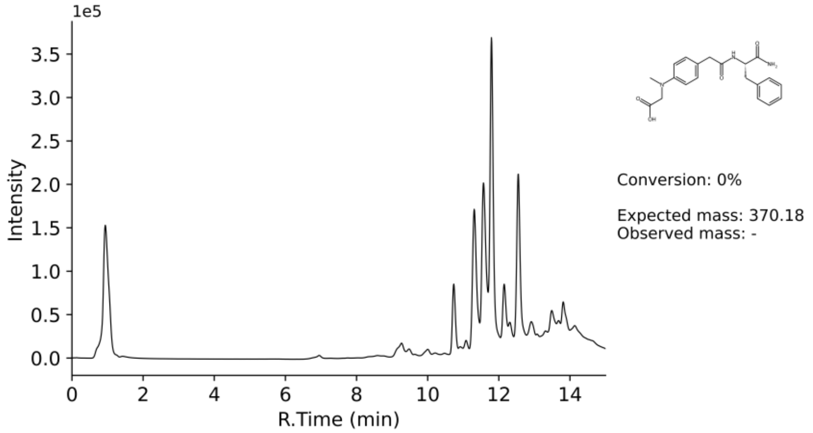

Fig. S222. LC-MS chromatogram of compound 10az.

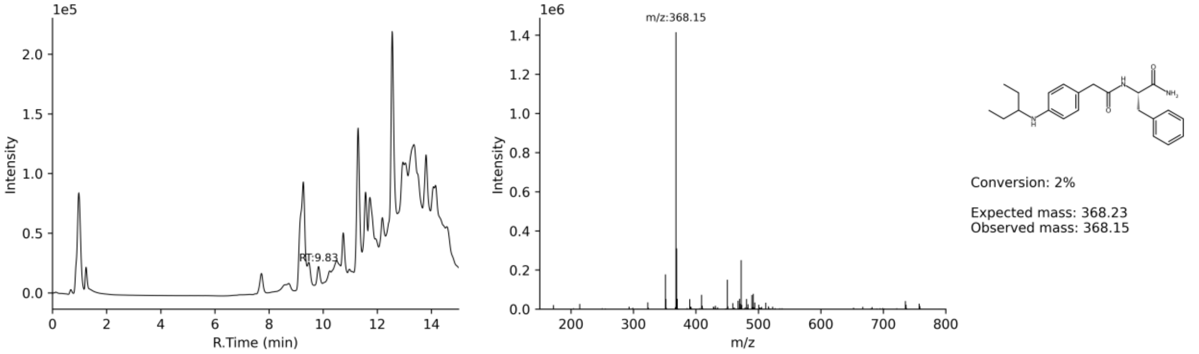

Fig. S223. LC-MS chromatogram of compound 10ba.

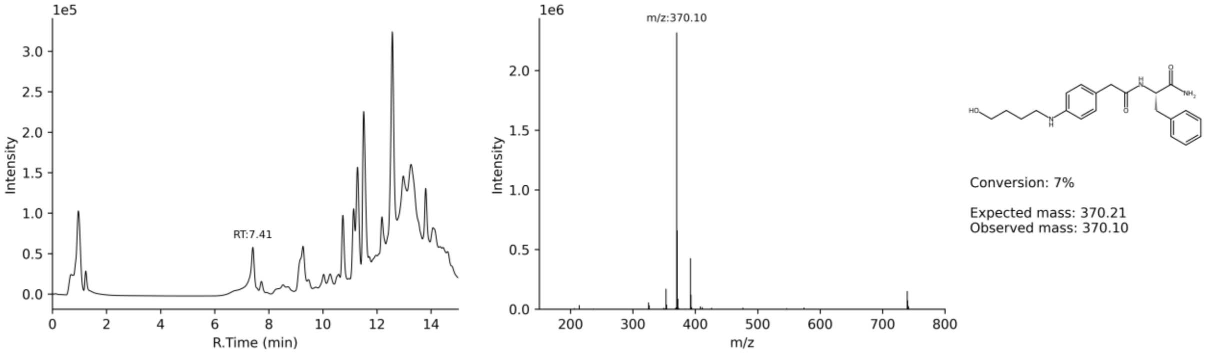

Fig. S224. LC-MS chromatogram of compound 10bb.

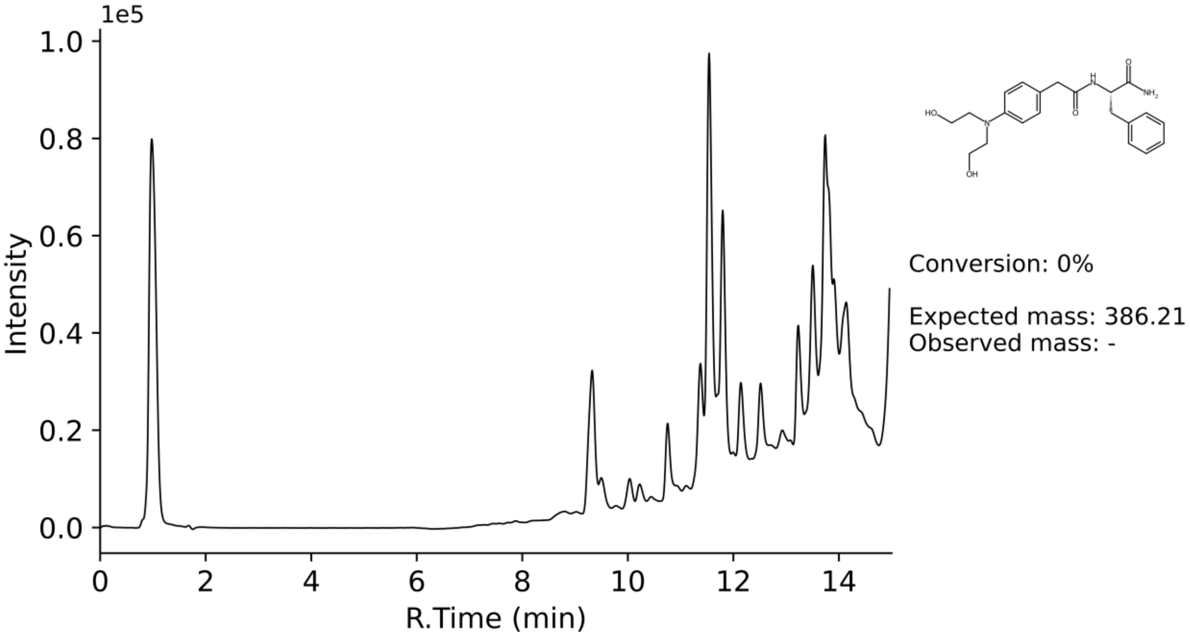

Fig. S225. LC-MS chromatogram of compound 10bc.
